# Coalescent-Based Genome Analyses Resolve the Early Branches of the Euarchontoglires

**DOI:** 10.1371/journal.pone.0060019

**Published:** 2013-04-01

**Authors:** Vikas Kumar, Björn M. Hallström, Axel Janke

**Affiliations:** 1 Biodiversity and Climate Research Centre (BiK-F) and Senckenberg Gesellschaft für Naturforschung, Frankfurt am Main, Germany; 2 Goethe University Frankfurt, Institute for Ecology, Evolution and Diversity, Frankfurt am Main, Germany; 3 Science for Life Laboratory, Royal Institute of Technology, Stockholm, Sweden; University of Uppsala, Sweden

## Abstract

Despite numerous large-scale phylogenomic studies, certain parts of the mammalian tree are extraordinarily difficult to resolve. We used the coding regions from 19 completely sequenced genomes to study the relationships within the super-clade Euarchontoglires (Primates, Rodentia, Lagomorpha, Dermoptera and Scandentia) because the placement of Scandentia within this clade is controversial. The difficulty in resolving this issue is due to the short time spans between the early divergences of Euarchontoglires, which may cause incongruent gene trees. The conflict in the data can be depicted by network analyses and the contentious relationships are best reconstructed by coalescent-based analyses. This method is expected to be superior to analyses of concatenated data in reconstructing a species tree from numerous gene trees. The total concatenated dataset used to study the relationships in this group comprises 5,875 protein-coding genes (9,799,170 nucleotides) from all orders except Dermoptera (flying lemurs). Reconstruction of the species tree from 1,006 gene trees using coalescent models placed Scandentia as sister group to the primates, which is in agreement with maximum likelihood analyses of concatenated nucleotide sequence data. Additionally, both analytical approaches favoured the Tarsier to be sister taxon to Anthropoidea, thus belonging to the Haplorrhine clade. When divergence times are short such as in radiations over periods of a few million years, even genome scale analyses struggle to resolve phylogenetic relationships. On these short branches processes such as incomplete lineage sorting and possibly hybridization occur and make it preferable to base phylogenomic analyses on coalescent methods.

## Introduction

Improvements of phylogenetic methods and the availability of numerous placental mammalian genomes provide an invaluable resource to investigate controversial relationships with increasingly larger datasets and refined methods. The first phylogenomic studies utilising protein-coding sequences of mammals were optimistic to fully resolve the placental mammalian tree [1], [2]. However, some of the branches proved to be difficult to resolve even with these increasingly gene and taxon rich datasets and some relationships did not hold up to closer inspection [3]–[5]. Revisiting such groups using phylogenomic and network analyses provided new insights into placental mammalian evolution [4], [5]. One placental mammalian group with uncertain phylogenetic relationships among its orders is Euarchontoglires. This clade has been intensively studied through the use of concatenated genomic sequence data, retroposon insertion analyses, and multi-locus analyses of genomic data [6]–[9]. While some studies agree and give convincing support for internal branches [3], [4], [8], [9], the major difference was the placement of the tree shrews relative to primates and Glires. In phylogenomic analyses a grouping with the primates was preferred, but the alternatives could not formally be rejected [3], [4]. The conflicting results of their relationships in earlier studies makes the Euarchontoglires an interesting group to revisit with a significantly increased genomic sequence data set using a multi-locus analysis approach. Recent coalescent-based analyses seem to have solved the question of resolving Euarchontoglires [9], with results being congruent with previous phylogenomic studies [3].

Euarchontoglires is a super-clade of placental mammals that includes primates (apes, monkeys and allies), rodents (mouse, rat and guinea pig), lagomorphs, (rabbit and hares), dermopterans (flying lemurs) and scandentians (tree shrew), and has recently been established exclusively through molecular analyses [6], [10]. The original proposal of the Archonta clade [11] initially included Chiroptera (bat and flying foxes) and then Macroscelidea (elephant shrews), but these groups were later moved to other parts of the placental mammalian tree [12]–[14]. While the signal from molecular data for the monophyly of Euarchontoglires is strong, some details of the relationships within this clade remain ambiguous. In particular, the position of Scandentia differed in the numerous molecular studies, from being sister group to the primates [6], [8], [9], [14], [15], sister group to lagomorphs [16], [17], sister group to Glires (rodents plus lagomorphs) [4], [18] to being the first ordinal branch among the Euarchontoglires [19]. One large phylogenomic analysis using concatenated data from 2.9 million nucleotides (16 species) could reject a sister group relationship of Scandentia to primates plus Glires, but not Scandentia and primates as sister groups as an alternative to a first divergence among Euarchontoglires [4]. The branches of the placental mammalian tree that have been problematic to resolve are typically short with subsequent divergences occurring within 1–3 million years (Myr) [4], [20]. This duration is approximately the time-span establish a mammalian species [21]–[23] and a time frame in which introgression can occur and incomplete lineage sorting can later complicate phylogenomic analyses [24]. Speciation processes appear to be the main reason for the incongruent results of phylogenomic attempts to resolve the mammalian species tree.

Therefore, we revisit the Euarchontoglires with a new and larger set of genomic sequence data focusing on ordinal relationships within the Euarchontoglires along with the relationship of the tarsiers (haplorhine primates) relative to the other two major primate lineages, Strepsirrhini (lemurs, lorises and allies) and Anthropoidea (platyrrhines and catarrhines). The widely used concatenation method to infer the species tree can mislead inferences, especially when gene trees are in the so-called anomaly zone [25]. In addition to this, species trees generated using concatenated data can sometimes have very high bootstrap support values for incorrect relationships [26]. Coalescent theory tries to trace back alleles to the most recent common ancestor [27]. Recently developed tree reconstruction methods such as STAR, species tree based on ranks of coalescent [28] and MP-EST, maximum pseudo-likelihood estimates method [29] estimates a species tree from multiple gene trees. STAR and MP-EST methods can be applied to large data sets because of their faster and simpler algorithms. They provide reliable results in reasonable computational time compared to more computationally intensive and exact Bayesian species tree methods (such as BEST), which are preferable for smaller data sets [28].

Compared to recent phylogenomic studies [4], [9], [30] the new dataset now includes sequences of the Gibbon genome (*Nomascus leucogenys*). Genome data of the cow (*Bos taurus*) is used to root the tree. Among the possible outgroups to the Euarchontoglires [4] the bovine genome is arguably the one that has the best sequence coverage and annotation. The root is especially crucial for the coalescence-based species tree analyses such as STAR and MP-EST. Choosing a well-assembled genome of a closely related species facilitates the alignment and maximizes the amount of data that can be utilized. The placement of Dermoptera is also uncertain and previous studies have placed this order with the primates [7] or with Scandentia [6]. However, until a genome of this order is available, their position in the mammalian tree cannot be studied by phylogenomic approaches.

## Materials and Methods

The coding sequences (CDS) for the 19 species that are included in this study, listed in Table 1, were retrieved using Biomart from the Ensembl version 65 (http://www.ensembl.org/biomart/martview/). The procedure for alignment and phylogenomic analyses were similar to that previously described [4]. Therefore it is only briefly described here, detailing only additional procedures.

**Table 1:** List of species included in the study and the percent coverage of alignment.

Common name	Binomial name	Order	Coverage of alignment (%)
Chimpanzee	*Pan troglodytes*	Primates	95.4
Human	*Homo sapiens*	Primates	99.9
Gorilla	*Gorilla gorilla*	Primates	92.6
Orangutan	*Pongo abelii*	Primates	91.5
Gibbon	*Nomascus leucogenys*	Primates	94.0
Macaque	*Macaca mulatta*	Primates	89.9
Marmoset	*Callithrix jacchus*	Primates	92.3
Tarsier	*Tarsius syrichta*	Primates	66.2
Bushbaby	*Otolemur garnettii*	Primates	93.6
Mouse lemur	*Microcebus murinus*	Primates	71.2
Tree shrew	*Tupaia belangeri*	Scandentia	82.5
Mouse	*Mus musculus*	Rodentia	96.5
Rat	*Rattus norvegicus*	Rodentia	88.6
Kangaroo rat	*Dipodomys ordii*	Rodentia	70.2
Guinea pig	*Cavia porcellus*	Rodentia	91.5
Squirrel	*Ictidomys tridecemlineatus*	Rodentia	54.4
Pika	*Ochotona princeps*	Lagomorpha	71.6
Rabbit	*Oryctolagus cuniculus*	Lagomorpha	82.7
Cow	*Bos taurus*	Artiodactyla	94.5

Coverage of alignment is the percent sequence coverage in 9,799,170 nucleotide long alignment.

Human sequences longer than 300 nucleotides were used to find the orthologs from 18 different species using the recursive BLAST approach [3]. Only genes represented by at least 16 out of 19 species were kept for further analyses. All sequences were translated into corresponding amino acids and aligned using MAFFT version 6.833b [31]. Any alignment showing an overall nucleotides difference larger than 25% between any two species were discarded to ensure a conservative approach and further reduce the potential for incorrect alignments.

It has been shown that the quality of multiple sequence alignment is essential in phylogenetic inference [32]. Therefore, we used BMGE, Block Mapping and Gathering with Entropy, [33] that utilizes similarity matrices such as BLOSUM and PAM to remove ambiguously aligned regions. We used the option of stringent trimming based on the scoring scheme BLOSUM 95. The selected amino acid alignments were then back translated to nucleotide-alignments. Both types of sequence data were analysed.

Base composition analysis was done using the Treefinder (TF) version of March 2011 [34] to test for the compositional equilibrium of the bases across the species. For this we applied the sliding window approach using the default size of 500.

It is prohibitive to run the model-test for large genomic scale data sets because of the large computational demands [3]. Similar phylogenomic studies [3], [5] estimated the preferred model to be GTR [35] and WAG2000 [36] with 4 gamma rate categories (4G) for nucleotide and amino acids respectively. These models were also estimated for a smaller dataset of 1,006 loci using the model test of TF. Assuming the remaining data set to have similar properties, these models were used subsequently for all maximum-likelihood (ML) analyses. Initially concatenated data sets for both nucleotide and amino acid sequences were used to infer the species tree using TF, reconstructing a ML tree using the GTR and WAG2000 models, respectively, and 4 gamma rate categories (4G) as rate heterogeneity parameter. ML hypothesis testing of different topologies for Scandentia and the tarsier within the Euarchontoglires were done with the approximately unbiased test (AU) [37] and Shimodaira- Hasegawa (SH) [38], as implemented in TF. These analyses are consistent with previous phylogenomic studies, thereby enabling comparisons to previous results.

In the second step we analysed the nucleotide data using coalescent methods. We constructed the nucleotide species tree using the coalescent model of evolution implemented in the STAR method and with the MP-EST method implemented as an R package in Phybase [39]. The STAR method is motivated by multispecies coalescent model [40], which assumes deep coalescence to be the major factor for the differences between the gene trees and species tree. STAR uses the rank of coalescence to coalesce the gene trees into species tree. For this analysis each taxon must be represented by a sequence for each gene. From the 5,875 gene data set, 1,006 gene alignments fulfilled this criterion. We also constructed the concatenated tree using the nucleotide and amino acid data.

The 1,006 (Table S1) loci were used for the species tree reconstruction. The longest sequence length is 25,728 base pairs and average length was 1,757 base pairs (Figure S1). Computational constraints limited the multi-locus bootstrapping [26] to 100 replicates, using the bootstrap 'mulgene' method implemented in Phybase. Each gene was bootstrapped and the combined trees (100,600) served as input for the STAR program. Individual ML gene trees were generated for each alignment using PhyML 3.0 [41] applying the GTR model with gamma distribution. From these trees, 100 re-sampled species trees were generated using the STAR method implemented in R. The species trees were rooted with the cow and finally a consensus tree was made using the “consense” module in Phylip, version 3.69 [42].

A second coalescent-based method “MP-EST”, which implements a pseudo maximum likelihood method under the coalescent model to estimate the species tree, was also used to generate a species tree. The MP-EST method has been shown to be more accurate than STAR when inferring short branches in a species tree [29]. All 1,006 gene trees were again used as input for the MP-EST method to generate the species tree. The procedures of tree construction, including the bootstrap analyses, were similar to that described for the STAR method.

We also performed network analyses to depict conflicting signal. From the 1,006 amino acid sequence alignment, individual ML trees were generated by TF. A consensus network was built from the individual gene trees using the SplitsTree4 program [43], with a threshold of 10%. In addition, we selected the best ML tree from the three alternative hypotheses regarding the position of the tree shrew. Only gene trees that were separated from the second best ML tree by an arbitrary value >0.7 standard deviations (s.d.) were retained and used for network construction. Choosing a cut-off of 0.7 s.d. allows best depicting the conflict in the networks and retains only topologies with some moderate support from single gene analyses. The three different placements of the tree shrew as earlier described were evaluated for all the three different topologies of tree shrew. A consensus network in Splits Tree4 then summarized the trees.

## Results

The final dataset consisted of 5,875 orthologous gene alignments from 19 different species, including ten primates, seven rodents and lagomorphs, one scandentian, and one outgroup species, the cow (Table 1). After trimming the alignments using BMGE [33], we generated a concatenated alignment of 9,799,170 nucleotides with an average sequence coverage of 85% for each species (Table 1). This resulted in 71% more sites than a previous phylogenomic studies that included Euarchontoglires [4]. The base composition showed high homogeneity between the species both for all codon positions (NT123) and first and second codon position alone (NT12) (Table S2, Table S3).

**Figure 1:**
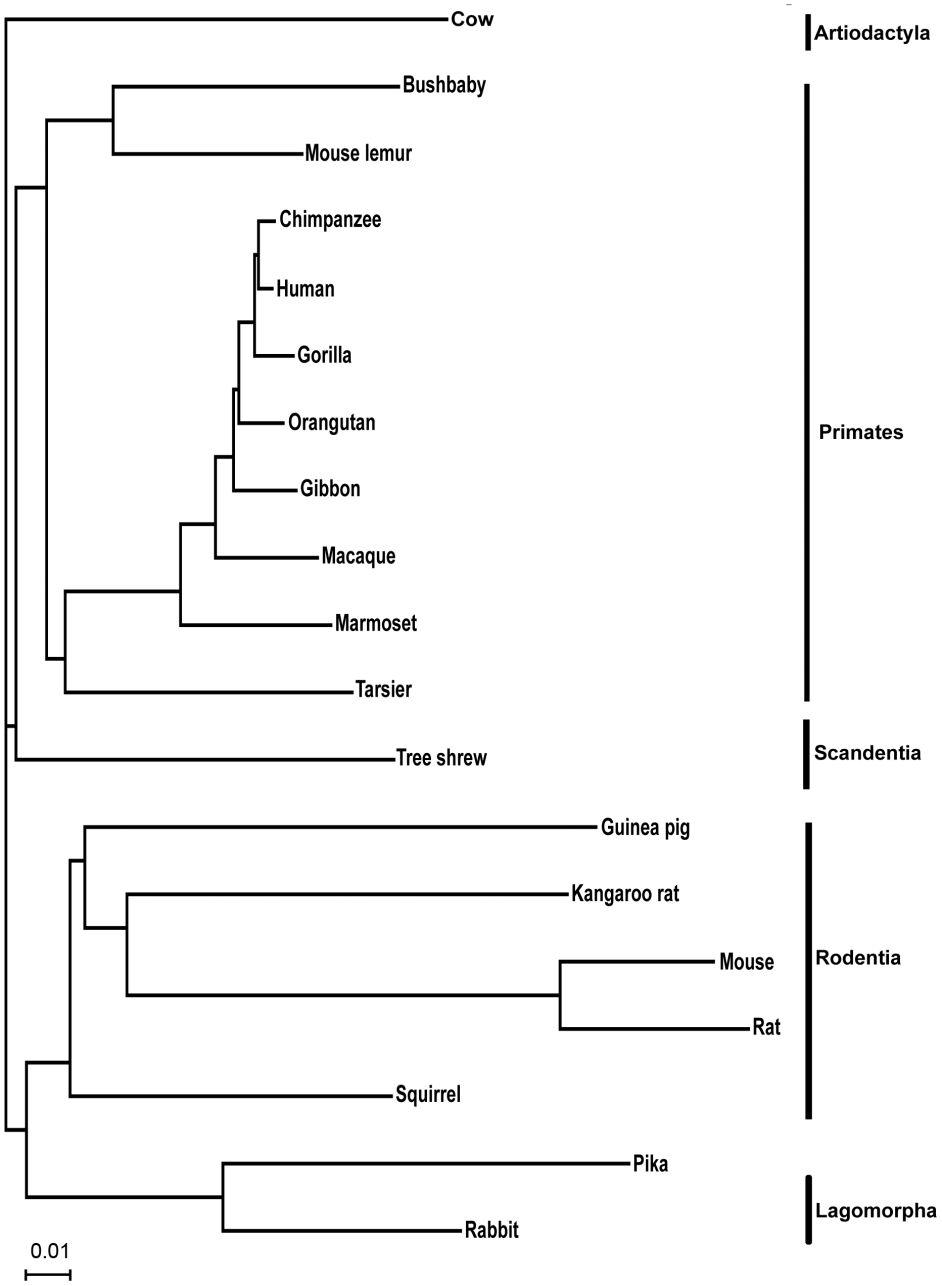
The ML tree of concatenated nucleotides data from 5,875 genes with all the branches being unanimously supported by TF.

**Figure 2:**
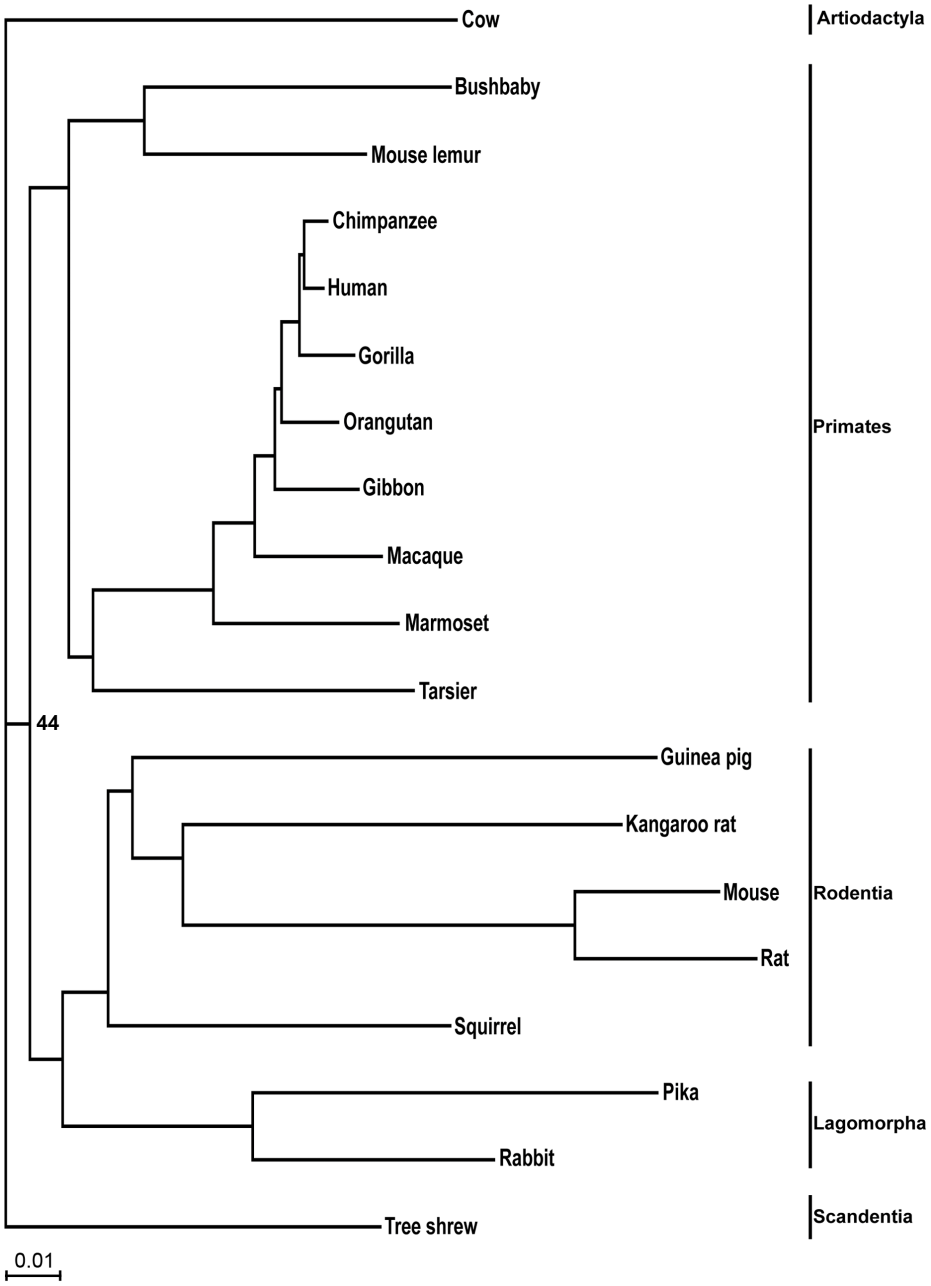
The ML tree based on amino acid data from 5,875 genes representing the best option for a bifurcating topology. Only the TF support values <99 are shown.

The ML consensus trees from concatenated amino acid and nucleotide data supported different topologies for the position of Scandentia. The nucleotide analysis supported a sister group relationship between Scandentia and the primates, with 100% support both including and excluding the third codon position (Figure 1). The amino acid ML analysis, involved 3,266,390 sites and found Scandentia as the outgroup to both the primates and Glires, albeit with negligible support (Figure 2). The three proposed hypotheses for the position of Scandentia were tested by ML analyses and the results are summarized in Table 2. The support from both the resampling and comparative likelihood tests for the position of Scandentia is ambiguous when analysing the amino acid sequences, supporting the hypothesis of Scandentia either as outgroup to primates or to Glires. Yet, nucleotide data analyses unambiguously support the Scandentia and Primate sister group relationship both when tested with all the three codon positions and first two codon positions.

**Table 2:** ML tests statistics for different relationships of the Scandentia within the Euarchontoglires.

Topologies	pSH (AA)	pAU (AA)	pSH (NT12)	pAU (NT12)	pSH (NT123)	pAU (NT123)
**((Scandentia,Primates),Glires)**	0.23	<0.001	1	0.11	1	0.23
**((Scandentia,Glires),Primates)**	<0.001	<0.001	<0.001	<0.001	<0.001	<0.001
**(Scandentia,(Primates,Glires))**	1	0.94	<0.001	<0.001	<0.001	<0.001

pSH (probability Shimodaira Hasegawa) and pAU (probability Approximate Unbiased) ML test values are shown.

**Figure 3:**
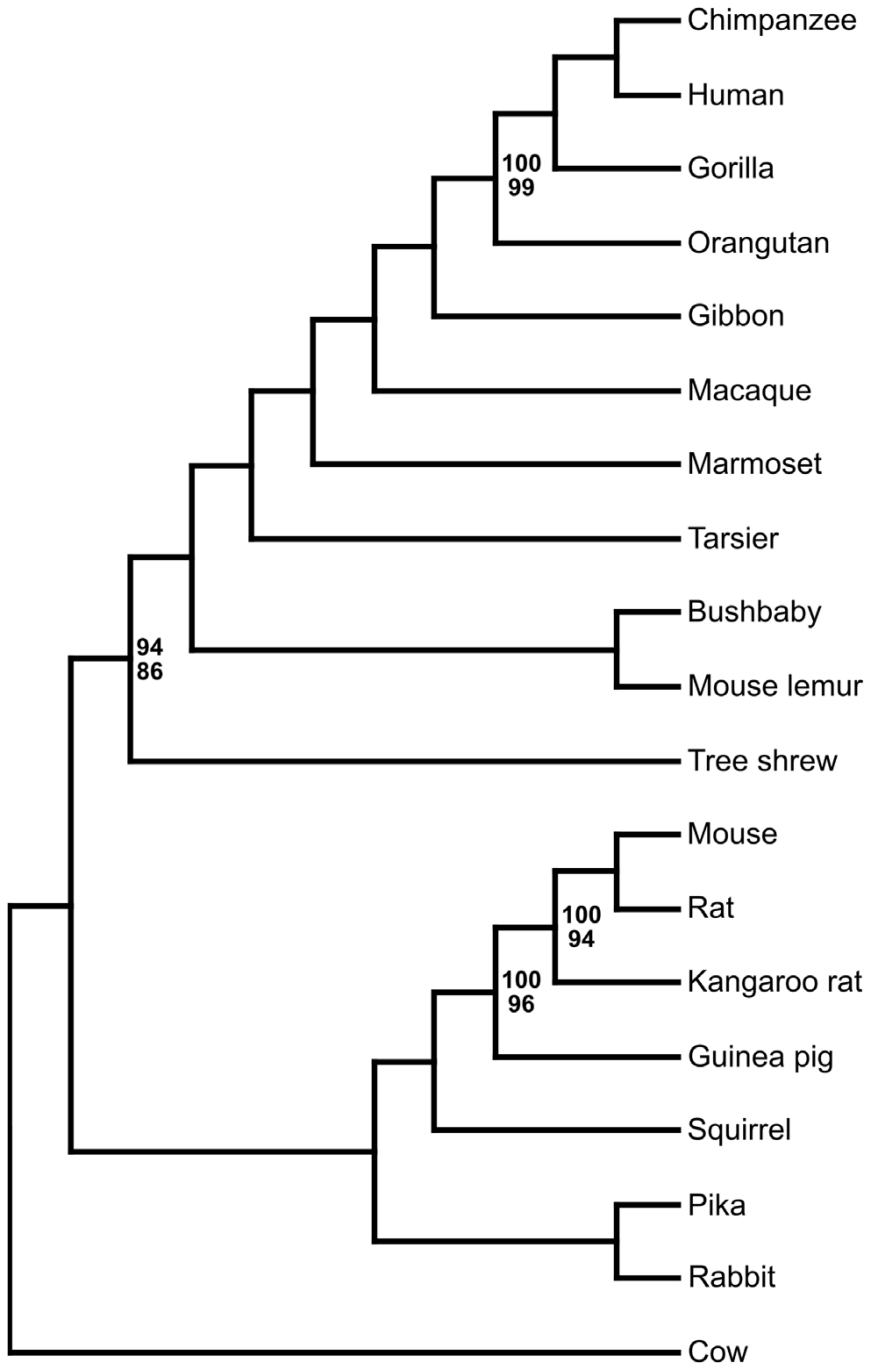
Species tree based on 1,006 gene trees with bootstrap support values (>99% not shown). Above value indicates the STAR support value and MP-EST values are indicated below.

The concatenated tree analysis of the nucleotide sequence data from 1,006 genes was congruent with the analysis of the 5,875 genes, but lacked strong support most likely due to the reduced amount of data. The species tree topology constructed by STAR, using the 1,006 gene trees, supported the concatenated nucleotide consensus species tree using the 5,875 loci (Figure 3). The bootstrap support for the Scandentia-primates relationship was 94%. MP-EST yielded the same topology for the species tree with a bootstrap support of 86% for the Scandentia-primates branch (Figure 3). Thus, both coalescent multi gene analyses of nucleotide sequences support the Scandentia-primates relationship with high bootstrap support values.

The position of the tarsier within Euarchontoglires is established with high support in all analyses. The tarsier is placed as sister taxon to the Simiformes with both the concatenated nucleotide and amino acid data analysis (Figure 1, 2). Statistical ML analyses of the three different hypotheses for the position of the tarsier and for all types of sequences data reject alternative positions (Table 3). Also, the coalescent based methods supported the same topology as the concatenated analysis with 100% bootstrap support, suggesting the tarsier as sister taxon to the Simiiformes (Figure 3).

**Table 3:** ML tests statistics for different relationships of the tarsier within the Euarchontoglires.

Topologies	pSH (AA)	pAU (AA)	pSH (NT12)	pAU (NT12)	pSH (NT123)	pSH (NT123)
**((Tarsier,Anthropoidea),Strepsirrhini)**	1	0.23	1	0.23	1	0.23
**((Tarsier,Strepsirrhini),Anthropoidea)**	<0.001	<0.001	<0.001	<0.001	<0.001	<0.001
**(Tarsier,(Anthropoidea,Strepsirrhini))**	<0.001	<0.001	<0.001	<0.001	<0.001	<0.001

pSH (probability Shimodaira Hasegawa) and pAU (probability Approximate Unbiased) ML test values are shown.

**Figure 4:**
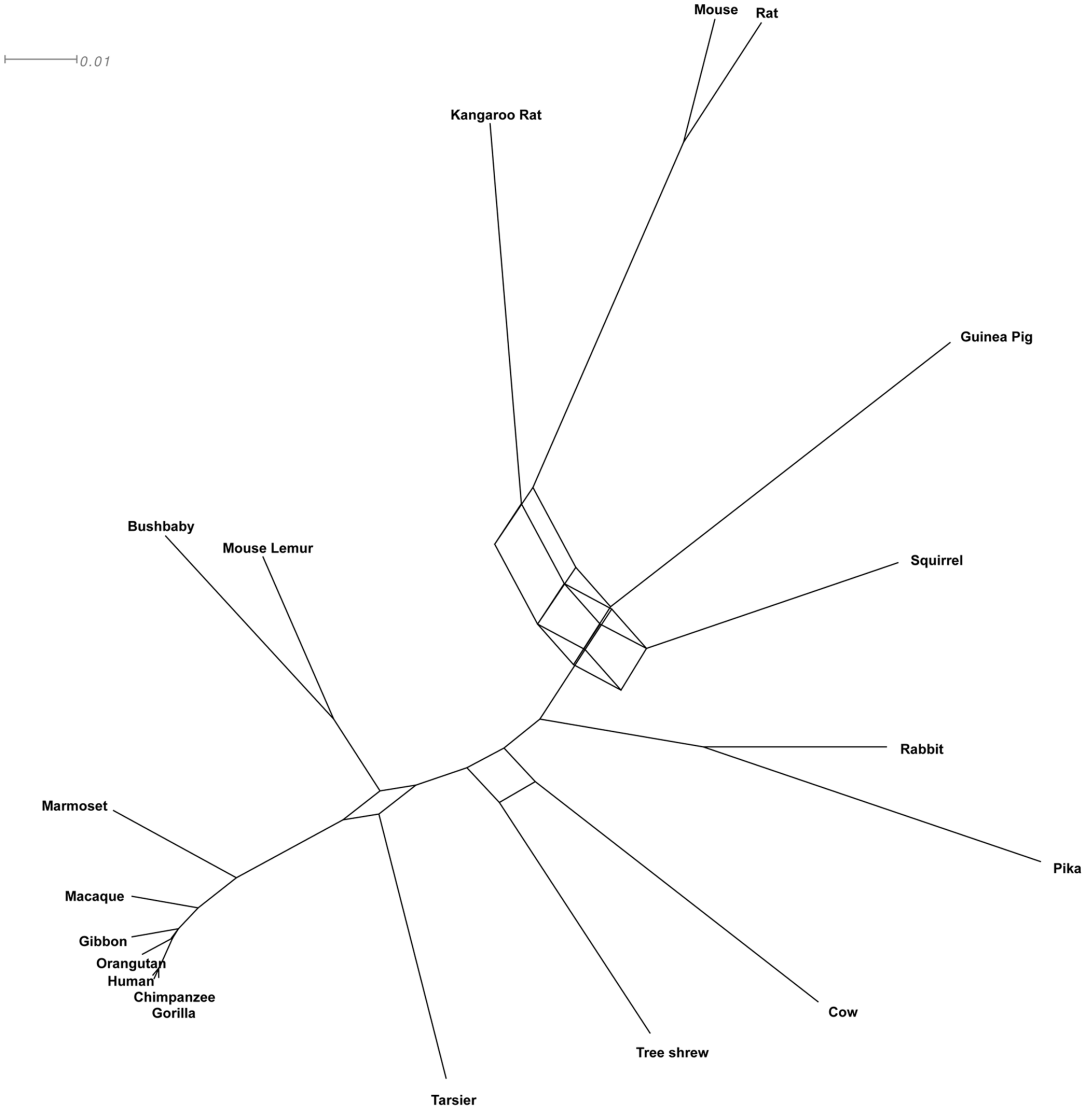
Consensus network in which at least 10% of the 1006 ML gene trees have common branches (threshold value 10%).

The consensus network analysis of all 1,006 genes that are represented in all species shows conflicting branches when limited to splits that are present in at least 10% of the data (10% threshold value) shown in Figure 4. The major signal from this single gene analyses places the tree shrew as sister group to the primates, or basal to all Euarchontoglires. Depicting the position of the tree shrew in a network based on the best ML trees from three alternative hypotheses of the tree shrew position, yielded no further resolution or insight into the evolutionary process (Figure S2).

## Discussion

The increase in the availability of complete mammalian genomes has been seen as an opportunity to fully resolve all branches in the mammalian tree. Yet, numerous studies using genome scale data [3]–[5], [44] yielded the insight that species tree reconstruction is complicated by incomplete lineage sorting and possibly introgression. One major group that was problematic to resolve is the Euarchontoglires clade. In many studies the branches connecting Scandentia and tarsier to the tree were found to be difficult to place within this super-order. The reason for this is that short internal branches have been identified to be a major cause of uncertainty in most phylogenomic studies [3], [4], [45]. By increasing the taxon sampling and taking advantage of coalescent-based analytical approaches this study investigated the relationships within Euarchontoglires in more detail and with new analytical approaches.

It has been shown that in multi-locus data analysis, gene tree heterogeneity and the conflicts arising due to different gene trees complicate the process of correctly inferring the species tree [24]. Gene duplication, incomplete lineage sorting and deep coalescence are obstacles in correctly inferring the species tree. Also, the entire process of inferring the species tree becomes more complicated as the amount of multi-locus data increases. To avoid analytical problems due to gene duplication we selected strictly orthologous genes. For getting a comprehensive representation of the ML tree, we began the analysis with the normal concatenation method, which has been used in numerous studies of mammalian evolution [1], [2], [4], [9], [44]. The resulting ML tree supported the general consensus that Scandentia is the sister group to primates. Thus, focusing on the resolution of the Euarchontoglires and increasing the dataset, yielded robust results [6], [9], [15]. Only the analysis of amino acid sequences failed to provide the statistical support for the position of Scandentia. Yet, testing different hypotheses for the position of Scandentia yielded high support from nucleotide sequences for the topology shown in Figure 1 and 3.

It has been debated [46]–[49] whether nucleotide or amino acid data contains more reliable phylogenetic information. Generally, the use of amino acid data is advised, because amino acid sequences are expected to be less randomized than nucleotide sequences for ancient divergences [48]. However, by using a set of selected genes it has been shown that nucleotide sequence data can outperform the amino acid sequence data for phylogenetic information on time scales of less than 500 million years [48]. In this study we analyse time scales in the range of 80–90 Ma and with short divergence intervals, where coalescence and introgression can complicate phylogenetic analysis. Under these conditions amino acid sequence data may be too conserved to contain sufficient phylogenetic information.

It has been observed that the approach of using concatenated sequences under certain conditions can obscure important phylogenetic signal [50]. Incongruent gene-trees can mislead phylogenetic analyses of concatenated sequences and result in erroneous interpretations of the species relationships and sometimes the incorrect species trees receive high support values [51]. The solution to this is the analysis of individual genes and their associated evolutionary signal (gene-tree) in a coalescence based framework to recover a final species tree [40]. STAR [28] is one such method for species tree reconstruction and has been successfully used for studying the phylogeny of placental mammals from protein coding genes [9] and from ultra conserved element sequences within mammals [45]. Similar to a recent phylogenomic study using multi-locus analyses [9], albeit with more than twice as much data and using a strict approach to identify orthologs, we find a clear support of Scandentia being the sister group to primates. The large data set used in this study found the same species tree in the multi locus STAR analysis as in the concatenated analysis from nucleotide sequence data. Thus, analyses of large concatenated data sets can yield the same phylogenetic results as multi-locus analyses. Congruence between the approaches increases the confidence to have identified the historic species tree, despite the conflict in individual gene analyses that is revealed by networks. For theoretical reasons a full multi-locus coalescent analysis had been preferable, but this approach is still prohibitive for large datasets. The dramatic increase in data was only possible by focussing on the phylogeny of the Euarchontoglires phylogeny and choice of a well sequenced outgroup.

To further consolidate the results obtained from the STAR analysis we employed MP-EST analysis [29] as an additional coalescent-based method. It has been shown to be equally reliable as STAR [28], but uses a pseudo-likelihood method in the environment of coalescence theory. The results from MP-EST are congruent with the STAR method, yielding identical topology with high support values. Both methods, STAR and MP-EST require more data to reconstruct confident species trees, because they use a partial parametric method and summary statistics [28], [29]. However, compared to fully parametric methods, STAR and MP-EST allow analysing large and taxon-rich datasets within reasonable time. In contrast to the congruence of the concatenated and multi-locus coalescent analyses, the analysis of a smaller data set (447 genes) on the whole mammalian tree [9] found differences in the two approaches. The Scandentia grouped with the primates using multi-locus analyses, a result favoured by the authors for the new methodology [9]. The concatenated analyses grouped the Scandentia with Rodentia [9], however, this grouping that has been rejected previously [3], [4].

Regardless of the analytical approach, conflict in phylogenetic data needs to be shown either by careful ML analyses of alternative trees or by the phylogenetic signal from single gene analyses. The phylogenetic signal of conflicting data from single gene trees can be ideally depicted by network analyses [52]. The network depicts the previous difficulties to resolve the relationship of the Scandentia by sequence analyses even from concatenating genome sequence data [4], [5], [9] with nearly equally long edges, but no connection to the rodents. Interestingly, retroposon insertion analyses have so far yielded a clear signal [8] with no conflicting data for this branch. In comparison, in other studies of deep mammalian divergences some splits were problematic to resolve from this data as bifurcating tree, because of conflicting signal [4], [5], [53], suggesting that incomplete lineage sorting and/or hybridization obscure short branches [4], [53]. It remains to be shown, if conflicting retroposon insertions are present for Euarchontoglires. However, the high evolutionary rate in the rodents will make it difficult to study neutral sequences like that of retroposon insertions in further detail, because sequence similarity in rodents is highly eroded over the 80 million years of their evolution [4].

Another challenging to resolve relationship has been the tarsier’s grouping with anthropoids (platyrrhines and catarrhines) or Strepsirrhini (Lemuriformes and Lorisiforms). It has remained controversial, because contrasting phylogenetic signals from molecular data support different topologies [30]. This conflict is also visible in the network analysis (Figure 4). Our coalescent based analyses, however, confirm tarsier as sister taxon to the Simiiformes [30], which together with the Tarsiiformes form the Haplorhini clade. This relationship was identified by analyses of concatenated data with unanimous support.

### Conclusions

The presently largest data set for a multi-locus analyses of mammalian relationships resolved the long challenge of placing Scandentia as the sister group to the primates, as has been previously suggested [6], [9], [15]. Multi-locus analyses settled the grouping of the tarsier with anthropoids. This leaves the dermopterans as the last order to be placed in the euarchontogliran tree. New mammalian genomes, and further development of methods will soon finalize the ordinal relationships among the Euarchontoglires.

Network analyses are a valuable tool to depict and evaluate conflict in gene trees that can only be identified in genome-scale phylogenetic analyses. These conflicts from multiple gene trees can now be resolved into a reliable species tree by recent implementations of coalescence-based methods into phylogenetic analysis programs [28], [29]. The necessity of using methods developed for population genetics for deep divergences is a surprising development, because higher-level relationships have been expected to be deeper than the coalescent times of most genes. However, phylogenomic studies have shown that this is not always the case and speciation related processes interfere with phylogenetic analyses [3]–[5]. While the use of concatenated sequences generally improves the resolution of the phylogenetic tree, the current development in mammalian evolutionary studies [9] show that this approach may in some cases be uncertain and multi-locus species tree analyses are preferred to yield a reliable and sound species phylogeny even for divergences as deep as that among mammalian orders.

## Supporting Information

Figure S1 **Length Distribution of the 1006 longest gene trees.**
(TIF)

Figure S2 **Consensus Network of selected 661 genes.** Gene selected on basis of supporting the best topology of tree shrew position with a LogL value larger than standard deviation of >0.7 compared to best tree.
(TIF)

Table S1 **List of 1,006 loci (.xls file, Ensembl transcript id) from all the species.**
(DOC)

Table S2 **Base composition for each species for all the three nucleotide positions (NT123).**
(DOC)

Table S3 **Base Composition for each species for all the first two nucleotide positions (NT12).**
(DOC)
