# Supplementary figures and images for: Coalescent-Based Genome Analyses Resolve the Early Branches of the Euarchontoglires
Source: PLoS One. 2013 Apr 1;8(4):e60019. doi: 10.1371/journal.pone.0060019 (PMC3613385; doi:10.1371/journal.pone.0060019)

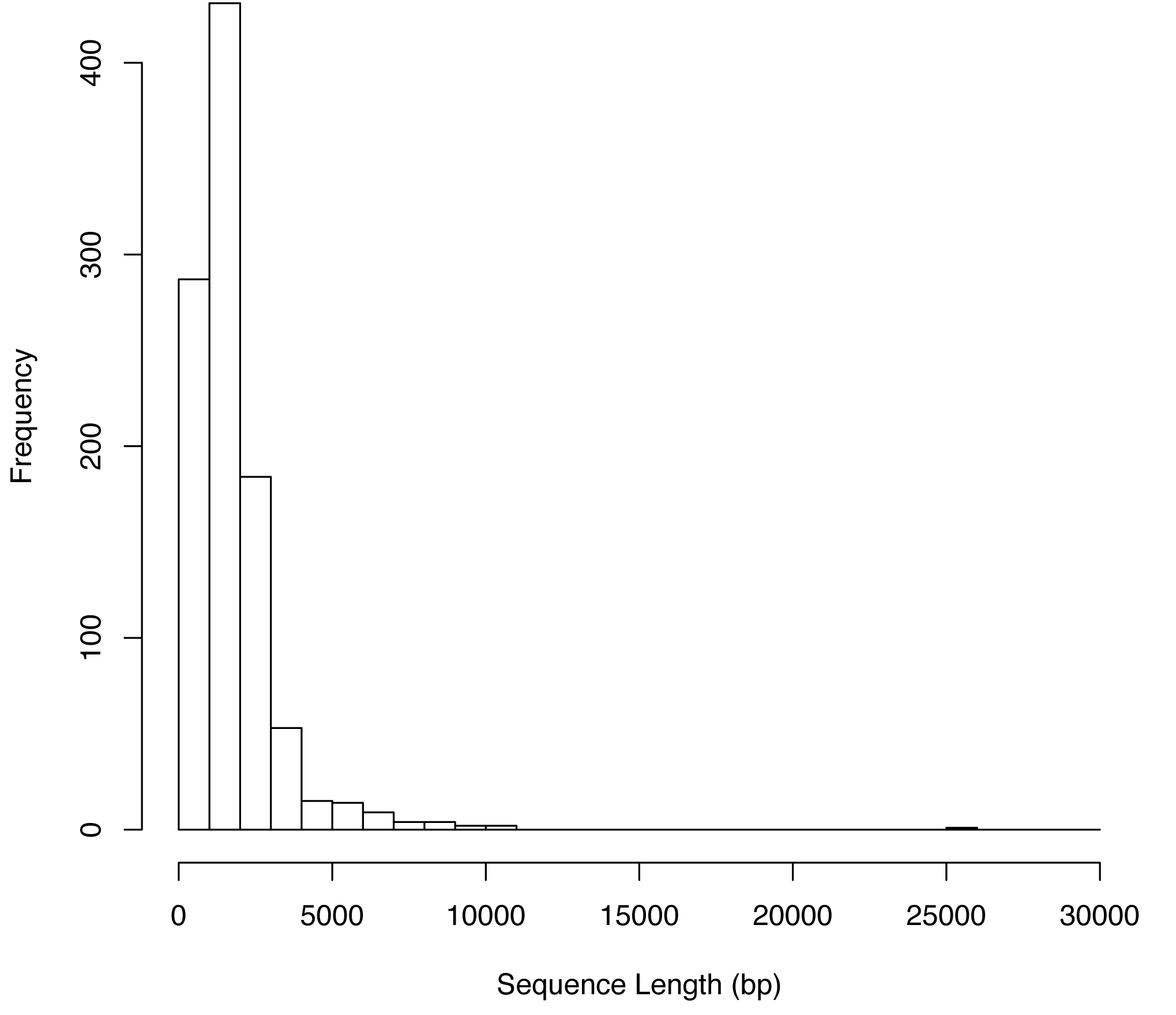

Supplement: Figure S1 — Length Distribution of the 1006 longest gene trees. (TIF) [file pone.0060019.s001.tif]

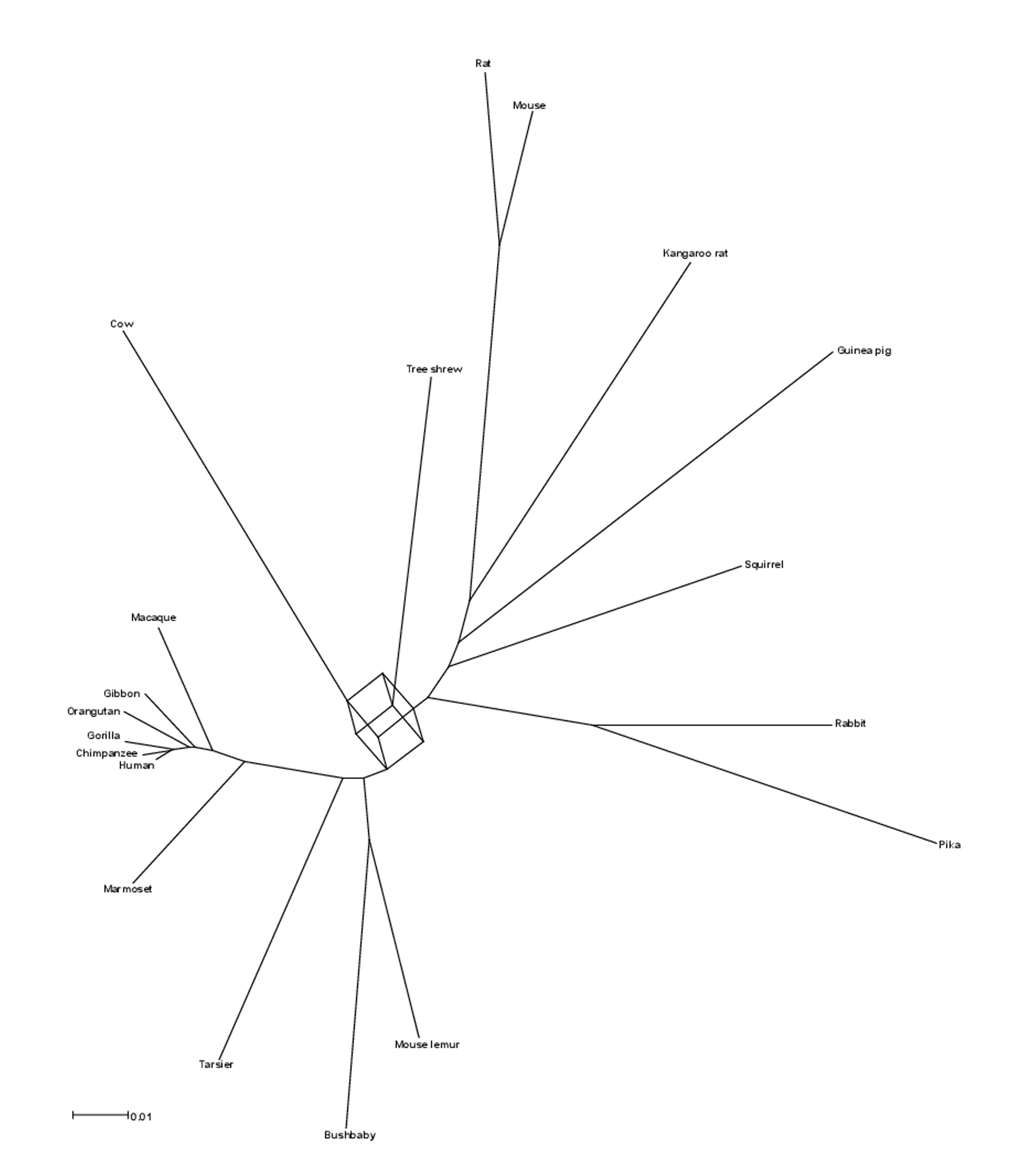

Supplement: Figure S2 — Consensus Network of selected 661 genes. Gene selected on basis of supporting the best topology of tree shrew position with a LogL value larger than standard deviation of >0.7 compared to best tree. (TIF) [file pone.0060019.s002.tif]
