# Supplementary material for: Coalescent-Based Genome Analyses Resolve the Early Branches of the Euarchontoglires
Source: PLoS One. 2013 Apr 1;8(4):e60019. doi: 10.1371/journal.pone.0060019 (PMC3613385; doi:10.1371/journal.pone.0060019)
Supplement: Table S2 — Base composition for each species for all the three nucleotide positions (NT123). (DOC) [file pone.0060019.s004.doc]

|  | **T** | **C** | **A** | **G** | **T+C** | **A+G** |
| --- | --- | --- | --- | --- | --- | --- |
| **Bushbaby** | 0.233 | 0.245 | 0.268 | 0.253 | 0.479 | 0.521 |
| **Chimpanzee** | 0.234 | 0.243 | 0.271 | 0.252 | 0.477 | 0.522 |
| **Cow** | 0.225 | 0.254 | 0.261 | 0.260 | 0.479 | 0.521 |
| **Gibbon** | 0.235 | 0.243 | 0.271 | 0.252 | 0.478 | 0.522 |
| **Gorilla** | 0.233 | 0.245 | 0.269 | 0.252 | 0.478 | 0.521 |
| **Guinea Pig** | 0.227 | 0.252 | 0.262 | 0.258 | 0.479 | 0.520 |
| **Human** | 0.233 | 0.244 | 0.270 | 0.253 | 0.477 | 0.523 |
| **Kangaroo rat** | 0.229 | 0.250 | 0.265 | 0.256 | 0.480 | 0.520 |
| **Macaque** | 0.234 | 0.244 | 0.270 | 0.252 | 0.478 | 0.522 |
| **Marmoset** | 0.235 | 0.243 | 0.270 | 0.252 | 0.478 | 0.522 |
| **Mouse Lemur** | 0.224 | 0.255 | 0.261 | 0.260 | 0.479 | 0.521 |
| **Mouse** | 0.227 | 0.251 | 0.263 | 0.259 | 0.479 | 0.521 |
| **Orangutan** | 0.233 | 0.244 | 0.270 | 0.253 | 0.478 | 0.522 |
| **Pika** | 0.218 | 0.268 | 0.253 | 0.267 | 0.480 | 0.520 |
| **Rabbit** | 0.221 | 0.257 | 0.259 | 0.263 | 0.478 | 0.522 |
| **Rat** | 0.226 | 0.252 | 0.263 | 0.258 | 0.479 | 0.521 |
| **Squirrel** | 0.235 | 0.243 | 0.271 | 0.250 | 0.479 | 0.521 |
| **Tree Shrew** | 0.228 | 0.251 | 0.266 | 0.256 | 0.478 | 0.522 |
| **Tarsier** | 0.241 | 0.234 | 0.280 | 0.244 | 0.475 | 0.525 |
| **Average** | **0.230** | **0.248** | **0.266** | **0.255** | **0.478** | **0.522** |
