# Supplementary material for: Coalescent-Based Genome Analyses Resolve the Early Branches of the Euarchontoglires
Source: PLoS One. 2013 Apr 1;8(4):e60019. doi: 10.1371/journal.pone.0060019 (PMC3613385; doi:10.1371/journal.pone.0060019)
Supplement: Table S3 — Base Composition for each species for all the first two nucleotide positions (NT12). (DOC) [file pone.0060019.s005.doc]

|  | **T** | **C** | **A** | **G** | **T+C** | **A+G** |
| --- | --- | --- | --- | --- | --- | --- |
| **Bushbaby** | 0.226 | 0.231 | 0.297 | 0.246 | 0.457 | 0.543 |
| **Chimpanzee** | 0.225 | 0.230 | 0.298 | 0.246 | 0.456 | 0.544 |
| **Cow** | 0.224 | 0.234 | 0.294 | 0.249 | 0.457 | 0.543 |
| **Gibbon** | 0.226 | 0.230 | 0.298 | 0.246 | 0.456 | 0.544 |
| **Gorilla** | 0.226 | 0.231 | 0.297 | 0.246 | 0.457 | 0.543 |
| **Guinea Pig** | 0.225 | 0.234 | 0.294 | 0.248 | 0.458 | 0.542 |
| **Human** | 0.225 | 0.231 | 0.298 | 0.246 | 0.456 | 0.544 |
| **Kangaroo rat** | 0.224 | 0.234 | 0.295 | 0.247 | 0.458 | 0.542 |
| **Macaque** | 0.226 | 0.231 | 0.297 | 0.246 | 0.457 | 0.543 |
| **Marmoset** | 0.226 | 0.231 | 0.298 | 0.246 | 0.456 | 0.544 |
| **Mouse Lemur** | 0.223 | 0.235 | 0.293 | 0.249 | 0.457 | 0.543 |
| **Mouse** | 0.224 | 0.233 | 0.295 | 0.248 | 0.457 | 0.543 |
| **Orangutan** | 0.225 | 0.231 | 0.298 | 0.246 | 0.456 | 0.544 |
| **Pika** | 0.221 | 0.238 | 0.290 | 0.251 | 0.459 | 0.541 |
| **Rabbit** | 0.222 | 0.234 | 0.293 | 0.250 | 0.457 | 0.543 |
| **Rat** | 0.224 | 0.233 | 0.295 | 0.248 | 0.457 | 0.543 |
| **Squirrel** | 0.226 | 0.231 | 0.298 | 0.244 | 0.457 | 0.543 |
| **Tree Shrew** | 0.224 | 0.233 | 0.296 | 0.247 | 0.457 | 0.543 |
| **Tarsier** | 0.228 | 0.225 | 0.305 | 0.242 | 0.453 | 0.547 |
| **Average** | **0.225** | **0.232** | **0.296** | **0.247** | **0.457** | **0.54** |
