# Supplementary material for: Coalescent-Based Genome Analyses Resolve the Early Branches of the Euarchontoglires
Source: PLoS One. 2013 Apr 1;8(4):e60019. doi: 10.1371/journal.pone.0060019 (PMC3613385; doi:10.1371/journal.pone.0060019)
Supplement: Supplementary file 4 [file pone.0060019.s003.doc]

## Sheet: Sheet1

All 1006 Loci under study 
Human	Bushbaby	Chimpanzee	Cow	Gibbon	Gorilla	Guinea pig	Kangaroo rat	Macaque	Marmoset	Mouse	Mouse lemur	Orangutan	Pika	Rabbit	Rat	Squirrel	Tree shrew	Tarsier
ENST00000335420	ENSOGAT00000005539	ENSPTRT00000036477	ENSBTAT00000000718	ENSNLET00000016228	ENSGGOT00000031521	ENSCPOT00000003718	ENSDORT00000006508	ENSMMUT00000013032	ENSCJAT00000020592	ENSMUST00000068259	ENSMICT00000009167	ENSPPYT00000020988	ENSOPRT00000000743	ENSOCUT00000001698	ENSRNOT00000013654	ENSSTOT00000015395	ENSTBET00000015528	ENSTSYT00000006674
ENST00000265404	ENSOGAT00000014533	ENSPTRT00000029999	ENSBTAT00000001271	ENSNLET00000011604	ENSGGOT00000000322	ENSCPOT00000001895	ENSDORT00000006168	ENSMMUT00000013117	ENSCJAT00000018789	ENSMUST00000031171	ENSMICT00000014208	ENSPPYT00000017129	ENSOPRT00000009685	ENSOCUT00000008156	ENSRNOT00000002760	ENSSTOT00000013015	ENSTBET00000016770	ENSTSYT00000014567
ENST00000331373	ENSOGAT00000003610	ENSPTRT00000037466	ENSBTAT00000004757	ENSNLET00000016553	ENSGGOT00000017092	ENSCPOT00000007037	ENSDORT00000016045	ENSMMUT00000029931	ENSCJAT00000064658	ENSMUST00000061850	ENSMICT00000006897	ENSPPYT00000021653	ENSOPRT00000003847	ENSOCUT00000007723	ENSRNOT00000019630	ENSSTOT00000006787	ENSTBET00000012021	ENSTSYT00000006373
ENST00000514480	ENSOGAT00000011801	ENSPTRT00000073581	ENSBTAT00000009462	ENSNLET00000010191	ENSGGOT00000002502	ENSCPOT00000021818	ENSDORT00000003942	ENSMMUT00000012835	ENSCJAT00000039170	ENSMUST00000094611	ENSMICT00000009484	ENSPPYT00000017248	ENSOPRT00000012379	ENSOCUT00000000366	ENSRNOT00000003431	ENSSTOT00000012954	ENSTBET00000011888	ENSTSYT00000013152
ENST00000413266	ENSOGAT00000008529	ENSPTRT00000010165	ENSBTAT00000020937	ENSNLET00000020158	ENSGGOT00000025576	ENSCPOT00000012448	ENSDORT00000006157	ENSMMUT00000045497	ENSCJAT00000002389	ENSMUST00000112097	ENSMICT00000008681	ENSPPYT00000005957	ENSOPRT00000004300	ENSOCUT00000014881	ENSRNOT00000001549	ENSSTOT00000012569	ENSTBET00000009474	ENSTSYT00000006137
ENST00000263864	ENSOGAT00000012283	ENSPTRT00000022557	ENSBTAT00000033040	ENSNLET00000002516	ENSGGOT00000004969	ENSCPOT00000001428	ENSDORT00000010738	ENSMMUT00000043392	ENSCJAT00000038543	ENSMUST00000059983	ENSMICT00000011477	ENSPPYT00000014191	ENSOPRT00000001941	ENSOCUT00000002372	ENSRNOT00000017301	ENSSTOT00000005953	ENSTBET00000001161	ENSTSYT00000009907
ENST00000277632	ENSOGAT00000008071	ENSPTRT00000004259	ENSBTAT00000013714	ENSNLET00000015845	ENSGGOT00000010952	ENSCPOT00000013229	ENSDORT00000005911	ENSMMUT00000033450	ENSCJAT00000017581	ENSMUST00000028105	ENSMICT00000004504	ENSPPYT00000002530	ENSOPRT00000011554	ENSOCUT00000016579	ENSRNOT00000022986	ENSSTOT00000006967	ENSTBET00000007716	ENSTSYT00000003161
ENST00000297578	ENSOGAT00000002278	ENSPTRT00000037914	ENSBTAT00000048106	ENSNLET00000004800	ENSGGOT00000023535	ENSCPOT00000012402	ENSDORT00000011870	ENSMMUT00000027254	ENSCJAT00000031464	ENSMUST00000022908	ENSMICT00000006895	ENSPPYT00000021942	ENSOPRT00000008979	ENSOCUT00000009191	ENSRNOT00000006029	ENSSTOT00000007987	ENSTBET00000015650	ENSTSYT00000003303
ENST00000263780	ENSOGAT00000004579	ENSPTRT00000028290	ENSBTAT00000010436	ENSNLET00000015035	ENSGGOT00000029760	ENSCPOT00000006658	ENSDORT00000016032	ENSMMUT00000022826	ENSCJAT00000017144	ENSMUST00000004965	ENSMICT00000009681	ENSPPYT00000015855	ENSOPRT00000013419	ENSOCUT00000003986	ENSRNOT00000000904	ENSSTOT00000011456	ENSTBET00000015982	ENSTSYT00000014034
ENST00000368977	ENSOGAT00000004122	ENSPTRT00000034143	ENSBTAT00000019425	ENSNLET00000017423	ENSGGOT00000004266	ENSCPOT00000011432	ENSDORT00000014560	ENSMMUT00000028645	ENSCJAT00000011543	ENSMUST00000041024	ENSMICT00000014297	ENSPPYT00000019662	ENSOPRT00000012263	ENSOCUT00000005863	ENSRNOT00000065669	ENSSTOT00000001121	ENSTBET00000009663	ENSTSYT00000011804
ENST00000262995	ENSOGAT00000003740	ENSPTRT00000030617	ENSBTAT00000029804	ENSNLET00000006842	ENSGGOT00000000020	ENSCPOT00000012396	ENSDORT00000015902	ENSMMUT00000001194	ENSCJAT00000005408	ENSMUST00000034150	ENSMICT00000009955	ENSPPYT00000017537	ENSOPRT00000015333	ENSOCUT00000003271	ENSRNOT00000024390	ENSSTOT00000012286	ENSTBET00000005115	ENSTSYT00000007292
ENST00000334822	ENSOGAT00000013999	ENSPTRT00000009641	ENSBTAT00000028999	ENSNLET00000006224	ENSGGOT00000030170	ENSCPOT00000012654	ENSDORT00000012438	ENSMMUT00000025647	ENSCJAT00000037537	ENSMUST00000041723	ENSMICT00000009278	ENSPPYT00000005672	ENSOPRT00000011962	ENSOCUT00000010407	ENSRNOT00000005078	ENSSTOT00000010870	ENSTBET00000017770	ENSTSYT00000007948
ENST00000258042	ENSOGAT00000011690	ENSPTRT00000034457	ENSBTAT00000061022	ENSNLET00000019202	ENSGGOT00000000880	ENSCPOT00000001729	ENSDORT00000012980	ENSMMUT00000004844	ENSCJAT00000037482	ENSMUST00000020015	ENSMICT00000001958	ENSPPYT00000019849	ENSOPRT00000004307	ENSOCUT00000001941	ENSRNOT00000016215	ENSSTOT00000006841	ENSTBET00000015265	ENSTSYT00000012211
ENST00000244061	ENSOGAT00000003955	ENSPTRT00000025365	ENSBTAT00000039156	ENSNLET00000008916	ENSGGOT00000033370	ENSCPOT00000005148	ENSDORT00000014932	ENSMMUT00000001088	ENSCJAT00000000938	ENSMUST00000109214	ENSMICT00000011152	ENSPPYT00000012914	ENSOPRT00000004722	ENSOCUT00000011224	ENSRNOT00000012675	ENSSTOT00000005719	ENSTBET00000002971	ENSTSYT00000000275
ENST00000264677	ENSOGAT00000013877	ENSPTRT00000029084	ENSBTAT00000015176	ENSNLET00000006202	ENSGGOT00000010581	ENSCPOT00000020589	ENSDORT00000001653	ENSMMUT00000013180	ENSCJAT00000057331	ENSMUST00000039047	ENSMICT00000001200	ENSPPYT00000016592	ENSOPRT00000001821	ENSOCUT00000009781	ENSRNOT00000013452	ENSSTOT00000010822	ENSTBET00000008223	ENSTSYT00000004205
ENST00000288228	ENSOGAT00000012899	ENSPTRT00000013151	ENSBTAT00000019564	ENSNLET00000014846	ENSGGOT00000015959	ENSCPOT00000015783	ENSDORT00000008942	ENSMMUT00000023010	ENSCJAT00000003805	ENSMUST00000034749	ENSMICT00000011127	ENSPPYT00000007698	ENSOPRT00000015652	ENSOCUT00000005790	ENSRNOT00000014812	ENSSTOT00000005012	ENSTBET00000006769	ENSTSYT00000002146
ENST00000552656	ENSOGAT00000001526	ENSPTRT00000009352	ENSBTAT00000038204	ENSNLET00000014058	ENSGGOT00000011161	ENSCPOT00000015036	ENSDORT00000012934	ENSMMUT00000030927	ENSCJAT00000022291	ENSMUST00000096386	ENSMICT00000014469	ENSPPYT00000005505	ENSOPRT00000001343	ENSOCUT00000010065	ENSRNOT00000029778	ENSSTOT00000003516	ENSTBET00000007359	ENSTSYT00000000953
ENST00000342988	ENSOGAT00000012083	ENSPTRT00000018403	ENSBTAT00000009081	ENSNLET00000015131	ENSGGOT00000013714	ENSCPOT00000006535	ENSDORT00000009088	ENSMMUT00000025767	ENSCJAT00000003648	ENSMUST00000025393	ENSMICT00000017525	ENSPPYT00000010703	ENSOPRT00000013596	ENSOCUT00000003931	ENSRNOT00000066857	ENSSTOT00000010602	ENSTBET00000014117	ENSTSYT00000009827
ENST00000434757	ENSOGAT00000001343	ENSPTRT00000014112	ENSBTAT00000007182	ENSNLET00000012013	ENSGGOT00000013560	ENSCPOT00000010133	ENSDORT00000000425	ENSMMUT00000039646	ENSCJAT00000029024	ENSMUST00000168410	ENSMICT00000017102	ENSPPYT00000008252	ENSOPRT00000002957	ENSOCUT00000006119	ENSRNOT00000060859	ENSSTOT00000013413	ENSTBET00000000225	ENSTSYT00000007003
ENST00000398004	ENSOGAT00000002670	ENSPTRT00000009551	ENSBTAT00000045262	ENSNLET00000004757	ENSGGOT00000032519	ENSCPOT00000004368	ENSDORT00000005182	ENSMMUT00000019907	ENSCJAT00000014602	ENSMUST00000079041	ENSMICT00000013172	ENSPPYT00000005625	ENSOPRT00000017273	ENSOCUT00000010899	ENSRNOT00000029493	ENSSTOT00000003921	ENSTBET00000013442	ENSTSYT00000013201
ENST00000228027	ENSOGAT00000000331	ENSPTRT00000007611	ENSBTAT00000001536	ENSNLET00000021504	ENSGGOT00000002925	ENSCPOT00000004457	ENSDORT00000015911	ENSMMUT00000000276	ENSCJAT00000029855	ENSMUST00000033001	ENSMICT00000015267	ENSPPYT00000004394	ENSOPRT00000000936	ENSOCUT00000014222	ENSRNOT00000022557	ENSSTOT00000012015	ENSTBET00000005180	ENSTSYT00000008480
ENST00000414170	ENSOGAT00000029271	ENSPTRT00000013134	ENSBTAT00000007206	ENSNLET00000014508	ENSGGOT00000029641	ENSCPOT00000003834	ENSDORT00000007458	ENSMMUT00000013347	ENSCJAT00000040125	ENSMUST00000164994	ENSMICT00000005868	ENSPPYT00000007688	ENSOPRT00000007378	ENSOCUT00000001646	ENSRNOT00000065883	ENSSTOT00000000548	ENSTBET00000004250	ENSTSYT00000003002
ENST00000375079	ENSOGAT00000000731	ENSPTRT00000000564	ENSBTAT00000017965	ENSNLET00000010110	ENSGGOT00000033382	ENSCPOT00000003053	ENSDORT00000010907	ENSMMUT00000007219	ENSCJAT00000012777	ENSMUST00000030533	ENSMICT00000010998	ENSPPYT00000012356	ENSOPRT00000007343	ENSOCUT00000016450	ENSRNOT00000022282	ENSSTOT00000008736	ENSTBET00000008307	ENSTSYT00000002112
ENST00000376360	ENSOGAT00000010674	ENSPTRT00000011016	ENSBTAT00000005451	ENSNLET00000009949	ENSGGOT00000006535	ENSCPOT00000002770	ENSDORT00000002578	ENSMMUT00000005030	ENSCJAT00000041230	ENSMUST00000026625	ENSMICT00000015286	ENSPPYT00000006482	ENSOPRT00000007509	ENSOCUT00000013719	ENSRNOT00000018918	ENSSTOT00000002451	ENSTBET00000002879	ENSTSYT00000004801
ENST00000272797	ENSOGAT00000003737	ENSPTRT00000023374	ENSBTAT00000001447	ENSNLET00000006310	ENSGGOT00000003762	ENSCPOT00000007617	ENSDORT00000011741	ENSMMUT00000028938	ENSCJAT00000013056	ENSMUST00000053087	ENSMICT00000004333	ENSPPYT00000015003	ENSOPRT00000009960	ENSOCUT00000010392	ENSRNOT00000010508	ENSSTOT00000001127	ENSTBET00000013160	ENSTSYT00000014518
ENST00000267436	ENSOGAT00000014289	ENSPTRT00000011584	ENSBTAT00000020226	ENSNLET00000015498	ENSGGOT00000003044	ENSCPOT00000010797	ENSDORT00000014349	ENSMMUT00000010439	ENSCJAT00000012274	ENSMUST00000021370	ENSMICT00000013196	ENSPPYT00000006851	ENSOPRT00000007680	ENSOCUT00000011421	ENSRNOT00000006473	ENSSTOT00000011887	ENSTBET00000014252	ENSTSYT00000011944
ENST00000262441	ENSOGAT00000000984	ENSPTRT00000016130	ENSBTAT00000006662	ENSNLET00000012919	ENSGGOT00000008575	ENSCPOT00000002835	ENSDORT00000000952	ENSMMUT00000025410	ENSCJAT00000024716	ENSMUST00000051765	ENSMICT00000005362	ENSPPYT00000009334	ENSOPRT00000004117	ENSOCUT00000015494	ENSRNOT00000004946	ENSSTOT00000010732	ENSTBET00000000279	ENSTSYT00000013348
ENST00000333634	ENSOGAT00000001721	ENSPTRT00000025955	ENSBTAT00000036848	ENSNLET00000016774	ENSGGOT00000005031	ENSCPOT00000003532	ENSDORT00000010101	ENSMMUT00000001599	ENSCJAT00000011023	ENSMUST00000166952	ENSMICT00000007753	ENSPPYT00000013268	ENSOPRT00000013658	ENSOCUT00000001522	ENSRNOT00000043998	ENSSTOT00000012742	ENSTBET00000007784	ENSTSYT00000006030
ENST00000558613	ENSOGAT00000013426	ENSPTRT00000013811	ENSBTAT00000014852	ENSNLET00000001090	ENSGGOT00000014934	ENSCPOT00000003777	ENSDORT00000011289	ENSMMUT00000040151	ENSCJAT00000032965	ENSMUST00000107470	ENSMICT00000007313	ENSPPYT00000008041	ENSOPRT00000014545	ENSOCUT00000006428	ENSRNOT00000018639	ENSSTOT00000004360	ENSTBET00000016552	ENSTSYT00000004513
ENST00000345732	ENSOGAT00000010169	ENSPTRT00000055547	ENSBTAT00000006236	ENSNLET00000017157	ENSGGOT00000006409	ENSCPOT00000012517	ENSDORT00000006284	ENSMMUT00000001784	ENSCJAT00000062579	ENSMUST00000025576	ENSMICT00000010978	ENSPPYT00000003835	ENSOPRT00000011615	ENSOCUT00000007941	ENSRNOT00000028433	ENSSTOT00000006740	ENSTBET00000001554	ENSTSYT00000011670
ENST00000445488	ENSOGAT00000008435	ENSPTRT00000027543	ENSBTAT00000020296	ENSNLET00000005755	ENSGGOT00000015840	ENSCPOT00000004540	ENSDORT00000008900	ENSMMUT00000028289	ENSCJAT00000037788	ENSMUST00000063042	ENSMICT00000010825	ENSPPYT00000016323	ENSOPRT00000005057	ENSOCUT00000005826	ENSRNOT00000013632	ENSSTOT00000010027	ENSTBET00000004879	ENSTSYT00000014027
ENST00000345496	ENSOGAT00000012066	ENSPTRT00000026103	ENSBTAT00000063929	ENSNLET00000002962	ENSGGOT00000004150	ENSCPOT00000024529	ENSDORT00000006247	ENSMMUT00000009459	ENSCJAT00000020504	ENSMUST00000174510	ENSMICT00000012996	ENSPPYT00000013357	ENSOPRT00000003205	ENSOCUT00000012943	ENSRNOT00000001636	ENSSTOT00000007960	ENSTBET00000017193	ENSTSYT00000001660
ENST00000409699	ENSOGAT00000007651	ENSPTRT00000013268	ENSBTAT00000036967	ENSNLET00000016440	ENSGGOT00000000297	ENSCPOT00000006533	ENSDORT00000001010	ENSMMUT00000011194	ENSCJAT00000014512	ENSMUST00000118485	ENSMICT00000004368	ENSPPYT00000007759	ENSOPRT00000004732	ENSOCUT00000014766	ENSRNOT00000058007	ENSSTOT00000015271	ENSTBET00000005917	ENSTSYT00000004997
ENST00000359541	ENSOGAT00000007160	ENSPTRT00000021905	ENSBTAT00000022977	ENSNLET00000004266	ENSGGOT00000004774	ENSCPOT00000003884	ENSDORT00000007499	ENSMMUT00000014698	ENSCJAT00000001990	ENSMUST00000088010	ENSMICT00000017124	ENSPPYT00000014585	ENSOPRT00000010771	ENSOCUT00000017063	ENSRNOT00000007868	ENSSTOT00000011361	ENSTBET00000017088	ENSTSYT00000006231
ENST00000393674	ENSOGAT00000005536	ENSPTRT00000067771	ENSBTAT00000008831	ENSNLET00000008523	ENSGGOT00000005368	ENSCPOT00000010441	ENSDORT00000010990	ENSMMUT00000041493	ENSCJAT00000009986	ENSMUST00000040218	ENSMICT00000009952	ENSPPYT00000017680	ENSOPRT00000007600	ENSOCUT00000008882	ENSRNOT00000014015	ENSSTOT00000012389	ENSTBET00000013324	ENSTSYT00000001249
ENST00000297354	ENSOGAT00000005853	ENSPTRT00000037669	ENSBTAT00000022924	ENSNLET00000001623	ENSGGOT00000015462	ENSCPOT00000011034	ENSDORT00000003658	ENSMMUT00000032648	ENSCJAT00000015045	ENSMUST00000040695	ENSMICT00000007776	ENSPPYT00000021780	ENSOPRT00000008456	ENSOCUT00000012472	ENSRNOT00000009530	ENSSTOT00000004566	ENSTBET00000011944	ENSTSYT00000003933
ENST00000394125	ENSOGAT00000034431	ENSPTRT00000056255	ENSBTAT00000020093	ENSNLET00000022116	ENSGGOT00000003265	ENSCPOT00000023834	ENSDORT00000014416	ENSMMUT00000007343	ENSCJAT00000057955	ENSMUST00000145903	ENSMICT00000002943	ENSPPYT00000022879	ENSOPRT00000012069	ENSOCUT00000016226	ENSRNOT00000025194	ENSSTOT00000011233	ENSTBET00000000806	ENSTSYT00000002645
ENST00000399054	ENSOGAT00000005308	ENSPTRT00000024631	ENSBTAT00000049460	ENSNLET00000018479	ENSGGOT00000032989	ENSCPOT00000007108	ENSDORT00000003525	ENSMMUT00000044474	ENSCJAT00000041991	ENSMUST00000028730	ENSMICT00000007129	ENSPPYT00000012442	ENSOPRT00000002724	ENSOCUT00000002869	ENSRNOT00000009540	ENSSTOT00000014171	ENSTBET00000016589	ENSTSYT00000012392
ENST00000372169	ENSOGAT00000013393	ENSPTRT00000042150	ENSBTAT00000042924	ENSNLET00000016384	ENSGGOT00000028975	ENSCPOT00000021554	ENSDORT00000002320	ENSMMUT00000025962	ENSCJAT00000035741	ENSMUST00000061578	ENSMICT00000010545	ENSPPYT00000023008	ENSOPRT00000002946	ENSOCUT00000005624	ENSRNOT00000017984	ENSSTOT00000010597	ENSTBET00000000102	ENSTSYT00000003285
ENST00000356495	ENSOGAT00000004091	ENSPTRT00000050074	ENSBTAT00000026377	ENSNLET00000001143	ENSGGOT00000010048	ENSCPOT00000009235	ENSDORT00000001830	ENSMMUT00000015697	ENSCJAT00000036221	ENSMUST00000133792	ENSMICT00000008389	ENSPPYT00000000311	ENSOPRT00000011415	ENSOCUT00000014326	ENSRNOT00000005853	ENSSTOT00000006493	ENSTBET00000013772	ENSTSYT00000009197
ENST00000264670	ENSOGAT00000015026	ENSPTRT00000045008	ENSBTAT00000020559	ENSNLET00000014550	ENSGGOT00000016641	ENSCPOT00000008271	ENSDORT00000010247	ENSMMUT00000018494	ENSCJAT00000016929	ENSMUST00000109699	ENSMICT00000016547	ENSPPYT00000017822	ENSOPRT00000011335	ENSOCUT00000007923	ENSRNOT00000023641	ENSSTOT00000004117	ENSTBET00000014725	ENSTSYT00000005716
ENST00000336431	ENSOGAT00000012971	ENSPTRT00000024940	ENSBTAT00000017688	ENSNLET00000012932	ENSGGOT00000015022	ENSCPOT00000004232	ENSDORT00000008568	ENSMMUT00000025195	ENSCJAT00000035419	ENSMUST00000029131	ENSMICT00000004909	ENSPPYT00000012698	ENSOPRT00000010934	ENSOCUT00000002254	ENSRNOT00000025315	ENSSTOT00000011605	ENSTBET00000009812	ENSTSYT00000003700
ENST00000372257	ENSOGAT00000010822	ENSPTRT00000001254	ENSBTAT00000020671	ENSNLET00000017913	ENSGGOT00000003572	ENSCPOT00000014104	ENSDORT00000005989	ENSMMUT00000016325	ENSCJAT00000007753	ENSMUST00000037127	ENSMICT00000013645	ENSPPYT00000001715	ENSOPRT00000009938	ENSOCUT00000008586	ENSRNOT00000026222	ENSSTOT00000006184	ENSTBET00000007627	ENSTSYT00000002498
ENST00000295962	ENSOGAT00000006926	ENSPTRT00000028187	ENSBTAT00000022100	ENSNLET00000009327	ENSGGOT00000015880	ENSCPOT00000001596	ENSDORT00000014648	ENSMMUT00000046489	ENSCJAT00000038790	ENSMUST00000166497	ENSMICT00000014525	ENSPPYT00000016010	ENSOPRT00000013323	ENSOCUT00000000831	ENSRNOT00000012271	ENSSTOT00000015391	ENSTBET00000010623	ENSTSYT00000013341
ENST00000304771	ENSOGAT00000005334	ENSPTRT00000007609	ENSBTAT00000001366	ENSNLET00000021517	ENSGGOT00000005427	ENSCPOT00000027818	ENSDORT00000002979	ENSMMUT00000010753	ENSCJAT00000029902	ENSMUST00000068973	ENSMICT00000015257	ENSPPYT00000004392	ENSOPRT00000007516	ENSOCUT00000003301	ENSRNOT00000034389	ENSSTOT00000015950	ENSTBET00000001618	ENSTSYT00000002841
ENST00000380871	ENSOGAT00000001304	ENSPTRT00000037208	ENSBTAT00000001615	ENSNLET00000020336	ENSGGOT00000014415	ENSCPOT00000010652	ENSDORT00000013252	ENSMMUT00000013683	ENSCJAT00000039454	ENSMUST00000022646	ENSMICT00000011185	ENSPPYT00000021495	ENSOPRT00000003163	ENSOCUT00000004290	ENSRNOT00000020753	ENSSTOT00000015081	ENSTBET00000002294	ENSTSYT00000007110
ENST00000408901	ENSOGAT00000008739	ENSPTRT00000042541	ENSBTAT00000012628	ENSNLET00000019233	ENSGGOT00000005023	ENSCPOT00000008315	ENSDORT00000019781	ENSMMUT00000020715	ENSCJAT00000014766	ENSMUST00000022815	ENSMICT00000010105	ENSPPYT00000006702	ENSOPRT00000016591	ENSOCUT00000013972	ENSRNOT00000024337	ENSSTOT00000002638	ENSTBET00000001409	ENSTSYT00000009601
ENST00000371461	ENSOGAT00000003900	ENSPTRT00000033706	ENSBTAT00000046690	ENSNLET00000004597	ENSGGOT00000014686	ENSCPOT00000024598	ENSDORT00000007643	ENSMMUT00000030088	ENSCJAT00000023647	ENSMUST00000127798	ENSMICT00000012237	ENSPPYT00000019379	ENSOPRT00000002905	ENSOCUT00000014573	ENSRNOT00000027293	ENSSTOT00000015420	ENSTBET00000012625	ENSTSYT00000000815
ENST00000371584	ENSOGAT00000005388	ENSPTRT00000025388	ENSBTAT00000010153	ENSNLET00000008941	ENSGGOT00000009033	ENSCPOT00000014379	ENSDORT00000010186	ENSMMUT00000003920	ENSCJAT00000012769	ENSMUST00000154111	ENSMICT00000003187	ENSPPYT00000012926	ENSOPRT00000001757	ENSOCUT00000014711	ENSRNOT00000016150	ENSSTOT00000014661	ENSTBET00000003787	ENSTSYT00000012096
ENST00000460849	ENSOGAT00000007712	ENSPTRT00000028152	ENSBTAT00000013237	ENSNLET00000009248	ENSGGOT00000041036	ENSCPOT00000026391	ENSDORT00000002136	ENSMMUT00000030427	ENSCJAT00000015269	ENSMUST00000100776	ENSMICT00000001004	ENSPPYT00000016032	ENSOPRT00000005025	ENSOCUT00000008043	ENSRNOT00000012390	ENSSTOT00000004740	ENSTBET00000016225	ENSTSYT00000013663
ENST00000423656	ENSOGAT00000002001	ENSPTRT00000028021	ENSBTAT00000030668	ENSNLET00000008981	ENSGGOT00000001049	ENSCPOT00000020329	ENSDORT00000009856	ENSMMUT00000006085	ENSCJAT00000010746	ENSMUST00000159645	ENSMICT00000013709	ENSPPYT00000016093	ENSOPRT00000016708	ENSOCUT00000010336	ENSRNOT00000018932	ENSSTOT00000008698	ENSTBET00000000408	ENSTSYT00000003095
ENST00000369816	ENSOGAT00000011001	ENSPTRT00000033977	ENSBTAT00000020598	ENSNLET00000015808	ENSGGOT00000009400	ENSCPOT00000006131	ENSDORT00000009079	ENSMMUT00000028429	ENSCJAT00000021190	ENSMUST00000034796	ENSMICT00000003263	ENSPPYT00000019542	ENSOPRT00000010231	ENSOCUT00000005331	ENSRNOT00000012999	ENSSTOT00000006143	ENSTBET00000017634	ENSTSYT00000007194
ENST00000234038	ENSOGAT00000000736	ENSPTRT00000024349	ENSBTAT00000001045	ENSNLET00000017594	ENSGGOT00000012855	ENSCPOT00000006350	ENSDORT00000004575	ENSMMUT00000022774	ENSCJAT00000012264	ENSMUST00000027494	ENSMICT00000017782	ENSPPYT00000015532	ENSOPRT00000002596	ENSOCUT00000003429	ENSRNOT00000022854	ENSSTOT00000016307	ENSTBET00000016785	ENSTSYT00000005964
ENST00000543196	ENSOGAT00000008004	ENSPTRT00000009150	ENSBTAT00000023861	ENSNLET00000022670	ENSGGOT00000009484	ENSCPOT00000013666	ENSDORT00000015295	ENSMMUT00000032585	ENSCJAT00000034548	ENSMUST00000159715	ENSMICT00000003536	ENSPPYT00000005368	ENSOPRT00000002633	ENSOCUT00000016142	ENSRNOT00000048488	ENSSTOT00000009226	ENSTBET00000011056	ENSTSYT00000013017
ENST00000262487	ENSOGAT00000001279	ENSPTRT00000024644	ENSBTAT00000022843	ENSNLET00000018555	ENSGGOT00000002717	ENSCPOT00000013574	ENSDORT00000010162	ENSMMUT00000022416	ENSCJAT00000008806	ENSMUST00000099307	ENSMICT00000013543	ENSPPYT00000012449	ENSOPRT00000003997	ENSOCUT00000001309	ENSRNOT00000064211	ENSSTOT00000000673	ENSTBET00000000289	ENSTSYT00000009897
ENST00000376542	ENSOGAT00000008506	ENSPTRT00000024781	ENSBTAT00000001863	ENSNLET00000019956	ENSGGOT00000014355	ENSCPOT00000004123	ENSDORT00000007179	ENSMMUT00000012865	ENSCJAT00000022688	ENSMUST00000056149	ENSMICT00000005782	ENSPPYT00000012525	ENSOPRT00000008410	ENSOCUT00000002799	ENSRNOT00000010641	ENSSTOT00000014397	ENSTBET00000011968	ENSTSYT00000010155
ENST00000402881	ENSOGAT00000000037	ENSPTRT00000026905	ENSBTAT00000023204	ENSNLET00000017720	ENSGGOT00000010150	ENSCPOT00000008768	ENSDORT00000004949	ENSMMUT00000006372	ENSCJAT00000004954	ENSMUST00000166030	ENSMICT00000011441	ENSPPYT00000013750	ENSOPRT00000010560	ENSOCUT00000012850	ENSRNOT00000023843	ENSSTOT00000008208	ENSTBET00000005812	ENSTSYT00000000583
ENST00000440869	ENSOGAT00000012136	ENSPTRT00000056773	ENSBTAT00000037722	ENSNLET00000019284	ENSGGOT00000031615	ENSCPOT00000013850	ENSDORT00000010265	ENSMMUT00000007526	ENSCJAT00000037816	ENSMUST00000105547	ENSMICT00000009816	ENSPPYT00000019860	ENSOPRT00000001133	ENSOCUT00000009614	ENSRNOT00000065809	ENSSTOT00000012864	ENSTBET00000015561	ENSTSYT00000009297
ENST00000409792	ENSOGAT00000016833	ENSPTRT00000027789	ENSBTAT00000003838	ENSNLET00000007974	ENSGGOT00000024070	ENSCPOT00000008290	ENSDORT00000014190	ENSMMUT00000029696	ENSCJAT00000061085	ENSMUST00000035068	ENSMICT00000017655	ENSPPYT00000016194	ENSOPRT00000005224	ENSOCUT00000002840	ENSRNOT00000028409	ENSSTOT00000003163	ENSTBET00000015770	ENSTSYT00000011571
ENST00000377960	ENSOGAT00000007852	ENSPTRT00000074451	ENSBTAT00000000086	ENSNLET00000004990	ENSGGOT00000025403	ENSCPOT00000013459	ENSDORT00000012831	ENSMMUT00000014262	ENSCJAT00000019819	ENSMUST00000030202	ENSMICT00000017673	ENSPPYT00000022235	ENSOPRT00000009299	ENSOCUT00000000006	ENSRNOT00000019916	ENSSTOT00000005006	ENSTBET00000009272	ENSTSYT00000005135
ENST00000370500	ENSOGAT00000005755	ENSPTRT00000001764	ENSBTAT00000044137	ENSNLET00000013060	ENSGGOT00000026761	ENSCPOT00000014170	ENSDORT00000008028	ENSMMUT00000003964	ENSCJAT00000014663	ENSMUST00000029938	ENSMICT00000014701	ENSPPYT00000001410	ENSOPRT00000009923	ENSOCUT00000000139	ENSRNOT00000015032	ENSSTOT00000015437	ENSTBET00000001388	ENSTSYT00000010347
ENST00000256538	ENSOGAT00000028388	ENSPTRT00000012716	ENSBTAT00000055382	ENSNLET00000014300	ENSGGOT00000015574	ENSCPOT00000015684	ENSDORT00000012219	ENSMMUT00000005908	ENSCJAT00000041303	ENSMUST00000055144	ENSMICT00000016999	ENSPPYT00000007465	ENSOPRT00000014011	ENSOCUT00000003948	ENSRNOT00000056522	ENSSTOT00000010525	ENSTBET00000004283	ENSTSYT00000000775
ENST00000305904	ENSOGAT00000000908	ENSPTRT00000015451	ENSBTAT00000023603	ENSNLET00000013301	ENSGGOT00000008724	ENSCPOT00000024112	ENSDORT00000000889	ENSMMUT00000002695	ENSCJAT00000032495	ENSMUST00000078170	ENSMICT00000000628	ENSPPYT00000008902	ENSOPRT00000009525	ENSOCUT00000008571	ENSRNOT00000016713	ENSSTOT00000012999	ENSTBET00000000007	ENSTSYT00000007772
ENST00000265395	ENSOGAT00000000425	ENSPTRT00000035166	ENSBTAT00000001374	ENSNLET00000019156	ENSGGOT00000013315	ENSCPOT00000025371	ENSDORT00000002792	ENSMMUT00000042179	ENSCJAT00000016031	ENSMUST00000031788	ENSMICT00000000511	ENSPPYT00000020615	ENSOPRT00000005558	ENSOCUT00000010346	ENSRNOT00000011069	ENSSTOT00000004111	ENSTBET00000003808	ENSTSYT00000014186
ENST00000453539	ENSOGAT00000013729	ENSPTRT00000022671	ENSBTAT00000002533	ENSNLET00000000004	ENSGGOT00000007880	ENSCPOT00000004456	ENSDORT00000016625	ENSMMUT00000027289	ENSCJAT00000037108	ENSMUST00000110368	ENSMICT00000011273	ENSPPYT00000013988	ENSOPRT00000001816	ENSOCUT00000003558	ENSRNOT00000020367	ENSSTOT00000007534	ENSTBET00000012834	ENSTSYT00000004899
ENST00000435533	ENSOGAT00000014778	ENSPTRT00000003382	ENSBTAT00000040284	ENSNLET00000023105	ENSGGOT00000009204	ENSCPOT00000023555	ENSDORT00000004280	ENSMMUT00000025086	ENSCJAT00000063214	ENSMUST00000027725	ENSMICT00000007930	ENSPPYT00000000397	ENSOPRT00000010201	ENSOCUT00000009219	ENSRNOT00000005832	ENSSTOT00000015455	ENSTBET00000014858	ENSTSYT00000008934
ENST00000264167	ENSOGAT00000013186	ENSPTRT00000023485	ENSBTAT00000061434	ENSNLET00000007687	ENSGGOT00000026006	ENSCPOT00000000684	ENSDORT00000006482	ENSMMUT00000011881	ENSCJAT00000042314	ENSMUST00000047232	ENSMICT00000003755	ENSPPYT00000015075	ENSOPRT00000009170	ENSOCUT00000011526	ENSRNOT00000002111	ENSSTOT00000012628	ENSTBET00000001944	ENSTSYT00000004480
ENST00000444448	ENSOGAT00000007900	ENSPTRT00000023352	ENSBTAT00000013682	ENSNLET00000005919	ENSGGOT00000029967	ENSCPOT00000005980	ENSDORT00000005395	ENSMMUT00000028065	ENSCJAT00000054925	ENSMUST00000041865	ENSMICT00000017322	ENSPPYT00000014990	ENSOPRT00000013250	ENSOCUT00000005730	ENSRNOT00000067161	ENSSTOT00000007421	ENSTBET00000011370	ENSTSYT00000013127
ENST00000399050	ENSOGAT00000000780	ENSPTRT00000043390	ENSBTAT00000043778	ENSNLET00000016585	ENSGGOT00000015287	ENSCPOT00000005373	ENSDORT00000014693	ENSMMUT00000044231	ENSCJAT00000016697	ENSMUST00000111694	ENSMICT00000004915	ENSPPYT00000003885	ENSOPRT00000002688	ENSOCUT00000014218	ENSRNOT00000066861	ENSSTOT00000010841	ENSTBET00000003566	ENSTSYT00000005805
ENST00000306726	ENSOGAT00000000156	ENSPTRT00000013475	ENSBTAT00000022588	ENSNLET00000016994	ENSGGOT00000015801	ENSCPOT00000012124	ENSDORT00000010772	ENSMMUT00000005355	ENSCJAT00000030452	ENSMUST00000034832	ENSMICT00000016179	ENSPPYT00000007864	ENSOPRT00000008380	ENSOCUT00000012603	ENSRNOT00000023831	ENSSTOT00000005883	ENSTBET00000010978	ENSTSYT00000014162
ENST00000238831	ENSOGAT00000005960	ENSPTRT00000021992	ENSBTAT00000010857	ENSNLET00000020051	ENSGGOT00000012216	ENSCPOT00000014056	ENSDORT00000004768	ENSMMUT00000022631	ENSCJAT00000006317	ENSMUST00000024873	ENSMICT00000009519	ENSPPYT00000014533	ENSOPRT00000015337	ENSOCUT00000007589	ENSRNOT00000007607	ENSSTOT00000012129	ENSTBET00000017577	ENSTSYT00000005379
ENST00000305798	ENSOGAT00000006273	ENSPTRT00000030330	ENSBTAT00000016375	ENSNLET00000017857	ENSGGOT00000033901	ENSCPOT00000004863	ENSDORT00000005085	ENSMMUT00000016394	ENSCJAT00000013992	ENSMUST00000029800	ENSMICT00000005800	ENSPPYT00000017364	ENSOPRT00000005006	ENSOCUT00000016549	ENSRNOT00000021381	ENSSTOT00000012042	ENSTBET00000010275	ENSTSYT00000006279
ENST00000259782	ENSOGAT00000013016	ENSPTRT00000057114	ENSBTAT00000016790	ENSNLET00000004275	ENSGGOT00000002381	ENSCPOT00000002945	ENSDORT00000011595	ENSMMUT00000017935	ENSCJAT00000021300	ENSMUST00000034911	ENSMICT00000014442	ENSPPYT00000019454	ENSOPRT00000007698	ENSOCUT00000015801	ENSRNOT00000041567	ENSSTOT00000011231	ENSTBET00000016122	ENSTSYT00000008940
ENST00000371073	ENSOGAT00000011156	ENSPTRT00000001562	ENSBTAT00000021878	ENSNLET00000019305	ENSGGOT00000007732	ENSCPOT00000011813	ENSDORT00000011104	ENSMMUT00000010601	ENSCJAT00000032490	ENSMUST00000030257	ENSMICT00000013710	ENSPPYT00000001530	ENSOPRT00000014433	ENSOCUT00000015538	ENSRNOT00000014354	ENSSTOT00000009830	ENSTBET00000008071	ENSTSYT00000014675
ENST00000367874	ENSOGAT00000007625	ENSPTRT00000002962	ENSBTAT00000013804	ENSNLET00000018847	ENSGGOT00000008206	ENSCPOT00000022977	ENSDORT00000006823	ENSMMUT00000025003	ENSCJAT00000031152	ENSMUST00000027846	ENSMICT00000011765	ENSPPYT00000000676	ENSOPRT00000010386	ENSOCUT00000010561	ENSRNOT00000005090	ENSSTOT00000006198	ENSTBET00000007270	ENSTSYT00000011078
ENST00000369103	ENSOGAT00000034179	ENSPTRT00000005704	ENSBTAT00000003425	ENSNLET00000021325	ENSGGOT00000008449	ENSCPOT00000004371	ENSDORT00000003520	ENSMMUT00000030416	ENSCJAT00000038594	ENSMUST00000033133	ENSMICT00000005517	ENSPPYT00000003261	ENSOPRT00000017206	ENSOCUT00000014613	ENSRNOT00000027375	ENSSTOT00000004921	ENSTBET00000006998	ENSTSYT00000008950
ENST00000395895	ENSOGAT00000013989	ENSPTRT00000004475	ENSBTAT00000021381	ENSNLET00000023164	ENSGGOT00000004020	ENSCPOT00000015593	ENSDORT00000001161	ENSMMUT00000031662	ENSCJAT00000027337	ENSMUST00000150235	ENSMICT00000000834	ENSPPYT00000002645	ENSOPRT00000008801	ENSOCUT00000001657	ENSRNOT00000068545	ENSSTOT00000005395	ENSTBET00000004835	ENSTSYT00000012092
ENST00000264414	ENSOGAT00000012103	ENSPTRT00000024062	ENSBTAT00000029018	ENSNLET00000012473	ENSGGOT00000007485	ENSCPOT00000005719	ENSDORT00000005559	ENSMMUT00000044483	ENSCJAT00000059716	ENSMUST00000163119	ENSMICT00000013988	ENSPPYT00000015383	ENSOPRT00000012352	ENSOCUT00000010660	ENSRNOT00000021528	ENSSTOT00000015379	ENSTBET00000010646	ENSTSYT00000007393
ENST00000514860	ENSOGAT00000009280	ENSPTRT00000030803	ENSBTAT00000055600	ENSNLET00000008284	ENSGGOT00000034516	ENSCPOT00000005883	ENSDORT00000016605	ENSMMUT00000024384	ENSCJAT00000018002	ENSMUST00000034017	ENSMICT00000000793	ENSPPYT00000017644	ENSOPRT00000011286	ENSOCUT00000003762	ENSRNOT00000047223	ENSSTOT00000012264	ENSTBET00000005306	ENSTSYT00000009269
ENST00000397033	ENSOGAT00000016135	ENSPTRT00000023547	ENSBTAT00000012194	ENSNLET00000008034	ENSGGOT00000031256	ENSCPOT00000009565	ENSDORT00000002416	ENSMMUT00000000438	ENSCJAT00000022442	ENSMUST00000028395	ENSMICT00000012624	ENSPPYT00000015097	ENSOPRT00000008563	ENSOCUT00000003013	ENSRNOT00000066126	ENSSTOT00000009860	ENSTBET00000007111	ENSTSYT00000008313
ENST00000393158	ENSOGAT00000009989	ENSPTRT00000007883	ENSBTAT00000049292	ENSNLET00000009190	ENSGGOT00000002115	ENSCPOT00000005653	ENSDORT00000014006	ENSMMUT00000007533	ENSCJAT00000018988	ENSMUST00000168039	ENSMICT00000002458	ENSPPYT00000004545	ENSOPRT00000009535	ENSOCUT00000015311	ENSRNOT00000049325	ENSSTOT00000004225	ENSTBET00000014904	ENSTSYT00000012239
ENST00000369406	ENSOGAT00000000816	ENSPTRT00000002189	ENSBTAT00000005088	ENSNLET00000006939	ENSGGOT00000010338	ENSCPOT00000006404	ENSDORT00000008532	ENSMMUT00000032496	ENSCJAT00000039095	ENSMUST00000120541	ENSMICT00000004469	ENSPPYT00000001156	ENSOPRT00000006210	ENSOCUT00000005765	ENSRNOT00000026121	ENSSTOT00000001915	ENSTBET00000005044	ENSTSYT00000008505
ENST00000373030	ENSOGAT00000007704	ENSPTRT00000025136	ENSBTAT00000026122	ENSNLET00000008368	ENSGGOT00000030791	ENSCPOT00000021655	ENSDORT00000011592	ENSMMUT00000000578	ENSCJAT00000034833	ENSMUST00000018002	ENSMICT00000015197	ENSPPYT00000012790	ENSOPRT00000002072	ENSOCUT00000006677	ENSRNOT00000064649	ENSSTOT00000010140	ENSTBET00000015628	ENSTSYT00000002368
ENST00000520429	ENSOGAT00000001117	ENSPTRT00000034085	ENSBTAT00000037858	ENSNLET00000017642	ENSGGOT00000007458	ENSCPOT00000011907	ENSDORT00000008392	ENSMMUT00000005765	ENSCJAT00000002650	ENSMUST00000102997	ENSMICT00000001094	ENSPPYT00000019624	ENSOPRT00000012980	ENSOCUT00000003309	ENSRNOT00000043469	ENSSTOT00000016569	ENSTBET00000017527	ENSTSYT00000014408
ENST00000411696	ENSOGAT00000031751	ENSPTRT00000023955	ENSBTAT00000044020	ENSNLET00000011213	ENSGGOT00000033629	ENSCPOT00000002573	ENSDORT00000004707	ENSMMUT00000009431	ENSCJAT00000021709	ENSMUST00000066986	ENSMICT00000005164	ENSPPYT00000015323	ENSOPRT00000006006	ENSOCUT00000001997	ENSRNOT00000067080	ENSSTOT00000006441	ENSTBET00000011426	ENSTSYT00000007130
ENST00000261712	ENSOGAT00000015461	ENSPTRT00000016575	ENSBTAT00000025152	ENSNLET00000007647	ENSGGOT00000001061	ENSCPOT00000009886	ENSDORT00000011440	ENSMMUT00000026658	ENSCJAT00000028214	ENSMUST00000173938	ENSMICT00000011850	ENSPPYT00000009546	ENSOPRT00000014784	ENSOCUT00000008496	ENSRNOT00000008083	ENSSTOT00000009691	ENSTBET00000001969	ENSTSYT00000012072
ENST00000396148	ENSOGAT00000003628	ENSPTRT00000001374	ENSBTAT00000014685	ENSNLET00000020711	ENSGGOT00000017079	ENSCPOT00000000832	ENSDORT00000015488	ENSMMUT00000026777	ENSCJAT00000042398	ENSMUST00000063531	ENSMICT00000003303	ENSPPYT00000001639	ENSOPRT00000012506	ENSOCUT00000008382	ENSRNOT00000012088	ENSSTOT00000013795	ENSTBET00000012418	ENSTSYT00000001318
ENST00000255152	ENSOGAT00000004996	ENSPTRT00000025260	ENSBTAT00000001267	ENSNLET00000008655	ENSGGOT00000003079	ENSCPOT00000012935	ENSDORT00000001580	ENSMMUT00000017544	ENSCJAT00000033712	ENSMUST00000052107	ENSMICT00000011239	ENSPPYT00000012859	ENSOPRT00000014333	ENSOCUT00000012187	ENSRNOT00000021052	ENSSTOT00000006540	ENSTBET00000003523	ENSTSYT00000009076
ENST00000440851	ENSOGAT00000015568	ENSPTRT00000015199	ENSBTAT00000008396	ENSNLET00000006272	ENSGGOT00000001469	ENSCPOT00000001021	ENSDORT00000010700	ENSMMUT00000010929	ENSCJAT00000031171	ENSMUST00000040776	ENSMICT00000012229	ENSPPYT00000008781	ENSOPRT00000013708	ENSOCUT00000006314	ENSRNOT00000036702	ENSSTOT00000002866	ENSTBET00000010764	ENSTSYT00000005845
ENST00000451791	ENSOGAT00000000540	ENSPTRT00000037552	ENSBTAT00000006478	ENSNLET00000001627	ENSGGOT00000016317	ENSCPOT00000012900	ENSDORT00000011718	ENSMMUT00000009481	ENSCJAT00000032284	ENSMUST00000070375	ENSMICT00000010647	ENSPPYT00000021698	ENSOPRT00000016410	ENSOCUT00000007054	ENSRNOT00000011892	ENSSTOT00000011135	ENSTBET00000011402	ENSTSYT00000000718
ENST00000377602	ENSOGAT00000013543	ENSPTRT00000004279	ENSBTAT00000039438	ENSNLET00000016053	ENSGGOT00000008891	ENSCPOT00000014988	ENSDORT00000005803	ENSMMUT00000021684	ENSCJAT00000016873	ENSMUST00000170090	ENSMICT00000002147	ENSPPYT00000002539	ENSOPRT00000002057	ENSOCUT00000007238	ENSRNOT00000024475	ENSSTOT00000013709	ENSTBET00000011216	ENSTSYT00000013850
ENST00000373367	ENSOGAT00000016924	ENSPTRT00000041030	ENSBTAT00000010589	ENSNLET00000001381	ENSGGOT00000023006	ENSCPOT00000006811	ENSDORT00000009329	ENSMMUT00000029838	ENSCJAT00000057691	ENSMUST00000042070	ENSMICT00000001081	ENSPPYT00000023895	ENSOPRT00000006720	ENSOCUT00000011748	ENSRNOT00000003714	ENSSTOT00000001590	ENSTBET00000016818	ENSTSYT00000012578
ENST00000316549	ENSOGAT00000004345	ENSPTRT00000028901	ENSBTAT00000044136	ENSNLET00000006396	ENSGGOT00000041020	ENSCPOT00000000668	ENSDORT00000011135	ENSMMUT00000013534	ENSCJAT00000003044	ENSMUST00000033463	ENSMICT00000013053	ENSPPYT00000016488	ENSOPRT00000011396	ENSOCUT00000009911	ENSRNOT00000011358	ENSSTOT00000016007	ENSTBET00000015239	ENSTSYT00000010733
ENST00000409204	ENSOGAT00000004916	ENSPTRT00000010868	ENSBTAT00000015745	ENSNLET00000020316	ENSGGOT00000005558	ENSCPOT00000010761	ENSDORT00000001881	ENSMMUT00000008095	ENSCJAT00000015263	ENSMUST00000061628	ENSMICT00000000412	ENSPPYT00000006397	ENSOPRT00000003423	ENSOCUT00000016875	ENSRNOT00000017765	ENSSTOT00000009137	ENSTBET00000006305	ENSTSYT00000006159
ENST00000380490	ENSOGAT00000015499	ENSPTRT00000010590	ENSBTAT00000017570	ENSNLET00000000984	ENSGGOT00000013909	ENSCPOT00000025234	ENSDORT00000006041	ENSMMUT00000007833	ENSCJAT00000042504	ENSMUST00000071130	ENSMICT00000001951	ENSPPYT00000006219	ENSOPRT00000001904	ENSOCUT00000008200	ENSRNOT00000001207	ENSSTOT00000010916	ENSTBET00000013632	ENSTSYT00000005492
ENST00000314400	ENSOGAT00000003003	ENSPTRT00000028454	ENSBTAT00000007523	ENSNLET00000005663	ENSGGOT00000009317	ENSCPOT00000004514	ENSDORT00000008911	ENSMMUT00000020871	ENSCJAT00000039899	ENSMUST00000048788	ENSMICT00000017773	ENSPPYT00000015747	ENSOPRT00000008901	ENSOCUT00000010818	ENSRNOT00000018473	ENSSTOT00000002145	ENSTBET00000006220	ENSTSYT00000008329
ENST00000304877	ENSOGAT00000015422	ENSPTRT00000034303	ENSBTAT00000004332	ENSNLET00000017793	ENSGGOT00000004874	ENSCPOT00000013277	ENSDORT00000003050	ENSMMUT00000004442	ENSCJAT00000007290	ENSMUST00000169276	ENSMICT00000014042	ENSPPYT00000019770	ENSOPRT00000000333	ENSOCUT00000011858	ENSRNOT00000030327	ENSSTOT00000009709	ENSTBET00000011748	ENSTSYT00000011157
ENST00000380605	ENSOGAT00000004696	ENSPTRT00000024471	ENSBTAT00000053534	ENSNLET00000011634	ENSGGOT00000007770	ENSCPOT00000012051	ENSDORT00000003236	ENSMMUT00000030023	ENSCJAT00000041391	ENSMUST00000028897	ENSMICT00000016565	ENSPPYT00000012579	ENSOPRT00000009629	ENSOCUT00000006187	ENSRNOT00000028818	ENSSTOT00000016356	ENSTBET00000012188	ENSTSYT00000014353
ENST00000502959	ENSOGAT00000004326	ENSPTRT00000030753	ENSBTAT00000005009	ENSNLET00000007964	ENSGGOT00000007196	ENSCPOT00000015078	ENSDORT00000004396	ENSMMUT00000041593	ENSCJAT00000003260	ENSMUST00000029635	ENSMICT00000015315	ENSPPYT00000017609	ENSOPRT00000013758	ENSOCUT00000001814	ENSRNOT00000064930	ENSSTOT00000007478	ENSTBET00000013402	ENSTSYT00000014670
ENST00000314616	ENSOGAT00000012100	ENSPTRT00000016468	ENSBTAT00000011900	ENSNLET00000003503	ENSGGOT00000016764	ENSCPOT00000001461	ENSDORT00000009241	ENSMMUT00000006649	ENSCJAT00000025387	ENSMUST00000002121	ENSMICT00000016904	ENSPPYT00000009483	ENSOPRT00000012580	ENSOCUT00000000849	ENSRNOT00000016759	ENSSTOT00000000585	ENSTBET00000001942	ENSTSYT00000004556
ENST00000370454	ENSOGAT00000005171	ENSPTRT00000001779	ENSBTAT00000001193	ENSNLET00000013116	ENSGGOT00000024004	ENSCPOT00000008110	ENSDORT00000002339	ENSMMUT00000011191	ENSCJAT00000003682	ENSMUST00000067924	ENSMICT00000006685	ENSPPYT00000001399	ENSOPRT00000014394	ENSOCUT00000010779	ENSRNOT00000002896	ENSSTOT00000010185	ENSTBET00000015607	ENSTSYT00000010962
ENST00000323570	ENSOGAT00000006806	ENSPTRT00000030598	ENSBTAT00000018612	ENSNLET00000006569	ENSGGOT00000033891	ENSCPOT00000001990	ENSDORT00000001945	ENSMMUT00000021367	ENSCJAT00000005813	ENSMUST00000053902	ENSMICT00000003976	ENSPPYT00000017527	ENSOPRT00000004612	ENSOCUT00000006809	ENSRNOT00000031614	ENSSTOT00000003092	ENSTBET00000000015	ENSTSYT00000012301
ENST00000300584	ENSOGAT00000014096	ENSPTRT00000013526	ENSBTAT00000015943	ENSNLET00000010727	ENSGGOT00000009072	ENSCPOT00000001578	ENSDORT00000011071	ENSMMUT00000013836	ENSCJAT00000019623	ENSMUST00000041767	ENSMICT00000009143	ENSPPYT00000007899	ENSOPRT00000005574	ENSOCUT00000006503	ENSRNOT00000030645	ENSSTOT00000003703	ENSTBET00000000099	ENSTSYT00000000295
ENST00000522781	ENSOGAT00000027325	ENSPTRT00000005807	ENSBTAT00000027987	ENSNLET00000000703	ENSGGOT00000006521	ENSCPOT00000015652	ENSDORT00000003447	ENSMMUT00000026079	ENSCJAT00000027654	ENSMUST00000171394	ENSMICT00000006483	ENSPPYT00000003330	ENSOPRT00000005762	ENSOCUT00000001461	ENSRNOT00000047069	ENSSTOT00000008114	ENSTBET00000014700	ENSTSYT00000003105
ENST00000299339	ENSOGAT00000004466	ENSPTRT00000010960	ENSBTAT00000004641	ENSNLET00000021173	ENSGGOT00000025874	ENSCPOT00000003861	ENSDORT00000011637	ENSMMUT00000013577	ENSCJAT00000006432	ENSMUST00000100314	ENSMICT00000011948	ENSPPYT00000006454	ENSOPRT00000017357	ENSOCUT00000009436	ENSRNOT00000013520	ENSSTOT00000010139	ENSTBET00000007713	ENSTSYT00000011830
ENST00000372383	ENSOGAT00000032911	ENSPTRT00000041273	ENSBTAT00000066170	ENSNLET00000017789	ENSGGOT00000005218	ENSCPOT00000006305	ENSDORT00000004688	ENSMMUT00000011473	ENSCJAT00000015395	ENSMUST00000112996	ENSMICT00000001930	ENSPPYT00000024044	ENSOPRT00000013734	ENSOCUT00000012392	ENSRNOT00000019514	ENSSTOT00000002763	ENSTBET00000012498	ENSTSYT00000011307
ENST00000534548	ENSOGAT00000015368	ENSPTRT00000008322	ENSBTAT00000039478	ENSNLET00000015905	ENSGGOT00000006818	ENSCPOT00000006606	ENSDORT00000012781	ENSMMUT00000009911	ENSCJAT00000038414	ENSMUST00000073127	ENSMICT00000005029	ENSPPYT00000004839	ENSOPRT00000009965	ENSOCUT00000013680	ENSRNOT00000012078	ENSSTOT00000005557	ENSTBET00000017448	ENSTSYT00000005696
ENST00000303714	ENSOGAT00000012186	ENSPTRT00000022345	ENSBTAT00000061292	ENSNLET00000012887	ENSGGOT00000005657	ENSCPOT00000010053	ENSDORT00000015979	ENSMMUT00000019990	ENSCJAT00000037514	ENSMUST00000042025	ENSMICT00000003759	ENSPPYT00000014322	ENSOPRT00000003362	ENSOCUT00000010237	ENSRNOT00000011675	ENSSTOT00000010372	ENSTBET00000006259	ENSTSYT00000000931
ENST00000414689	ENSOGAT00000011924	ENSPTRT00000014719	ENSBTAT00000029038	ENSNLET00000009698	ENSGGOT00000012015	ENSCPOT00000022013	ENSDORT00000003062	ENSMMUT00000028516	ENSCJAT00000013868	ENSMUST00000120007	ENSMICT00000009083	ENSPPYT00000008534	ENSOPRT00000009614	ENSOCUT00000006315	ENSRNOT00000027094	ENSSTOT00000002541	ENSTBET00000008842	ENSTSYT00000000591
ENST00000442201	ENSOGAT00000006901	ENSPTRT00000029187	ENSBTAT00000002557	ENSNLET00000007522	ENSGGOT00000041102	ENSCPOT00000015463	ENSDORT00000002728	ENSMMUT00000027616	ENSCJAT00000025035	ENSMUST00000029222	ENSMICT00000009505	ENSPPYT00000016664	ENSOPRT00000002753	ENSOCUT00000000815	ENSRNOT00000015847	ENSSTOT00000003861	ENSTBET00000009396	ENSTSYT00000012671
ENST00000369109	ENSOGAT00000004846	ENSPTRT00000050506	ENSBTAT00000046131	ENSNLET00000012548	ENSGGOT00000017175	ENSCPOT00000006574	ENSDORT00000011693	ENSMMUT00000030104	ENSCJAT00000057771	ENSMUST00000056710	ENSMICT00000015299	ENSPPYT00000001093	ENSOPRT00000010089	ENSOCUT00000009036	ENSRNOT00000032722	ENSSTOT00000006037	ENSTBET00000003471	ENSTSYT00000012166
ENST00000286688	ENSOGAT00000007225	ENSPTRT00000037815	ENSBTAT00000060954	ENSNLET00000004525	ENSGGOT00000007857	ENSCPOT00000012532	ENSDORT00000003247	ENSMMUT00000023170	ENSCJAT00000040353	ENSMUST00000080517	ENSMICT00000004576	ENSPPYT00000021886	ENSOPRT00000000773	ENSOCUT00000001061	ENSRNOT00000037547	ENSSTOT00000007760	ENSTBET00000002806	ENSTSYT00000002884
ENST00000016171	ENSOGAT00000001582	ENSPTRT00000046834	ENSBTAT00000064176	ENSNLET00000018680	ENSGGOT00000010617	ENSCPOT00000009871	ENSDORT00000005427	ENSMMUT00000033444	ENSCJAT00000033251	ENSMUST00000045562	ENSMICT00000010507	ENSPPYT00000003071	ENSOPRT00000003018	ENSOCUT00000003142	ENSRNOT00000023215	ENSSTOT00000010458	ENSTBET00000004340	ENSTSYT00000007658
ENST00000376344	ENSOGAT00000031863	ENSPTRT00000038955	ENSBTAT00000008492	ENSNLET00000004426	ENSGGOT00000011336	ENSCPOT00000002016	ENSDORT00000016023	ENSMMUT00000026527	ENSCJAT00000007165	ENSMUST00000022032	ENSMICT00000015768	ENSPPYT00000022542	ENSOPRT00000016270	ENSOCUT00000008834	ENSRNOT00000061249	ENSSTOT00000013497	ENSTBET00000001156	ENSTSYT00000014478
ENST00000263733	ENSOGAT00000012753	ENSPTRT00000003122	ENSBTAT00000018604	ENSNLET00000019848	ENSGGOT00000002498	ENSCPOT00000013890	ENSDORT00000009524	ENSMMUT00000008763	ENSCJAT00000016294	ENSMUST00000122424	ENSMICT00000002494	ENSPPYT00000000557	ENSOPRT00000009708	ENSOCUT00000009243	ENSRNOT00000006018	ENSSTOT00000005086	ENSTBET00000017150	ENSTSYT00000005469
ENST00000282226	ENSOGAT00000005009	ENSPTRT00000031581	ENSBTAT00000042733	ENSNLET00000016552	ENSGGOT00000034019	ENSCPOT00000011533	ENSDORT00000015177	ENSMMUT00000027341	ENSCJAT00000038388	ENSMUST00000040106	ENSMICT00000002408	ENSPPYT00000018143	ENSOPRT00000015280	ENSOCUT00000016322	ENSRNOT00000031909	ENSSTOT00000009446	ENSTBET00000004009	ENSTSYT00000003589
ENST00000311762	ENSOGAT00000006516	ENSPTRT00000037942	ENSBTAT00000026634	ENSNLET00000005374	ENSGGOT00000016712	ENSCPOT00000011445	ENSDORT00000004995	ENSMMUT00000012202	ENSCJAT00000012499	ENSMUST00000038856	ENSMICT00000000931	ENSPPYT00000021961	ENSOPRT00000016088	ENSOCUT00000003834	ENSRNOT00000006783	ENSSTOT00000015262	ENSTBET00000006283	ENSTSYT00000005808
ENST00000170447	ENSOGAT00000003258	ENSPTRT00000027385	ENSBTAT00000005927	ENSNLET00000006024	ENSGGOT00000000431	ENSCPOT00000001221	ENSDORT00000015620	ENSMMUT00000013310	ENSCJAT00000032533	ENSMUST00000000449	ENSMICT00000012159	ENSPPYT00000015574	ENSOPRT00000003489	ENSOCUT00000014170	ENSRNOT00000044916	ENSSTOT00000010519	ENSTBET00000012109	ENSTSYT00000007419
ENST00000234256	ENSOGAT00000005686	ENSPTRT00000022303	ENSBTAT00000010206	ENSNLET00000012694	ENSGGOT00000011255	ENSCPOT00000008832	ENSDORT00000002394	ENSMMUT00000014365	ENSCJAT00000055063	ENSMUST00000109594	ENSMICT00000012575	ENSPPYT00000014344	ENSOPRT00000006580	ENSOCUT00000010820	ENSRNOT00000066154	ENSSTOT00000011470	ENSTBET00000004914	ENSTSYT00000001915
ENST00000373381	ENSOGAT00000012761	ENSPTRT00000000989	ENSBTAT00000007609	ENSNLET00000004413	ENSGGOT00000017235	ENSCPOT00000007601	ENSDORT00000007442	ENSMMUT00000047405	ENSCJAT00000002534	ENSMUST00000074081	ENSMICT00000010422	ENSPPYT00000001870	ENSOPRT00000006284	ENSOCUT00000007203	ENSRNOT00000036029	ENSSTOT00000009154	ENSTBET00000004883	ENSTSYT00000003469
ENST00000393153	ENSOGAT00000009450	ENSPTRT00000012271	ENSBTAT00000065331	ENSNLET00000021963	ENSGGOT00000014516	ENSCPOT00000013187	ENSDORT00000003785	ENSMMUT00000017380	ENSCJAT00000035984	ENSMUST00000101099	ENSMICT00000008334	ENSPPYT00000007198	ENSOPRT00000000516	ENSOCUT00000010978	ENSRNOT00000066306	ENSSTOT00000011204	ENSTBET00000015587	ENSTSYT00000007225
ENST00000315870	ENSOGAT00000004214	ENSPTRT00000036348	ENSBTAT00000021725	ENSNLET00000015112	ENSGGOT00000004372	ENSCPOT00000021107	ENSDORT00000014497	ENSMMUT00000003458	ENSCJAT00000033368	ENSMUST00000031680	ENSMICT00000013934	ENSPPYT00000020917	ENSOPRT00000016916	ENSOCUT00000001882	ENSRNOT00000007476	ENSSTOT00000004038	ENSTBET00000004309	ENSTSYT00000000136
ENST00000344347	ENSOGAT00000003975	ENSPTRT00000026546	ENSBTAT00000007835	ENSNLET00000003212	ENSGGOT00000007185	ENSCPOT00000007870	ENSDORT00000004876	ENSMMUT00000010079	ENSCJAT00000039444	ENSMUST00000109866	ENSMICT00000004237	ENSPPYT00000013556	ENSOPRT00000013980	ENSOCUT00000006926	ENSRNOT00000014044	ENSSTOT00000004158	ENSTBET00000000321	ENSTSYT00000000513
ENST00000369983	ENSOGAT00000016377	ENSPTRT00000005500	ENSBTAT00000061619	ENSNLET00000019234	ENSGGOT00000006992	ENSCPOT00000004563	ENSDORT00000013406	ENSMMUT00000004232	ENSCJAT00000034963	ENSMUST00000026254	ENSMICT00000011845	ENSPPYT00000003126	ENSOPRT00000006561	ENSOCUT00000005726	ENSRNOT00000036156	ENSSTOT00000002181	ENSTBET00000006775	ENSTSYT00000006330
ENST00000296861	ENSOGAT00000027208	ENSPTRT00000033732	ENSBTAT00000026719	ENSNLET00000005466	ENSGGOT00000013525	ENSCPOT00000010381	ENSDORT00000008533	ENSMMUT00000016680	ENSCJAT00000023143	ENSMUST00000024708	ENSMICT00000017760	ENSPPYT00000019397	ENSOPRT00000016477	ENSOCUT00000016534	ENSRNOT00000015918	ENSSTOT00000004268	ENSTBET00000007307	ENSTSYT00000009091
ENST00000229329	ENSOGAT00000001975	ENSPTRT00000008803	ENSBTAT00000013373	ENSNLET00000006332	ENSGGOT00000015117	ENSCPOT00000000995	ENSDORT00000015219	ENSMMUT00000029276	ENSCJAT00000009352	ENSMUST00000032419	ENSMICT00000004161	ENSPPYT00000005174	ENSOPRT00000015541	ENSOCUT00000013126	ENSRNOT00000018734	ENSSTOT00000010302	ENSTBET00000010914	ENSTSYT00000006196
ENST00000493277	ENSOGAT00000009918	ENSPTRT00000036019	ENSBTAT00000025885	ENSNLET00000014250	ENSGGOT00000002500	ENSCPOT00000003874	ENSDORT00000006846	ENSMMUT00000028341	ENSCJAT00000030737	ENSMUST00000167316	ENSMICT00000006009	ENSPPYT00000020251	ENSOPRT00000013441	ENSOCUT00000007930	ENSRNOT00000001296	ENSSTOT00000008194	ENSTBET00000014142	ENSTSYT00000002635
ENST00000537543	ENSOGAT00000001193	ENSPTRT00000045412	ENSBTAT00000006855	ENSNLET00000016714	ENSGGOT00000028506	ENSCPOT00000003445	ENSDORT00000012762	ENSMMUT00000039976	ENSCJAT00000052367	ENSMUST00000048010	ENSMICT00000008107	ENSPPYT00000019722	ENSOPRT00000005680	ENSOCUT00000007341	ENSRNOT00000001093	ENSSTOT00000013912	ENSTBET00000009307	ENSTSYT00000009566
ENST00000321582	ENSOGAT00000015092	ENSPTRT00000039430	ENSBTAT00000017043	ENSNLET00000021886	ENSGGOT00000000561	ENSCPOT00000010275	ENSDORT00000007585	ENSMMUT00000020309	ENSCJAT00000039587	ENSMUST00000112976	ENSMICT00000016130	ENSPPYT00000022830	ENSOPRT00000010316	ENSOCUT00000009379	ENSRNOT00000032204	ENSSTOT00000007888	ENSTBET00000016374	ENSTSYT00000003660
ENST00000400968	ENSOGAT00000026238	ENSPTRT00000058396	ENSBTAT00000018350	ENSNLET00000023036	ENSGGOT00000040987	ENSCPOT00000000674	ENSDORT00000006152	ENSMMUT00000031864	ENSCJAT00000062939	ENSMUST00000159879	ENSMICT00000011504	ENSPPYT00000000470	ENSOPRT00000017247	ENSOCUT00000012816	ENSRNOT00000004701	ENSSTOT00000012311	ENSTBET00000006801	ENSTSYT00000004225
ENST00000378509	ENSOGAT00000016155	ENSPTRT00000055180	ENSBTAT00000004376	ENSNLET00000016617	ENSGGOT00000001748	ENSCPOT00000005654	ENSDORT00000006339	ENSMMUT00000041757	ENSCJAT00000023700	ENSMUST00000019143	ENSMICT00000001918	ENSPPYT00000021007	ENSOPRT00000016890	ENSOCUT00000017220	ENSRNOT00000012179	ENSSTOT00000012364	ENSTBET00000012812	ENSTSYT00000009670
ENST00000559878	ENSOGAT00000001848	ENSPTRT00000013055	ENSBTAT00000019823	ENSNLET00000008125	ENSGGOT00000014079	ENSCPOT00000013950	ENSDORT00000009484	ENSMMUT00000003636	ENSCJAT00000002193	ENSMUST00000034811	ENSMICT00000010783	ENSPPYT00000007640	ENSOPRT00000001868	ENSOCUT00000012304	ENSRNOT00000000212	ENSSTOT00000006005	ENSTBET00000007436	ENSTSYT00000008359
ENST00000397382	ENSOGAT00000005107	ENSPTRT00000011283	ENSBTAT00000016916	ENSNLET00000019008	ENSGGOT00000014598	ENSCPOT00000014186	ENSDORT00000004064	ENSMMUT00000011851	ENSCJAT00000063526	ENSMUST00000022786	ENSMICT00000009207	ENSPPYT00000006681	ENSOPRT00000001147	ENSOCUT00000013096	ENSRNOT00000017857	ENSSTOT00000007115	ENSTBET00000012561	ENSTSYT00000001534
ENST00000265351	ENSOGAT00000016332	ENSPTRT00000033664	ENSBTAT00000025250	ENSNLET00000005113	ENSGGOT00000007989	ENSCPOT00000010006	ENSDORT00000000436	ENSMMUT00000021307	ENSCJAT00000011167	ENSMUST00000087031	ENSMICT00000005035	ENSPPYT00000019360	ENSOPRT00000000147	ENSOCUT00000005518	ENSRNOT00000025921	ENSSTOT00000010674	ENSTBET00000002855	ENSTSYT00000011943
ENST00000295542	ENSOGAT00000006397	ENSPTRT00000002569	ENSBTAT00000014083	ENSNLET00000014838	ENSGGOT00000013923	ENSCPOT00000001157	ENSDORT00000006973	ENSMMUT00000032830	ENSCJAT00000017999	ENSMUST00000070820	ENSMICT00000007980	ENSPPYT00000000916	ENSOPRT00000014861	ENSOCUT00000007060	ENSRNOT00000027986	ENSSTOT00000008720	ENSTBET00000002869	ENSTSYT00000011089
ENST00000283285	ENSOGAT00000008897	ENSPTRT00000028428	ENSBTAT00000019209	ENSNLET00000005181	ENSGGOT00000010790	ENSCPOT00000014896	ENSDORT00000005887	ENSMMUT00000000987	ENSCJAT00000039679	ENSMUST00000023336	ENSMICT00000017237	ENSPPYT00000015766	ENSOPRT00000013669	ENSOCUT00000001351	ENSRNOT00000035485	ENSSTOT00000004138	ENSTBET00000016810	ENSTSYT00000000790
ENST00000397461	ENSOGAT00000029431	ENSPTRT00000027230	ENSBTAT00000060980	ENSNLET00000004046	ENSGGOT00000001084	ENSCPOT00000019495	ENSDORT00000014028	ENSMMUT00000002602	ENSCJAT00000030039	ENSMUST00000113264	ENSMICT00000016576	ENSPPYT00000015936	ENSOPRT00000004019	ENSOCUT00000011027	ENSRNOT00000007788	ENSSTOT00000000148	ENSTBET00000005618	ENSTSYT00000004277
ENST00000409283	ENSOGAT00000011667	ENSPTRT00000023267	ENSBTAT00000015797	ENSNLET00000001062	ENSGGOT00000002949	ENSCPOT00000008869	ENSDORT00000013457	ENSMMUT00000026013	ENSCJAT00000029922	ENSMUST00000056376	ENSMICT00000006862	ENSPPYT00000014942	ENSOPRT00000012247	ENSOCUT00000002439	ENSRNOT00000006963	ENSSTOT00000004698	ENSTBET00000016892	ENSTSYT00000005100
ENST00000234739	ENSOGAT00000003600	ENSPTRT00000002273	ENSBTAT00000003141	ENSNLET00000000286	ENSGGOT00000009340	ENSCPOT00000012318	ENSDORT00000003296	ENSMMUT00000023584	ENSCJAT00000004589	ENSMUST00000046521	ENSMICT00000005549	ENSPPYT00000001138	ENSOPRT00000008254	ENSOCUT00000012141	ENSRNOT00000023548	ENSSTOT00000000851	ENSTBET00000002380	ENSTSYT00000005936
ENST00000454584	ENSOGAT00000012012	ENSPTRT00000006502	ENSBTAT00000028855	ENSNLET00000022760	ENSGGOT00000028930	ENSCPOT00000015338	ENSDORT00000007787	ENSMMUT00000029287	ENSCJAT00000012440	ENSMUST00000107591	ENSMICT00000014082	ENSPPYT00000004070	ENSOPRT00000005344	ENSOCUT00000014464	ENSRNOT00000022499	ENSSTOT00000015649	ENSTBET00000011345	ENSTSYT00000002339
ENST00000439373	ENSOGAT00000002542	ENSPTRT00000058530	ENSBTAT00000034148	ENSNLET00000019274	ENSGGOT00000011789	ENSCPOT00000001589	ENSDORT00000001437	ENSMMUT00000032055	ENSCJAT00000019312	ENSMUST00000159679	ENSMICT00000012350	ENSPPYT00000000630	ENSOPRT00000014615	ENSOCUT00000015745	ENSRNOT00000039581	ENSSTOT00000003074	ENSTBET00000011670	ENSTSYT00000002758
ENST00000265970	ENSOGAT00000002132	ENSPTRT00000006426	ENSBTAT00000046627	ENSNLET00000022605	ENSGGOT00000026535	ENSCPOT00000022630	ENSDORT00000002510	ENSMMUT00000031726	ENSCJAT00000054257	ENSMUST00000170430	ENSMICT00000003732	ENSPPYT00000004119	ENSOPRT00000000168	ENSOCUT00000005551	ENSRNOT00000055120	ENSSTOT00000009221	ENSTBET00000008010	ENSTSYT00000002289
ENST00000300452	ENSOGAT00000003515	ENSPTRT00000039638	ENSBTAT00000061485	ENSNLET00000022392	ENSGGOT00000014842	ENSCPOT00000004615	ENSDORT00000016223	ENSMMUT00000019857	ENSCJAT00000039325	ENSMUST00000028137	ENSMICT00000017636	ENSPPYT00000022928	ENSOPRT00000001996	ENSOCUT00000016195	ENSRNOT00000038602	ENSSTOT00000008041	ENSTBET00000000756	ENSTSYT00000009028
ENST00000262593	ENSOGAT00000005110	ENSPTRT00000025424	ENSBTAT00000064819	ENSNLET00000008970	ENSGGOT00000025240	ENSCPOT00000012905	ENSDORT00000007240	ENSMMUT00000009897	ENSCJAT00000004992	ENSMUST00000029075	ENSMICT00000004138	ENSPPYT00000012940	ENSOPRT00000015981	ENSOCUT00000009222	ENSRNOT00000017654	ENSSTOT00000000567	ENSTBET00000012194	ENSTSYT00000006305
ENST00000309859	ENSOGAT00000015281	ENSPTRT00000014533	ENSBTAT00000003035	ENSNLET00000015373	ENSGGOT00000004249	ENSCPOT00000002684	ENSDORT00000011649	ENSMMUT00000028881	ENSCJAT00000040471	ENSMUST00000033160	ENSMICT00000007736	ENSPPYT00000008465	ENSOPRT00000011958	ENSOCUT00000008428	ENSRNOT00000025309	ENSSTOT00000010923	ENSTBET00000005977	ENSTSYT00000003422
ENST00000257910	ENSOGAT00000032527	ENSPTRT00000009465	ENSBTAT00000009419	ENSNLET00000021958	ENSGGOT00000033774	ENSCPOT00000011600	ENSDORT00000007266	ENSMMUT00000033346	ENSCJAT00000013220	ENSMUST00000060991	ENSMICT00000003778	ENSPPYT00000005568	ENSOPRT00000006618	ENSOCUT00000012465	ENSRNOT00000031272	ENSSTOT00000001130	ENSTBET00000010655	ENSTSYT00000011917
ENST00000300151	ENSOGAT00000001487	ENSPTRT00000006911	ENSBTAT00000028236	ENSNLET00000016741	ENSGGOT00000015755	ENSCPOT00000005089	ENSDORT00000015110	ENSMMUT00000031402	ENSCJAT00000031708	ENSMUST00000167199	ENSMICT00000011019	ENSPPYT00000003850	ENSOPRT00000014628	ENSOCUT00000013389	ENSRNOT00000028517	ENSSTOT00000007229	ENSTBET00000007948	ENSTSYT00000013334
ENST00000376496	ENSOGAT00000009739	ENSPTRT00000000345	ENSBTAT00000027587	ENSNLET00000012416	ENSGGOT00000012987	ENSCPOT00000003552	ENSDORT00000014042	ENSMMUT00000010967	ENSCJAT00000056091	ENSMUST00000030879	ENSMICT00000003386	ENSPPYT00000002242	ENSOPRT00000015881	ENSOCUT00000007301	ENSRNOT00000011287	ENSSTOT00000007641	ENSTBET00000009084	ENSTSYT00000006597
ENST00000393119	ENSOGAT00000006784	ENSPTRT00000017324	ENSBTAT00000049437	ENSNLET00000010385	ENSGGOT00000003156	ENSCPOT00000000512	ENSDORT00000012529	ENSMMUT00000016769	ENSCJAT00000033577	ENSMUST00000038196	ENSMICT00000015953	ENSPPYT00000010342	ENSOPRT00000000118	ENSOCUT00000010017	ENSRNOT00000011500	ENSSTOT00000009991	ENSTBET00000011764	ENSTSYT00000002627
ENST00000335257	ENSOGAT00000011435	ENSPTRT00000017538	ENSBTAT00000061409	ENSNLET00000001772	ENSGGOT00000006367	ENSCPOT00000011518	ENSDORT00000003695	ENSMMUT00000003007	ENSCJAT00000017006	ENSMUST00000040380	ENSMICT00000012916	ENSPPYT00000010004	ENSOPRT00000008751	ENSOCUT00000015600	ENSRNOT00000021493	ENSSTOT00000003687	ENSTBET00000006713	ENSTSYT00000000697
ENST00000458276	ENSOGAT00000014021	ENSPTRT00000027689	ENSBTAT00000030667	ENSNLET00000007519	ENSGGOT00000004899	ENSCPOT00000005706	ENSDORT00000003979	ENSMMUT00000001469	ENSCJAT00000003317	ENSMUST00000156520	ENSMICT00000012061	ENSPPYT00000016242	ENSOPRT00000003691	ENSOCUT00000006493	ENSRNOT00000000239	ENSSTOT00000002080	ENSTBET00000010639	ENSTSYT00000012876
ENST00000307864	ENSOGAT00000005039	ENSPTRT00000004855	ENSBTAT00000001550	ENSNLET00000014379	ENSGGOT00000005978	ENSCPOT00000006395	ENSDORT00000011221	ENSMMUT00000008969	ENSCJAT00000031900	ENSMUST00000067857	ENSMICT00000001632	ENSPPYT00000002852	ENSOPRT00000016490	ENSOCUT00000004539	ENSRNOT00000035982	ENSSTOT00000012755	ENSTBET00000007445	ENSTSYT00000013881
ENST00000297623	ENSOGAT00000026640	ENSPTRT00000038627	ENSBTAT00000011027	ENSNLET00000006287	ENSGGOT00000004959	ENSCPOT00000013473	ENSDORT00000000551	ENSMMUT00000029216	ENSCJAT00000016657	ENSMUST00000030152	ENSMICT00000002344	ENSPPYT00000022294	ENSOPRT00000009143	ENSOCUT00000004769	ENSRNOT00000017903	ENSSTOT00000010111	ENSTBET00000003462	ENSTSYT00000008840
ENST00000340934	ENSOGAT00000016018	ENSPTRT00000003658	ENSBTAT00000009990	ENSNLET00000017278	ENSGGOT00000000641	ENSCPOT00000013735	ENSDORT00000000987	ENSMMUT00000021067	ENSCJAT00000012821	ENSMUST00000057062	ENSMICT00000017180	ENSPPYT00000000212	ENSOPRT00000013917	ENSOCUT00000016384	ENSRNOT00000004949	ENSSTOT00000003079	ENSTBET00000016123	ENSTSYT00000005749
ENST00000356956	ENSOGAT00000005276	ENSPTRT00000034646	ENSBTAT00000037678	ENSNLET00000014432	ENSGGOT00000027820	ENSCPOT00000013334	ENSDORT00000009528	ENSMMUT00000021965	ENSCJAT00000023292	ENSMUST00000024599	ENSMICT00000007613	ENSPPYT00000019962	ENSOPRT00000006114	ENSOCUT00000008799	ENSRNOT00000021840	ENSSTOT00000009002	ENSTBET00000015137	ENSTSYT00000005892
ENST00000307921	ENSOGAT00000005903	ENSPTRT00000015418	ENSBTAT00000020810	ENSNLET00000016665	ENSGGOT00000014716	ENSCPOT00000012146	ENSDORT00000004779	ENSMMUT00000008077	ENSCJAT00000027681	ENSMUST00000120457	ENSMICT00000011986	ENSPPYT00000008887	ENSOPRT00000012525	ENSOCUT00000015536	ENSRNOT00000026309	ENSSTOT00000004180	ENSTBET00000015815	ENSTSYT00000000811
ENST00000394684	ENSOGAT00000004365	ENSPTRT00000045334	ENSBTAT00000022353	ENSNLET00000016561	ENSGGOT00000012641	ENSCPOT00000011858	ENSDORT00000010108	ENSMMUT00000025063	ENSCJAT00000060387	ENSMUST00000090246	ENSMICT00000008734	ENSPPYT00000017414	ENSOPRT00000003399	ENSOCUT00000009997	ENSRNOT00000015020	ENSSTOT00000001348	ENSTBET00000002343	ENSTSYT00000008953
ENST00000315251	ENSOGAT00000016940	ENSPTRT00000028139	ENSBTAT00000013227	ENSNLET00000009238	ENSGGOT00000001481	ENSCPOT00000025757	ENSDORT00000005168	ENSMMUT00000027059	ENSCJAT00000014801	ENSMUST00000067620	ENSMICT00000017511	ENSPPYT00000016039	ENSOPRT00000012546	ENSOCUT00000009078	ENSRNOT00000021407	ENSSTOT00000000494	ENSTBET00000005608	ENSTSYT00000013525
ENST00000264451	ENSOGAT00000000502	ENSPTRT00000046447	ENSBTAT00000061481	ENSNLET00000022013	ENSGGOT00000001874	ENSCPOT00000000377	ENSDORT00000005520	ENSMMUT00000032058	ENSCJAT00000037738	ENSMUST00000031129	ENSMICT00000016315	ENSPPYT00000017087	ENSOPRT00000013482	ENSOCUT00000017142	ENSRNOT00000003066	ENSSTOT00000005381	ENSTBET00000006090	ENSTSYT00000002154
ENST00000478927	ENSOGAT00000005045	ENSPTRT00000028502	ENSBTAT00000053553	ENSNLET00000006327	ENSGGOT00000005180	ENSCPOT00000004935	ENSDORT00000009732	ENSMMUT00000001286	ENSCJAT00000017345	ENSMUST00000002923	ENSMICT00000007998	ENSPPYT00000015716	ENSOPRT00000007633	ENSOCUT00000009505	ENSRNOT00000038439	ENSSTOT00000003927	ENSTBET00000016242	ENSTSYT00000006425
ENST00000237264	ENSOGAT00000006622	ENSPTRT00000034390	ENSBTAT00000049877	ENSNLET00000018729	ENSGGOT00000030494	ENSCPOT00000012014	ENSDORT00000001096	ENSMMUT00000032262	ENSCJAT00000063265	ENSMUST00000095794	ENSMICT00000015734	ENSPPYT00000019812	ENSOPRT00000017269	ENSOCUT00000007090	ENSRNOT00000014805	ENSSTOT00000006778	ENSTBET00000013134	ENSTSYT00000005756
ENST00000258439	ENSOGAT00000034481	ENSPTRT00000022707	ENSBTAT00000020807	ENSNLET00000000025	ENSGGOT00000005192	ENSCPOT00000006834	ENSDORT00000005855	ENSMMUT00000018057	ENSCJAT00000014676	ENSMUST00000035871	ENSMICT00000004694	ENSPPYT00000014002	ENSOPRT00000015428	ENSOCUT00000009859	ENSRNOT00000031577	ENSSTOT00000016347	ENSTBET00000005387	ENSTSYT00000010958
ENST00000377962	ENSOGAT00000015192	ENSPTRT00000010848	ENSBTAT00000035859	ENSNLET00000020135	ENSGGOT00000004027	ENSCPOT00000006584	ENSDORT00000013066	ENSMMUT00000023031	ENSCJAT00000007001	ENSMUST00000022603	ENSMICT00000014633	ENSPPYT00000006387	ENSOPRT00000013069	ENSOCUT00000005851	ENSRNOT00000017400	ENSSTOT00000004176	ENSTBET00000000828	ENSTSYT00000000040
ENST00000394511	ENSOGAT00000017089	ENSPTRT00000030479	ENSBTAT00000006007	ENSNLET00000020639	ENSGGOT00000033471	ENSCPOT00000012155	ENSDORT00000009982	ENSMMUT00000020279	ENSCJAT00000006649	ENSMUST00000057944	ENSMICT00000008666	ENSPPYT00000017452	ENSOPRT00000000426	ENSOCUT00000015031	ENSRNOT00000012676	ENSSTOT00000005628	ENSTBET00000015080	ENSTSYT00000008930
ENST00000261658	ENSOGAT00000014638	ENSPTRT00000014345	ENSBTAT00000028683	ENSNLET00000013123	ENSGGOT00000026267	ENSCPOT00000023305	ENSDORT00000015689	ENSMMUT00000011547	ENSCJAT00000030860	ENSMUST00000023365	ENSMICT00000016086	ENSPPYT00000008390	ENSOPRT00000012213	ENSOCUT00000005924	ENSRNOT00000004217	ENSSTOT00000007562	ENSTBET00000010877	ENSTSYT00000003740
ENST00000410020	ENSOGAT00000001158	ENSPTRT00000063779	ENSBTAT00000033154	ENSNLET00000013209	ENSGGOT00000007253	ENSCPOT00000010290	ENSDORT00000015057	ENSMMUT00000010022	ENSCJAT00000007260	ENSMUST00000081904	ENSMICT00000010258	ENSPPYT00000014285	ENSOPRT00000003097	ENSOCUT00000005284	ENSRNOT00000051257	ENSSTOT00000016013	ENSTBET00000005929	ENSTSYT00000007973
ENST00000543237	ENSOGAT00000005175	ENSPTRT00000011828	ENSBTAT00000061297	ENSNLET00000000627	ENSGGOT00000010263	ENSCPOT00000013350	ENSDORT00000009286	ENSMMUT00000039797	ENSCJAT00000053027	ENSMUST00000052472	ENSMICT00000010146	ENSPPYT00000006989	ENSOPRT00000011027	ENSOCUT00000000900	ENSRNOT00000030470	ENSSTOT00000001534	ENSTBET00000013333	ENSTSYT00000009227
ENST00000444387	ENSOGAT00000016060	ENSPTRT00000000580	ENSBTAT00000001431	ENSNLET00000010175	ENSGGOT00000014659	ENSCPOT00000002823	ENSDORT00000006971	ENSMMUT00000012371	ENSCJAT00000012483	ENSMUST00000105823	ENSMICT00000010607	ENSPPYT00000002109	ENSOPRT00000010965	ENSOCUT00000007118	ENSRNOT00000067834	ENSSTOT00000015693	ENSTBET00000008550	ENSTSYT00000001856
ENST00000511148	ENSOGAT00000031608	ENSPTRT00000029747	ENSBTAT00000004355	ENSNLET00000021138	ENSGGOT00000031609	ENSCPOT00000008413	ENSDORT00000008036	ENSMMUT00000008048	ENSCJAT00000024558	ENSMUST00000053250	ENSMICT00000000595	ENSPPYT00000017003	ENSOPRT00000002885	ENSOCUT00000001567	ENSRNOT00000022589	ENSSTOT00000009916	ENSTBET00000010737	ENSTSYT00000003707
ENST00000302759	ENSOGAT00000007843	ENSPTRT00000022916	ENSBTAT00000028226	ENSNLET00000001701	ENSGGOT00000023126	ENSCPOT00000013285	ENSDORT00000014603	ENSMMUT00000024818	ENSCJAT00000026251	ENSMUST00000028858	ENSMICT00000008319	ENSPPYT00000014104	ENSOPRT00000003037	ENSOCUT00000014014	ENSRNOT00000051783	ENSSTOT00000001914	ENSTBET00000008999	ENSTSYT00000001695
ENST00000275016	ENSOGAT00000000401	ENSPTRT00000033719	ENSBTAT00000004736	ENSNLET00000005824	ENSGGOT00000032096	ENSCPOT00000006795	ENSDORT00000002545	ENSMMUT00000027037	ENSCJAT00000023435	ENSMUST00000055172	ENSMICT00000017753	ENSPPYT00000019389	ENSOPRT00000010921	ENSOCUT00000000754	ENSRNOT00000029005	ENSSTOT00000013009	ENSTBET00000008135	ENSTSYT00000011075
ENST00000407644	ENSOGAT00000000725	ENSPTRT00000022385	ENSBTAT00000011966	ENSNLET00000013062	ENSGGOT00000005212	ENSCPOT00000005758	ENSDORT00000012675	ENSMMUT00000014817	ENSCJAT00000037946	ENSMUST00000032069	ENSMICT00000014595	ENSPPYT00000014300	ENSOPRT00000002792	ENSOCUT00000014453	ENSRNOT00000021491	ENSSTOT00000000808	ENSTBET00000017273	ENSTSYT00000002647
ENST00000333082	ENSOGAT00000014587	ENSPTRT00000024411	ENSBTAT00000024505	ENSNLET00000012003	ENSGGOT00000029817	ENSCPOT00000013902	ENSDORT00000008283	ENSMMUT00000006655	ENSCJAT00000041044	ENSMUST00000042452	ENSMICT00000014332	ENSPPYT00000012607	ENSOPRT00000003473	ENSOCUT00000016748	ENSRNOT00000029620	ENSSTOT00000016148	ENSTBET00000012881	ENSTSYT00000005227
ENST00000258991	ENSOGAT00000003875	ENSPTRT00000017493	ENSBTAT00000006196	ENSNLET00000019227	ENSGGOT00000011499	ENSCPOT00000003650	ENSDORT00000008565	ENSMMUT00000024638	ENSCJAT00000038038	ENSMUST00000042780	ENSMICT00000005722	ENSPPYT00000009980	ENSOPRT00000008151	ENSOCUT00000012113	ENSRNOT00000055114	ENSSTOT00000015414	ENSTBET00000006613	ENSTSYT00000013538
ENST00000369124	ENSOGAT00000006278	ENSPTRT00000002312	ENSBTAT00000006381	ENSNLET00000012483	ENSGGOT00000002032	ENSCPOT00000025266	ENSDORT00000005015	ENSMMUT00000019215	ENSCJAT00000001570	ENSMUST00000015889	ENSMICT00000015248	ENSPPYT00000001096	ENSOPRT00000010920	ENSOCUT00000006317	ENSRNOT00000028747	ENSSTOT00000014248	ENSTBET00000003287	ENSTSYT00000005288
ENST00000285420	ENSOGAT00000011301	ENSPTRT00000037775	ENSBTAT00000004524	ENSNLET00000003124	ENSGGOT00000015884	ENSCPOT00000013731	ENSDORT00000005620	ENSMMUT00000038964	ENSCJAT00000056306	ENSMUST00000117268	ENSMICT00000012273	ENSPPYT00000021858	ENSOPRT00000007654	ENSOCUT00000000218	ENSRNOT00000008725	ENSSTOT00000002951	ENSTBET00000017064	ENSTSYT00000013749
ENST00000219794	ENSOGAT00000014454	ENSPTRT00000014817	ENSBTAT00000013909	ENSNLET00000020598	ENSGGOT00000013644	ENSCPOT00000001006	ENSDORT00000002991	ENSMMUT00000022541	ENSCJAT00000005980	ENSMUST00000071056	ENSMICT00000001623	ENSPPYT00000008594	ENSOPRT00000010746	ENSOCUT00000002292	ENSRNOT00000026466	ENSSTOT00000000633	ENSTBET00000017294	ENSTSYT00000004762
ENST00000369111	ENSOGAT00000004845	ENSPTRT00000002314	ENSBTAT00000028634	ENSNLET00000012516	ENSGGOT00000017174	ENSCPOT00000006143	ENSDORT00000011692	ENSMMUT00000030102	ENSCJAT00000018959	ENSMUST00000036181	ENSMICT00000015263	ENSPPYT00000001094	ENSOPRT00000010072	ENSOCUT00000009030	ENSRNOT00000025523	ENSSTOT00000006024	ENSTBET00000002646	ENSTSYT00000012163
ENST00000553373	ENSOGAT00000010021	ENSPTRT00000011636	ENSBTAT00000019278	ENSNLET00000016111	ENSGGOT00000027974	ENSCPOT00000009562	ENSDORT00000000195	ENSMMUT00000039842	ENSCJAT00000014138	ENSMUST00000045905	ENSMICT00000014369	ENSPPYT00000006880	ENSOPRT00000015942	ENSOCUT00000005035	ENSRNOT00000012400	ENSSTOT00000008577	ENSTBET00000005879	ENSTSYT00000008116
ENST00000388918	ENSOGAT00000013048	ENSPTRT00000038438	ENSBTAT00000027945	ENSNLET00000022522	ENSGGOT00000016314	ENSCPOT00000014220	ENSDORT00000013534	ENSMMUT00000018654	ENSCJAT00000023160	ENSMUST00000102831	ENSMICT00000017521	ENSPPYT00000022404	ENSOPRT00000006459	ENSOCUT00000004219	ENSRNOT00000046503	ENSSTOT00000003658	ENSTBET00000011396	ENSTSYT00000013424
ENST00000356493	ENSOGAT00000008624	ENSPTRT00000038675	ENSBTAT00000015136	ENSNLET00000005873	ENSGGOT00000003230	ENSCPOT00000012151	ENSDORT00000010302	ENSMMUT00000016624	ENSCJAT00000017813	ENSMUST00000030169	ENSMICT00000005518	ENSPPYT00000022271	ENSOPRT00000010790	ENSOCUT00000006450	ENSRNOT00000013151	ENSSTOT00000015234	ENSTBET00000008787	ENSTSYT00000004639
ENST00000283025	ENSOGAT00000010527	ENSPTRT00000014293	ENSBTAT00000022625	ENSNLET00000012944	ENSGGOT00000007063	ENSCPOT00000006620	ENSDORT00000011812	ENSMMUT00000027722	ENSCJAT00000033295	ENSMUST00000043415	ENSMICT00000004051	ENSPPYT00000008351	ENSOPRT00000014386	ENSOCUT00000001109	ENSRNOT00000003465	ENSSTOT00000004498	ENSTBET00000008016	ENSTSYT00000004810
ENST00000296343	ENSOGAT00000012092	ENSPTRT00000047544	ENSBTAT00000061001	ENSNLET00000019799	ENSGGOT00000011548	ENSCPOT00000002078	ENSDORT00000011682	ENSMMUT00000009264	ENSCJAT00000032054	ENSMUST00000089684	ENSMICT00000010781	ENSPPYT00000016812	ENSOPRT00000004594	ENSOCUT00000012753	ENSRNOT00000046530	ENSSTOT00000008246	ENSTBET00000002867	ENSTSYT00000002268
ENST00000521688	ENSOGAT00000033483	ENSPTRT00000037945	ENSBTAT00000009712	ENSNLET00000005475	ENSGGOT00000016592	ENSCPOT00000011089	ENSDORT00000006207	ENSMMUT00000008535	ENSCJAT00000012432	ENSMUST00000060652	ENSMICT00000012937	ENSPPYT00000029367	ENSOPRT00000015047	ENSOCUT00000004685	ENSRNOT00000006402	ENSSTOT00000015268	ENSTBET00000000268	ENSTSYT00000005583
ENST00000542937	ENSOGAT00000025438	ENSPTRT00000025267	ENSBTAT00000000732	ENSNLET00000008743	ENSGGOT00000016003	ENSCPOT00000012948	ENSDORT00000007080	ENSMMUT00000013908	ENSCJAT00000033627	ENSMUST00000059954	ENSMICT00000011274	ENSPPYT00000012864	ENSOPRT00000014404	ENSOCUT00000000745	ENSRNOT00000022594	ENSSTOT00000005077	ENSTBET00000005803	ENSTSYT00000000728
ENST00000297063	ENSOGAT00000031996	ENSPTRT00000035267	ENSBTAT00000002605	ENSNLET00000002489	ENSGGOT00000005278	ENSCPOT00000008113	ENSDORT00000007049	ENSMMUT00000014975	ENSCJAT00000024036	ENSMUST00000058868	ENSMICT00000009320	ENSPPYT00000020545	ENSOPRT00000001395	ENSOCUT00000005152	ENSRNOT00000031893	ENSSTOT00000006624	ENSTBET00000011002	ENSTSYT00000010006
ENST00000350761	ENSOGAT00000006176	ENSPTRT00000049940	ENSBTAT00000015257	ENSNLET00000000988	ENSGGOT00000007410	ENSCPOT00000006948	ENSDORT00000015750	ENSMMUT00000003366	ENSCJAT00000052227	ENSMUST00000006286	ENSMICT00000003861	ENSPPYT00000009114	ENSOPRT00000002950	ENSOCUT00000000073	ENSRNOT00000067999	ENSSTOT00000005022	ENSTBET00000004941	ENSTSYT00000008492
ENST00000518525	ENSOGAT00000013607	ENSPTRT00000032386	ENSBTAT00000003604	ENSNLET00000000350	ENSGGOT00000003855	ENSCPOT00000015799	ENSDORT00000012082	ENSMMUT00000000826	ENSCJAT00000038087	ENSMUST00000020366	ENSMICT00000012630	ENSPPYT00000018783	ENSOPRT00000009568	ENSOCUT00000008186	ENSRNOT00000043036	ENSSTOT00000014695	ENSTBET00000007754	ENSTSYT00000010148
ENST00000228820	ENSOGAT00000008253	ENSPTRT00000008417	ENSBTAT00000049732	ENSNLET00000003870	ENSGGOT00000012428	ENSCPOT00000009872	ENSDORT00000011165	ENSMMUT00000025675	ENSCJAT00000002975	ENSMUST00000039680	ENSMICT00000008780	ENSPPYT00000004931	ENSOPRT00000006102	ENSOCUT00000016535	ENSRNOT00000027092	ENSSTOT00000014421	ENSTBET00000009783	ENSTSYT00000004275
ENST00000344768	ENSOGAT00000007085	ENSPTRT00000011611	ENSBTAT00000052708	ENSNLET00000015807	ENSGGOT00000002263	ENSCPOT00000003162	ENSDORT00000009872	ENSMMUT00000025682	ENSCJAT00000013492	ENSMUST00000057859	ENSMICT00000008702	ENSPPYT00000006866	ENSOPRT00000001283	ENSOCUT00000015129	ENSRNOT00000009610	ENSSTOT00000003287	ENSTBET00000013406	ENSTSYT00000009131
ENST00000358896	ENSOGAT00000016902	ENSPTRT00000049055	ENSBTAT00000019500	ENSNLET00000006506	ENSGGOT00000007417	ENSCPOT00000013887	ENSDORT00000001655	ENSMMUT00000030731	ENSCJAT00000030831	ENSMUST00000034375	ENSMICT00000011361	ENSPPYT00000008794	ENSOPRT00000013921	ENSOCUT00000010041	ENSRNOT00000026827	ENSSTOT00000002076	ENSTBET00000006453	ENSTSYT00000013145
ENST00000267102	ENSOGAT00000005453	ENSPTRT00000009061	ENSBTAT00000061378	ENSNLET00000022771	ENSGGOT00000034308	ENSCPOT00000003038	ENSDORT00000008221	ENSMMUT00000017916	ENSCJAT00000041019	ENSMUST00000023736	ENSMICT00000017550	ENSPPYT00000005305	ENSOPRT00000015414	ENSOCUT00000011587	ENSRNOT00000046287	ENSSTOT00000008775	ENSTBET00000001597	ENSTSYT00000010004
ENST00000216294	ENSOGAT00000016123	ENSPTRT00000011755	ENSBTAT00000027888	ENSNLET00000017876	ENSGGOT00000005058	ENSCPOT00000008514	ENSDORT00000013434	ENSMMUT00000024319	ENSCJAT00000037700	ENSMUST00000021532	ENSMICT00000002231	ENSPPYT00000006951	ENSOPRT00000008819	ENSOCUT00000014091	ENSRNOT00000012361	ENSSTOT00000004855	ENSTBET00000012071	ENSTSYT00000008800
ENST00000547058	ENSOGAT00000011583	ENSPTRT00000036651	ENSBTAT00000004732	ENSNLET00000003350	ENSGGOT00000008405	ENSCPOT00000023565	ENSDORT00000007425	ENSMMUT00000008307	ENSCJAT00000026854	ENSMUST00000063523	ENSMICT00000001147	ENSPPYT00000021078	ENSOPRT00000007652	ENSOCUT00000000389	ENSRNOT00000017611	ENSSTOT00000005922	ENSTBET00000009838	ENSTSYT00000011799
ENST00000342697	ENSOGAT00000008874	ENSPTRT00000037812	ENSBTAT00000037186	ENSNLET00000004492	ENSGGOT00000009226	ENSCPOT00000015573	ENSDORT00000014957	ENSMMUT00000007542	ENSCJAT00000000013	ENSMUST00000029865	ENSMICT00000015601	ENSPPYT00000021882	ENSOPRT00000007061	ENSOCUT00000016236	ENSRNOT00000010591	ENSSTOT00000004453	ENSTBET00000017062	ENSTSYT00000001853
ENST00000345003	ENSOGAT00000008324	ENSPTRT00000028939	ENSBTAT00000002261	ENSNLET00000003303	ENSGGOT00000009016	ENSCPOT00000001501	ENSDORT00000016120	ENSMMUT00000007773	ENSCJAT00000002817	ENSMUST00000119748	ENSMICT00000005413	ENSPPYT00000029378	ENSOPRT00000009516	ENSOCUT00000003515	ENSRNOT00000014837	ENSSTOT00000001685	ENSTBET00000008901	ENSTSYT00000014641
ENST00000269159	ENSOGAT00000006138	ENSPTRT00000018136	ENSBTAT00000061313	ENSNLET00000011409	ENSGGOT00000011411	ENSCPOT00000001457	ENSDORT00000006464	ENSMMUT00000048294	ENSCJAT00000010280	ENSMUST00000025403	ENSMICT00000004773	ENSPPYT00000010494	ENSOPRT00000005116	ENSOCUT00000017058	ENSRNOT00000025147	ENSSTOT00000012412	ENSTBET00000011609	ENSTSYT00000014462
ENST00000216452	ENSOGAT00000015792	ENSPTRT00000011843	ENSBTAT00000024352	ENSNLET00000000909	ENSGGOT00000001517	ENSCPOT00000003371	ENSDORT00000007977	ENSMMUT00000030014	ENSCJAT00000029756	ENSMUST00000072154	ENSMICT00000010826	ENSPPYT00000006998	ENSOPRT00000015225	ENSOCUT00000000582	ENSRNOT00000014775	ENSSTOT00000000248	ENSTBET00000001830	ENSTSYT00000008435
ENST00000377252	ENSOGAT00000013723	ENSPTRT00000004304	ENSBTAT00000012472	ENSNLET00000016229	ENSGGOT00000016246	ENSCPOT00000007372	ENSDORT00000005917	ENSMMUT00000021549	ENSCJAT00000016105	ENSMUST00000114702	ENSMICT00000001135	ENSPPYT00000002558	ENSOPRT00000009880	ENSOCUT00000014480	ENSRNOT00000000155	ENSSTOT00000005044	ENSTBET00000002666	ENSTSYT00000010946
ENST00000452315	ENSOGAT00000005693	ENSPTRT00000022316	ENSBTAT00000025918	ENSNLET00000012727	ENSGGOT00000007299	ENSCPOT00000000666	ENSDORT00000012175	ENSMMUT00000008409	ENSCJAT00000013076	ENSMUST00000093298	ENSMICT00000006431	ENSPPYT00000014340	ENSOPRT00000014849	ENSOCUT00000004913	ENSRNOT00000006573	ENSSTOT00000012565	ENSTBET00000008551	ENSTSYT00000000425
ENST00000278816	ENSOGAT00000001916	ENSPTRT00000024687	ENSBTAT00000025259	ENSNLET00000012446	ENSGGOT00000010154	ENSCPOT00000013251	ENSDORT00000013196	ENSMMUT00000032553	ENSCJAT00000009809	ENSMUST00000028911	ENSMICT00000014381	ENSPPYT00000012470	ENSOPRT00000010982	ENSOCUT00000015365	ENSRNOT00000009437	ENSSTOT00000010251	ENSTBET00000000610	ENSTSYT00000000384
ENST00000264595	ENSOGAT00000015195	ENSPTRT00000030870	ENSBTAT00000022716	ENSNLET00000021452	ENSGGOT00000007633	ENSCPOT00000002708	ENSDORT00000014411	ENSMMUT00000010339	ENSCJAT00000010572	ENSMUST00000033920	ENSMICT00000009150	ENSPPYT00000017692	ENSOPRT00000005468	ENSOCUT00000002030	ENSRNOT00000000120	ENSSTOT00000011870	ENSTBET00000006472	ENSTSYT00000005389
ENST00000315684	ENSOGAT00000001971	ENSPTRT00000016084	ENSBTAT00000044661	ENSNLET00000012716	ENSGGOT00000002893	ENSCPOT00000003334	ENSDORT00000011177	ENSMMUT00000002742	ENSCJAT00000028250	ENSMUST00000021278	ENSMICT00000013803	ENSPPYT00000009308	ENSOPRT00000016989	ENSOCUT00000010813	ENSRNOT00000007447	ENSSTOT00000005468	ENSTBET00000007841	ENSTSYT00000003739
ENST00000393561	ENSOGAT00000003761	ENSPTRT00000036276	ENSBTAT00000015166	ENSNLET00000010969	ENSGGOT00000010779	ENSCPOT00000013302	ENSDORT00000001570	ENSMMUT00000009200	ENSCJAT00000032189	ENSMUST00000002979	ENSMICT00000001255	ENSPPYT00000020866	ENSOPRT00000015756	ENSOCUT00000014698	ENSRNOT00000008321	ENSSTOT00000007502	ENSTBET00000014124	ENSTSYT00000010755
ENST00000322886	ENSOGAT00000010827	ENSPTRT00000009643	ENSBTAT00000022518	ENSNLET00000006259	ENSGGOT00000012698	ENSCPOT00000007447	ENSDORT00000003404	ENSMMUT00000032161	ENSCJAT00000037578	ENSMUST00000173471	ENSMICT00000013872	ENSPPYT00000005674	ENSOPRT00000001886	ENSOCUT00000004932	ENSRNOT00000031986	ENSSTOT00000007886	ENSTBET00000005424	ENSTSYT00000008233
ENST00000545117	ENSOGAT00000014872	ENSPTRT00000037821	ENSBTAT00000019090	ENSNLET00000004657	ENSGGOT00000011441	ENSCPOT00000010411	ENSDORT00000010306	ENSMMUT00000001375	ENSCJAT00000058560	ENSMUST00000022871	ENSMICT00000011761	ENSPPYT00000021892	ENSOPRT00000008933	ENSOCUT00000002662	ENSRNOT00000007255	ENSSTOT00000008042	ENSTBET00000003503	ENSTSYT00000004082
ENST00000304987	ENSOGAT00000012648	ENSPTRT00000007952	ENSBTAT00000027019	ENSNLET00000009279	ENSGGOT00000024475	ENSCPOT00000013535	ENSDORT00000009028	ENSMMUT00000043718	ENSCJAT00000020554	ENSMUST00000041375	ENSMICT00000015730	ENSPPYT00000004599	ENSOPRT00000012522	ENSOCUT00000012918	ENSRNOT00000066893	ENSSTOT00000007698	ENSTBET00000015150	ENSTSYT00000011713
ENST00000302506	ENSOGAT00000002997	ENSPTRT00000027832	ENSBTAT00000012611	ENSNLET00000008160	ENSGGOT00000003934	ENSCPOT00000001239	ENSDORT00000011077	ENSMMUT00000029466	ENSCJAT00000005181	ENSMUST00000170365	ENSMICT00000000734	ENSPPYT00000016180	ENSOPRT00000017210	ENSOCUT00000012339	ENSRNOT00000067208	ENSSTOT00000015150	ENSTBET00000004657	ENSTSYT00000003042
ENST00000528157	ENSOGAT00000034753	ENSPTRT00000028019	ENSBTAT00000045083	ENSNLET00000008978	ENSGGOT00000001044	ENSCPOT00000006241	ENSDORT00000009852	ENSMMUT00000006082	ENSCJAT00000010650	ENSMUST00000159283	ENSMICT00000013664	ENSPPYT00000016094	ENSOPRT00000016685	ENSOCUT00000010314	ENSRNOT00000019107	ENSSTOT00000008638	ENSTBET00000008596	ENSTSYT00000001224
ENST00000294409	ENSOGAT00000006965	ENSPTRT00000000850	ENSBTAT00000001544	ENSNLET00000012583	ENSGGOT00000009259	ENSCPOT00000013533	ENSDORT00000000188	ENSMMUT00000015950	ENSCJAT00000018991	ENSMUST00000105964	ENSMICT00000003722	ENSPPYT00000001948	ENSOPRT00000008286	ENSOCUT00000010894	ENSRNOT00000014506	ENSSTOT00000000089	ENSTBET00000014887	ENSTSYT00000002781
ENST00000261434	ENSOGAT00000027809	ENSPTRT00000029834	ENSBTAT00000019299	ENSNLET00000021812	ENSGGOT00000032179	ENSCPOT00000004596	ENSDORT00000001091	ENSMMUT00000019439	ENSCJAT00000001302	ENSMUST00000031101	ENSMICT00000016774	ENSPPYT00000017062	ENSOPRT00000008785	ENSOCUT00000009096	ENSRNOT00000003779	ENSSTOT00000002085	ENSTBET00000006409	ENSTSYT00000013877
ENST00000438362	ENSOGAT00000012133	ENSPTRT00000066905	ENSBTAT00000000901	ENSNLET00000006264	ENSGGOT00000002180	ENSCPOT00000000543	ENSDORT00000011610	ENSMMUT00000002788	ENSCJAT00000024495	ENSMUST00000029446	ENSMICT00000002051	ENSPPYT00000001200	ENSOPRT00000005010	ENSOCUT00000011966	ENSRNOT00000041529	ENSSTOT00000001673	ENSTBET00000011898	ENSTSYT00000003750
ENST00000270517	ENSOGAT00000007081	ENSPTRT00000019363	ENSBTAT00000020029	ENSNLET00000016067	ENSGGOT00000005492	ENSCPOT00000026641	ENSDORT00000003435	ENSMMUT00000029011	ENSCJAT00000010774	ENSMUST00000098937	ENSMICT00000006039	ENSPPYT00000011169	ENSOPRT00000012173	ENSOCUT00000004389	ENSRNOT00000019115	ENSSTOT00000003655	ENSTBET00000011666	ENSTSYT00000005065
ENST00000557943	ENSOGAT00000009275	ENSPTRT00000013491	ENSBTAT00000016570	ENSNLET00000010469	ENSGGOT00000009073	ENSCPOT00000006245	ENSDORT00000000236	ENSMMUT00000015439	ENSCJAT00000021134	ENSMUST00000034866	ENSMICT00000000961	ENSPPYT00000007880	ENSOPRT00000000758	ENSOCUT00000000770	ENSRNOT00000020544	ENSSTOT00000005958	ENSTBET00000012556	ENSTSYT00000007893
ENST00000219345	ENSOGAT00000012346	ENSPTRT00000015230	ENSBTAT00000009888	ENSNLET00000006594	ENSGGOT00000001568	ENSCPOT00000021863	ENSDORT00000010903	ENSMMUT00000014169	ENSCJAT00000030547	ENSMUST00000034377	ENSMICT00000011403	ENSPPYT00000008798	ENSOPRT00000013413	ENSOCUT00000008512	ENSRNOT00000026996	ENSSTOT00000013688	ENSTBET00000011534	ENSTSYT00000003479
ENST00000332509	ENSOGAT00000010002	ENSPTRT00000026838	ENSBTAT00000037697	ENSNLET00000019219	ENSGGOT00000017141	ENSCPOT00000026089	ENSDORT00000002592	ENSMMUT00000003843	ENSCJAT00000060211	ENSMUST00000174021	ENSMICT00000011565	ENSPPYT00000013717	ENSOPRT00000000431	ENSOCUT00000006137	ENSRNOT00000017108	ENSSTOT00000005303	ENSTBET00000012718	ENSTSYT00000007613
ENST00000265070	ENSOGAT00000014259	ENSPTRT00000031135	ENSBTAT00000036701	ENSNLET00000017980	ENSGGOT00000029451	ENSCPOT00000011539	ENSDORT00000009431	ENSMMUT00000019588	ENSCJAT00000036934	ENSMUST00000059680	ENSMICT00000002265	ENSPPYT00000017868	ENSOPRT00000014131	ENSOCUT00000011529	ENSRNOT00000017276	ENSSTOT00000015875	ENSTBET00000009927	ENSTSYT00000004192
ENST00000256858	ENSOGAT00000032985	ENSPTRT00000018478	ENSBTAT00000022164	ENSNLET00000011016	ENSGGOT00000015083	ENSCPOT00000015389	ENSDORT00000014883	ENSMMUT00000003127	ENSCJAT00000006428	ENSMUST00000086721	ENSMICT00000010965	ENSPPYT00000010743	ENSOPRT00000007057	ENSOCUT00000005300	ENSRNOT00000020892	ENSSTOT00000009294	ENSTBET00000007697	ENSTSYT00000006458
ENST00000504169	ENSOGAT00000031164	ENSPTRT00000030884	ENSBTAT00000001266	ENSNLET00000013659	ENSGGOT00000034535	ENSCPOT00000012222	ENSDORT00000015273	ENSMMUT00000002840	ENSCJAT00000010791	ENSMUST00000038738	ENSMICT00000002222	ENSPPYT00000017701	ENSOPRT00000011227	ENSOCUT00000005490	ENSRNOT00000030519	ENSSTOT00000014277	ENSTBET00000013347	ENSTSYT00000007784
ENST00000299721	ENSOGAT00000002575	ENSPTRT00000018452	ENSBTAT00000054988	ENSNLET00000010688	ENSGGOT00000006777	ENSCPOT00000014697	ENSDORT00000011949	ENSMMUT00000010608	ENSCJAT00000006718	ENSMUST00000025397	ENSMICT00000001932	ENSPPYT00000010732	ENSOPRT00000000328	ENSOCUT00000003191	ENSRNOT00000022718	ENSSTOT00000013125	ENSTBET00000011129	ENSTSYT00000005435
ENST00000304372	ENSOGAT00000000017	ENSPTRT00000060173	ENSBTAT00000045955	ENSNLET00000005904	ENSGGOT00000016016	ENSCPOT00000010970	ENSDORT00000005866	ENSMMUT00000007009	ENSCJAT00000031584	ENSMUST00000167294	ENSMICT00000007814	ENSPPYT00000008763	ENSOPRT00000013305	ENSOCUT00000010075	ENSRNOT00000022497	ENSSTOT00000016445	ENSTBET00000012428	ENSTSYT00000005462
ENST00000219281	ENSOGAT00000003275	ENSPTRT00000015070	ENSBTAT00000020923	ENSNLET00000004240	ENSGGOT00000017204	ENSCPOT00000014467	ENSDORT00000001887	ENSMMUT00000031892	ENSCJAT00000022803	ENSMUST00000034245	ENSMICT00000004899	ENSPPYT00000008705	ENSOPRT00000011795	ENSOCUT00000011060	ENSRNOT00000017960	ENSSTOT00000007543	ENSTBET00000001499	ENSTSYT00000006974
ENST00000368211	ENSOGAT00000016829	ENSPTRT00000046177	ENSBTAT00000001991	ENSNLET00000016265	ENSGGOT00000028955	ENSCPOT00000014780	ENSDORT00000013739	ENSMMUT00000000055	ENSCJAT00000011921	ENSMUST00000019854	ENSMICT00000008940	ENSPPYT00000000846	ENSOPRT00000000642	ENSOCUT00000001463	ENSRNOT00000035271	ENSSTOT00000007763	ENSTBET00000015321	ENSTSYT00000010228
ENST00000537246	ENSOGAT00000012510	ENSPTRT00000030642	ENSBTAT00000029806	ENSNLET00000007053	ENSGGOT00000002572	ENSCPOT00000004194	ENSDORT00000008070	ENSMMUT00000041726	ENSCJAT00000004846	ENSMUST00000048718	ENSMICT00000000563	ENSPPYT00000017552	ENSOPRT00000014041	ENSOCUT00000016134	ENSRNOT00000015508	ENSSTOT00000010845	ENSTBET00000017050	ENSTSYT00000000837
ENST00000250617	ENSOGAT00000004742	ENSPTRT00000049100	ENSBTAT00000023731	ENSNLET00000003761	ENSGGOT00000014043	ENSCPOT00000010606	ENSDORT00000005918	ENSMMUT00000019339	ENSCJAT00000027442	ENSMUST00000114736	ENSMICT00000017848	ENSPPYT00000024224	ENSOPRT00000002554	ENSOCUT00000000255	ENSRNOT00000064525	ENSSTOT00000006193	ENSTBET00000011012	ENSTSYT00000013115
ENST00000374049	ENSOGAT00000007650	ENSPTRT00000039369	ENSBTAT00000000242	ENSNLET00000021468	ENSGGOT00000031993	ENSCPOT00000007075	ENSDORT00000008245	ENSMMUT00000006920	ENSCJAT00000040162	ENSMUST00000080336	ENSMICT00000015369	ENSPPYT00000022790	ENSOPRT00000005796	ENSOCUT00000004649	ENSRNOT00000011799	ENSSTOT00000014838	ENSTBET00000011241	ENSTSYT00000009407
ENST00000413830	ENSOGAT00000015553	ENSPTRT00000030097	ENSBTAT00000005295	ENSNLET00000010139	ENSGGOT00000015386	ENSCPOT00000013921	ENSDORT00000013906	ENSMMUT00000003272	ENSCJAT00000026955	ENSMUST00000041516	ENSMICT00000003866	ENSPPYT00000017241	ENSOPRT00000007251	ENSOCUT00000016266	ENSRNOT00000003720	ENSSTOT00000003140	ENSTBET00000010068	ENSTSYT00000008943
ENST00000377833	ENSOGAT00000006658	ENSPTRT00000004269	ENSBTAT00000039417	ENSNLET00000015968	ENSGGOT00000008538	ENSCPOT00000007189	ENSDORT00000016267	ENSMMUT00000022609	ENSCJAT00000017385	ENSMUST00000091436	ENSMICT00000011552	ENSPPYT00000002535	ENSOPRT00000010999	ENSOCUT00000005098	ENSRNOT00000040052	ENSSTOT00000009377	ENSTBET00000000238	ENSTSYT00000001964
ENST00000380815	ENSOGAT00000005958	ENSPTRT00000032653	ENSBTAT00000015992	ENSNLET00000013872	ENSGGOT00000022915	ENSCPOT00000012370	ENSDORT00000006796	ENSMMUT00000008111	ENSCJAT00000038466	ENSMUST00000041859	ENSMICT00000003143	ENSPPYT00000018806	ENSOPRT00000017117	ENSOCUT00000015144	ENSRNOT00000023691	ENSSTOT00000011255	ENSTBET00000007504	ENSTSYT00000012010
ENST00000268655	ENSOGAT00000025859	ENSPTRT00000014187	ENSBTAT00000002117	ENSNLET00000012321	ENSGGOT00000016496	ENSCPOT00000024351	ENSDORT00000009750	ENSMMUT00000010585	ENSCJAT00000038509	ENSMUST00000041778	ENSMICT00000014551	ENSPPYT00000008298	ENSOPRT00000012597	ENSOCUT00000014503	ENSRNOT00000010059	ENSSTOT00000013176	ENSTBET00000003354	ENSTSYT00000006891
ENST00000268802	ENSOGAT00000004594	ENSPTRT00000015281	ENSBTAT00000027497	ENSNLET00000007105	ENSGGOT00000026969	ENSCPOT00000019922	ENSDORT00000000483	ENSMMUT00000009122	ENSCJAT00000029681	ENSMUST00000003946	ENSMICT00000012137	ENSPPYT00000008825	ENSOPRT00000006725	ENSOCUT00000004404	ENSRNOT00000034386	ENSSTOT00000001209	ENSTBET00000000702	ENSTSYT00000003061
ENST00000372406	ENSOGAT00000009792	ENSPTRT00000056139	ENSBTAT00000005512	ENSNLET00000022540	ENSGGOT00000001265	ENSCPOT00000006070	ENSDORT00000006286	ENSMMUT00000005969	ENSCJAT00000040013	ENSMUST00000056433	ENSMICT00000009630	ENSPPYT00000022975	ENSOPRT00000005498	ENSOCUT00000014847	ENSRNOT00000037742	ENSSTOT00000005892	ENSTBET00000011553	ENSTSYT00000003925
ENST00000370136	ENSOGAT00000005625	ENSPTRT00000058970	ENSBTAT00000053822	ENSNLET00000015024	ENSGGOT00000016409	ENSCPOT00000000701	ENSDORT00000015809	ENSMMUT00000046597	ENSCJAT00000000354	ENSMUST00000029573	ENSMICT00000005286	ENSPPYT00000001341	ENSOPRT00000012062	ENSOCUT00000007836	ENSRNOT00000020297	ENSSTOT00000013502	ENSTBET00000009038	ENSTSYT00000010819
ENST00000338458	ENSOGAT00000007782	ENSPTRT00000028161	ENSBTAT00000018734	ENSNLET00000009260	ENSGGOT00000000673	ENSCPOT00000000845	ENSDORT00000000277	ENSMMUT00000014193	ENSCJAT00000021342	ENSMUST00000049206	ENSMICT00000011591	ENSPPYT00000016026	ENSOPRT00000005104	ENSOCUT00000013277	ENSRNOT00000019511	ENSSTOT00000000365	ENSTBET00000016951	ENSTSYT00000003625
ENST00000380009	ENSOGAT00000008327	ENSPTRT00000048019	ENSBTAT00000026877	ENSNLET00000018030	ENSGGOT00000005812	ENSCPOT00000013889	ENSDORT00000004578	ENSMMUT00000001711	ENSCJAT00000020066	ENSMUST00000120573	ENSMICT00000006709	ENSPPYT00000018193	ENSOPRT00000005477	ENSOCUT00000010878	ENSRNOT00000029250	ENSSTOT00000016555	ENSTBET00000006320	ENSTSYT00000013768
ENST00000369681	ENSOGAT00000012338	ENSPTRT00000033996	ENSBTAT00000054027	ENSNLET00000016277	ENSGGOT00000002933	ENSCPOT00000000052	ENSDORT00000010583	ENSMMUT00000013376	ENSCJAT00000029544	ENSMUST00000174724	ENSMICT00000012782	ENSPPYT00000019562	ENSOPRT00000006946	ENSOCUT00000007139	ENSRNOT00000013908	ENSSTOT00000009374	ENSTBET00000010292	ENSTSYT00000010349
ENST00000457354	ENSOGAT00000006430	ENSPTRT00000011608	ENSBTAT00000014908	ENSNLET00000015775	ENSGGOT00000009811	ENSCPOT00000015584	ENSDORT00000014646	ENSMMUT00000002688	ENSCJAT00000013454	ENSMUST00000021471	ENSMICT00000008687	ENSPPYT00000006865	ENSOPRT00000002171	ENSOCUT00000003980	ENSRNOT00000009587	ENSSTOT00000016241	ENSTBET00000004277	ENSTSYT00000004866
ENST00000337363	ENSOGAT00000005734	ENSPTRT00000018420	ENSBTAT00000010727	ENSNLET00000010193	ENSGGOT00000031025	ENSCPOT00000003613	ENSDORT00000012696	ENSMMUT00000030402	ENSCJAT00000008003	ENSMUST00000043929	ENSMICT00000015448	ENSPPYT00000010712	ENSOPRT00000016855	ENSOCUT00000014517	ENSRNOT00000039702	ENSSTOT00000003414	ENSTBET00000013021	ENSTSYT00000010218
ENST00000259605	ENSOGAT00000002068	ENSPTRT00000045497	ENSBTAT00000007260	ENSNLET00000004878	ENSGGOT00000016395	ENSCPOT00000024596	ENSDORT00000005360	ENSMMUT00000023330	ENSCJAT00000020134	ENSMUST00000098098	ENSMICT00000017677	ENSPPYT00000022228	ENSOPRT00000004383	ENSOCUT00000014161	ENSRNOT00000018863	ENSSTOT00000005078	ENSTBET00000011999	ENSTSYT00000006300
ENST00000339839	ENSOGAT00000006078	ENSPTRT00000009744	ENSBTAT00000005881	ENSNLET00000007320	ENSGGOT00000034057	ENSCPOT00000009080	ENSDORT00000012105	ENSMMUT00000025630	ENSCJAT00000005515	ENSMUST00000020212	ENSMICT00000016684	ENSPPYT00000005730	ENSOPRT00000014664	ENSOCUT00000016919	ENSRNOT00000034639	ENSSTOT00000000734	ENSTBET00000015492	ENSTSYT00000008305
ENST00000299192	ENSOGAT00000011663	ENSPTRT00000014933	ENSBTAT00000022133	ENSNLET00000000981	ENSGGOT00000033587	ENSCPOT00000013944	ENSDORT00000004409	ENSMMUT00000028212	ENSCJAT00000003787	ENSMUST00000034079	ENSMICT00000012811	ENSPPYT00000008625	ENSOPRT00000014212	ENSOCUT00000014188	ENSRNOT00000020793	ENSSTOT00000001188	ENSTBET00000016540	ENSTSYT00000008400
ENST00000374391	ENSOGAT00000002585	ENSPTRT00000004581	ENSBTAT00000027080	ENSNLET00000020377	ENSGGOT00000032199	ENSCPOT00000003983	ENSDORT00000003390	ENSMMUT00000018848	ENSCJAT00000026038	ENSMUST00000026795	ENSMICT00000008714	ENSPPYT00000002681	ENSOPRT00000006811	ENSOCUT00000005427	ENSRNOT00000017633	ENSSTOT00000015952	ENSTBET00000007034	ENSTSYT00000001450
ENST00000320892	ENSOGAT00000013107	ENSPTRT00000021672	ENSBTAT00000061067	ENSNLET00000019668	ENSGGOT00000012412	ENSCPOT00000008188	ENSDORT00000010145	ENSMMUT00000019318	ENSCJAT00000019892	ENSMUST00000062149	ENSMICT00000006184	ENSPPYT00000014721	ENSOPRT00000016249	ENSOCUT00000016069	ENSRNOT00000009877	ENSSTOT00000003181	ENSTBET00000004432	ENSTSYT00000006453
ENST00000430451	ENSOGAT00000016720	ENSPTRT00000011756	ENSBTAT00000025211	ENSNLET00000017895	ENSGGOT00000016167	ENSCPOT00000022332	ENSDORT00000016197	ENSMMUT00000012247	ENSCJAT00000053627	ENSMUST00000065968	ENSMICT00000011457	ENSPPYT00000006952	ENSOPRT00000004719	ENSOCUT00000016865	ENSRNOT00000064208	ENSSTOT00000003898	ENSTBET00000011389	ENSTSYT00000000821
ENST00000514743	ENSOGAT00000011458	ENSPTRT00000030313	ENSBTAT00000064437	ENSNLET00000018114	ENSGGOT00000025421	ENSCPOT00000014887	ENSDORT00000003185	ENSMMUT00000031299	ENSCJAT00000013496	ENSMUST00000058626	ENSMICT00000012958	ENSPPYT00000017358	ENSOPRT00000013924	ENSOCUT00000009748	ENSRNOT00000022387	ENSSTOT00000011296	ENSTBET00000004466	ENSTSYT00000011568
ENST00000368488	ENSOGAT00000009077	ENSPTRT00000034264	ENSBTAT00000017179	ENSNLET00000017210	ENSGGOT00000003849	ENSCPOT00000005181	ENSDORT00000008982	ENSMMUT00000031200	ENSCJAT00000008048	ENSMUST00000095691	ENSMICT00000014567	ENSPPYT00000019744	ENSOPRT00000004919	ENSOCUT00000013849	ENSRNOT00000063920	ENSSTOT00000004891	ENSTBET00000000681	ENSTSYT00000013190
ENST00000373840	ENSOGAT00000012976	ENSPTRT00000039418	ENSBTAT00000023671	ENSNLET00000021820	ENSGGOT00000032739	ENSCPOT00000006756	ENSDORT00000016328	ENSMMUT00000007872	ENSCJAT00000039744	ENSMUST00000028238	ENSMICT00000003349	ENSPPYT00000022821	ENSOPRT00000016425	ENSOCUT00000013651	ENSRNOT00000025649	ENSSTOT00000013128	ENSTBET00000009873	ENSTSYT00000014136
ENST00000337526	ENSOGAT00000002723	ENSPTRT00000062142	ENSBTAT00000014745	ENSNLET00000011756	ENSGGOT00000032075	ENSCPOT00000003217	ENSDORT00000003398	ENSMMUT00000029977	ENSCJAT00000019617	ENSMUST00000102843	ENSMICT00000016356	ENSPPYT00000014403	ENSOPRT00000011150	ENSOCUT00000012050	ENSRNOT00000051817	ENSSTOT00000000469	ENSTBET00000015717	ENSTSYT00000006360
ENST00000268138	ENSOGAT00000003840	ENSPTRT00000013711	ENSBTAT00000003143	ENSNLET00000014946	ENSGGOT00000008540	ENSCPOT00000015512	ENSDORT00000007284	ENSMMUT00000001777	ENSCJAT00000024706	ENSMUST00000035977	ENSMICT00000008278	ENSPPYT00000007989	ENSOPRT00000015469	ENSOCUT00000011001	ENSRNOT00000031783	ENSSTOT00000006120	ENSTBET00000014852	ENSTSYT00000014121
ENST00000341552	ENSOGAT00000014106	ENSPTRT00000023969	ENSBTAT00000013615	ENSNLET00000011561	ENSGGOT00000010851	ENSCPOT00000015398	ENSDORT00000002780	ENSMMUT00000001127	ENSCJAT00000054288	ENSMUST00000094844	ENSMICT00000007063	ENSPPYT00000015337	ENSOPRT00000011006	ENSOCUT00000000209	ENSRNOT00000036987	ENSSTOT00000009367	ENSTBET00000010895	ENSTSYT00000001844
ENST00000540432	ENSOGAT00000003739	ENSPTRT00000001597	ENSBTAT00000017252	ENSNLET00000011892	ENSGGOT00000015515	ENSCPOT00000012541	ENSDORT00000007449	ENSMMUT00000032248	ENSCJAT00000034160	ENSMUST00000068952	ENSMICT00000010217	ENSPPYT00000001503	ENSOPRT00000017055	ENSOCUT00000000533	ENSRNOT00000055245	ENSSTOT00000008155	ENSTBET00000000483	ENSTSYT00000000383
ENST00000261714	ENSOGAT00000000827	ENSPTRT00000016524	ENSBTAT00000028490	ENSNLET00000004676	ENSGGOT00000005528	ENSCPOT00000005102	ENSDORT00000008206	ENSMMUT00000001463	ENSCJAT00000026863	ENSMUST00000021197	ENSMICT00000000349	ENSPPYT00000009512	ENSOPRT00000007925	ENSOCUT00000003645	ENSRNOT00000005125	ENSSTOT00000004206	ENSTBET00000005398	ENSTSYT00000011700
ENST00000366929	ENSOGAT00000010186	ENSPTRT00000003624	ENSBTAT00000007053	ENSNLET00000018064	ENSGGOT00000004617	ENSCPOT00000007720	ENSDORT00000004060	ENSMMUT00000005778	ENSCJAT00000025099	ENSMUST00000045288	ENSMICT00000001599	ENSPPYT00000000234	ENSOPRT00000010172	ENSOCUT00000005281	ENSRNOT00000003313	ENSSTOT00000001247	ENSTBET00000015035	ENSTSYT00000012716
ENST00000282030	ENSOGAT00000010414	ENSPTRT00000018330	ENSBTAT00000024078	ENSNLET00000013747	ENSGGOT00000014202	ENSCPOT00000011268	ENSDORT00000006424	ENSMMUT00000048212	ENSCJAT00000007745	ENSMUST00000161465	ENSMICT00000017494	ENSPPYT00000010655	ENSOPRT00000015767	ENSOCUT00000009705	ENSRNOT00000021717	ENSSTOT00000002562	ENSTBET00000015548	ENSTSYT00000000207
ENST00000264444	ENSOGAT00000008415	ENSPTRT00000022374	ENSBTAT00000001384	ENSNLET00000012969	ENSGGOT00000011330	ENSCPOT00000007233	ENSDORT00000001608	ENSMMUT00000014296	ENSCJAT00000059382	ENSMUST00000001184	ENSMICT00000004970	ENSPPYT00000014311	ENSOPRT00000006514	ENSOCUT00000015821	ENSRNOT00000023856	ENSSTOT00000000716	ENSTBET00000005526	ENSTSYT00000005732
ENST00000360409	ENSOGAT00000007412	ENSPTRT00000023814	ENSBTAT00000015885	ENSNLET00000009041	ENSGGOT00000009581	ENSCPOT00000004438	ENSDORT00000012350	ENSMMUT00000028192	ENSCJAT00000059879	ENSMUST00000114157	ENSMICT00000009383	ENSPPYT00000015234	ENSOPRT00000010875	ENSOCUT00000007983	ENSRNOT00000042562	ENSSTOT00000010233	ENSTBET00000004624	ENSTSYT00000009015
ENST00000340553	ENSOGAT00000011747	ENSPTRT00000022155	ENSBTAT00000020154	ENSNLET00000011066	ENSGGOT00000008205	ENSCPOT00000025644	ENSDORT00000007019	ENSMMUT00000029081	ENSCJAT00000021234	ENSMUST00000112238	ENSMICT00000014854	ENSPPYT00000014430	ENSOPRT00000007671	ENSOCUT00000009784	ENSRNOT00000061693	ENSSTOT00000000860	ENSTBET00000012363	ENSTSYT00000014704
ENST00000360057	ENSOGAT00000010420	ENSPTRT00000036722	ENSBTAT00000044036	ENSNLET00000004381	ENSGGOT00000006851	ENSCPOT00000003297	ENSDORT00000016338	ENSMMUT00000012094	ENSCJAT00000019287	ENSMUST00000067888	ENSMICT00000002271	ENSPPYT00000021146	ENSOPRT00000015260	ENSOCUT00000003405	ENSRNOT00000048203	ENSSTOT00000009103	ENSTBET00000014807	ENSTSYT00000005150
ENST00000368508	ENSOGAT00000005033	ENSPTRT00000034251	ENSBTAT00000065858	ENSNLET00000017055	ENSGGOT00000014124	ENSCPOT00000013124	ENSDORT00000007168	ENSMMUT00000033424	ENSCJAT00000008301	ENSMUST00000117992	ENSMICT00000007805	ENSPPYT00000019736	ENSOPRT00000015377	ENSOCUT00000003216	ENSRNOT00000000459	ENSSTOT00000006662	ENSTBET00000016563	ENSTSYT00000010589
ENST00000396013	ENSOGAT00000001230	ENSPTRT00000035282	ENSBTAT00000057451	ENSNLET00000003491	ENSGGOT00000013516	ENSCPOT00000004442	ENSDORT00000005579	ENSMMUT00000042245	ENSCJAT00000024456	ENSMUST00000039694	ENSMICT00000000942	ENSPPYT00000020535	ENSOPRT00000015477	ENSOCUT00000008937	ENSRNOT00000041099	ENSSTOT00000014754	ENSTBET00000010512	ENSTSYT00000010092
ENST00000260191	ENSOGAT00000004320	ENSPTRT00000008003	ENSBTAT00000029852	ENSNLET00000009345	ENSGGOT00000005110	ENSCPOT00000010927	ENSDORT00000014038	ENSMMUT00000009881	ENSCJAT00000023391	ENSMUST00000008734	ENSMICT00000010051	ENSPPYT00000004631	ENSOPRT00000012273	ENSOCUT00000007455	ENSRNOT00000009574	ENSSTOT00000002628	ENSTBET00000016653	ENSTSYT00000008831
ENST00000508632	ENSOGAT00000011041	ENSPTRT00000060138	ENSBTAT00000027443	ENSNLET00000022223	ENSGGOT00000013826	ENSCPOT00000025441	ENSDORT00000016565	ENSMMUT00000042275	ENSCJAT00000036557	ENSMUST00000087195	ENSMICT00000006142	ENSPPYT00000017115	ENSOPRT00000001250	ENSOCUT00000007903	ENSRNOT00000002991	ENSSTOT00000014973	ENSTBET00000012445	ENSTSYT00000009217
ENST00000304527	ENSOGAT00000012452	ENSPTRT00000045624	ENSBTAT00000022368	ENSNLET00000007398	ENSGGOT00000010776	ENSCPOT00000008963	ENSDORT00000002251	ENSMMUT00000041684	ENSCJAT00000052638	ENSMUST00000107664	ENSMICT00000004927	ENSPPYT00000017576	ENSOPRT00000014609	ENSOCUT00000007016	ENSRNOT00000015802	ENSSTOT00000009652	ENSTBET00000010129	ENSTSYT00000003776
ENST00000261637	ENSOGAT00000015143	ENSPTRT00000009835	ENSBTAT00000004204	ENSNLET00000007916	ENSGGOT00000027598	ENSCPOT00000003172	ENSDORT00000014767	ENSMMUT00000018092	ENSCJAT00000003736	ENSMUST00000004470	ENSMICT00000015789	ENSPPYT00000005773	ENSOPRT00000010626	ENSOCUT00000010496	ENSRNOT00000008317	ENSSTOT00000016018	ENSTBET00000012054	ENSTSYT00000003098
ENST00000336089	ENSOGAT00000012545	ENSPTRT00000064568	ENSBTAT00000000607	ENSNLET00000008912	ENSGGOT00000007103	ENSCPOT00000023354	ENSDORT00000007854	ENSMMUT00000028306	ENSCJAT00000052203	ENSMUST00000010192	ENSMICT00000016334	ENSPPYT00000016110	ENSOPRT00000015355	ENSOCUT00000002364	ENSRNOT00000022092	ENSSTOT00000016209	ENSTBET00000008324	ENSTSYT00000000075
ENST00000337227	ENSOGAT00000002920	ENSPTRT00000024696	ENSBTAT00000046618	ENSNLET00000012513	ENSGGOT00000008350	ENSCPOT00000002436	ENSDORT00000016357	ENSMMUT00000005882	ENSCJAT00000009977	ENSMUST00000028915	ENSMICT00000010742	ENSPPYT00000012476	ENSOPRT00000003391	ENSOCUT00000015069	ENSRNOT00000010678	ENSSTOT00000000818	ENSTBET00000008188	ENSTSYT00000004210
ENST00000455345	ENSOGAT00000002220	ENSPTRT00000065087	ENSBTAT00000061334	ENSNLET00000006180	ENSGGOT00000022861	ENSCPOT00000004435	ENSDORT00000002785	ENSMMUT00000032428	ENSCJAT00000005407	ENSMUST00000124618	ENSMICT00000001197	ENSPPYT00000016591	ENSOPRT00000011008	ENSOCUT00000016778	ENSRNOT00000013111	ENSSTOT00000005405	ENSTBET00000008031	ENSTSYT00000005893
ENST00000377122	ENSOGAT00000013738	ENSPTRT00000004305	ENSBTAT00000061339	ENSNLET00000016278	ENSGGOT00000005469	ENSCPOT00000013450	ENSDORT00000014082	ENSMMUT00000014819	ENSCJAT00000016038	ENSMUST00000124270	ENSMICT00000001140	ENSPPYT00000002559	ENSOPRT00000017310	ENSOCUT00000012217	ENSRNOT00000037601	ENSSTOT00000013983	ENSTBET00000007175	ENSTSYT00000002589
ENST00000188790	ENSOGAT00000012612	ENSPTRT00000023310	ENSBTAT00000010702	ENSNLET00000003463	ENSGGOT00000015152	ENSCPOT00000012111	ENSDORT00000001905	ENSMMUT00000004535	ENSCJAT00000011544	ENSMUST00000102732	ENSMICT00000005394	ENSPPYT00000014966	ENSOPRT00000015743	ENSOCUT00000005116	ENSRNOT00000048755	ENSSTOT00000004941	ENSTBET00000005202	ENSTSYT00000005894
ENST00000405578	ENSOGAT00000032402	ENSPTRT00000059907	ENSBTAT00000035212	ENSNLET00000011595	ENSGGOT00000002756	ENSCPOT00000003719	ENSDORT00000007467	ENSMMUT00000014643	ENSCJAT00000022142	ENSMUST00000018593	ENSMICT00000012696	ENSPPYT00000009220	ENSOPRT00000013132	ENSOCUT00000004794	ENSRNOT00000009082	ENSSTOT00000003272	ENSTBET00000007569	ENSTSYT00000005429
ENST00000392996	ENSOGAT00000029845	ENSPTRT00000008007	ENSBTAT00000014968	ENSNLET00000009351	ENSGGOT00000000320	ENSCPOT00000004196	ENSDORT00000011901	ENSMMUT00000009890	ENSCJAT00000023301	ENSMUST00000093852	ENSMICT00000010065	ENSPPYT00000004633	ENSOPRT00000012295	ENSOCUT00000007468	ENSRNOT00000045356	ENSSTOT00000003567	ENSTBET00000016870	ENSTSYT00000007548
ENST00000359834	ENSOGAT00000003380	ENSPTRT00000018052	ENSBTAT00000001995	ENSNLET00000008751	ENSGGOT00000000656	ENSCPOT00000015730	ENSDORT00000002601	ENSMMUT00000005754	ENSCJAT00000000187	ENSMUST00000168707	ENSMICT00000009442	ENSPPYT00000010555	ENSOPRT00000006012	ENSOCUT00000005876	ENSRNOT00000056250	ENSSTOT00000013641	ENSTBET00000000809	ENSTSYT00000003045
ENST00000278919	ENSOGAT00000005190	ENSPTRT00000008224	ENSBTAT00000012027	ENSNLET00000009502	ENSGGOT00000005210	ENSCPOT00000003206	ENSDORT00000008636	ENSMMUT00000011262	ENSCJAT00000016301	ENSMUST00000163816	ENSMICT00000003010	ENSPPYT00000004789	ENSOPRT00000009349	ENSOCUT00000014644	ENSRNOT00000008285	ENSSTOT00000004253	ENSTBET00000004675	ENSTSYT00000003688
ENST00000330542	ENSOGAT00000006462	ENSPTRT00000031826	ENSBTAT00000029302	ENSNLET00000015667	ENSGGOT00000010235	ENSCPOT00000010822	ENSDORT00000015204	ENSMMUT00000011465	ENSCJAT00000037671	ENSMUST00000025490	ENSMICT00000009010	ENSPPYT00000018308	ENSOPRT00000015515	ENSOCUT00000012160	ENSRNOT00000022017	ENSSTOT00000003269	ENSTBET00000007439	ENSTSYT00000011966
ENST00000534526	ENSOGAT00000011505	ENSPTRT00000008909	ENSBTAT00000007175	ENSNLET00000021116	ENSGGOT00000028493	ENSCPOT00000015420	ENSDORT00000004450	ENSMMUT00000006856	ENSCJAT00000035758	ENSMUST00000162671	ENSMICT00000017663	ENSPPYT00000005224	ENSOPRT00000011663	ENSOCUT00000013918	ENSRNOT00000002491	ENSSTOT00000012551	ENSTBET00000005408	ENSTSYT00000005032
ENST00000268699	ENSOGAT00000005437	ENSPTRT00000015684	ENSBTAT00000009333	ENSNLET00000020473	ENSGGOT00000016290	ENSCPOT00000010906	ENSDORT00000008795	ENSMMUT00000000587	ENSCJAT00000020381	ENSMUST00000093043	ENSMICT00000013975	ENSPPYT00000009011	ENSOPRT00000006782	ENSOCUT00000008152	ENSRNOT00000029999	ENSSTOT00000005624	ENSTBET00000011772	ENSTSYT00000005616
ENST00000326621	ENSOGAT00000015711	ENSPTRT00000008205	ENSBTAT00000030293	ENSNLET00000009488	ENSGGOT00000005821	ENSCPOT00000000604	ENSDORT00000002738	ENSMMUT00000011859	ENSCJAT00000015614	ENSMUST00000002008	ENSMICT00000001692	ENSPPYT00000004776	ENSOPRT00000015054	ENSOCUT00000002615	ENSRNOT00000042679	ENSSTOT00000016280	ENSTBET00000005943	ENSTSYT00000011575
ENST00000382142	ENSOGAT00000014261	ENSPTRT00000031137	ENSBTAT00000060955	ENSNLET00000017953	ENSGGOT00000009428	ENSCPOT00000002593	ENSDORT00000004437	ENSMMUT00000009235	ENSCJAT00000036891	ENSMUST00000038172	ENSMICT00000002270	ENSPPYT00000017873	ENSOPRT00000014151	ENSOCUT00000014941	ENSRNOT00000036633	ENSSTOT00000008716	ENSTBET00000017647	ENSTSYT00000010535
ENST00000538336	ENSOGAT00000001137	ENSPTRT00000035240	ENSBTAT00000033703	ENSNLET00000019634	ENSGGOT00000007501	ENSCPOT00000020218	ENSDORT00000007111	ENSMMUT00000016381	ENSCJAT00000017736	ENSMUST00000031795	ENSMICT00000000718	ENSPPYT00000020569	ENSOPRT00000002897	ENSOCUT00000010669	ENSRNOT00000007821	ENSSTOT00000009173	ENSTBET00000007384	ENSTSYT00000007048
ENST00000394957	ENSOGAT00000011926	ENSPTRT00000043609	ENSBTAT00000047028	ENSNLET00000020778	ENSGGOT00000008464	ENSCPOT00000003143	ENSDORT00000014479	ENSMMUT00000042827	ENSCJAT00000014115	ENSMUST00000105459	ENSMICT00000000086	ENSPPYT00000029343	ENSOPRT00000006276	ENSOCUT00000016251	ENSRNOT00000057752	ENSSTOT00000003509	ENSTBET00000010308	ENSTSYT00000007823
ENST00000435927	ENSOGAT00000004962	ENSPTRT00000003848	ENSBTAT00000029091	ENSNLET00000002324	ENSGGOT00000025364	ENSCPOT00000002715	ENSDORT00000001380	ENSMMUT00000008247	ENSCJAT00000036115	ENSMUST00000034463	ENSMICT00000004225	ENSPPYT00000000126	ENSOPRT00000000865	ENSOCUT00000000492	ENSRNOT00000025695	ENSSTOT00000004200	ENSTBET00000004238	ENSTSYT00000009223
ENST00000285116	ENSOGAT00000016662	ENSPTRT00000018395	ENSBTAT00000024245	ENSNLET00000015058	ENSGGOT00000016699	ENSCPOT00000013410	ENSDORT00000013702	ENSMMUT00000001036	ENSCJAT00000016688	ENSMUST00000040188	ENSMICT00000006425	ENSPPYT00000010697	ENSOPRT00000007145	ENSOCUT00000007924	ENSRNOT00000020637	ENSSTOT00000003360	ENSTBET00000013444	ENSTSYT00000000175
ENST00000261448	ENSOGAT00000014411	ENSPTRT00000002140	ENSBTAT00000015285	ENSNLET00000006367	ENSGGOT00000017087	ENSCPOT00000001724	ENSDORT00000013120	ENSMMUT00000012534	ENSCJAT00000024806	ENSMUST00000029454	ENSMICT00000002845	ENSPPYT00000001191	ENSOPRT00000006649	ENSOCUT00000010596	ENSRNOT00000021846	ENSSTOT00000015740	ENSTBET00000006060	ENSTSYT00000006753
ENST00000269209	ENSOGAT00000001240	ENSPTRT00000018276	ENSBTAT00000027036	ENSNLET00000008140	ENSGGOT00000010006	ENSCPOT00000026655	ENSDORT00000012068	ENSMMUT00000032392	ENSCJAT00000041045	ENSMUST00000049260	ENSMICT00000013173	ENSPPYT00000010616	ENSOPRT00000009518	ENSOCUT00000013757	ENSRNOT00000061278	ENSSTOT00000002306	ENSTBET00000007717	ENSTSYT00000004271
ENST00000551379	ENSOGAT00000007878	ENSPTRT00000009829	ENSBTAT00000018079	ENSNLET00000007778	ENSGGOT00000008173	ENSCPOT00000002196	ENSDORT00000001223	ENSMMUT00000045780	ENSCJAT00000004018	ENSMUST00000105296	ENSMICT00000013522	ENSPPYT00000005769	ENSOPRT00000010992	ENSOCUT00000007179	ENSRNOT00000009672	ENSSTOT00000011771	ENSTBET00000016709	ENSTSYT00000002983
ENST00000281772	ENSOGAT00000002785	ENSPTRT00000023876	ENSBTAT00000064044	ENSNLET00000009167	ENSGGOT00000011197	ENSCPOT00000001523	ENSDORT00000005847	ENSMMUT00000014713	ENSCJAT00000021222	ENSMUST00000042389	ENSMICT00000009966	ENSPPYT00000015271	ENSOPRT00000005229	ENSOCUT00000017007	ENSRNOT00000067589	ENSSTOT00000010331	ENSTBET00000011199	ENSTSYT00000003315
ENST00000373593	ENSOGAT00000024527	ENSPTRT00000000932	ENSBTAT00000012078	ENSNLET00000003075	ENSGGOT00000001586	ENSCPOT00000012104	ENSDORT00000000199	ENSMMUT00000047418	ENSCJAT00000013251	ENSMUST00000102591	ENSMICT00000017799	ENSPPYT00000001902	ENSOPRT00000000816	ENSOCUT00000007615	ENSRNOT00000000136	ENSSTOT00000012061	ENSTBET00000017200	ENSTSYT00000008645
ENST00000395673	ENSOGAT00000013463	ENSPTRT00000006638	ENSBTAT00000026177	ENSNLET00000022931	ENSGGOT00000010784	ENSCPOT00000012046	ENSDORT00000012848	ENSMMUT00000018368	ENSCJAT00000022227	ENSMUST00000028623	ENSMICT00000014693	ENSPPYT00000003979	ENSOPRT00000006849	ENSOCUT00000007361	ENSRNOT00000012161	ENSSTOT00000004494	ENSTBET00000016756	ENSTSYT00000007169
ENST00000411996	ENSOGAT00000011507	ENSPTRT00000057295	ENSBTAT00000037956	ENSNLET00000021189	ENSGGOT00000006671	ENSCPOT00000015422	ENSDORT00000011488	ENSMMUT00000046968	ENSCJAT00000035623	ENSMUST00000096229	ENSMICT00000017715	ENSPPYT00000005226	ENSOPRT00000007796	ENSOCUT00000000020	ENSRNOT00000002479	ENSSTOT00000011910	ENSTBET00000007728	ENSTSYT00000013710
ENST00000358273	ENSOGAT00000029719	ENSPTRT00000016543	ENSBTAT00000015699	ENSNLET00000013968	ENSGGOT00000007040	ENSCPOT00000006176	ENSDORT00000011469	ENSMMUT00000012031	ENSCJAT00000027268	ENSMUST00000071325	ENSMICT00000002870	ENSPPYT00000009529	ENSOPRT00000004014	ENSOCUT00000008588	ENSRNOT00000044938	ENSSTOT00000005531	ENSTBET00000008393	ENSTSYT00000009479
ENST00000424014	ENSOGAT00000009201	ENSPTRT00000010295	ENSBTAT00000009560	ENSNLET00000002313	ENSGGOT00000009205	ENSCPOT00000010112	ENSDORT00000003039	ENSMMUT00000018489	ENSCJAT00000040223	ENSMUST00000031334	ENSMICT00000006474	ENSPPYT00000006020	ENSOPRT00000000862	ENSOCUT00000003464	ENSRNOT00000001373	ENSSTOT00000006159	ENSTBET00000010889	ENSTSYT00000007438
ENST00000392544	ENSOGAT00000011377	ENSPTRT00000023457	ENSBTAT00000002963	ENSNLET00000007572	ENSGGOT00000033790	ENSCPOT00000004686	ENSDORT00000003774	ENSMMUT00000005599	ENSCJAT00000015929	ENSMUST00000112017	ENSMICT00000006412	ENSPPYT00000015048	ENSOPRT00000003850	ENSOCUT00000012156	ENSRNOT00000002174	ENSSTOT00000014083	ENSTBET00000011922	ENSTSYT00000002606
ENST00000376652	ENSOGAT00000031496	ENSPTRT00000042064	ENSBTAT00000013034	ENSNLET00000019909	ENSGGOT00000014958	ENSCPOT00000009674	ENSDORT00000007175	ENSMMUT00000044373	ENSCJAT00000022910	ENSMUST00000094467	ENSMICT00000005757	ENSPPYT00000012522	ENSOPRT00000011765	ENSOCUT00000015936	ENSRNOT00000009946	ENSSTOT00000006953	ENSTBET00000003679	ENSTSYT00000009767
ENST00000500337	ENSOGAT00000009109	ENSPTRT00000031260	ENSBTAT00000047186	ENSNLET00000013307	ENSGGOT00000004904	ENSCPOT00000001915	ENSDORT00000009735	ENSMMUT00000041749	ENSCJAT00000002459	ENSMUST00000099147	ENSMICT00000006810	ENSPPYT00000017954	ENSOPRT00000000382	ENSOCUT00000009354	ENSRNOT00000037659	ENSSTOT00000016335	ENSTBET00000004565	ENSTSYT00000004024
ENST00000300107	ENSOGAT00000013631	ENSPTRT00000013234	ENSBTAT00000011038	ENSNLET00000016115	ENSGGOT00000024131	ENSCPOT00000003434	ENSDORT00000016116	ENSMMUT00000009398	ENSCJAT00000013498	ENSMUST00000015501	ENSMICT00000014969	ENSPPYT00000007748	ENSOPRT00000010155	ENSOCUT00000007293	ENSRNOT00000048302	ENSSTOT00000003769	ENSTBET00000008005	ENSTSYT00000011711
ENST00000307365	ENSOGAT00000031677	ENSPTRT00000004911	ENSBTAT00000000187	ENSNLET00000020924	ENSGGOT00000002775	ENSCPOT00000001696	ENSDORT00000015070	ENSMMUT00000017227	ENSCJAT00000014311	ENSMUST00000020308	ENSMICT00000004229	ENSPPYT00000002826	ENSOPRT00000004970	ENSOCUT00000016765	ENSRNOT00000000703	ENSSTOT00000000691	ENSTBET00000007816	ENSTSYT00000005156
ENST00000300255	ENSOGAT00000009263	ENSPTRT00000025836	ENSBTAT00000023019	ENSNLET00000004664	ENSGGOT00000031026	ENSCPOT00000019999	ENSDORT00000014732	ENSMMUT00000014394	ENSCJAT00000017728	ENSMUST00000037539	ENSMICT00000016445	ENSPPYT00000013185	ENSOPRT00000013560	ENSOCUT00000012277	ENSRNOT00000002838	ENSSTOT00000015794	ENSTBET00000015596	ENSTSYT00000009824
ENST00000333371	ENSOGAT00000005757	ENSPTRT00000044446	ENSBTAT00000000029	ENSNLET00000015692	ENSGGOT00000008381	ENSCPOT00000003751	ENSDORT00000014070	ENSMMUT00000010541	ENSCJAT00000054713	ENSMUST00000032749	ENSMICT00000014855	ENSPPYT00000008019	ENSOPRT00000011306	ENSOCUT00000005470	ENSRNOT00000017862	ENSSTOT00000016540	ENSTBET00000014949	ENSTSYT00000012971
ENST00000307046	ENSOGAT00000003338	ENSPTRT00000044261	ENSBTAT00000063328	ENSNLET00000008149	ENSGGOT00000004966	ENSCPOT00000019487	ENSDORT00000000240	ENSMMUT00000003166	ENSCJAT00000027829	ENSMUST00000105300	ENSMICT00000001959	ENSPPYT00000005786	ENSOPRT00000004229	ENSOCUT00000014681	ENSRNOT00000005995	ENSSTOT00000015845	ENSTBET00000014003	ENSTSYT00000009101
ENST00000295379	ENSOGAT00000032410	ENSPTRT00000022341	ENSBTAT00000001312	ENSNLET00000012839	ENSGGOT00000013648	ENSCPOT00000021328	ENSDORT00000012857	ENSMMUT00000021101	ENSCJAT00000037433	ENSMUST00000032125	ENSMICT00000003740	ENSPPYT00000014325	ENSOPRT00000002695	ENSOCUT00000010214	ENSRNOT00000012365	ENSSTOT00000010353	ENSTBET00000004317	ENSTSYT00000001416
ENST00000361983	ENSOGAT00000001851	ENSPTRT00000049750	ENSBTAT00000019353	ENSNLET00000014773	ENSGGOT00000012837	ENSCPOT00000002985	ENSDORT00000005104	ENSMMUT00000012551	ENSCJAT00000025001	ENSMUST00000065887	ENSMICT00000015120	ENSPPYT00000002866	ENSOPRT00000001660	ENSOCUT00000002892	ENSRNOT00000000444	ENSSTOT00000007755	ENSTBET00000003122	ENSTSYT00000012564
ENST00000477853	ENSOGAT00000013108	ENSPTRT00000059560	ENSBTAT00000042854	ENSNLET00000010011	ENSGGOT00000000535	ENSCPOT00000000811	ENSDORT00000005808	ENSMMUT00000009373	ENSCJAT00000013424	ENSMUST00000042096	ENSMICT00000004011	ENSPPYT00000002143	ENSOPRT00000012729	ENSOCUT00000000272	ENSRNOT00000068168	ENSSTOT00000015019	ENSTBET00000013017	ENSTSYT00000011787
ENST00000393094	ENSOGAT00000007207	ENSPTRT00000007919	ENSBTAT00000061240	ENSNLET00000009230	ENSGGOT00000026789	ENSCPOT00000011542	ENSDORT00000008149	ENSMMUT00000002530	ENSCJAT00000019650	ENSMUST00000163470	ENSMICT00000000377	ENSPPYT00000004573	ENSOPRT00000005961	ENSOCUT00000010913	ENSRNOT00000010956	ENSSTOT00000014166	ENSTBET00000006111	ENSTSYT00000009382
ENST00000373382	ENSOGAT00000005912	ENSPTRT00000004809	ENSBTAT00000022455	ENSNLET00000014753	ENSGGOT00000010477	ENSCPOT00000014586	ENSDORT00000016728	ENSMMUT00000005821	ENSCJAT00000025050	ENSMUST00000105447	ENSMICT00000014937	ENSPPYT00000002864	ENSOPRT00000001687	ENSOCUT00000001514	ENSRNOT00000067883	ENSSTOT00000009073	ENSTBET00000015350	ENSTSYT00000012243
ENST00000381031	ENSOGAT00000004876	ENSPTRT00000027123	ENSBTAT00000056392	ENSNLET00000020070	ENSGGOT00000001098	ENSCPOT00000006105	ENSDORT00000005113	ENSMMUT00000022848	ENSCJAT00000000337	ENSMUST00000146088	ENSMICT00000016771	ENSPPYT00000013872	ENSOPRT00000006082	ENSOCUT00000000374	ENSRNOT00000021582	ENSSTOT00000008127	ENSTBET00000017667	ENSTSYT00000013691
ENST00000264864	ENSOGAT00000029872	ENSPTRT00000029783	ENSBTAT00000047316	ENSNLET00000021442	ENSGGOT00000014128	ENSCPOT00000015548	ENSDORT00000000491	ENSMMUT00000005143	ENSCJAT00000025707	ENSMUST00000031081	ENSMICT00000017176	ENSPPYT00000017029	ENSOPRT00000016455	ENSOCUT00000015978	ENSRNOT00000005349	ENSSTOT00000016150	ENSTBET00000012584	ENSTSYT00000000481
ENST00000266126	ENSOGAT00000008238	ENSPTRT00000012011	ENSBTAT00000011424	ENSNLET00000020906	ENSGGOT00000000518	ENSCPOT00000008230	ENSDORT00000004892	ENSMMUT00000004128	ENSCJAT00000040431	ENSMUST00000004910	ENSMICT00000006099	ENSPPYT00000007086	ENSOPRT00000011886	ENSOCUT00000010175	ENSRNOT00000008730	ENSSTOT00000007287	ENSTBET00000008840	ENSTSYT00000000752
ENST00000395451	ENSOGAT00000005172	ENSPTRT00000074130	ENSBTAT00000043983	ENSNLET00000015374	ENSGGOT00000011714	ENSCPOT00000010194	ENSDORT00000015658	ENSMMUT00000014895	ENSCJAT00000033660	ENSMUST00000025417	ENSMICT00000001707	ENSPPYT00000018289	ENSOPRT00000003964	ENSOCUT00000012043	ENSRNOT00000023507	ENSSTOT00000006389	ENSTBET00000014186	ENSTSYT00000014395
ENST00000369291	ENSOGAT00000000211	ENSPTRT00000002252	ENSBTAT00000010691	ENSNLET00000007170	ENSGGOT00000007325	ENSCPOT00000023667	ENSDORT00000007837	ENSMMUT00000023511	ENSCJAT00000004453	ENSMUST00000029740	ENSMICT00000004902	ENSPPYT00000001127	ENSOPRT00000004264	ENSOCUT00000003054	ENSRNOT00000067925	ENSSTOT00000013565	ENSTBET00000006266	ENSTSYT00000004868
ENST00000427954	ENSOGAT00000005398	ENSPTRT00000017243	ENSBTAT00000028385	ENSNLET00000014331	ENSGGOT00000006544	ENSCPOT00000013407	ENSDORT00000000142	ENSMMUT00000024226	ENSCJAT00000036885	ENSMUST00000103164	ENSMICT00000009720	ENSPPYT00000009682	ENSOPRT00000013277	ENSOCUT00000002692	ENSRNOT00000004673	ENSSTOT00000011722	ENSTBET00000014500	ENSTSYT00000000351
ENST00000430055	ENSOGAT00000001311	ENSPTRT00000011896	ENSBTAT00000008922	ENSNLET00000002363	ENSGGOT00000006362	ENSCPOT00000012649	ENSDORT00000014901	ENSMMUT00000008112	ENSCJAT00000028811	ENSMUST00000161211	ENSMICT00000012992	ENSPPYT00000007036	ENSOPRT00000008951	ENSOCUT00000003769	ENSRNOT00000009483	ENSSTOT00000014084	ENSTBET00000002297	ENSTSYT00000008414
ENST00000355810	ENSOGAT00000032157	ENSPTRT00000050497	ENSBTAT00000054479	ENSNLET00000010302	ENSGGOT00000029713	ENSCPOT00000023717	ENSDORT00000001340	ENSMMUT00000029398	ENSCJAT00000028179	ENSMUST00000125462	ENSMICT00000000928	ENSPPYT00000017265	ENSOPRT00000002857	ENSOCUT00000003372	ENSRNOT00000052035	ENSSTOT00000009514	ENSTBET00000007971	ENSTSYT00000005236
ENST00000375592	ENSOGAT00000005098	ENSPTRT00000000465	ENSBTAT00000011217	ENSNLET00000009975	ENSGGOT00000014643	ENSCPOT00000004530	ENSDORT00000012524	ENSMMUT00000017856	ENSCJAT00000016175	ENSMUST00000030757	ENSMICT00000016900	ENSPPYT00000002179	ENSOPRT00000008569	ENSOCUT00000011411	ENSRNOT00000019195	ENSSTOT00000014744	ENSTBET00000013887	ENSTSYT00000014274
ENST00000340253	ENSOGAT00000001398	ENSPTRT00000032169	ENSBTAT00000027060	ENSNLET00000012921	ENSGGOT00000004717	ENSCPOT00000012542	ENSDORT00000008957	ENSMMUT00000023749	ENSCJAT00000041759	ENSMUST00000048688	ENSMICT00000002557	ENSPPYT00000018532	ENSOPRT00000013435	ENSOCUT00000017298	ENSRNOT00000025790	ENSSTOT00000009094	ENSTBET00000002498	ENSTSYT00000007478
ENST00000360131	ENSOGAT00000006830	ENSPTRT00000021878	ENSBTAT00000017831	ENSNLET00000003485	ENSGGOT00000016988	ENSCPOT00000011364	ENSDORT00000001040	ENSMMUT00000022557	ENSCJAT00000002433	ENSMUST00000114704	ENSMICT00000017021	ENSPPYT00000014602	ENSOPRT00000006881	ENSOCUT00000014885	ENSRNOT00000011594	ENSSTOT00000015510	ENSTBET00000015082	ENSTSYT00000011549
ENST00000512369	ENSOGAT00000005338	ENSPTRT00000030350	ENSBTAT00000010415	ENSNLET00000017498	ENSGGOT00000001138	ENSCPOT00000015183	ENSDORT00000015497	ENSMMUT00000041947	ENSCJAT00000029590	ENSMUST00000029806	ENSMICT00000002456	ENSPPYT00000017376	ENSOPRT00000012531	ENSOCUT00000001151	ENSRNOT00000014139	ENSSTOT00000010934	ENSTBET00000003215	ENSTSYT00000013841
ENST00000499023	ENSOGAT00000011314	ENSPTRT00000030732	ENSBTAT00000029844	ENSNLET00000007681	ENSGGOT00000023747	ENSCPOT00000003523	ENSDORT00000016294	ENSMMUT00000029979	ENSCJAT00000003611	ENSMUST00000150268	ENSMICT00000003222	ENSPPYT00000017599	ENSOPRT00000016705	ENSOCUT00000011225	ENSRNOT00000009113	ENSSTOT00000010146	ENSTBET00000011841	ENSTSYT00000007123
ENST00000259119	ENSOGAT00000008575	ENSPTRT00000029117	ENSBTAT00000005438	ENSNLET00000006688	ENSGGOT00000033607	ENSCPOT00000006752	ENSDORT00000005119	ENSMMUT00000012959	ENSCJAT00000012827	ENSMUST00000118204	ENSMICT00000017296	ENSPPYT00000016612	ENSOPRT00000015604	ENSOCUT00000013508	ENSRNOT00000013191	ENSSTOT00000008060	ENSTBET00000008724	ENSTSYT00000009968
ENST00000367615	ENSOGAT00000008027	ENSPTRT00000003138	ENSBTAT00000002806	ENSNLET00000020049	ENSGGOT00000015406	ENSCPOT00000006875	ENSDORT00000004700	ENSMMUT00000005336	ENSCJAT00000015863	ENSMUST00000027896	ENSMICT00000002673	ENSPPYT00000000550	ENSOPRT00000014249	ENSOCUT00000011106	ENSRNOT00000005425	ENSSTOT00000012132	ENSTBET00000013106	ENSTSYT00000008054
ENST00000320623	ENSOGAT00000004591	ENSPTRT00000015279	ENSBTAT00000032316	ENSNLET00000007071	ENSGGOT00000003310	ENSCPOT00000012549	ENSDORT00000000488	ENSMMUT00000039255	ENSCJAT00000004117	ENSMUST00000003947	ENSMICT00000012108	ENSPPYT00000008824	ENSOPRT00000010662	ENSOCUT00000004396	ENSRNOT00000017174	ENSSTOT00000016056	ENSTBET00000014512	ENSTSYT00000000709
ENST00000369209	ENSOGAT00000010174	ENSPTRT00000005672	ENSBTAT00000018304	ENSNLET00000021032	ENSGGOT00000000987	ENSCPOT00000003128	ENSDORT00000015666	ENSMMUT00000015268	ENSCJAT00000038137	ENSMUST00000066285	ENSMICT00000015604	ENSPPYT00000003241	ENSOPRT00000016971	ENSOCUT00000014113	ENSRNOT00000024548	ENSSTOT00000008902	ENSTBET00000013595	ENSTSYT00000009431
ENST00000366960	ENSOGAT00000013198	ENSPTRT00000003602	ENSBTAT00000001565	ENSNLET00000018505	ENSGGOT00000002169	ENSCPOT00000009576	ENSDORT00000000754	ENSMMUT00000007336	ENSCJAT00000030382	ENSMUST00000061611	ENSMICT00000014821	ENSPPYT00000000248	ENSOPRT00000016875	ENSOCUT00000013957	ENSRNOT00000005032	ENSSTOT00000001101	ENSTBET00000010459	ENSTSYT00000000486
ENST00000303372	ENSOGAT00000009217	ENSPTRT00000010298	ENSBTAT00000009562	ENSNLET00000002461	ENSGGOT00000009227	ENSCPOT00000010124	ENSDORT00000003044	ENSMMUT00000006491	ENSCJAT00000040248	ENSMUST00000165376	ENSMICT00000006498	ENSPPYT00000006023	ENSOPRT00000006392	ENSOCUT00000003477	ENSRNOT00000057966	ENSSTOT00000006207	ENSTBET00000011121	ENSTSYT00000005895
ENST00000377182	ENSOGAT00000016903	ENSPTRT00000038849	ENSBTAT00000023092	ENSNLET00000000952	ENSGGOT00000027884	ENSCPOT00000002465	ENSDORT00000000884	ENSMMUT00000011666	ENSCJAT00000003118	ENSMUST00000036069	ENSMICT00000001042	ENSPPYT00000022477	ENSOPRT00000012787	ENSOCUT00000000271	ENSRNOT00000034216	ENSSTOT00000009298	ENSTBET00000013409	ENSTSYT00000010249
ENST00000436586	ENSOGAT00000014243	ENSPTRT00000001685	ENSBTAT00000011751	ENSNLET00000012515	ENSGGOT00000030348	ENSCPOT00000026522	ENSDORT00000016535	ENSMMUT00000004117	ENSCJAT00000013922	ENSMUST00000079497	ENSMICT00000014471	ENSPPYT00000001460	ENSOPRT00000001192	ENSOCUT00000002225	ENSRNOT00000066199	ENSSTOT00000014320	ENSTBET00000017344	ENSTSYT00000000149
ENST00000296782	ENSOGAT00000032221	ENSPTRT00000050416	ENSBTAT00000008812	ENSNLET00000020921	ENSGGOT00000028351	ENSCPOT00000015235	ENSDORT00000010369	ENSMMUT00000002281	ENSCJAT00000014814	ENSMUST00000061656	ENSMICT00000006683	ENSPPYT00000017919	ENSOPRT00000007376	ENSOCUT00000012684	ENSRNOT00000029565	ENSSTOT00000005750	ENSTBET00000017713	ENSTSYT00000009260
ENST00000262394	ENSOGAT00000006339	ENSPTRT00000016404	ENSBTAT00000006593	ENSNLET00000001813	ENSGGOT00000015415	ENSCPOT00000010694	ENSDORT00000007447	ENSMMUT00000028454	ENSCJAT00000023994	ENSMUST00000017821	ENSMICT00000012869	ENSPPYT00000009452	ENSOPRT00000014295	ENSOCUT00000004643	ENSRNOT00000017949	ENSSTOT00000014710	ENSTBET00000013249	ENSTSYT00000009988
ENST00000360388	ENSOGAT00000001897	ENSPTRT00000034263	ENSBTAT00000047815	ENSNLET00000017152	ENSGGOT00000014839	ENSCPOT00000007149	ENSDORT00000011291	ENSMMUT00000031448	ENSCJAT00000008108	ENSMUST00000105473	ENSMICT00000014556	ENSPPYT00000019742	ENSOPRT00000007351	ENSOCUT00000015301	ENSRNOT00000000468	ENSSTOT00000004344	ENSTBET00000001712	ENSTSYT00000002397
ENST00000337334	ENSOGAT00000014310	ENSPTRT00000011602	ENSBTAT00000061312	ENSNLET00000015681	ENSGGOT00000022919	ENSCPOT00000015017	ENSDORT00000003797	ENSMMUT00000020566	ENSCJAT00000013266	ENSMUST00000169156	ENSMICT00000013597	ENSPPYT00000006862	ENSOPRT00000002084	ENSOCUT00000015514	ENSRNOT00000036933	ENSSTOT00000011953	ENSTBET00000005010	ENSTSYT00000004265
ENST00000378444	ENSOGAT00000006766	ENSPTRT00000040478	ENSBTAT00000065036	ENSNLET00000001766	ENSGGOT00000014946	ENSCPOT00000003922	ENSDORT00000014046	ENSMMUT00000007444	ENSCJAT00000007275	ENSMUST00000115513	ENSMICT00000006172	ENSPPYT00000023622	ENSOPRT00000005068	ENSOCUT00000017056	ENSRNOT00000041003	ENSSTOT00000003232	ENSTBET00000008192	ENSTSYT00000002024
ENST00000290158	ENSOGAT00000006157	ENSPTRT00000017140	ENSBTAT00000008650	ENSNLET00000002058	ENSGGOT00000008131	ENSCPOT00000006632	ENSDORT00000010067	ENSMMUT00000027779	ENSCJAT00000015549	ENSMUST00000001479	ENSMICT00000012063	ENSPPYT00000029414	ENSOPRT00000003816	ENSOCUT00000015431	ENSRNOT00000012376	ENSSTOT00000007919	ENSTBET00000010570	ENSTSYT00000014483
ENST00000299001	ENSOGAT00000012159	ENSPTRT00000007812	ENSBTAT00000019851	ENSNLET00000009087	ENSGGOT00000016339	ENSCPOT00000000070	ENSDORT00000013375	ENSMMUT00000012528	ENSCJAT00000007903	ENSMUST00000115644	ENSMICT00000006380	ENSPPYT00000004501	ENSOPRT00000004263	ENSOCUT00000002861	ENSRNOT00000012051	ENSSTOT00000006949	ENSTBET00000013379	ENSTSYT00000005588
ENST00000294811	ENSOGAT00000010708	ENSPTRT00000003554	ENSBTAT00000003693	ENSNLET00000002462	ENSGGOT00000006231	ENSCPOT00000014942	ENSDORT00000008889	ENSMMUT00000031041	ENSCJAT00000044121	ENSMUST00000160822	ENSMICT00000001228	ENSPPYT00000000279	ENSOPRT00000014275	ENSOCUT00000004030	ENSRNOT00000007341	ENSSTOT00000016546	ENSTBET00000017415	ENSTSYT00000010967
ENST00000375599	ENSOGAT00000005094	ENSPTRT00000000464	ENSBTAT00000018903	ENSNLET00000009971	ENSGGOT00000004342	ENSCPOT00000004622	ENSDORT00000001961	ENSMMUT00000047539	ENSCJAT00000016192	ENSMUST00000097813	ENSMICT00000002367	ENSPPYT00000002181	ENSOPRT00000008557	ENSOCUT00000013646	ENSRNOT00000037450	ENSSTOT00000014739	ENSTBET00000013741	ENSTSYT00000013247
ENST00000434715	ENSOGAT00000005239	ENSPTRT00000044815	ENSBTAT00000014053	ENSNLET00000022048	ENSGGOT00000002004	ENSCPOT00000013004	ENSDORT00000007138	ENSMMUT00000046301	ENSCJAT00000014087	ENSMUST00000026479	ENSMICT00000005468	ENSPPYT00000005556	ENSOPRT00000006547	ENSOCUT00000011265	ENSRNOT00000008120	ENSSTOT00000001158	ENSTBET00000000679	ENSTSYT00000010473
ENST00000368626	ENSOGAT00000009143	ENSPTRT00000034226	ENSBTAT00000026661	ENSNLET00000016673	ENSGGOT00000041135	ENSCPOT00000007023	ENSDORT00000000702	ENSMMUT00000020529	ENSCJAT00000008836	ENSMUST00000019913	ENSMICT00000003599	ENSPPYT00000019716	ENSOPRT00000005986	ENSOCUT00000001180	ENSRNOT00000000653	ENSSTOT00000010403	ENSTBET00000016324	ENSTSYT00000006419
ENST00000538538	ENSOGAT00000031715	ENSPTRT00000032421	ENSBTAT00000065187	ENSNLET00000000550	ENSGGOT00000022471	ENSCPOT00000014529	ENSDORT00000013936	ENSMMUT00000008263	ENSCJAT00000037660	ENSMUST00000062519	ENSMICT00000017725	ENSPPYT00000018684	ENSOPRT00000000672	ENSOCUT00000000038	ENSRNOT00000028186	ENSSTOT00000004369	ENSTBET00000005781	ENSTSYT00000003979
ENST00000243052	ENSOGAT00000017234	ENSPTRT00000009281	ENSBTAT00000005680	ENSNLET00000022482	ENSGGOT00000008412	ENSCPOT00000015249	ENSDORT00000002125	ENSMMUT00000000161	ENSCJAT00000037395	ENSMUST00000023132	ENSMICT00000008478	ENSPPYT00000005459	ENSOPRT00000017280	ENSOCUT00000013540	ENSRNOT00000055272	ENSSTOT00000008142	ENSTBET00000009436	ENSTSYT00000008502
ENST00000379925	ENSOGAT00000005134	ENSPTRT00000014958	ENSBTAT00000016585	ENSNLET00000001447	ENSGGOT00000011189	ENSCPOT00000013047	ENSDORT00000009324	ENSMMUT00000028955	ENSCJAT00000002809	ENSMUST00000047783	ENSMICT00000016831	ENSPPYT00000008643	ENSOPRT00000017282	ENSOCUT00000001739	ENSRNOT00000015876	ENSSTOT00000012684	ENSTBET00000004393	ENSTSYT00000009349
ENST00000268099	ENSOGAT00000005607	ENSPTRT00000013453	ENSBTAT00000007681	ENSNLET00000018752	ENSGGOT00000002707	ENSCPOT00000021126	ENSDORT00000014978	ENSMMUT00000018195	ENSCJAT00000030313	ENSMUST00000045791	ENSMICT00000009722	ENSPPYT00000007849	ENSOPRT00000010579	ENSOCUT00000003646	ENSRNOT00000066923	ENSSTOT00000012529	ENSTBET00000014169	ENSTSYT00000000553
ENST00000558170	ENSOGAT00000010023	ENSPTRT00000023211	ENSBTAT00000061551	ENSNLET00000011555	ENSGGOT00000003202	ENSCPOT00000004227	ENSDORT00000007230	ENSMMUT00000023668	ENSCJAT00000024189	ENSMUST00000028229	ENSMICT00000017462	ENSPPYT00000014906	ENSOPRT00000006203	ENSOCUT00000006517	ENSRNOT00000006350	ENSSTOT00000003810	ENSTBET00000013986	ENSTSYT00000009427
ENST00000393454	ENSOGAT00000006617	ENSPTRT00000012167	ENSBTAT00000017463	ENSNLET00000021696	ENSGGOT00000000706	ENSCPOT00000012234	ENSDORT00000009282	ENSMMUT00000006316	ENSCJAT00000034428	ENSMUST00000153627	ENSMICT00000016717	ENSPPYT00000007162	ENSOPRT00000000412	ENSOCUT00000002106	ENSRNOT00000005167	ENSSTOT00000004836	ENSTBET00000002580	ENSTSYT00000005916
ENST00000285928	ENSOGAT00000009230	ENSPTRT00000036524	ENSBTAT00000020944	ENSNLET00000016599	ENSGGOT00000034663	ENSCPOT00000006833	ENSDORT00000016100	ENSMMUT00000001309	ENSCJAT00000023646	ENSMUST00000070189	ENSMICT00000011306	ENSPPYT00000021005	ENSOPRT00000016881	ENSOCUT00000008830	ENSRNOT00000065620	ENSSTOT00000012354	ENSTBET00000012195	ENSTSYT00000009648
ENST00000334414	ENSOGAT00000014224	ENSPTRT00000009599	ENSBTAT00000020345	ENSNLET00000005358	ENSGGOT00000030893	ENSCPOT00000005761	ENSDORT00000011204	ENSMMUT00000015481	ENSCJAT00000002905	ENSMUST00000092167	ENSMICT00000007270	ENSPPYT00000005646	ENSOPRT00000002708	ENSOCUT00000012053	ENSRNOT00000006501	ENSSTOT00000013122	ENSTBET00000009011	ENSTSYT00000002142
ENST00000336686	ENSOGAT00000008430	ENSPTRT00000027572	ENSBTAT00000056583	ENSNLET00000006437	ENSGGOT00000001796	ENSCPOT00000006846	ENSDORT00000005832	ENSMMUT00000031718	ENSCJAT00000024389	ENSMUST00000098339	ENSMICT00000017580	ENSPPYT00000016303	ENSOPRT00000010728	ENSOCUT00000003171	ENSRNOT00000033210	ENSSTOT00000005469	ENSTBET00000008400	ENSTSYT00000005433
ENST00000324331	ENSOGAT00000001041	ENSPTRT00000032696	ENSBTAT00000024059	ENSNLET00000014668	ENSGGOT00000011886	ENSCPOT00000004038	ENSDORT00000014795	ENSMMUT00000022961	ENSCJAT00000042017	ENSMUST00000021857	ENSMICT00000003826	ENSPPYT00000018839	ENSOPRT00000000429	ENSOCUT00000014163	ENSRNOT00000021660	ENSSTOT00000012153	ENSTBET00000001793	ENSTSYT00000014678
ENST00000440333	ENSOGAT00000003196	ENSPTRT00000028562	ENSBTAT00000014796	ENSNLET00000000367	ENSGGOT00000031488	ENSCPOT00000005773	ENSDORT00000003168	ENSMMUT00000022992	ENSCJAT00000059262	ENSMUST00000004057	ENSMICT00000008018	ENSPPYT00000015686	ENSOPRT00000004749	ENSOCUT00000002686	ENSRNOT00000043230	ENSSTOT00000004434	ENSTBET00000003256	ENSTSYT00000002894
ENST00000258975	ENSOGAT00000006861	ENSPTRT00000017447	ENSBTAT00000013031	ENSNLET00000005656	ENSGGOT00000016920	ENSCPOT00000015135	ENSDORT00000013365	ENSMMUT00000020422	ENSCJAT00000038742	ENSMUST00000002048	ENSMICT00000003818	ENSPPYT00000009959	ENSOPRT00000014862	ENSOCUT00000014259	ENSRNOT00000063973	ENSSTOT00000009423	ENSTBET00000016994	ENSTSYT00000012184
ENST00000445506	ENSOGAT00000015145	ENSPTRT00000037073	ENSBTAT00000003751	ENSNLET00000002987	ENSGGOT00000022921	ENSCPOT00000021828	ENSDORT00000008190	ENSMMUT00000039133	ENSCJAT00000023135	ENSMUST00000026021	ENSMICT00000005419	ENSPPYT00000021421	ENSOPRT00000017328	ENSOCUT00000014196	ENSRNOT00000017339	ENSSTOT00000004036	ENSTBET00000012543	ENSTSYT00000000723
ENST00000381638	ENSOGAT00000008830	ENSPTRT00000015845	ENSBTAT00000061398	ENSNLET00000022077	ENSGGOT00000002035	ENSCPOT00000007524	ENSDORT00000013549	ENSMMUT00000026949	ENSCJAT00000022735	ENSMUST00000069395	ENSMICT00000015916	ENSPPYT00000009176	ENSOPRT00000011492	ENSOCUT00000014829	ENSRNOT00000036278	ENSSTOT00000015738	ENSTBET00000009181	ENSTSYT00000002719
ENST00000331159	ENSOGAT00000012427	ENSPTRT00000016703	ENSBTAT00000011251	ENSNLET00000000684	ENSGGOT00000011003	ENSCPOT00000011579	ENSDORT00000007712	ENSMMUT00000028895	ENSCJAT00000015788	ENSMUST00000045540	ENSMICT00000000635	ENSPPYT00000010351	ENSOPRT00000004400	ENSOCUT00000015725	ENSRNOT00000014943	ENSSTOT00000000068	ENSTBET00000002429	ENSTSYT00000000643
ENST00000369923	ENSOGAT00000013515	ENSPTRT00000058914	ENSBTAT00000017232	ENSNLET00000003354	ENSGGOT00000001765	ENSCPOT00000015868	ENSDORT00000006920	ENSMMUT00000030726	ENSCJAT00000005431	ENSMUST00000102625	ENSMICT00000016222	ENSPPYT00000001294	ENSOPRT00000008745	ENSOCUT00000008625	ENSRNOT00000027449	ENSSTOT00000011169	ENSTBET00000007015	ENSTSYT00000001109
ENST00000541869	ENSOGAT00000007372	ENSPTRT00000022962	ENSBTAT00000018830	ENSNLET00000002162	ENSGGOT00000013544	ENSCPOT00000015899	ENSDORT00000014310	ENSMMUT00000013214	ENSCJAT00000032030	ENSMUST00000103205	ENSMICT00000013252	ENSPPYT00000014121	ENSOPRT00000012745	ENSOCUT00000006059	ENSRNOT00000068384	ENSSTOT00000008169	ENSTBET00000014867	ENSTSYT00000003704
ENST00000311322	ENSOGAT00000000562	ENSPTRT00000037130	ENSBTAT00000017086	ENSNLET00000004312	ENSGGOT00000015491	ENSCPOT00000004098	ENSDORT00000003252	ENSMMUT00000006658	ENSCJAT00000027926	ENSMUST00000168401	ENSMICT00000010243	ENSPPYT00000021446	ENSOPRT00000013623	ENSOCUT00000008235	ENSRNOT00000016543	ENSSTOT00000001477	ENSTBET00000015295	ENSTSYT00000006403
ENST00000360351	ENSOGAT00000030902	ENSPTRT00000042392	ENSBTAT00000024134	ENSNLET00000009139	ENSGGOT00000029465	ENSCPOT00000008714	ENSDORT00000005836	ENSMMUT00000026348	ENSCJAT00000020978	ENSMUST00000114013	ENSMICT00000009892	ENSPPYT00000015266	ENSOPRT00000011935	ENSOCUT00000005164	ENSRNOT00000043627	ENSSTOT00000004536	ENSTBET00000003664	ENSTSYT00000010983
ENST00000317483	ENSOGAT00000005676	ENSPTRT00000033850	ENSBTAT00000007050	ENSNLET00000003542	ENSGGOT00000002947	ENSCPOT00000002526	ENSDORT00000005048	ENSMMUT00000025288	ENSCJAT00000019946	ENSMUST00000115174	ENSMICT00000014030	ENSPPYT00000019472	ENSOPRT00000010859	ENSOCUT00000004815	ENSRNOT00000016933	ENSSTOT00000010965	ENSTBET00000008337	ENSTSYT00000009813
ENST00000281382	ENSOGAT00000029838	ENSPTRT00000022130	ENSBTAT00000020863	ENSNLET00000021580	ENSGGOT00000022302	ENSCPOT00000008153	ENSDORT00000006083	ENSMMUT00000007580	ENSCJAT00000001247	ENSMUST00000024957	ENSMICT00000009126	ENSPPYT00000014451	ENSOPRT00000010252	ENSOCUT00000001128	ENSRNOT00000020600	ENSSTOT00000001368	ENSTBET00000000882	ENSTSYT00000002836
ENST00000343811	ENSOGAT00000014200	ENSPTRT00000025060	ENSBTAT00000018251	ENSNLET00000007926	ENSGGOT00000013568	ENSCPOT00000001363	ENSDORT00000014489	ENSMMUT00000020180	ENSCJAT00000056694	ENSMUST00000143663	ENSMICT00000005649	ENSPPYT00000012745	ENSOPRT00000012374	ENSOCUT00000005825	ENSRNOT00000055219	ENSSTOT00000016158	ENSTBET00000017535	ENSTSYT00000000310
ENST00000263774	ENSOGAT00000011609	ENSPTRT00000006726	ENSBTAT00000024600	ENSNLET00000018759	ENSGGOT00000014441	ENSCPOT00000006205	ENSDORT00000015594	ENSMMUT00000006095	ENSCJAT00000039680	ENSMUST00000005647	ENSMICT00000001088	ENSPPYT00000003929	ENSOPRT00000003295	ENSOCUT00000006353	ENSRNOT00000012425	ENSSTOT00000000623	ENSTBET00000006421	ENSTSYT00000002398
ENST00000338891	ENSOGAT00000005091	ENSPTRT00000056866	ENSBTAT00000020593	ENSNLET00000017353	ENSGGOT00000011023	ENSCPOT00000009816	ENSDORT00000010011	ENSMMUT00000018674	ENSCJAT00000007953	ENSMUST00000020003	ENSMICT00000016587	ENSPPYT00000019751	ENSOPRT00000004991	ENSOCUT00000005736	ENSRNOT00000000475	ENSSTOT00000012135	ENSTBET00000001001	ENSTSYT00000014609
ENST00000555605	ENSOGAT00000009120	ENSPTRT00000016213	ENSBTAT00000035038	ENSNLET00000018904	ENSGGOT00000001660	ENSCPOT00000000852	ENSDORT00000012685	ENSMMUT00000012511	ENSCJAT00000023663	ENSMUST00000050646	ENSMICT00000007110	ENSPPYT00000009376	ENSOPRT00000006956	ENSOCUT00000010452	ENSRNOT00000004002	ENSSTOT00000004269	ENSTBET00000014156	ENSTSYT00000006111
ENST00000356957	ENSOGAT00000010403	ENSPTRT00000046922	ENSBTAT00000019741	ENSNLET00000013009	ENSGGOT00000009764	ENSCPOT00000005119	ENSDORT00000004727	ENSMMUT00000019983	ENSCJAT00000028491	ENSMUST00000038424	ENSMICT00000008219	ENSPPYT00000008371	ENSOPRT00000004361	ENSOCUT00000016995	ENSRNOT00000003332	ENSSTOT00000016065	ENSTBET00000013420	ENSTSYT00000012454
ENST00000238616	ENSOGAT00000008254	ENSPTRT00000012019	ENSBTAT00000022098	ENSNLET00000020953	ENSGGOT00000025321	ENSCPOT00000004034	ENSDORT00000004896	ENSMMUT00000021264	ENSCJAT00000040507	ENSMUST00000040992	ENSMICT00000016003	ENSPPYT00000007093	ENSOPRT00000011969	ENSOCUT00000010203	ENSRNOT00000009300	ENSSTOT00000007375	ENSTBET00000001699	ENSTSYT00000005042
ENST00000378617	ENSOGAT00000008621	ENSPTRT00000038673	ENSBTAT00000061353	ENSNLET00000005921	ENSGGOT00000003225	ENSCPOT00000012131	ENSDORT00000010301	ENSMMUT00000016626	ENSCJAT00000017693	ENSMUST00000098109	ENSMICT00000005508	ENSPPYT00000022272	ENSOPRT00000010782	ENSOCUT00000002654	ENSRNOT00000013213	ENSSTOT00000015218	ENSTBET00000007100	ENSTSYT00000011951
ENST00000543976	ENSOGAT00000007773	ENSPTRT00000067767	ENSBTAT00000046850	ENSNLET00000002771	ENSGGOT00000006785	ENSCPOT00000013432	ENSDORT00000000515	ENSMMUT00000046567	ENSCJAT00000026359	ENSMUST00000095664	ENSMICT00000003400	ENSPPYT00000015968	ENSOPRT00000014630	ENSOCUT00000008160	ENSRNOT00000065696	ENSSTOT00000003189	ENSTBET00000008992	ENSTSYT00000011112
ENST00000334660	ENSOGAT00000013101	ENSPTRT00000012795	ENSBTAT00000038963	ENSNLET00000004435	ENSGGOT00000007346	ENSCPOT00000010672	ENSDORT00000012084	ENSMMUT00000024027	ENSCJAT00000040802	ENSMUST00000014221	ENSMICT00000014350	ENSPPYT00000007512	ENSOPRT00000016447	ENSOCUT00000013828	ENSRNOT00000056405	ENSSTOT00000014499	ENSTBET00000009074	ENSTSYT00000004062
ENST00000496811	ENSOGAT00000011262	ENSPTRT00000029008	ENSBTAT00000008082	ENSNLET00000004714	ENSGGOT00000041156	ENSCPOT00000007790	ENSDORT00000004849	ENSMMUT00000015892	ENSCJAT00000001872	ENSMUST00000029336	ENSMICT00000006638	ENSPPYT00000016533	ENSOPRT00000000179	ENSOCUT00000001182	ENSRNOT00000019824	ENSSTOT00000003928	ENSTBET00000013546	ENSTSYT00000002393
ENST00000367963	ENSOGAT00000003260	ENSPTRT00000034362	ENSBTAT00000025923	ENSNLET00000018591	ENSGGOT00000003644	ENSCPOT00000002571	ENSDORT00000015277	ENSMMUT00000007042	ENSCJAT00000005708	ENSMUST00000095784	ENSMICT00000003213	ENSPPYT00000019801	ENSOPRT00000006362	ENSOCUT00000008470	ENSRNOT00000061234	ENSSTOT00000010380	ENSTBET00000017159	ENSTSYT00000006398
ENST00000209728	ENSOGAT00000008546	ENSPTRT00000016796	ENSBTAT00000013706	ENSNLET00000002248	ENSGGOT00000015548	ENSCPOT00000010399	ENSDORT00000015184	ENSMMUT00000006719	ENSCJAT00000026092	ENSMUST00000092706	ENSMICT00000009735	ENSPPYT00000009893	ENSOPRT00000000888	ENSOCUT00000004898	ENSRNOT00000038228	ENSSTOT00000004727	ENSTBET00000014518	ENSTSYT00000007570
ENST00000287025	ENSOGAT00000004306	ENSPTRT00000037819	ENSBTAT00000001994	ENSNLET00000004584	ENSGGOT00000011579	ENSCPOT00000003531	ENSDORT00000010477	ENSMMUT00000012315	ENSCJAT00000040365	ENSMUST00000021991	ENSMICT00000009418	ENSPPYT00000021889	ENSOPRT00000008895	ENSOCUT00000004274	ENSRNOT00000006185	ENSSTOT00000008015	ENSTBET00000002603	ENSTSYT00000007338
ENST00000416516	ENSOGAT00000024434	ENSPTRT00000027566	ENSBTAT00000057378	ENSNLET00000006208	ENSGGOT00000016835	ENSCPOT00000002312	ENSDORT00000011086	ENSMMUT00000009528	ENSCJAT00000023986	ENSMUST00000111879	ENSMICT00000004658	ENSPPYT00000016308	ENSOPRT00000010623	ENSOCUT00000003654	ENSRNOT00000044742	ENSSTOT00000005331	ENSTBET00000014749	ENSTSYT00000002827
ENST00000260600	ENSOGAT00000000760	ENSPTRT00000021823	ENSBTAT00000032950	ENSNLET00000001362	ENSGGOT00000004101	ENSCPOT00000005624	ENSDORT00000007778	ENSMMUT00000013396	ENSCJAT00000015753	ENSMUST00000124505	ENSMICT00000001593	ENSPPYT00000014633	ENSOPRT00000008629	ENSOCUT00000015449	ENSRNOT00000005389	ENSSTOT00000006064	ENSTBET00000007633	ENSTSYT00000010033
ENST00000308511	ENSOGAT00000010585	ENSPTRT00000037185	ENSBTAT00000007194	ENSNLET00000020091	ENSGGOT00000002438	ENSCPOT00000011086	ENSDORT00000000934	ENSMMUT00000030941	ENSCJAT00000039692	ENSMUST00000035612	ENSMICT00000007758	ENSPPYT00000021477	ENSOPRT00000013279	ENSOCUT00000013233	ENSRNOT00000024895	ENSSTOT00000011669	ENSTBET00000010160	ENSTSYT00000001104
ENST00000502717	ENSOGAT00000004598	ENSPTRT00000032198	ENSBTAT00000035157	ENSNLET00000013069	ENSGGOT00000031962	ENSCPOT00000002641	ENSDORT00000009890	ENSMMUT00000016181	ENSCJAT00000056647	ENSMUST00000025524	ENSMICT00000002762	ENSPPYT00000018552	ENSOPRT00000008201	ENSOCUT00000007505	ENSRNOT00000024704	ENSSTOT00000008796	ENSTBET00000013179	ENSTSYT00000012752
ENST00000427714	ENSOGAT00000001714	ENSPTRT00000007572	ENSBTAT00000008644	ENSNLET00000021716	ENSGGOT00000007890	ENSCPOT00000003645	ENSDORT00000011067	ENSMMUT00000024474	ENSCJAT00000030608	ENSMUST00000057023	ENSMICT00000002250	ENSPPYT00000004369	ENSOPRT00000016932	ENSOCUT00000010110	ENSRNOT00000046943	ENSSTOT00000007909	ENSTBET00000012459	ENSTSYT00000001207
ENST00000369705	ENSOGAT00000014422	ENSPTRT00000033991	ENSBTAT00000007465	ENSNLET00000016194	ENSGGOT00000026938	ENSCPOT00000002690	ENSDORT00000014298	ENSMMUT00000017651	ENSCJAT00000054530	ENSMUST00000034989	ENSMICT00000013755	ENSPPYT00000019556	ENSOPRT00000011648	ENSOCUT00000010816	ENSRNOT00000013244	ENSSTOT00000001291	ENSTBET00000010724	ENSTSYT00000010910
ENST00000230122	ENSOGAT00000015522	ENSPTRT00000034165	ENSBTAT00000064541	ENSNLET00000016146	ENSGGOT00000000432	ENSCPOT00000003153	ENSDORT00000006492	ENSMMUT00000005206	ENSCJAT00000011091	ENSMUST00000080771	ENSMICT00000014482	ENSPPYT00000019678	ENSOPRT00000012652	ENSOCUT00000012551	ENSRNOT00000057175	ENSSTOT00000011458	ENSTBET00000010997	ENSTSYT00000008489
ENST00000537135	ENSOGAT00000010075	ENSPTRT00000014449	ENSBTAT00000026109	ENSNLET00000015893	ENSGGOT00000023420	ENSCPOT00000002437	ENSDORT00000000815	ENSMMUT00000003853	ENSCJAT00000018614	ENSMUST00000008878	ENSMICT00000011121	ENSPPYT00000008422	ENSOPRT00000002311	ENSOCUT00000001921	ENSRNOT00000021441	ENSSTOT00000015214	ENSTBET00000000477	ENSTSYT00000010339
ENST00000388914	ENSOGAT00000016367	ENSPTRT00000030134	ENSBTAT00000016131	ENSNLET00000010391	ENSGGOT00000007544	ENSCPOT00000011824	ENSDORT00000009455	ENSMMUT00000029414	ENSCJAT00000028612	ENSMUST00000060930	ENSMICT00000017894	ENSPPYT00000017269	ENSOPRT00000002990	ENSOCUT00000013559	ENSRNOT00000003009	ENSSTOT00000012611	ENSTBET00000003622	ENSTSYT00000002033
ENST00000304992	ENSOGAT00000012790	ENSPTRT00000074067	ENSBTAT00000046260	ENSNLET00000001115	ENSGGOT00000029657	ENSCPOT00000010473	ENSDORT00000006186	ENSMMUT00000042437	ENSCJAT00000003812	ENSMUST00000102510	ENSMICT00000010340	ENSPPYT00000009121	ENSOPRT00000003171	ENSOCUT00000000085	ENSRNOT00000005016	ENSSTOT00000005178	ENSTBET00000006509	ENSTSYT00000014122
ENST00000314103	ENSOGAT00000031901	ENSPTRT00000025906	ENSBTAT00000015764	ENSNLET00000005689	ENSGGOT00000015782	ENSCPOT00000006398	ENSDORT00000012469	ENSMMUT00000010350	ENSCJAT00000005314	ENSMUST00000117099	ENSMICT00000013152	ENSPPYT00000013235	ENSOPRT00000008967	ENSOCUT00000014011	ENSRNOT00000002299	ENSSTOT00000001424	ENSTBET00000009944	ENSTSYT00000007595
ENST00000507110	ENSOGAT00000001150	ENSPTRT00000031269	ENSBTAT00000000091	ENSNLET00000013549	ENSGGOT00000016150	ENSCPOT00000002094	ENSDORT00000014828	ENSMMUT00000022731	ENSCJAT00000002654	ENSMUST00000022245	ENSMICT00000008010	ENSPPYT00000017959	ENSOPRT00000013364	ENSOCUT00000004204	ENSRNOT00000016190	ENSSTOT00000015850	ENSTBET00000005816	ENSTSYT00000009379
ENST00000388884	ENSOGAT00000010165	ENSPTRT00000005671	ENSBTAT00000018285	ENSNLET00000021013	ENSGGOT00000000985	ENSCPOT00000003127	ENSDORT00000015664	ENSMMUT00000015267	ENSCJAT00000038107	ENSMUST00000028299	ENSMICT00000015594	ENSPPYT00000003240	ENSOPRT00000001858	ENSOCUT00000013112	ENSRNOT00000039453	ENSSTOT00000008894	ENSTBET00000013559	ENSTSYT00000003118
ENST00000229416	ENSOGAT00000007023	ENSPTRT00000033810	ENSBTAT00000049040	ENSNLET00000004441	ENSGGOT00000006751	ENSCPOT00000008389	ENSDORT00000009967	ENSMMUT00000012140	ENSCJAT00000058894	ENSMUST00000034905	ENSMICT00000003750	ENSPPYT00000019448	ENSOPRT00000005153	ENSOCUT00000012806	ENSRNOT00000033196	ENSSTOT00000000903	ENSTBET00000015361	ENSTSYT00000006044
ENST00000326172	ENSOGAT00000034837	ENSPTRT00000028384	ENSBTAT00000047710	ENSNLET00000004288	ENSGGOT00000029549	ENSCPOT00000009812	ENSDORT00000009331	ENSMMUT00000030353	ENSCJAT00000000878	ENSMUST00000096026	ENSMICT00000002305	ENSPPYT00000015794	ENSOPRT00000004330	ENSOCUT00000000134	ENSRNOT00000041940	ENSSTOT00000011770	ENSTBET00000007659	ENSTSYT00000014139
ENST00000007722	ENSOGAT00000005144	ENSPTRT00000017219	ENSBTAT00000025974	ENSNLET00000011375	ENSGGOT00000013617	ENSCPOT00000003626	ENSDORT00000013492	ENSMMUT00000041887	ENSCJAT00000013622	ENSMUST00000120375	ENSMICT00000007211	ENSPPYT00000010424	ENSOPRT00000013111	ENSOCUT00000009992	ENSRNOT00000005644	ENSSTOT00000014849	ENSTBET00000013828	ENSTSYT00000014402
ENST00000488594	ENSOGAT00000013762	ENSPTRT00000003893	ENSBTAT00000017376	ENSNLET00000003586	ENSGGOT00000008935	ENSCPOT00000001717	ENSDORT00000008647	ENSMMUT00000044383	ENSCJAT00000057413	ENSMUST00000170957	ENSMICT00000007363	ENSPPYT00000000105	ENSOPRT00000011115	ENSOCUT00000013580	ENSRNOT00000022483	ENSSTOT00000004254	ENSTBET00000004274	ENSTSYT00000010833
ENST00000355849	ENSOGAT00000005153	ENSPTRT00000048098	ENSBTAT00000002827	ENSNLET00000013717	ENSGGOT00000000946	ENSCPOT00000000345	ENSDORT00000014596	ENSMMUT00000031295	ENSCJAT00000008304	ENSMUST00000022954	ENSMICT00000009494	ENSPPYT00000022048	ENSOPRT00000000402	ENSOCUT00000000742	ENSRNOT00000012753	ENSSTOT00000008706	ENSTBET00000004774	ENSTSYT00000004117
ENST00000447532	ENSOGAT00000016130	ENSPTRT00000006974	ENSBTAT00000024228	ENSNLET00000003141	ENSGGOT00000004094	ENSCPOT00000027400	ENSDORT00000000133	ENSMMUT00000031467	ENSCJAT00000037841	ENSMUST00000099694	ENSMICT00000013758	ENSPPYT00000003813	ENSOPRT00000008320	ENSOCUT00000001676	ENSRNOT00000028094	ENSSTOT00000007645	ENSTBET00000010238	ENSTSYT00000001812
ENST00000523221	ENSOGAT00000011481	ENSPTRT00000046270	ENSBTAT00000006322	ENSNLET00000002507	ENSGGOT00000033511	ENSCPOT00000009338	ENSDORT00000007698	ENSMMUT00000032243	ENSCJAT00000014174	ENSMUST00000137266	ENSMICT00000013344	ENSPPYT00000014022	ENSOPRT00000002939	ENSOCUT00000003367	ENSRNOT00000024152	ENSSTOT00000006059	ENSTBET00000015638	ENSTSYT00000001952
ENST00000395743	ENSOGAT00000029129	ENSPTRT00000030100	ENSBTAT00000005549	ENSNLET00000010157	ENSGGOT00000013864	ENSCPOT00000022276	ENSDORT00000016193	ENSMMUT00000003944	ENSCJAT00000039241	ENSMUST00000121044	ENSMICT00000009437	ENSPPYT00000017244	ENSOPRT00000008107	ENSOCUT00000003209	ENSRNOT00000003686	ENSSTOT00000014017	ENSTBET00000013616	ENSTSYT00000001453
ENST00000334166	ENSOGAT00000009027	ENSPTRT00000023225	ENSBTAT00000064259	ENSNLET00000011130	ENSGGOT00000007433	ENSCPOT00000007760	ENSDORT00000006428	ENSMMUT00000002707	ENSCJAT00000061685	ENSMUST00000169232	ENSMICT00000005945	ENSPPYT00000014914	ENSOPRT00000008635	ENSOCUT00000008117	ENSRNOT00000059588	ENSSTOT00000007826	ENSTBET00000003331	ENSTSYT00000005946
ENST00000264977	ENSOGAT00000006815	ENSPTRT00000028809	ENSBTAT00000011215	ENSNLET00000004966	ENSGGOT00000000565	ENSCPOT00000002621	ENSDORT00000008587	ENSMMUT00000001329	ENSCJAT00000005123	ENSMUST00000075941	ENSMICT00000000458	ENSPPYT00000016428	ENSOPRT00000010943	ENSOCUT00000001838	ENSRNOT00000010320	ENSSTOT00000014779	ENSTBET00000014182	ENSTSYT00000013221
ENST00000398167	ENSOGAT00000015966	ENSPTRT00000000969	ENSBTAT00000027374	ENSNLET00000004088	ENSGGOT00000014944	ENSCPOT00000004972	ENSDORT00000004407	ENSMMUT00000008799	ENSCJAT00000002127	ENSMUST00000106068	ENSMICT00000012458	ENSPPYT00000001878	ENSOPRT00000005039	ENSOCUT00000009990	ENSRNOT00000065598	ENSSTOT00000016046	ENSTBET00000015521	ENSTSYT00000001436
ENST00000307602	ENSOGAT00000010917	ENSPTRT00000037459	ENSBTAT00000010044	ENSNLET00000016544	ENSGGOT00000004936	ENSCPOT00000000251	ENSDORT00000001526	ENSMMUT00000039038	ENSCJAT00000006707	ENSMUST00000037182	ENSMICT00000001084	ENSPPYT00000021651	ENSOPRT00000009760	ENSOCUT00000001399	ENSRNOT00000019356	ENSSTOT00000011616	ENSTBET00000010000	ENSTSYT00000003168
ENST00000372136	ENSOGAT00000008872	ENSPTRT00000049875	ENSBTAT00000006841	ENSNLET00000016478	ENSGGOT00000002149	ENSCPOT00000004464	ENSDORT00000012260	ENSMMUT00000029765	ENSCJAT00000061380	ENSMUST00000102877	ENSMICT00000010626	ENSPPYT00000023018	ENSOPRT00000000117	ENSOCUT00000014252	ENSRNOT00000017004	ENSSTOT00000003455	ENSTBET00000014131	ENSTSYT00000001838
ENST00000359322	ENSOGAT00000017206	ENSPTRT00000004953	ENSBTAT00000028159	ENSNLET00000021303	ENSGGOT00000024189	ENSCPOT00000003435	ENSDORT00000010297	ENSMMUT00000042842	ENSCJAT00000003035	ENSMUST00000090469	ENSMICT00000007992	ENSPPYT00000002801	ENSOPRT00000001191	ENSOCUT00000012553	ENSRNOT00000011554	ENSSTOT00000002363	ENSTBET00000005173	ENSTSYT00000002098
ENST00000320963	ENSOGAT00000001998	ENSPTRT00000065774	ENSBTAT00000020950	ENSNLET00000010555	ENSGGOT00000032958	ENSCPOT00000014511	ENSDORT00000013690	ENSMMUT00000011481	ENSCJAT00000021516	ENSMUST00000114276	ENSMICT00000003053	ENSPPYT00000007885	ENSOPRT00000016852	ENSOCUT00000003253	ENSRNOT00000021817	ENSSTOT00000005798	ENSTBET00000013516	ENSTSYT00000008038
ENST00000437989	ENSOGAT00000006792	ENSPTRT00000012720	ENSBTAT00000002823	ENSNLET00000014343	ENSGGOT00000013207	ENSCPOT00000002606	ENSDORT00000009106	ENSMMUT00000009561	ENSCJAT00000041295	ENSMUST00000110918	ENSMICT00000014636	ENSPPYT00000007469	ENSOPRT00000000239	ENSOCUT00000016094	ENSRNOT00000006177	ENSSTOT00000004314	ENSTBET00000014933	ENSTSYT00000002094
ENST00000328457	ENSOGAT00000002607	ENSPTRT00000031232	ENSBTAT00000014357	ENSNLET00000021208	ENSGGOT00000007562	ENSCPOT00000007165	ENSDORT00000006478	ENSMMUT00000025289	ENSCJAT00000014172	ENSMUST00000061925	ENSMICT00000014434	ENSPPYT00000017937	ENSOPRT00000003849	ENSOCUT00000002874	ENSRNOT00000019524	ENSSTOT00000008256	ENSTBET00000009032	ENSTSYT00000010370
ENST00000350221	ENSOGAT00000025164	ENSPTRT00000012738	ENSBTAT00000015941	ENSNLET00000002759	ENSGGOT00000008260	ENSCPOT00000014929	ENSDORT00000013010	ENSMMUT00000008437	ENSCJAT00000041138	ENSMUST00000028820	ENSMICT00000003184	ENSPPYT00000007478	ENSOPRT00000014356	ENSOCUT00000003805	ENSRNOT00000007808	ENSSTOT00000012078	ENSTBET00000012876	ENSTSYT00000002582
ENST00000366621	ENSOGAT00000002274	ENSPTRT00000003878	ENSBTAT00000005929	ENSNLET00000003156	ENSGGOT00000011005	ENSCPOT00000001080	ENSDORT00000005648	ENSMMUT00000031549	ENSCJAT00000035621	ENSMUST00000046765	ENSMICT00000015514	ENSPPYT00000000114	ENSOPRT00000013765	ENSOCUT00000016002	ENSRNOT00000027058	ENSSTOT00000001207	ENSTBET00000004108	ENSTSYT00000006906
ENST00000314475	ENSOGAT00000032434	ENSPTRT00000055169	ENSBTAT00000040031	ENSNLET00000009454	ENSGGOT00000009941	ENSCPOT00000010571	ENSDORT00000003565	ENSMMUT00000017163	ENSCJAT00000027592	ENSMUST00000061833	ENSMICT00000011143	ENSPPYT00000004729	ENSOPRT00000005387	ENSOCUT00000005190	ENSRNOT00000038647	ENSSTOT00000015851	ENSTBET00000007018	ENSTSYT00000001228
ENST00000393727	ENSOGAT00000008638	ENSPTRT00000036217	ENSBTAT00000012245	ENSNLET00000011849	ENSGGOT00000000531	ENSCPOT00000008201	ENSDORT00000002470	ENSMMUT00000004857	ENSCJAT00000024743	ENSMUST00000030878	ENSMICT00000008391	ENSPPYT00000020836	ENSOPRT00000005894	ENSOCUT00000010228	ENSRNOT00000015733	ENSSTOT00000011752	ENSTBET00000014208	ENSTSYT00000000667
ENST00000182096	ENSOGAT00000012164	ENSPTRT00000028322	ENSBTAT00000032404	ENSNLET00000002577	ENSGGOT00000026834	ENSCPOT00000011176	ENSDORT00000006619	ENSMMUT00000025113	ENSCJAT00000016616	ENSMUST00000044604	ENSMICT00000002998	ENSPPYT00000015836	ENSOPRT00000015738	ENSOCUT00000012879	ENSRNOT00000002292	ENSSTOT00000005222	ENSTBET00000004079	ENSTSYT00000003874
ENST00000371269	ENSOGAT00000003161	ENSPTRT00000001481	ENSBTAT00000006153	ENSNLET00000018621	ENSGGOT00000010905	ENSCPOT00000015784	ENSDORT00000006691	ENSMMUT00000030362	ENSCJAT00000030473	ENSMUST00000047973	ENSMICT00000007630	ENSPPYT00000001573	ENSOPRT00000016129	ENSOCUT00000005192	ENSRNOT00000009402	ENSSTOT00000004061	ENSTBET00000007273	ENSTSYT00000007121
ENST00000228534	ENSOGAT00000024448	ENSPTRT00000009369	ENSBTAT00000005745	ENSNLET00000022317	ENSGGOT00000001671	ENSCPOT00000015072	ENSDORT00000012955	ENSMMUT00000046362	ENSCJAT00000021700	ENSMUST00000026449	ENSMICT00000000088	ENSPPYT00000005515	ENSOPRT00000001492	ENSOCUT00000007231	ENSRNOT00000004363	ENSSTOT00000015982	ENSTBET00000003885	ENSTSYT00000002170
ENST00000361874	ENSOGAT00000003519	ENSPTRT00000022474	ENSBTAT00000006430	ENSNLET00000000745	ENSGGOT00000023209	ENSCPOT00000012834	ENSDORT00000001938	ENSMMUT00000021902	ENSCJAT00000034562	ENSMUST00000113919	ENSMICT00000006163	ENSPPYT00000014249	ENSOPRT00000013890	ENSOCUT00000015263	ENSRNOT00000013513	ENSSTOT00000000997	ENSTBET00000017222	ENSTSYT00000013093
ENST00000367028	ENSOGAT00000034452	ENSPTRT00000003551	ENSBTAT00000020078	ENSNLET00000002194	ENSGGOT00000006214	ENSCPOT00000005656	ENSDORT00000008881	ENSMMUT00000001442	ENSCJAT00000029105	ENSMUST00000016338	ENSMICT00000001214	ENSPPYT00000000281	ENSOPRT00000014245	ENSOCUT00000004024	ENSRNOT00000007870	ENSSTOT00000016544	ENSTBET00000003789	ENSTSYT00000006707
ENST00000232607	ENSOGAT00000005427	ENSPTRT00000028600	ENSBTAT00000018248	ENSNLET00000000840	ENSGGOT00000027508	ENSCPOT00000008060	ENSDORT00000004039	ENSMMUT00000046818	ENSCJAT00000012741	ENSMUST00000023510	ENSMICT00000001316	ENSPPYT00000015664	ENSOPRT00000010433	ENSOCUT00000000412	ENSRNOT00000002450	ENSSTOT00000005680	ENSTBET00000000753	ENSTSYT00000012385
ENST00000301788	ENSOGAT00000027589	ENSPTRT00000007054	ENSBTAT00000012481	ENSNLET00000005205	ENSGGOT00000011782	ENSCPOT00000001165	ENSDORT00000015696	ENSMMUT00000020163	ENSCJAT00000036662	ENSMUST00000096261	ENSMICT00000009960	ENSPPYT00000003765	ENSOPRT00000014763	ENSOCUT00000009484	ENSRNOT00000026313	ENSSTOT00000004386	ENSTBET00000015759	ENSTSYT00000011908
ENST00000360635	ENSOGAT00000001680	ENSPTRT00000021692	ENSBTAT00000017362	ENSNLET00000016989	ENSGGOT00000001766	ENSCPOT00000010387	ENSDORT00000014262	ENSMMUT00000025334	ENSCJAT00000012173	ENSMUST00000076260	ENSMICT00000002356	ENSPPYT00000014715	ENSOPRT00000008882	ENSOCUT00000003724	ENSRNOT00000009401	ENSSTOT00000004458	ENSTBET00000001612	ENSTSYT00000014536
ENST00000375120	ENSOGAT00000002449	ENSPTRT00000000555	ENSBTAT00000031588	ENSNLET00000010067	ENSGGOT00000003524	ENSCPOT00000015216	ENSDORT00000010171	ENSMMUT00000015046	ENSCJAT00000012912	ENSMUST00000097830	ENSMICT00000014382	ENSPPYT00000002127	ENSOPRT00000007129	ENSOCUT00000006571	ENSRNOT00000023143	ENSSTOT00000007921	ENSTBET00000006727	ENSTSYT00000010863
ENST00000297145	ENSOGAT00000004830	ENSPTRT00000049470	ENSBTAT00000010002	ENSNLET00000014504	ENSGGOT00000031853	ENSCPOT00000014274	ENSDORT00000015222	ENSMMUT00000021800	ENSCJAT00000033066	ENSMUST00000045235	ENSMICT00000000004	ENSPPYT00000020882	ENSOPRT00000016907	ENSOCUT00000010791	ENSRNOT00000009752	ENSSTOT00000002738	ENSTBET00000003905	ENSTSYT00000012644
ENST00000439068	ENSOGAT00000004199	ENSPTRT00000036307	ENSBTAT00000013956	ENSNLET00000014464	ENSGGOT00000016221	ENSCPOT00000004848	ENSDORT00000014428	ENSMMUT00000041913	ENSCJAT00000032980	ENSMUST00000095760	ENSMICT00000001374	ENSPPYT00000020879	ENSOPRT00000008919	ENSOCUT00000000894	ENSRNOT00000059823	ENSSTOT00000010660	ENSTBET00000016273	ENSTSYT00000012835
ENST00000285737	ENSOGAT00000000676	ENSPTRT00000014924	ENSBTAT00000014427	ENSNLET00000000800	ENSGGOT00000006593	ENSCPOT00000003903	ENSDORT00000008241	ENSMMUT00000010345	ENSCJAT00000024455	ENSMUST00000034141	ENSMICT00000014748	ENSPPYT00000008616	ENSOPRT00000007923	ENSOCUT00000007287	ENSRNOT00000020770	ENSSTOT00000008788	ENSTBET00000010506	ENSTSYT00000001399
ENST00000367998	ENSOGAT00000010059	ENSPTRT00000002876	ENSBTAT00000057494	ENSNLET00000018010	ENSGGOT00000007541	ENSCPOT00000015474	ENSDORT00000001695	ENSMMUT00000031844	ENSCJAT00000010023	ENSMUST00000064272	ENSMICT00000007258	ENSPPYT00000000736	ENSOPRT00000003302	ENSOCUT00000002405	ENSRNOT00000004735	ENSSTOT00000006455	ENSTBET00000016911	ENSTSYT00000000536
ENST00000337702	ENSOGAT00000011948	ENSPTRT00000031223	ENSBTAT00000000013	ENSNLET00000020979	ENSGGOT00000026846	ENSCPOT00000009325	ENSDORT00000014180	ENSMMUT00000033497	ENSCJAT00000014510	ENSMUST00000022751	ENSMICT00000012328	ENSPPYT00000017926	ENSOPRT00000003626	ENSOCUT00000000896	ENSRNOT00000017665	ENSSTOT00000008097	ENSTBET00000007116	ENSTSYT00000000915
ENST00000546306	ENSOGAT00000005234	ENSPTRT00000012113	ENSBTAT00000061260	ENSNLET00000021490	ENSGGOT00000003787	ENSCPOT00000013978	ENSDORT00000004005	ENSMMUT00000031123	ENSCJAT00000033470	ENSMUST00000052969	ENSMICT00000010863	ENSPPYT00000007141	ENSOPRT00000012235	ENSOCUT00000012412	ENSRNOT00000005881	ENSSTOT00000005914	ENSTBET00000005672	ENSTSYT00000001552
ENST00000274341	ENSOGAT00000009057	ENSPTRT00000031609	ENSBTAT00000016468	ENSNLET00000017065	ENSGGOT00000024208	ENSCPOT00000011793	ENSDORT00000015895	ENSMMUT00000016129	ENSCJAT00000021729	ENSMUST00000022108	ENSMICT00000005005	ENSPPYT00000018163	ENSOPRT00000016889	ENSOCUT00000002628	ENSRNOT00000042958	ENSSTOT00000003871	ENSTBET00000004050	ENSTSYT00000004172
ENST00000397997	ENSOGAT00000002310	ENSPTRT00000009567	ENSBTAT00000025495	ENSNLET00000004894	ENSGGOT00000005305	ENSCPOT00000002872	ENSDORT00000010321	ENSMMUT00000015130	ENSCJAT00000003355	ENSMUST00000020381	ENSMICT00000011462	ENSPPYT00000005632	ENSOPRT00000003490	ENSOCUT00000017189	ENSRNOT00000007472	ENSSTOT00000014029	ENSTBET00000007024	ENSTSYT00000011445
ENST00000372745	ENSOGAT00000016708	ENSPTRT00000046293	ENSBTAT00000029169	ENSNLET00000021444	ENSGGOT00000011162	ENSCPOT00000005123	ENSDORT00000012888	ENSMMUT00000029296	ENSCJAT00000054859	ENSMUST00000154460	ENSMICT00000011744	ENSPPYT00000002788	ENSOPRT00000006360	ENSOCUT00000009946	ENSRNOT00000016387	ENSSTOT00000012882	ENSTBET00000014714	ENSTSYT00000000135
ENST00000263847	ENSOGAT00000004352	ENSPTRT00000006906	ENSBTAT00000027095	ENSNLET00000016674	ENSGGOT00000011387	ENSCPOT00000005841	ENSDORT00000000514	ENSMMUT00000002466	ENSCJAT00000031479	ENSMUST00000025590	ENSMICT00000004166	ENSPPYT00000003854	ENSOPRT00000007944	ENSOCUT00000002149	ENSRNOT00000065411	ENSSTOT00000013752	ENSTBET00000009314	ENSTSYT00000009859
ENST00000261534	ENSOGAT00000006200	ENSPTRT00000012062	ENSBTAT00000003502	ENSNLET00000021281	ENSGGOT00000006233	ENSCPOT00000007155	ENSDORT00000003506	ENSMMUT00000001552	ENSCJAT00000032598	ENSMUST00000037788	ENSMICT00000017150	ENSPPYT00000007117	ENSOPRT00000013920	ENSOCUT00000005777	ENSRNOT00000016242	ENSSTOT00000002770	ENSTBET00000012179	ENSTSYT00000002820
ENST00000271640	ENSOGAT00000010325	ENSPTRT00000002349	ENSBTAT00000000107	ENSNLET00000012798	ENSGGOT00000016371	ENSCPOT00000001653	ENSDORT00000006829	ENSMMUT00000046077	ENSCJAT00000013764	ENSMUST00000015841	ENSMICT00000000437	ENSPPYT00000001070	ENSOPRT00000015362	ENSOCUT00000012786	ENSRNOT00000028709	ENSSTOT00000006783	ENSTBET00000009315	ENSTSYT00000012647
ENST00000281830	ENSOGAT00000016325	ENSPTRT00000024045	ENSBTAT00000008433	ENSNLET00000012395	ENSGGOT00000017213	ENSCPOT00000013993	ENSDORT00000001670	ENSMMUT00000001560	ENSCJAT00000023906	ENSMUST00000057262	ENSMICT00000003047	ENSPPYT00000015375	ENSOPRT00000005708	ENSOCUT00000005455	ENSRNOT00000020169	ENSSTOT00000010992	ENSTBET00000006522	ENSTSYT00000003132
ENST00000545121	ENSOGAT00000010802	ENSPTRT00000043145	ENSBTAT00000026862	ENSNLET00000004758	ENSGGOT00000009616	ENSCPOT00000011169	ENSDORT00000009332	ENSMMUT00000046412	ENSCJAT00000025637	ENSMUST00000068301	ENSMICT00000011931	ENSPPYT00000001249	ENSOPRT00000009398	ENSOCUT00000015742	ENSRNOT00000036551	ENSSTOT00000011071	ENSTBET00000010595	ENSTSYT00000004994
ENST00000380639	ENSOGAT00000008393	ENSPTRT00000031471	ENSBTAT00000019535	ENSNLET00000005800	ENSGGOT00000010738	ENSCPOT00000006282	ENSDORT00000004921	ENSMMUT00000025165	ENSCJAT00000031834	ENSMUST00000022153	ENSMICT00000004541	ENSPPYT00000018078	ENSOPRT00000007286	ENSOCUT00000007967	ENSRNOT00000034992	ENSSTOT00000006599	ENSTBET00000016559	ENSTSYT00000000561
ENST00000296589	ENSOGAT00000009121	ENSPTRT00000031149	ENSBTAT00000024272	ENSNLET00000018246	ENSGGOT00000004845	ENSCPOT00000009516	ENSDORT00000002486	ENSMMUT00000041877	ENSCJAT00000036552	ENSMUST00000022851	ENSMICT00000005626	ENSPPYT00000017883	ENSOPRT00000011933	ENSOCUT00000004947	ENSRNOT00000025490	ENSSTOT00000002489	ENSTBET00000010879	ENSTSYT00000008601
ENST00000322507	ENSOGAT00000015719	ENSPTRT00000033939	ENSBTAT00000026725	ENSNLET00000006298	ENSGGOT00000032077	ENSCPOT00000014533	ENSDORT00000010156	ENSMMUT00000027056	ENSCJAT00000020003	ENSMUST00000071750	ENSMICT00000015731	ENSPPYT00000019524	ENSOPRT00000004555	ENSOCUT00000015357	ENSRNOT00000043691	ENSSTOT00000002528	ENSTBET00000003214	ENSTSYT00000008947
ENST00000471166	ENSOGAT00000015350	ENSPTRT00000036454	ENSBTAT00000006573	ENSNLET00000016003	ENSGGOT00000008380	ENSCPOT00000012050	ENSDORT00000002434	ENSMMUT00000006124	ENSCJAT00000035445	ENSMUST00000115251	ENSMICT00000000141	ENSPPYT00000020976	ENSOPRT00000006180	ENSOCUT00000010108	ENSRNOT00000043902	ENSSTOT00000004900	ENSTBET00000007243	ENSTSYT00000003053
ENST00000321599	ENSOGAT00000011809	ENSPTRT00000027945	ENSBTAT00000015405	ENSNLET00000008801	ENSGGOT00000010673	ENSCPOT00000011552	ENSDORT00000007515	ENSMMUT00000020464	ENSCJAT00000008305	ENSMUST00000035214	ENSMICT00000010747	ENSPPYT00000016126	ENSOPRT00000013043	ENSOCUT00000005016	ENSRNOT00000041402	ENSSTOT00000013732	ENSTBET00000007864	ENSTSYT00000006145
ENST00000261772	ENSOGAT00000011608	ENSPTRT00000060139	ENSBTAT00000026393	ENSNLET00000020428	ENSGGOT00000025762	ENSCPOT00000002406	ENSDORT00000004111	ENSMMUT00000030472	ENSCJAT00000028376	ENSMUST00000034441	ENSMICT00000000257	ENSPPYT00000008865	ENSOPRT00000003396	ENSOCUT00000008876	ENSRNOT00000025052	ENSSTOT00000006881	ENSTBET00000017163	ENSTSYT00000010991
ENST00000432060	ENSOGAT00000007906	ENSPTRT00000023361	ENSBTAT00000063743	ENSNLET00000005980	ENSGGOT00000013602	ENSCPOT00000006009	ENSDORT00000003025	ENSMMUT00000019419	ENSCJAT00000058962	ENSMUST00000063690	ENSMICT00000017337	ENSPPYT00000014994	ENSOPRT00000005216	ENSOCUT00000005663	ENSRNOT00000008921	ENSSTOT00000009894	ENSTBET00000008163	ENSTSYT00000004865
ENST00000375129	ENSOGAT00000027406	ENSPTRT00000056301	ENSBTAT00000056768	ENSNLET00000007820	ENSGGOT00000011111	ENSCPOT00000003947	ENSDORT00000013339	ENSMMUT00000045070	ENSCJAT00000042334	ENSMUST00000099986	ENSMICT00000009880	ENSPPYT00000015085	ENSOPRT00000007581	ENSOCUT00000005492	ENSRNOT00000058093	ENSSTOT00000006158	ENSTBET00000007029	ENSTSYT00000005287
ENST00000450536	ENSOGAT00000016830	ENSPTRT00000034449	ENSBTAT00000016319	ENSNLET00000019170	ENSGGOT00000009133	ENSCPOT00000000741	ENSDORT00000000650	ENSMMUT00000000335	ENSCJAT00000037449	ENSMUST00000126390	ENSMICT00000008985	ENSPPYT00000019843	ENSOPRT00000009968	ENSOCUT00000016848	ENSRNOT00000016818	ENSSTOT00000000923	ENSTBET00000006447	ENSTSYT00000003290
ENST00000533102	ENSOGAT00000009356	ENSPTRT00000008076	ENSBTAT00000017194	ENSNLET00000009410	ENSGGOT00000000960	ENSCPOT00000006337	ENSDORT00000007244	ENSMMUT00000007727	ENSCJAT00000026042	ENSMUST00000002100	ENSMICT00000015318	ENSPPYT00000004687	ENSOPRT00000011357	ENSOCUT00000010153	ENSRNOT00000067558	ENSSTOT00000014433	ENSTBET00000013364	ENSTSYT00000006397
ENST00000323011	ENSOGAT00000007638	ENSPTRT00000018516	ENSBTAT00000031089	ENSNLET00000011530	ENSGGOT00000011224	ENSCPOT00000001768	ENSDORT00000016386	ENSMMUT00000000833	ENSCJAT00000005617	ENSMUST00000172005	ENSMICT00000000924	ENSPPYT00000010765	ENSOPRT00000011880	ENSOCUT00000015601	ENSRNOT00000041413	ENSSTOT00000008888	ENSTBET00000015919	ENSTSYT00000003997
ENST00000309934	ENSOGAT00000012489	ENSPTRT00000028581	ENSBTAT00000019691	ENSNLET00000000429	ENSGGOT00000002474	ENSCPOT00000009259	ENSDORT00000000316	ENSMMUT00000010788	ENSCJAT00000015042	ENSMUST00000043521	ENSMICT00000008149	ENSPPYT00000015673	ENSOPRT00000014003	ENSOCUT00000001902	ENSRNOT00000063795	ENSSTOT00000000042	ENSTBET00000013692	ENSTSYT00000012049
ENST00000374647	ENSOGAT00000009072	ENSPTRT00000039255	ENSBTAT00000006570	ENSNLET00000022157	ENSGGOT00000027576	ENSCPOT00000005970	ENSDORT00000008616	ENSMMUT00000027191	ENSCJAT00000033111	ENSMUST00000030140	ENSMICT00000002066	ENSPPYT00000022718	ENSOPRT00000013558	ENSOCUT00000001874	ENSRNOT00000022836	ENSSTOT00000006457	ENSTBET00000004174	ENSTSYT00000009232
ENST00000368078	ENSOGAT00000025479	ENSPTRT00000002824	ENSBTAT00000026932	ENSNLET00000017175	ENSGGOT00000014693	ENSCPOT00000015060	ENSDORT00000013062	ENSMMUT00000025069	ENSCJAT00000011668	ENSMUST00000003554	ENSMICT00000007989	ENSPPYT00000000776	ENSOPRT00000011941	ENSOCUT00000009959	ENSRNOT00000009344	ENSSTOT00000007552	ENSTBET00000004823	ENSTSYT00000003241
ENST00000272438	ENSOGAT00000015336	ENSPTRT00000022398	ENSBTAT00000047005	ENSNLET00000013102	ENSGGOT00000009604	ENSCPOT00000023891	ENSDORT00000004535	ENSMMUT00000002956	ENSCJAT00000038034	ENSMUST00000014892	ENSMICT00000007049	ENSPPYT00000014292	ENSOPRT00000004240	ENSOCUT00000008896	ENSRNOT00000018569	ENSSTOT00000006450	ENSTBET00000005455	ENSTSYT00000005190
ENST00000370328	ENSOGAT00000002942	ENSPTRT00000041753	ENSBTAT00000065531	ENSNLET00000017571	ENSGGOT00000001385	ENSCPOT00000020449	ENSDORT00000005058	ENSMMUT00000015306	ENSCJAT00000061016	ENSMUST00000064780	ENSMICT00000004134	ENSPPYT00000024287	ENSOPRT00000011411	ENSOCUT00000009667	ENSRNOT00000039949	ENSSTOT00000001126	ENSTBET00000006465	ENSTSYT00000013935
ENST00000353214	ENSOGAT00000028908	ENSPTRT00000039515	ENSBTAT00000020318	ENSNLET00000022073	ENSGGOT00000004134	ENSCPOT00000014849	ENSDORT00000012125	ENSMMUT00000029023	ENSCJAT00000013132	ENSMUST00000090993	ENSMICT00000002923	ENSPPYT00000022875	ENSOPRT00000000406	ENSOCUT00000002997	ENSRNOT00000019405	ENSSTOT00000007896	ENSTBET00000008394	ENSTSYT00000009748
ENST00000403359	ENSOGAT00000011990	ENSPTRT00000022151	ENSBTAT00000001868	ENSNLET00000011059	ENSGGOT00000011655	ENSCPOT00000000939	ENSDORT00000012562	ENSMMUT00000032157	ENSCJAT00000021472	ENSMUST00000005504	ENSMICT00000010586	ENSPPYT00000014431	ENSOPRT00000004882	ENSOCUT00000014636	ENSRNOT00000021998	ENSSTOT00000004775	ENSTBET00000017373	ENSTSYT00000014512
ENST00000258484	ENSOGAT00000009022	ENSPTRT00000023218	ENSBTAT00000024730	ENSNLET00000011682	ENSGGOT00000011344	ENSCPOT00000008579	ENSDORT00000001354	ENSMMUT00000032599	ENSCJAT00000024566	ENSMUST00000092123	ENSMICT00000016657	ENSPPYT00000014912	ENSOPRT00000010397	ENSOCUT00000012714	ENSRNOT00000051953	ENSSTOT00000010470	ENSTBET00000009505	ENSTSYT00000000438
ENST00000251089	ENSOGAT00000000180	ENSPTRT00000012052	ENSBTAT00000025915	ENSNLET00000021194	ENSGGOT00000017099	ENSCPOT00000011581	ENSDORT00000007719	ENSMMUT00000026080	ENSCJAT00000040736	ENSMUST00000021682	ENSMICT00000003637	ENSPPYT00000007109	ENSOPRT00000013801	ENSOCUT00000009534	ENSRNOT00000014635	ENSSTOT00000011128	ENSTBET00000011010	ENSTSYT00000011919
ENST00000369764	ENSOGAT00000008725	ENSPTRT00000005552	ENSBTAT00000019995	ENSNLET00000019479	ENSGGOT00000009017	ENSCPOT00000006338	ENSDORT00000005994	ENSMMUT00000021485	ENSCJAT00000035911	ENSMUST00000049369	ENSMICT00000012553	ENSPPYT00000003162	ENSOPRT00000010701	ENSOCUT00000003029	ENSRNOT00000027614	ENSSTOT00000011907	ENSTBET00000000700	ENSTSYT00000001336
ENST00000372389	ENSOGAT00000016322	ENSPTRT00000033663	ENSBTAT00000025246	ENSNLET00000005174	ENSGGOT00000010946	ENSCPOT00000010001	ENSDORT00000000429	ENSMMUT00000040567	ENSCJAT00000010818	ENSMUST00000164448	ENSMICT00000005024	ENSPPYT00000019359	ENSOPRT00000000144	ENSOCUT00000005505	ENSRNOT00000025787	ENSSTOT00000010633	ENSTBET00000002237	ENSTSYT00000011913
ENST00000377041	ENSOGAT00000012920	ENSPTRT00000038868	ENSBTAT00000008957	ENSNLET00000001264	ENSGGOT00000009742	ENSCPOT00000005392	ENSDORT00000013122	ENSMMUT00000026541	ENSCJAT00000002707	ENSMUST00000052556	ENSMICT00000003029	ENSPPYT00000022488	ENSOPRT00000007062	ENSOCUT00000000819	ENSRNOT00000044486	ENSSTOT00000004662	ENSTBET00000015495	ENSTSYT00000007869
ENST00000441535	ENSOGAT00000016481	ENSPTRT00000003028	ENSBTAT00000003873	ENSNLET00000019322	ENSGGOT00000008928	ENSCPOT00000014128	ENSDORT00000004731	ENSMMUT00000027724	ENSCJAT00000019124	ENSMUST00000111510	ENSMICT00000017108	ENSPPYT00000000624	ENSOPRT00000011045	ENSOCUT00000005178	ENSRNOT00000004762	ENSSTOT00000003860	ENSTBET00000014857	ENSTSYT00000014472
ENST00000372904	ENSOGAT00000016089	ENSPTRT00000025166	ENSBTAT00000025123	ENSNLET00000008427	ENSGGOT00000004813	ENSCPOT00000002037	ENSDORT00000004955	ENSMMUT00000019605	ENSCJAT00000034573	ENSMUST00000109408	ENSMICT00000008260	ENSPPYT00000012804	ENSOPRT00000014311	ENSOCUT00000004258	ENSRNOT00000012606	ENSSTOT00000015678	ENSTBET00000005143	ENSTSYT00000007585
ENST00000369452	ENSOGAT00000012590	ENSPTRT00000005608	ENSBTAT00000010138	ENSNLET00000019903	ENSGGOT00000008504	ENSCPOT00000010984	ENSDORT00000002618	ENSMMUT00000024187	ENSCJAT00000037024	ENSMUST00000169861	ENSMICT00000008779	ENSPPYT00000003192	ENSOPRT00000014598	ENSOCUT00000014349	ENSRNOT00000020580	ENSSTOT00000015024	ENSTBET00000017566	ENSTSYT00000010094
ENST00000367098	ENSOGAT00000004083	ENSPTRT00000003492	ENSBTAT00000003574	ENSNLET00000001030	ENSGGOT00000013318	ENSCPOT00000005092	ENSDORT00000008856	ENSMMUT00000033157	ENSCJAT00000036305	ENSMUST00000027673	ENSMICT00000008363	ENSPPYT00000000314	ENSOPRT00000011337	ENSOCUT00000014282	ENSRNOT00000065296	ENSSTOT00000006436	ENSTBET00000012959	ENSTSYT00000008788
ENST00000388923	ENSOGAT00000006796	ENSPTRT00000057031	ENSBTAT00000023768	ENSNLET00000016483	ENSGGOT00000011646	ENSCPOT00000010584	ENSDORT00000015185	ENSMMUT00000031590	ENSCJAT00000001184	ENSMUST00000095129	ENSMICT00000016379	ENSPPYT00000019582	ENSOPRT00000012163	ENSOCUT00000017094	ENSRNOT00000012164	ENSSTOT00000007148	ENSTBET00000000816	ENSTSYT00000002167
ENST00000393751	ENSOGAT00000007056	ENSPTRT00000067517	ENSBTAT00000006504	ENSNLET00000008335	ENSGGOT00000007837	ENSCPOT00000008396	ENSDORT00000016695	ENSMMUT00000002648	ENSCJAT00000009065	ENSMUST00000034052	ENSMICT00000013531	ENSPPYT00000017651	ENSOPRT00000001641	ENSOCUT00000003738	ENSRNOT00000059724	ENSSTOT00000009521	ENSTBET00000000659	ENSTSYT00000002816
ENST00000515098	ENSOGAT00000033461	ENSPTRT00000032530	ENSBTAT00000008362	ENSNLET00000020067	ENSGGOT00000000014	ENSCPOT00000003463	ENSDORT00000003311	ENSMMUT00000026372	ENSCJAT00000023789	ENSMUST00000001081	ENSMICT00000005370	ENSPPYT00000018739	ENSOPRT00000007890	ENSOCUT00000003203	ENSRNOT00000036789	ENSSTOT00000003215	ENSTBET00000012521	ENSTSYT00000008075
ENST00000451373	ENSOGAT00000001352	ENSPTRT00000015697	ENSBTAT00000063495	ENSNLET00000000651	ENSGGOT00000015559	ENSCPOT00000004351	ENSDORT00000007546	ENSMMUT00000032886	ENSCJAT00000042687	ENSMUST00000094014	ENSMICT00000009339	ENSPPYT00000009099	ENSOPRT00000002442	ENSOCUT00000002201	ENSRNOT00000010168	ENSSTOT00000016425	ENSTBET00000014870	ENSTSYT00000005989
ENST00000541240	ENSOGAT00000006296	ENSPTRT00000015061	ENSBTAT00000032447	ENSNLET00000004167	ENSGGOT00000007182	ENSCPOT00000006741	ENSDORT00000013104	ENSMMUT00000022031	ENSCJAT00000022923	ENSMUST00000034240	ENSMICT00000006382	ENSPPYT00000008702	ENSOPRT00000008266	ENSOCUT00000012069	ENSRNOT00000061310	ENSSTOT00000007476	ENSTBET00000000366	ENSTSYT00000011318
ENST00000268164	ENSOGAT00000016825	ENSPTRT00000013779	ENSBTAT00000023784	ENSNLET00000015727	ENSGGOT00000012573	ENSCPOT00000022091	ENSDORT00000002034	ENSMMUT00000019394	ENSCJAT00000040110	ENSMUST00000026896	ENSMICT00000012123	ENSPPYT00000008023	ENSOPRT00000015648	ENSOCUT00000002902	ENSRNOT00000016427	ENSSTOT00000012528	ENSTBET00000002641	ENSTSYT00000009841
ENST00000555238	ENSOGAT00000009345	ENSPTRT00000044543	ENSBTAT00000052258	ENSNLET00000003883	ENSGGOT00000002153	ENSCPOT00000002450	ENSDORT00000007325	ENSMMUT00000028416	ENSCJAT00000027880	ENSMUST00000129335	ENSMICT00000017925	ENSPPYT00000007053	ENSOPRT00000002061	ENSOCUT00000010703	ENSRNOT00000049481	ENSSTOT00000003897	ENSTBET00000006924	ENSTSYT00000010351
ENST00000291900	ENSOGAT00000005002	ENSPTRT00000039676	ENSBTAT00000013468	ENSNLET00000022451	ENSGGOT00000012419	ENSCPOT00000009426	ENSDORT00000013503	ENSMMUT00000015203	ENSCJAT00000039677	ENSMUST00000113677	ENSMICT00000017646	ENSPPYT00000022943	ENSOPRT00000010708	ENSOCUT00000008974	ENSRNOT00000035002	ENSSTOT00000009406	ENSTBET00000014739	ENSTSYT00000013282
ENST00000306125	ENSOGAT00000003958	ENSPTRT00000027910	ENSBTAT00000025191	ENSNLET00000008646	ENSGGOT00000014942	ENSCPOT00000010057	ENSDORT00000007392	ENSMMUT00000004172	ENSCJAT00000006819	ENSMUST00000006838	ENSMICT00000007887	ENSPPYT00000016147	ENSOPRT00000006254	ENSOCUT00000005088	ENSRNOT00000064518	ENSSTOT00000001837	ENSTBET00000006140	ENSTSYT00000008500
ENST00000359530	ENSOGAT00000008890	ENSPTRT00000037519	ENSBTAT00000004480	ENSNLET00000001172	ENSGGOT00000000521	ENSCPOT00000012290	ENSDORT00000007689	ENSMMUT00000005770	ENSCJAT00000028187	ENSMUST00000044369	ENSMICT00000015656	ENSPPYT00000021678	ENSOPRT00000000895	ENSOCUT00000016296	ENSRNOT00000043993	ENSSTOT00000015286	ENSTBET00000014889	ENSTSYT00000013845
ENST00000513702	ENSOGAT00000000499	ENSPTRT00000029859	ENSBTAT00000061500	ENSNLET00000021987	ENSGGOT00000009183	ENSCPOT00000010142	ENSDORT00000011299	ENSMMUT00000042305	ENSCJAT00000037748	ENSMUST00000161369	ENSMICT00000010058	ENSPPYT00000017085	ENSOPRT00000013438	ENSOCUT00000017130	ENSRNOT00000003128	ENSSTOT00000005356	ENSTBET00000005712	ENSTSYT00000001319
ENST00000272732	ENSOGAT00000016573	ENSPTRT00000023444	ENSBTAT00000017239	ENSNLET00000007485	ENSGGOT00000041050	ENSCPOT00000010375	ENSDORT00000013677	ENSMMUT00000032378	ENSCJAT00000015475	ENSMUST00000090811	ENSMICT00000000366	ENSPPYT00000015042	ENSOPRT00000010503	ENSOCUT00000011498	ENSRNOT00000025255	ENSSTOT00000001233	ENSTBET00000007450	ENSTSYT00000012365
ENST00000415724	ENSOGAT00000014121	ENSPTRT00000010498	ENSBTAT00000008119	ENSNLET00000016887	ENSGGOT00000028644	ENSCPOT00000011595	ENSDORT00000003892	ENSMMUT00000010308	ENSCJAT00000036687	ENSMUST00000089473	ENSMICT00000003725	ENSPPYT00000006158	ENSOPRT00000011852	ENSOCUT00000001446	ENSRNOT00000063962	ENSSTOT00000005778	ENSTBET00000005972	ENSTSYT00000003043
ENST00000403721	ENSOGAT00000004309	ENSPTRT00000022208	ENSBTAT00000020573	ENSNLET00000011794	ENSGGOT00000007722	ENSCPOT00000005886	ENSDORT00000006292	ENSMMUT00000043541	ENSCJAT00000019492	ENSMUST00000093239	ENSMICT00000017441	ENSPPYT00000014401	ENSOPRT00000010485	ENSOCUT00000003617	ENSRNOT00000005834	ENSSTOT00000008172	ENSTBET00000004718	ENSTSYT00000000848
ENST00000248933	ENSOGAT00000010574	ENSPTRT00000047792	ENSBTAT00000008629	ENSNLET00000009849	ENSGGOT00000005001	ENSCPOT00000022051	ENSDORT00000007331	ENSMMUT00000022579	ENSCJAT00000005952	ENSMUST00000075387	ENSMICT00000013658	ENSPPYT00000013538	ENSOPRT00000013651	ENSOCUT00000011447	ENSRNOT00000032193	ENSSTOT00000013460	ENSTBET00000005442	ENSTSYT00000011897
ENST00000305815	ENSOGAT00000000113	ENSPTRT00000066112	ENSBTAT00000061361	ENSNLET00000005403	ENSGGOT00000001278	ENSCPOT00000009268	ENSDORT00000011261	ENSMMUT00000047005	ENSCJAT00000037687	ENSMUST00000159945	ENSMICT00000002666	ENSPPYT00000015756	ENSOPRT00000007444	ENSOCUT00000009943	ENSRNOT00000040908	ENSSTOT00000001759	ENSTBET00000003798	ENSTSYT00000008175
ENST00000356151	ENSOGAT00000004287	ENSPTRT00000046700	ENSBTAT00000030715	ENSNLET00000009334	ENSGGOT00000015078	ENSCPOT00000006775	ENSDORT00000009687	ENSMMUT00000046495	ENSCJAT00000038754	ENSMUST00000036682	ENSMICT00000014560	ENSPPYT00000016008	ENSOPRT00000013369	ENSOCUT00000000833	ENSRNOT00000044321	ENSSTOT00000015401	ENSTBET00000011363	ENSTSYT00000008155
ENST00000393695	ENSOGAT00000000961	ENSPTRT00000046165	ENSBTAT00000024553	ENSNLET00000022005	ENSGGOT00000017031	ENSCPOT00000008938	ENSDORT00000011934	ENSMMUT00000010249	ENSCJAT00000029813	ENSMUST00000170458	ENSMICT00000014842	ENSPPYT00000004336	ENSOPRT00000014347	ENSOCUT00000012344	ENSRNOT00000055249	ENSSTOT00000010847	ENSTBET00000016501	ENSTSYT00000013705
ENST00000447346	ENSOGAT00000007603	ENSPTRT00000031319	ENSBTAT00000005692	ENSNLET00000002606	ENSGGOT00000003003	ENSCPOT00000006542	ENSDORT00000001447	ENSMMUT00000024463	ENSCJAT00000003923	ENSMUST00000091226	ENSMICT00000011562	ENSPPYT00000017992	ENSOPRT00000008109	ENSOCUT00000007833	ENSRNOT00000041452	ENSSTOT00000004985	ENSTBET00000004082	ENSTSYT00000012109
ENST00000368662	ENSOGAT00000015682	ENSPTRT00000034214	ENSBTAT00000057035	ENSNLET00000016567	ENSGGOT00000004924	ENSCPOT00000004880	ENSDORT00000012028	ENSMMUT00000017635	ENSCJAT00000009367	ENSMUST00000019991	ENSMICT00000008741	ENSPPYT00000019704	ENSOPRT00000000301	ENSOCUT00000009645	ENSRNOT00000000735	ENSSTOT00000005278	ENSTBET00000015749	ENSTSYT00000003029
ENST00000310447	ENSOGAT00000013580	ENSPTRT00000036681	ENSBTAT00000024166	ENSNLET00000004042	ENSGGOT00000003600	ENSCPOT00000014843	ENSDORT00000002962	ENSMMUT00000017713	ENSCJAT00000018627	ENSMUST00000031895	ENSMICT00000015516	ENSPPYT00000021120	ENSOPRT00000009611	ENSOCUT00000002154	ENSRNOT00000022672	ENSSTOT00000005047	ENSTBET00000008272	ENSTSYT00000012300
ENST00000297265	ENSOGAT00000015009	ENSPTRT00000037719	ENSBTAT00000006291	ENSNLET00000003365	ENSGGOT00000016728	ENSCPOT00000013853	ENSDORT00000002077	ENSMMUT00000003735	ENSCJAT00000000760	ENSMUST00000029049	ENSMICT00000016733	ENSPPYT00000021824	ENSOPRT00000004767	ENSOCUT00000006846	ENSRNOT00000013720	ENSSTOT00000012902	ENSTBET00000002835	ENSTSYT00000013540
ENST00000371752	ENSOGAT00000002134	ENSPTRT00000025352	ENSBTAT00000026859	ENSNLET00000008894	ENSGGOT00000008542	ENSCPOT00000011038	ENSDORT00000014143	ENSMMUT00000033229	ENSCJAT00000031367	ENSMUST00000048988	ENSMICT00000002016	ENSPPYT00000012906	ENSOPRT00000014625	ENSOCUT00000009815	ENSRNOT00000010817	ENSSTOT00000003695	ENSTBET00000005841	ENSTSYT00000002158
ENST00000382085	ENSOGAT00000010557	ENSPTRT00000031152	ENSBTAT00000029075	ENSNLET00000018277	ENSGGOT00000030958	ENSCPOT00000009519	ENSDORT00000002487	ENSMMUT00000018900	ENSCJAT00000036519	ENSMUST00000070877	ENSMICT00000005632	ENSPPYT00000017885	ENSOPRT00000011944	ENSOCUT00000004950	ENSRNOT00000025323	ENSSTOT00000015062	ENSTBET00000010926	ENSTSYT00000008606
ENST00000289269	ENSOGAT00000028272	ENSPTRT00000032064	ENSBTAT00000020497	ENSNLET00000012099	ENSGGOT00000000669	ENSCPOT00000020156	ENSDORT00000006579	ENSMMUT00000020444	ENSCJAT00000031547	ENSMUST00000047479	ENSMICT00000015697	ENSPPYT00000018467	ENSOPRT00000016622	ENSOCUT00000004564	ENSRNOT00000027340	ENSSTOT00000012814	ENSTBET00000006149	ENSTSYT00000006719
ENST00000371610	ENSOGAT00000005490	ENSPTRT00000025380	ENSBTAT00000028786	ENSNLET00000008929	ENSGGOT00000010379	ENSCPOT00000021492	ENSDORT00000007031	ENSMMUT00000025523	ENSCJAT00000001029	ENSMUST00000052125	ENSMICT00000015928	ENSPPYT00000012923	ENSOPRT00000001077	ENSOCUT00000015985	ENSRNOT00000014568	ENSSTOT00000009323	ENSTBET00000002250	ENSTSYT00000012283
ENST00000282588	ENSOGAT00000017186	ENSPTRT00000031274	ENSBTAT00000034583	ENSNLET00000013874	ENSGGOT00000028144	ENSCPOT00000013813	ENSDORT00000016688	ENSMMUT00000002476	ENSCJAT00000002840	ENSMUST00000061673	ENSMICT00000004693	ENSPPYT00000017965	ENSOPRT00000010669	ENSOCUT00000011375	ENSRNOT00000016353	ENSSTOT00000010574	ENSTBET00000005328	ENSTSYT00000005199
ENST00000393925	ENSOGAT00000008902	ENSPTRT00000063774	ENSBTAT00000001599	ENSNLET00000005303	ENSGGOT00000015506	ENSCPOT00000005708	ENSDORT00000011246	ENSMMUT00000000998	ENSCJAT00000039711	ENSMUST00000036355	ENSMICT00000009341	ENSPPYT00000015764	ENSOPRT00000009504	ENSOCUT00000003964	ENSRNOT00000042754	ENSSTOT00000001289	ENSTBET00000016969	ENSTSYT00000005570
ENST00000371263	ENSOGAT00000014646	ENSPTRT00000025448	ENSBTAT00000061470	ENSNLET00000009002	ENSGGOT00000008888	ENSCPOT00000011790	ENSDORT00000003757	ENSMMUT00000000368	ENSCJAT00000037574	ENSMUST00000050442	ENSMICT00000000677	ENSPPYT00000012955	ENSOPRT00000000226	ENSOCUT00000000377	ENSRNOT00000008959	ENSSTOT00000000362	ENSTBET00000008953	ENSTSYT00000003868
ENST00000392796	ENSOGAT00000012348	ENSPTRT00000042520	ENSBTAT00000005476	ENSNLET00000001317	ENSGGOT00000015794	ENSCPOT00000008774	ENSDORT00000005592	ENSMMUT00000008649	ENSCJAT00000029519	ENSMUST00000028368	ENSMICT00000010493	ENSPPYT00000014948	ENSOPRT00000002550	ENSOCUT00000015138	ENSRNOT00000008168	ENSSTOT00000009760	ENSTBET00000002986	ENSTSYT00000001748
ENST00000357650	ENSOGAT00000014973	ENSPTRT00000001894	ENSBTAT00000025323	ENSNLET00000015217	ENSGGOT00000004442	ENSCPOT00000007700	ENSDORT00000013733	ENSMMUT00000005275	ENSCJAT00000006745	ENSMUST00000067485	ENSMICT00000005241	ENSPPYT00000001331	ENSOPRT00000004328	ENSOCUT00000010517	ENSRNOT00000019172	ENSSTOT00000005365	ENSTBET00000014213	ENSTSYT00000005177
ENST00000370565	ENSOGAT00000014151	ENSPTRT00000001743	ENSBTAT00000033512	ENSNLET00000012963	ENSGGOT00000003770	ENSCPOT00000015090	ENSDORT00000009201	ENSMMUT00000001956	ENSCJAT00000014282	ENSMUST00000040465	ENSMICT00000015863	ENSPPYT00000001423	ENSOPRT00000013530	ENSOCUT00000004729	ENSRNOT00000018435	ENSSTOT00000001519	ENSTBET00000004406	ENSTSYT00000010241
ENST00000253339	ENSOGAT00000012511	ENSPTRT00000034519	ENSBTAT00000020062	ENSNLET00000019536	ENSGGOT00000000549	ENSCPOT00000003621	ENSDORT00000007189	ENSMMUT00000013730	ENSCJAT00000017833	ENSMUST00000165952	ENSMICT00000006306	ENSPPYT00000019891	ENSOPRT00000009127	ENSOCUT00000016666	ENSRNOT00000020098	ENSSTOT00000013443	ENSTBET00000015437	ENSTSYT00000004345
ENST00000201979	ENSOGAT00000030205	ENSPTRT00000024804	ENSBTAT00000018843	ENSNLET00000012235	ENSGGOT00000000997	ENSCPOT00000011785	ENSDORT00000013290	ENSMMUT00000007215	ENSCJAT00000040891	ENSMUST00000000369	ENSMICT00000014666	ENSPPYT00000012638	ENSOPRT00000010870	ENSOCUT00000003184	ENSRNOT00000010149	ENSSTOT00000004597	ENSTBET00000010845	ENSTSYT00000007632
ENST00000354475	ENSOGAT00000000998	ENSPTRT00000036550	ENSBTAT00000023798	ENSNLET00000016970	ENSGGOT00000000902	ENSCPOT00000015104	ENSDORT00000000571	ENSMMUT00000003279	ENSCJAT00000024165	ENSMUST00000081214	ENSMICT00000001656	ENSPPYT00000021019	ENSOPRT00000002073	ENSOCUT00000014201	ENSRNOT00000014010	ENSSTOT00000003887	ENSTBET00000006720	ENSTSYT00000009636
ENST00000265748	ENSOGAT00000001790	ENSPTRT00000035268	ENSBTAT00000046371	ENSNLET00000002786	ENSGGOT00000001727	ENSCPOT00000008125	ENSDORT00000016592	ENSMMUT00000031332	ENSCJAT00000024131	ENSMUST00000040912	ENSMICT00000008945	ENSPPYT00000020544	ENSOPRT00000008909	ENSOCUT00000005162	ENSRNOT00000019601	ENSSTOT00000006667	ENSTBET00000016199	ENSTSYT00000008122
ENST00000241502	ENSOGAT00000011893	ENSPTRT00000029451	ENSBTAT00000046614	ENSNLET00000019830	ENSGGOT00000011708	ENSCPOT00000011569	ENSDORT00000011686	ENSMMUT00000009266	ENSCJAT00000061136	ENSMUST00000023489	ENSMICT00000010787	ENSPPYT00000016813	ENSOPRT00000008206	ENSOCUT00000012766	ENSRNOT00000050535	ENSSTOT00000008261	ENSTBET00000005596	ENSTSYT00000013767
ENST00000297596	ENSOGAT00000007854	ENSPTRT00000037794	ENSBTAT00000009992	ENSNLET00000003885	ENSGGOT00000013065	ENSCPOT00000009801	ENSDORT00000003903	ENSMMUT00000027773	ENSCJAT00000000162	ENSMUST00000029868	ENSMICT00000015215	ENSPPYT00000021872	ENSOPRT00000001702	ENSOCUT00000001030	ENSRNOT00000020826	ENSSTOT00000010782	ENSTBET00000009792	ENSTSYT00000006298
ENST00000278715	ENSOGAT00000007294	ENSPTRT00000008109	ENSBTAT00000007052	ENSNLET00000009431	ENSGGOT00000008096	ENSCPOT00000006534	ENSDORT00000001976	ENSMMUT00000020856	ENSCJAT00000027014	ENSMUST00000077353	ENSMICT00000002714	ENSPPYT00000004709	ENSOPRT00000016288	ENSOCUT00000015162	ENSRNOT00000014127	ENSSTOT00000014420	ENSTBET00000010846	ENSTSYT00000003521
ENST00000284987	ENSOGAT00000013029	ENSPTRT00000025785	ENSBTAT00000000849	ENSNLET00000003611	ENSGGOT00000007027	ENSCPOT00000010000	ENSDORT00000015010	ENSMMUT00000018801	ENSCJAT00000027211	ENSMUST00000023611	ENSMICT00000012535	ENSPPYT00000013134	ENSOPRT00000015088	ENSOCUT00000002858	ENSRNOT00000002185	ENSSTOT00000008915	ENSTBET00000010283	ENSTSYT00000000270
ENST00000392822	ENSOGAT00000008298	ENSPTRT00000045409	ENSBTAT00000005403	ENSNLET00000005302	ENSGGOT00000022343	ENSCPOT00000000430	ENSDORT00000014163	ENSMMUT00000006994	ENSCJAT00000000649	ENSMUST00000061322	ENSMICT00000001346	ENSPPYT00000016559	ENSOPRT00000011624	ENSOCUT00000013546	ENSRNOT00000017151	ENSSTOT00000010142	ENSTBET00000008385	ENSTSYT00000001358
ENST00000415871	ENSOGAT00000004212	ENSPTRT00000036344	ENSBTAT00000020385	ENSNLET00000015057	ENSGGOT00000041113	ENSCPOT00000011966	ENSDORT00000003103	ENSMMUT00000032795	ENSCJAT00000033285	ENSMUST00000031678	ENSMICT00000000958	ENSPPYT00000020915	ENSOPRT00000016906	ENSOCUT00000001875	ENSRNOT00000007020	ENSSTOT00000000207	ENSTBET00000013109	ENSTSYT00000001068
ENST00000451137	ENSOGAT00000010963	ENSPTRT00000001732	ENSBTAT00000012977	ENSNLET00000012833	ENSGGOT00000007004	ENSCPOT00000010211	ENSDORT00000005601	ENSMMUT00000018514	ENSCJAT00000024719	ENSMUST00000029846	ENSMICT00000015761	ENSPPYT00000001427	ENSOPRT00000011369	ENSOCUT00000003903	ENSRNOT00000019501	ENSSTOT00000003224	ENSTBET00000009007	ENSTSYT00000004928
ENST00000377603	ENSOGAT00000002918	ENSPTRT00000024694	ENSBTAT00000011619	ENSNLET00000012508	ENSGGOT00000011591	ENSCPOT00000002433	ENSDORT00000016353	ENSMMUT00000005881	ENSCJAT00000009963	ENSMUST00000028914	ENSMICT00000010736	ENSPPYT00000012475	ENSOPRT00000003377	ENSOCUT00000015066	ENSRNOT00000010468	ENSSTOT00000013588	ENSTBET00000007921	ENSTSYT00000004206
ENST00000355619	ENSOGAT00000000250	ENSPTRT00000047025	ENSBTAT00000025095	ENSNLET00000020692	ENSGGOT00000007033	ENSCPOT00000005037	ENSDORT00000003960	ENSMMUT00000030204	ENSCJAT00000041690	ENSMUST00000022720	ENSMICT00000002669	ENSPPYT00000006425	ENSOPRT00000000185	ENSOCUT00000011896	ENSRNOT00000057789	ENSSTOT00000002589	ENSTBET00000000722	ENSTSYT00000013859
ENST00000370903	ENSOGAT00000013051	ENSPTRT00000041535	ENSBTAT00000064385	ENSNLET00000019585	ENSGGOT00000008821	ENSCPOT00000014571	ENSDORT00000011660	ENSMMUT00000015587	ENSCJAT00000028219	ENSMUST00000033442	ENSMICT00000015222	ENSPPYT00000024168	ENSOPRT00000009624	ENSOCUT00000009775	ENSRNOT00000010325	ENSSTOT00000012047	ENSTBET00000002291	ENSTSYT00000009991
ENST00000536272	ENSOGAT00000031903	ENSPTRT00000047499	ENSBTAT00000021628	ENSNLET00000021079	ENSGGOT00000014370	ENSCPOT00000009253	ENSDORT00000011368	ENSMMUT00000046668	ENSCJAT00000034555	ENSMUST00000052420	ENSMICT00000015399	ENSPPYT00000022755	ENSOPRT00000006072	ENSOCUT00000016709	ENSRNOT00000022680	ENSSTOT00000012227	ENSTBET00000012848	ENSTSYT00000005983
ENST00000373765	ENSOGAT00000014956	ENSPTRT00000000877	ENSBTAT00000043181	ENSNLET00000001377	ENSGGOT00000007861	ENSCPOT00000010239	ENSDORT00000009341	ENSMMUT00000008987	ENSCJAT00000011602	ENSMUST00000102576	ENSMICT00000011540	ENSPPYT00000001935	ENSOPRT00000001724	ENSOCUT00000008153	ENSRNOT00000014666	ENSSTOT00000014705	ENSTBET00000012310	ENSTSYT00000008445
ENST00000555840	ENSOGAT00000015114	ENSPTRT00000011872	ENSBTAT00000023676	ENSNLET00000001967	ENSGGOT00000004777	ENSCPOT00000007152	ENSDORT00000014060	ENSMMUT00000039754	ENSCJAT00000054532	ENSMUST00000085245	ENSMICT00000009629	ENSPPYT00000007020	ENSOPRT00000000089	ENSOCUT00000004639	ENSRNOT00000006798	ENSSTOT00000012035	ENSTBET00000003841	ENSTSYT00000011100
ENST00000504595	ENSOGAT00000003174	ENSPTRT00000031097	ENSBTAT00000004177	ENSNLET00000015132	ENSGGOT00000011198	ENSCPOT00000013256	ENSDORT00000005829	ENSMMUT00000018754	ENSCJAT00000021097	ENSMUST00000059204	ENSMICT00000010530	ENSPPYT00000017847	ENSOPRT00000008229	ENSOCUT00000004403	ENSRNOT00000031170	ENSSTOT00000006654	ENSTBET00000012773	ENSTSYT00000002113
ENST00000379923	ENSOGAT00000010531	ENSPTRT00000038561	ENSBTAT00000000731	ENSNLET00000004434	ENSGGOT00000016384	ENSCPOT00000003812	ENSDORT00000014246	ENSMMUT00000011453	ENSCJAT00000015072	ENSMUST00000102973	ENSMICT00000006541	ENSPPYT00000022325	ENSOPRT00000016799	ENSOCUT00000015998	ENSRNOT00000008337	ENSSTOT00000006974	ENSTBET00000012390	ENSTSYT00000004187
ENST00000261556	ENSOGAT00000006981	ENSPTRT00000011684	ENSBTAT00000052373	ENSNLET00000016569	ENSGGOT00000015689	ENSCPOT00000005840	ENSDORT00000002946	ENSMMUT00000029373	ENSCJAT00000036412	ENSMUST00000111735	ENSMICT00000016996	ENSPPYT00000006904	ENSOPRT00000001827	ENSOCUT00000002624	ENSRNOT00000017871	ENSSTOT00000012688	ENSTBET00000013592	ENSTSYT00000013660
ENST00000396294	ENSOGAT00000012841	ENSPTRT00000011516	ENSBTAT00000000857	ENSNLET00000005002	ENSGGOT00000017038	ENSCPOT00000015606	ENSDORT00000006393	ENSMMUT00000013472	ENSCJAT00000031963	ENSMUST00000130447	ENSMICT00000003129	ENSPPYT00000006799	ENSOPRT00000013983	ENSOCUT00000003201	ENSRNOT00000059397	ENSSTOT00000011083	ENSTBET00000001832	ENSTSYT00000000527
ENST00000284116	ENSOGAT00000005846	ENSPTRT00000017359	ENSBTAT00000048450	ENSNLET00000010990	ENSGGOT00000003016	ENSCPOT00000011912	ENSDORT00000003735	ENSMMUT00000004799	ENSCJAT00000003076	ENSMUST00000020804	ENSMICT00000004588	ENSPPYT00000009615	ENSOPRT00000005016	ENSOCUT00000013113	ENSRNOT00000042199	ENSSTOT00000007580	ENSTBET00000012889	ENSTSYT00000001220
ENST00000337851	ENSOGAT00000014036	ENSPTRT00000072826	ENSBTAT00000061429	ENSNLET00000004935	ENSGGOT00000008155	ENSCPOT00000003782	ENSDORT00000000345	ENSMMUT00000006477	ENSCJAT00000014333	ENSMUST00000077221	ENSMICT00000003016	ENSPPYT00000018601	ENSOPRT00000008394	ENSOCUT00000001999	ENSRNOT00000003224	ENSSTOT00000004345	ENSTBET00000011725	ENSTSYT00000013870
ENST00000343813	ENSOGAT00000001698	ENSPTRT00000000176	ENSBTAT00000064297	ENSNLET00000013246	ENSGGOT00000005082	ENSCPOT00000021667	ENSDORT00000005186	ENSMMUT00000025788	ENSCJAT00000000273	ENSMUST00000048892	ENSMICT00000009427	ENSPPYT00000002328	ENSOPRT00000011023	ENSOCUT00000005002	ENSRNOT00000014625	ENSSTOT00000014189	ENSTBET00000001949	ENSTSYT00000004064
ENST00000393085	ENSOGAT00000024473	ENSPTRT00000050296	ENSBTAT00000010269	ENSNLET00000017342	ENSGGOT00000002525	ENSCPOT00000013253	ENSDORT00000012769	ENSMMUT00000024055	ENSCJAT00000024772	ENSMUST00000031866	ENSMICT00000004977	ENSPPYT00000021028	ENSOPRT00000010483	ENSOCUT00000005642	ENSRNOT00000015808	ENSSTOT00000008210	ENSTBET00000000928	ENSTSYT00000014292
ENST00000265598	ENSOGAT00000000661	ENSPTRT00000029200	ENSBTAT00000008608	ENSNLET00000007665	ENSGGOT00000005754	ENSCPOT00000015379	ENSDORT00000000477	ENSMMUT00000014774	ENSCJAT00000061794	ENSMUST00000081880	ENSMICT00000005519	ENSPPYT00000016672	ENSOPRT00000007279	ENSOCUT00000005561	ENSRNOT00000034498	ENSSTOT00000010373	ENSTBET00000011545	ENSTSYT00000002293
ENST00000361972	ENSOGAT00000003214	ENSPTRT00000004162	ENSBTAT00000007770	ENSNLET00000015063	ENSGGOT00000002129	ENSCPOT00000002741	ENSDORT00000011203	ENSMMUT00000026967	ENSCJAT00000040484	ENSMUST00000114868	ENSMICT00000000721	ENSPPYT00000002471	ENSOPRT00000001845	ENSOCUT00000013282	ENSRNOT00000020353	ENSSTOT00000012908	ENSTBET00000014158	ENSTSYT00000011559
ENST00000264106	ENSOGAT00000010773	ENSPTRT00000045930	ENSBTAT00000022960	ENSNLET00000007157	ENSGGOT00000007862	ENSCPOT00000008493	ENSDORT00000005158	ENSMMUT00000003360	ENSCJAT00000014662	ENSMUST00000112101	ENSMICT00000006992	ENSPPYT00000015026	ENSOPRT00000005653	ENSOCUT00000000403	ENSRNOT00000045394	ENSSTOT00000006448	ENSTBET00000013385	ENSTSYT00000007380
ENST00000389797	ENSOGAT00000010046	ENSPTRT00000010765	ENSBTAT00000003856	ENSNLET00000003637	ENSGGOT00000006865	ENSCPOT00000011180	ENSDORT00000013994	ENSMMUT00000022884	ENSCJAT00000018282	ENSMUST00000088970	ENSMICT00000000873	ENSPPYT00000006331	ENSOPRT00000011441	ENSOCUT00000009950	ENSRNOT00000012590	ENSSTOT00000002844	ENSTBET00000004552	ENSTSYT00000010423
ENST00000461166	ENSOGAT00000003457	ENSPTRT00000029030	ENSBTAT00000016071	ENSNLET00000005069	ENSGGOT00000030310	ENSCPOT00000000348	ENSDORT00000013554	ENSMMUT00000045586	ENSCJAT00000008950	ENSMUST00000047906	ENSMICT00000011229	ENSPPYT00000016547	ENSOPRT00000003949	ENSOCUT00000014910	ENSRNOT00000015387	ENSSTOT00000009474	ENSTBET00000005635	ENSTSYT00000009807
ENST00000283871	ENSOGAT00000005961	ENSPTRT00000028524	ENSBTAT00000061425	ENSNLET00000009818	ENSGGOT00000009075	ENSCPOT00000022924	ENSDORT00000014505	ENSMMUT00000002253	ENSCJAT00000016882	ENSMUST00000160847	ENSMICT00000005229	ENSPPYT00000015703	ENSOPRT00000018320	ENSOCUT00000009007	ENSRNOT00000003645	ENSSTOT00000015729	ENSTBET00000005102	ENSTSYT00000001686
ENST00000228641	ENSOGAT00000001039	ENSPTRT00000009671	ENSBTAT00000018607	ENSNLET00000006611	ENSGGOT00000004389	ENSCPOT00000007837	ENSDORT00000015435	ENSMMUT00000016343	ENSCJAT00000037915	ENSMUST00000044210	ENSMICT00000004269	ENSPPYT00000005686	ENSOPRT00000008990	ENSOCUT00000001454	ENSRNOT00000006523	ENSSTOT00000015805	ENSTBET00000007822	ENSTSYT00000003254
ENST00000541830	ENSOGAT00000010669	ENSPTRT00000011402	ENSBTAT00000013524	ENSNLET00000019674	ENSGGOT00000004718	ENSCPOT00000014554	ENSDORT00000012288	ENSMMUT00000014027	ENSCJAT00000012931	ENSMUST00000001497	ENSMICT00000001768	ENSPPYT00000006737	ENSOPRT00000016683	ENSOCUT00000002953	ENSRNOT00000027612	ENSSTOT00000002417	ENSTBET00000007178	ENSTSYT00000014214
ENST00000311955	ENSOGAT00000013394	ENSPTRT00000037935	ENSBTAT00000024417	ENSNLET00000005156	ENSGGOT00000016337	ENSCPOT00000027792	ENSDORT00000000539	ENSMMUT00000025087	ENSCJAT00000012774	ENSMUST00000054742	ENSMICT00000015693	ENSPPYT00000021955	ENSOPRT00000012661	ENSOCUT00000006567	ENSRNOT00000010539	ENSSTOT00000011663	ENSTBET00000004915	ENSTSYT00000010095
ENST00000396229	ENSOGAT00000010044	ENSPTRT00000065145	ENSBTAT00000037444	ENSNLET00000006851	ENSGGOT00000004241	ENSCPOT00000022132	ENSDORT00000000800	ENSMMUT00000039509	ENSCJAT00000019148	ENSMUST00000121715	ENSMICT00000011047	ENSPPYT00000008414	ENSOPRT00000002123	ENSOCUT00000003591	ENSRNOT00000055103	ENSSTOT00000008605	ENSTBET00000016704	ENSTSYT00000002283
ENST00000374492	ENSOGAT00000001828	ENSPTRT00000024956	ENSBTAT00000004969	ENSNLET00000013034	ENSGGOT00000013221	ENSCPOT00000005326	ENSDORT00000006204	ENSMMUT00000014003	ENSCJAT00000034940	ENSMUST00000040833	ENSMICT00000005034	ENSPPYT00000012704	ENSOPRT00000011114	ENSOCUT00000016588	ENSRNOT00000026120	ENSSTOT00000011911	ENSTBET00000009384	ENSTSYT00000002137
ENST00000394691	ENSOGAT00000002752	ENSPTRT00000011794	ENSBTAT00000063605	ENSNLET00000018212	ENSGGOT00000009419	ENSCPOT00000006768	ENSDORT00000001278	ENSMMUT00000026929	ENSCJAT00000038465	ENSMUST00000101279	ENSMICT00000013046	ENSPPYT00000006974	ENSOPRT00000013117	ENSOCUT00000000289	ENSRNOT00000008573	ENSSTOT00000010217	ENSTBET00000010207	ENSTSYT00000001239
ENST00000394642	ENSOGAT00000015464	ENSPTRT00000016580	ENSBTAT00000011732	ENSNLET00000007669	ENSGGOT00000013379	ENSCPOT00000000154	ENSDORT00000013275	ENSMMUT00000031450	ENSCJAT00000005380	ENSMUST00000040865	ENSMICT00000011885	ENSPPYT00000009551	ENSOPRT00000014840	ENSOCUT00000009594	ENSRNOT00000037072	ENSSTOT00000011945	ENSTBET00000011281	ENSTSYT00000007294
ENST00000336909	ENSOGAT00000007609	ENSPTRT00000031324	ENSBTAT00000023595	ENSNLET00000002790	ENSGGOT00000003750	ENSCPOT00000013484	ENSDORT00000001451	ENSMMUT00000002670	ENSCJAT00000003998	ENSMUST00000070731	ENSMICT00000011535	ENSPPYT00000017994	ENSOPRT00000005911	ENSOCUT00000007840	ENSRNOT00000018877	ENSSTOT00000005019	ENSTBET00000016213	ENSTSYT00000002561
ENST00000267396	ENSOGAT00000033326	ENSPTRT00000011270	ENSBTAT00000021784	ENSNLET00000018878	ENSGGOT00000015754	ENSCPOT00000024435	ENSDORT00000004925	ENSMMUT00000022639	ENSCJAT00000016575	ENSMUST00000164766	ENSMICT00000006357	ENSPPYT00000006673	ENSOPRT00000001069	ENSOCUT00000001253	ENSRNOT00000016020	ENSSTOT00000010340	ENSTBET00000011431	ENSTSYT00000006121
ENST00000200135	ENSOGAT00000011587	ENSPTRT00000007998	ENSBTAT00000026733	ENSNLET00000009340	ENSGGOT00000015340	ENSCPOT00000005152	ENSDORT00000011416	ENSMMUT00000033457	ENSCJAT00000023689	ENSMUST00000034803	ENSMICT00000009594	ENSPPYT00000004629	ENSOPRT00000002784	ENSOCUT00000007437	ENSRNOT00000010595	ENSSTOT00000008806	ENSTBET00000001385	ENSTSYT00000013471
ENST00000395578	ENSOGAT00000011256	ENSPTRT00000030172	ENSBTAT00000003877	ENSNLET00000010921	ENSGGOT00000006633	ENSCPOT00000015081	ENSDORT00000008383	ENSMMUT00000027910	ENSCJAT00000029427	ENSMUST00000161490	ENSMICT00000017519	ENSPPYT00000017294	ENSOPRT00000004901	ENSOCUT00000008473	ENSRNOT00000003237	ENSSTOT00000011250	ENSTBET00000017708	ENSTSYT00000012736
ENST00000556155	ENSOGAT00000005006	ENSPTRT00000009405	ENSBTAT00000008311	ENSNLET00000022210	ENSGGOT00000001436	ENSCPOT00000013066	ENSDORT00000012550	ENSMMUT00000024650	ENSCJAT00000060660	ENSMUST00000092074	ENSMICT00000009093	ENSPPYT00000005542	ENSOPRT00000013425	ENSOCUT00000007933	ENSRNOT00000039306	ENSSTOT00000013291	ENSTBET00000013688	ENSTSYT00000000266
ENST00000503451	ENSOGAT00000005509	ENSPTRT00000030412	ENSBTAT00000029948	ENSNLET00000016712	ENSGGOT00000025863	ENSCPOT00000006765	ENSDORT00000009968	ENSMMUT00000041906	ENSCJAT00000030779	ENSMUST00000093971	ENSMICT00000010776	ENSPPYT00000017408	ENSOPRT00000001174	ENSOCUT00000012534	ENSRNOT00000055544	ENSSTOT00000000887	ENSTBET00000002729	ENSTSYT00000012242
ENST00000371852	ENSOGAT00000002214	ENSPTRT00000005163	ENSBTAT00000014758	ENSNLET00000023659	ENSGGOT00000034259	ENSCPOT00000006517	ENSDORT00000000931	ENSMMUT00000042766	ENSCJAT00000043315	ENSMUST00000050562	ENSMICT00000004518	ENSPPYT00000002952	ENSOPRT00000015074	ENSOCUT00000000545	ENSRNOT00000025856	ENSSTOT00000013088	ENSTBET00000005265	ENSTSYT00000011613
ENST00000371201	ENSOGAT00000011718	ENSPTRT00000001512	ENSBTAT00000012479	ENSNLET00000018934	ENSGGOT00000011957	ENSCPOT00000006111	ENSDORT00000006672	ENSMMUT00000009455	ENSCJAT00000031415	ENSMUST00000107071	ENSMICT00000013486	ENSPPYT00000001554	ENSOPRT00000002204	ENSOCUT00000016080	ENSRNOT00000036030	ENSSTOT00000001017	ENSTBET00000009937	ENSTSYT00000002236
ENST00000417449	ENSOGAT00000004958	ENSPTRT00000007911	ENSBTAT00000014068	ENSNLET00000009218	ENSGGOT00000015560	ENSCPOT00000006944	ENSDORT00000008596	ENSMMUT00000007650	ENSCJAT00000019347	ENSMUST00000053407	ENSMICT00000001043	ENSPPYT00000004564	ENSOPRT00000009603	ENSOCUT00000011929	ENSRNOT00000003432	ENSSTOT00000007140	ENSTBET00000002726	ENSTSYT00000007339
ENST00000278483	ENSOGAT00000013962	ENSPTRT00000045765	ENSBTAT00000026632	ENSNLET00000020666	ENSGGOT00000031272	ENSCPOT00000026664	ENSDORT00000012508	ENSMMUT00000024921	ENSCJAT00000000123	ENSMUST00000153470	ENSMICT00000000944	ENSPPYT00000004459	ENSOPRT00000013859	ENSOCUT00000013105	ENSRNOT00000023468	ENSSTOT00000004693	ENSTBET00000009184	ENSTSYT00000003298
ENST00000222573	ENSOGAT00000003842	ENSPTRT00000035053	ENSBTAT00000004833	ENSNLET00000021259	ENSGGOT00000002488	ENSCPOT00000009193	ENSDORT00000000272	ENSMMUT00000009639	ENSCJAT00000002157	ENSMUST00000026360	ENSMICT00000009245	ENSPPYT00000020680	ENSOPRT00000011596	ENSOCUT00000002200	ENSRNOT00000008707	ENSSTOT00000016399	ENSTBET00000001629	ENSTSYT00000005456
ENST00000358161	ENSOGAT00000006492	ENSPTRT00000048390	ENSBTAT00000020395	ENSNLET00000022423	ENSGGOT00000007673	ENSCPOT00000009517	ENSDORT00000011866	ENSMMUT00000013037	ENSCJAT00000039594	ENSMUST00000100225	ENSMICT00000017641	ENSPPYT00000022937	ENSOPRT00000017093	ENSOCUT00000012350	ENSRNOT00000060787	ENSSTOT00000010528	ENSTBET00000009684	ENSTSYT00000005117
ENST00000370693	ENSOGAT00000000675	ENSPTRT00000033848	ENSBTAT00000007039	ENSNLET00000003560	ENSGGOT00000025441	ENSCPOT00000002525	ENSDORT00000008361	ENSMMUT00000025287	ENSCJAT00000019957	ENSMUST00000044691	ENSMICT00000014022	ENSPPYT00000019471	ENSOPRT00000010845	ENSOCUT00000004813	ENSRNOT00000016948	ENSSTOT00000010959	ENSTBET00000008241	ENSTSYT00000011222
ENST00000322564	ENSOGAT00000034293	ENSPTRT00000011874	ENSBTAT00000011311	ENSNLET00000001984	ENSGGOT00000011471	ENSCPOT00000020168	ENSDORT00000011234	ENSMMUT00000004584	ENSCJAT00000029061	ENSMUST00000140770	ENSMICT00000009636	ENSPPYT00000007021	ENSOPRT00000000091	ENSOCUT00000004911	ENSRNOT00000058301	ENSSTOT00000012046	ENSTBET00000004092	ENSTSYT00000004912
ENST00000524487	ENSOGAT00000027515	ENSPTRT00000045759	ENSBTAT00000028986	ENSNLET00000018685	ENSGGOT00000007360	ENSCPOT00000015607	ENSDORT00000000756	ENSMMUT00000013254	ENSCJAT00000039526	ENSMUST00000111446	ENSMICT00000007147	ENSPPYT00000003936	ENSOPRT00000013644	ENSOCUT00000008891	ENSRNOT00000068444	ENSSTOT00000011053	ENSTBET00000015885	ENSTSYT00000011536
ENST00000216489	ENSOGAT00000006932	ENSPTRT00000012081	ENSBTAT00000010693	ENSNLET00000021363	ENSGGOT00000011095	ENSCPOT00000001518	ENSDORT00000012377	ENSMMUT00000033234	ENSCJAT00000032935	ENSMUST00000162961	ENSMICT00000012069	ENSPPYT00000007126	ENSOPRT00000007540	ENSOCUT00000001437	ENSRNOT00000016389	ENSSTOT00000003255	ENSTBET00000013823	ENSTSYT00000010944
ENST00000296575	ENSOGAT00000006904	ENSPTRT00000030632	ENSBTAT00000021390	ENSNLET00000006924	ENSGGOT00000013413	ENSCPOT00000026796	ENSDORT00000016419	ENSMMUT00000011148	ENSCJAT00000005175	ENSMUST00000079038	ENSMICT00000003991	ENSPPYT00000017544	ENSOPRT00000014177	ENSOCUT00000004962	ENSRNOT00000024616	ENSSTOT00000006707	ENSTBET00000001478	ENSTSYT00000006026
ENST00000258534	ENSOGAT00000003320	ENSPTRT00000009849	ENSBTAT00000022976	ENSNLET00000008105	ENSGGOT00000003782	ENSCPOT00000027979	ENSDORT00000003108	ENSMMUT00000006622	ENSCJAT00000063487	ENSMUST00000020249	ENSMICT00000012066	ENSPPYT00000015947	ENSOPRT00000001202	ENSOCUT00000016493	ENSRNOT00000059487	ENSSTOT00000001863	ENSTBET00000012576	ENSTSYT00000002445
ENST00000397822	ENSOGAT00000016374	ENSPTRT00000044447	ENSBTAT00000017489	ENSNLET00000004292	ENSGGOT00000003804	ENSCPOT00000014649	ENSDORT00000005651	ENSMMUT00000014421	ENSCJAT00000039012	ENSMUST00000056290	ENSMICT00000017402	ENSPPYT00000013578	ENSOPRT00000007430	ENSOCUT00000005276	ENSRNOT00000010690	ENSSTOT00000005423	ENSTBET00000017083	ENSTSYT00000014617
ENST00000368219	ENSOGAT00000016821	ENSPTRT00000002705	ENSBTAT00000001989	ENSNLET00000016248	ENSGGOT00000016536	ENSCPOT00000026396	ENSDORT00000013737	ENSMMUT00000000053	ENSCJAT00000016004	ENSMUST00000055984	ENSMICT00000008924	ENSPPYT00000000848	ENSOPRT00000000631	ENSOCUT00000001458	ENSRNOT00000015471	ENSSTOT00000007728	ENSTBET00000015027	ENSTSYT00000002109
ENST00000465530	ENSOGAT00000004729	ENSPTRT00000004236	ENSBTAT00000021871	ENSNLET00000015484	ENSGGOT00000000867	ENSCPOT00000008560	ENSDORT00000004463	ENSMMUT00000024139	ENSCJAT00000018182	ENSMUST00000036350	ENSMICT00000002784	ENSPPYT00000002516	ENSOPRT00000009423	ENSOCUT00000012867	ENSRNOT00000036299	ENSSTOT00000014760	ENSTBET00000005206	ENSTSYT00000014460
ENST00000371547	ENSOGAT00000016994	ENSPTRT00000050933	ENSBTAT00000028134	ENSNLET00000016218	ENSGGOT00000024684	ENSCPOT00000003850	ENSDORT00000006735	ENSMMUT00000015210	ENSCJAT00000016976	ENSMUST00000066439	ENSMICT00000013106	ENSPPYT00000002982	ENSOPRT00000004876	ENSOCUT00000000175	ENSRNOT00000054748	ENSSTOT00000015612	ENSTBET00000003737	ENSTSYT00000011929
ENST00000371485	ENSOGAT00000013344	ENSPTRT00000005223	ENSBTAT00000006763	ENSNLET00000016380	ENSGGOT00000003976	ENSCPOT00000007377	ENSDORT00000004053	ENSMMUT00000020621	ENSCJAT00000061753	ENSMUST00000169673	ENSMICT00000005490	ENSPPYT00000002988	ENSOPRT00000004284	ENSOCUT00000010515	ENSRNOT00000022133	ENSSTOT00000016094	ENSTBET00000006009	ENSTSYT00000003447
ENST00000258449	ENSOGAT00000007267	ENSPTRT00000022850	ENSBTAT00000028967	ENSNLET00000013504	ENSGGOT00000008259	ENSCPOT00000001627	ENSDORT00000015470	ENSMMUT00000022175	ENSCJAT00000015869	ENSMUST00000095014	ENSMICT00000000226	ENSPPYT00000014071	ENSOPRT00000002210	ENSOCUT00000015028	ENSRNOT00000037094	ENSSTOT00000008882	ENSTBET00000009757	ENSTSYT00000002887
ENST00000431505	ENSOGAT00000001198	ENSPTRT00000046615	ENSBTAT00000027954	ENSNLET00000007536	ENSGGOT00000010922	ENSCPOT00000020920	ENSDORT00000007132	ENSMMUT00000004473	ENSCJAT00000027906	ENSMUST00000017836	ENSMICT00000007781	ENSPPYT00000009541	ENSOPRT00000014901	ENSOCUT00000008177	ENSRNOT00000007312	ENSSTOT00000000694	ENSTBET00000005020	ENSTSYT00000010942
ENST00000316157	ENSOGAT00000011547	ENSPTRT00000004091	ENSBTAT00000018457	ENSNLET00000017161	ENSGGOT00000003591	ENSCPOT00000009460	ENSDORT00000009328	ENSMMUT00000007426	ENSCJAT00000036106	ENSMUST00000091829	ENSMICT00000001302	ENSPPYT00000002425	ENSOPRT00000003317	ENSOCUT00000004955	ENSRNOT00000058548	ENSSTOT00000000587	ENSTBET00000005418	ENSTSYT00000002580
ENST00000369516	ENSOGAT00000011524	ENSPTRT00000002137	ENSBTAT00000021222	ENSNLET00000006331	ENSGGOT00000010123	ENSCPOT00000008451	ENSDORT00000003241	ENSMMUT00000046295	ENSCJAT00000024700	ENSMUST00000029451	ENSMICT00000002159	ENSPPYT00000001195	ENSOPRT00000003014	ENSOCUT00000001279	ENSRNOT00000035605	ENSSTOT00000005517	ENSTBET00000005099	ENSTSYT00000012981
ENST00000296003	ENSOGAT00000015624	ENSPTRT00000042203	ENSBTAT00000052053	ENSNLET00000005152	ENSGGOT00000003554	ENSCPOT00000010424	ENSDORT00000011006	ENSMMUT00000010937	ENSCJAT00000031173	ENSMUST00000113146	ENSMICT00000015194	ENSPPYT00000015909	ENSOPRT00000002148	ENSOCUT00000010089	ENSRNOT00000010723	ENSSTOT00000002051	ENSTBET00000007543	ENSTSYT00000005714
ENST00000376509	ENSOGAT00000009614	ENSPTRT00000040645	ENSBTAT00000006032	ENSNLET00000010130	ENSGGOT00000011481	ENSCPOT00000006175	ENSDORT00000002658	ENSMMUT00000006443	ENSCJAT00000004801	ENSMUST00000033495	ENSMICT00000005980	ENSPPYT00000023715	ENSOPRT00000010287	ENSOCUT00000015756	ENSRNOT00000011207	ENSSTOT00000014197	ENSTBET00000008919	ENSTSYT00000010015
ENST00000304760	ENSOGAT00000013074	ENSPTRT00000002540	ENSBTAT00000027370	ENSNLET00000014499	ENSGGOT00000015835	ENSCPOT00000001175	ENSDORT00000007804	ENSMMUT00000032345	ENSCJAT00000018520	ENSMUST00000050401	ENSMICT00000001412	ENSPPYT00000000940	ENSOPRT00000008624	ENSOCUT00000016366	ENSRNOT00000028225	ENSSTOT00000009308	ENSTBET00000012374	ENSTSYT00000001055
ENST00000377799	ENSOGAT00000006664	ENSPTRT00000049273	ENSBTAT00000027378	ENSNLET00000016019	ENSGGOT00000032549	ENSCPOT00000001062	ENSDORT00000004493	ENSMMUT00000004512	ENSCJAT00000062318	ENSMUST00000124488	ENSMICT00000011569	ENSPPYT00000002538	ENSOPRT00000011022	ENSOCUT00000007233	ENSRNOT00000038651	ENSSTOT00000013694	ENSTBET00000000364	ENSTSYT00000002999
ENST00000370544	ENSOGAT00000002297	ENSPTRT00000001754	ENSBTAT00000000389	ENSNLET00000013030	ENSGGOT00000000365	ENSCPOT00000012229	ENSDORT00000008315	ENSMMUT00000024447	ENSCJAT00000020830	ENSMUST00000121796	ENSMICT00000003017	ENSPPYT00000001413	ENSOPRT00000016193	ENSOCUT00000012635	ENSRNOT00000067502	ENSSTOT00000006663	ENSTBET00000013185	ENSTSYT00000005788
ENST00000426333	ENSOGAT00000012924	ENSPTRT00000017060	ENSBTAT00000017986	ENSNLET00000006632	ENSGGOT00000013038	ENSCPOT00000010661	ENSDORT00000011567	ENSMMUT00000018410	ENSCJAT00000024303	ENSMUST00000107060	ENSMICT00000016599	ENSPPYT00000009729	ENSOPRT00000016335	ENSOCUT00000006885	ENSRNOT00000055176	ENSSTOT00000012191	ENSTBET00000011374	ENSTSYT00000013172
ENST00000541917	ENSOGAT00000007418	ENSPTRT00000023764	ENSBTAT00000048464	ENSNLET00000008934	ENSGGOT00000010832	ENSCPOT00000000025	ENSDORT00000003748	ENSMMUT00000002651	ENSCJAT00000009562	ENSMUST00000114243	ENSMICT00000008944	ENSPPYT00000015208	ENSOPRT00000009003	ENSOCUT00000011448	ENSRNOT00000037555	ENSSTOT00000002049	ENSTBET00000011180	ENSTSYT00000013820
ENST00000436589	ENSOGAT00000001720	ENSPTRT00000032865	ENSBTAT00000005079	ENSNLET00000003402	ENSGGOT00000032163	ENSCPOT00000023341	ENSDORT00000007164	ENSMMUT00000041315	ENSCJAT00000042369	ENSMUST00000167746	ENSMICT00000016353	ENSPPYT00000018936	ENSOPRT00000013532	ENSOCUT00000004431	ENSRNOT00000018699	ENSSTOT00000013067	ENSTBET00000016842	ENSTSYT00000011335
ENST00000262065	ENSOGAT00000005904	ENSPTRT00000017295	ENSBTAT00000000244	ENSNLET00000010222	ENSGGOT00000028767	ENSCPOT00000011107	ENSDORT00000014019	ENSMMUT00000031842	ENSCJAT00000021967	ENSMUST00000004050	ENSMICT00000002862	ENSPPYT00000009650	ENSOPRT00000004201	ENSOCUT00000010549	ENSRNOT00000003308	ENSSTOT00000016230	ENSTBET00000005292	ENSTSYT00000003487
ENST00000296318	ENSOGAT00000007785	ENSPTRT00000028164	ENSBTAT00000060984	ENSNLET00000009265	ENSGGOT00000005038	ENSCPOT00000009676	ENSDORT00000005511	ENSMMUT00000023322	ENSCJAT00000021394	ENSMUST00000035336	ENSMICT00000001725	ENSPPYT00000016025	ENSOPRT00000005134	ENSOCUT00000014052	ENSRNOT00000030102	ENSSTOT00000000370	ENSTBET00000017234	ENSTSYT00000010619
ENST00000315366	ENSOGAT00000031747	ENSPTRT00000030477	ENSBTAT00000023672	ENSNLET00000020640	ENSGGOT00000012745	ENSCPOT00000009369	ENSDORT00000016246	ENSMMUT00000033196	ENSCJAT00000006655	ENSMUST00000093976	ENSMICT00000008139	ENSPPYT00000017450	ENSOPRT00000009100	ENSOCUT00000010866	ENSRNOT00000055633	ENSSTOT00000015415	ENSTBET00000009099	ENSTSYT00000000940
ENST00000371900	ENSOGAT00000001108	ENSPTRT00000041382	ENSBTAT00000000209	ENSNLET00000018801	ENSGGOT00000010872	ENSCPOT00000001849	ENSDORT00000002858	ENSMMUT00000029326	ENSCJAT00000004811	ENSMUST00000033414	ENSMICT00000000875	ENSPPYT00000024093	ENSOPRT00000016044	ENSOCUT00000015029	ENSRNOT00000007967	ENSSTOT00000007157	ENSTBET00000014993	ENSTSYT00000014708
ENST00000416849	ENSOGAT00000008905	ENSPTRT00000036610	ENSBTAT00000026934	ENSNLET00000002259	ENSGGOT00000025728	ENSCPOT00000000064	ENSDORT00000009521	ENSMMUT00000005994	ENSCJAT00000025926	ENSMUST00000003017	ENSMICT00000003158	ENSPPYT00000021051	ENSOPRT00000009935	ENSOCUT00000016141	ENSRNOT00000010796	ENSSTOT00000004558	ENSTBET00000009232	ENSTSYT00000013548
ENST00000265965	ENSOGAT00000011199	ENSPTRT00000006444	ENSBTAT00000007022	ENSNLET00000022631	ENSGGOT00000000690	ENSCPOT00000000913	ENSDORT00000013232	ENSMMUT00000008984	ENSCJAT00000014291	ENSMUST00000033127	ENSMICT00000005579	ENSPPYT00000004109	ENSOPRT00000002438	ENSOCUT00000002411	ENSRNOT00000015352	ENSSTOT00000003391	ENSTBET00000009080	ENSTSYT00000012346
ENST00000286063	ENSOGAT00000013196	ENSPTRT00000023491	ENSBTAT00000021805	ENSNLET00000007724	ENSGGOT00000041138	ENSCPOT00000000687	ENSDORT00000002644	ENSMMUT00000002850	ENSCJAT00000028422	ENSMUST00000099992	ENSMICT00000003776	ENSPPYT00000015079	ENSOPRT00000002585	ENSOCUT00000008038	ENSRNOT00000008301	ENSSTOT00000012644	ENSTBET00000008388	ENSTSYT00000010562
ENST00000236959	ENSOGAT00000005043	ENSPTRT00000023902	ENSBTAT00000025662	ENSNLET00000009227	ENSGGOT00000007953	ENSCPOT00000012927	ENSDORT00000010126	ENSMMUT00000017264	ENSCJAT00000022723	ENSMUST00000027384	ENSMICT00000014576	ENSPPYT00000015285	ENSOPRT00000000538	ENSOCUT00000001121	ENSRNOT00000021105	ENSSTOT00000002693	ENSTBET00000011019	ENSTSYT00000008736
ENST00000356839	ENSOGAT00000004387	ENSPTRT00000015974	ENSBTAT00000003999	ENSNLET00000010260	ENSGGOT00000006789	ENSCPOT00000020475	ENSDORT00000005217	ENSMMUT00000014749	ENSCJAT00000032570	ENSMUST00000102574	ENSMICT00000004312	ENSPPYT00000009250	ENSOPRT00000010096	ENSOCUT00000006970	ENSRNOT00000024973	ENSSTOT00000008585	ENSTBET00000000668	ENSTSYT00000002198
ENST00000309428	ENSOGAT00000009875	ENSPTRT00000016944	ENSBTAT00000046079	ENSNLET00000014905	ENSGGOT00000010933	ENSCPOT00000002880	ENSDORT00000006535	ENSMMUT00000023599	ENSCJAT00000009616	ENSMUST00000017946	ENSMICT00000010270	ENSPPYT00000009805	ENSOPRT00000012333	ENSOCUT00000015226	ENSRNOT00000027217	ENSSTOT00000003701	ENSTBET00000003284	ENSTSYT00000007664
ENST00000324103	ENSOGAT00000016596	ENSPTRT00000011372	ENSBTAT00000061147	ENSNLET00000019487	ENSGGOT00000016092	ENSCPOT00000027401	ENSDORT00000009267	ENSMMUT00000017489	ENSCJAT00000013988	ENSMUST00000019443	ENSMICT00000001706	ENSPPYT00000006720	ENSOPRT00000016583	ENSOCUT00000002864	ENSRNOT00000026333	ENSSTOT00000002270	ENSTBET00000017532	ENSTSYT00000007439
ENST00000225805	ENSOGAT00000001199	ENSPTRT00000016571	ENSBTAT00000027955	ENSNLET00000007553	ENSGGOT00000002529	ENSCPOT00000013289	ENSDORT00000007136	ENSMMUT00000031479	ENSCJAT00000027933	ENSMUST00000103225	ENSMICT00000007786	ENSPPYT00000009542	ENSOPRT00000014926	ENSOCUT00000008184	ENSRNOT00000000254	ENSSTOT00000000703	ENSTBET00000005752	ENSTSYT00000000965
ENST00000375847	ENSOGAT00000005434	ENSPTRT00000000425	ENSBTAT00000003212	ENSNLET00000009718	ENSGGOT00000002628	ENSCPOT00000012175	ENSDORT00000003972	ENSMMUT00000047549	ENSCJAT00000016941	ENSMUST00000038014	ENSMICT00000003464	ENSPPYT00000002201	ENSOPRT00000009462	ENSOCUT00000016057	ENSRNOT00000017247	ENSSTOT00000013805	ENSTBET00000006306	ENSTSYT00000009904
ENST00000429473	ENSOGAT00000005066	ENSPTRT00000035918	ENSBTAT00000003465	ENSNLET00000019669	ENSGGOT00000015112	ENSCPOT00000008504	ENSDORT00000009837	ENSMMUT00000028951	ENSCJAT00000026691	ENSMUST00000031673	ENSMICT00000014534	ENSPPYT00000020751	ENSOPRT00000004505	ENSOCUT00000017033	ENSRNOT00000014219	ENSSTOT00000009659	ENSTBET00000000698	ENSTSYT00000002381
ENST00000367162	ENSOGAT00000007391	ENSPTRT00000003448	ENSBTAT00000022108	ENSNLET00000018435	ENSGGOT00000003536	ENSCPOT00000011879	ENSDORT00000014247	ENSMMUT00000014659	ENSCJAT00000033484	ENSMUST00000045110	ENSMICT00000000052	ENSPPYT00000000353	ENSOPRT00000010145	ENSOCUT00000000098	ENSRNOT00000004810	ENSSTOT00000002678	ENSTBET00000013271	ENSTSYT00000002010
ENST00000355192	ENSOGAT00000025679	ENSPTRT00000046322	ENSBTAT00000060985	ENSNLET00000004015	ENSGGOT00000010191	ENSCPOT00000026165	ENSDORT00000009035	ENSMMUT00000005191	ENSCJAT00000028378	ENSMUST00000092143	ENSMICT00000012110	ENSPPYT00000005603	ENSOPRT00000002597	ENSOCUT00000000887	ENSRNOT00000064251	ENSSTOT00000014007	ENSTBET00000004247	ENSTSYT00000011866
ENST00000367815	ENSOGAT00000010048	ENSPTRT00000002998	ENSBTAT00000063162	ENSNLET00000019070	ENSGGOT00000002513	ENSCPOT00000004263	ENSDORT00000015636	ENSMMUT00000044679	ENSCJAT00000020132	ENSMUST00000027863	ENSMICT00000013182	ENSPPYT00000000651	ENSOPRT00000003503	ENSOCUT00000006867	ENSRNOT00000003932	ENSSTOT00000011469	ENSTBET00000004603	ENSTSYT00000010215
ENST00000295908	ENSOGAT00000017227	ENSPTRT00000030277	ENSBTAT00000007563	ENSNLET00000012358	ENSGGOT00000002780	ENSCPOT00000013968	ENSDORT00000001259	ENSMMUT00000006448	ENSCJAT00000012507	ENSMUST00000042766	ENSMICT00000007652	ENSPPYT00000017341	ENSOPRT00000009989	ENSOCUT00000003450	ENSRNOT00000009202	ENSSTOT00000012513	ENSTBET00000005594	ENSTSYT00000013455
ENST00000301608	ENSOGAT00000001943	ENSPTRT00000017725	ENSBTAT00000029362	ENSNLET00000002485	ENSGGOT00000004616	ENSCPOT00000015288	ENSDORT00000008607	ENSMMUT00000019888	ENSCJAT00000027022	ENSMUST00000072948	ENSMICT00000007685	ENSPPYT00000010093	ENSOPRT00000001503	ENSOCUT00000012925	ENSRNOT00000042372	ENSSTOT00000013440	ENSTBET00000015303	ENSTSYT00000004685
ENST00000372118	ENSOGAT00000025390	ENSPTRT00000033672	ENSBTAT00000056429	ENSNLET00000004949	ENSGGOT00000006330	ENSCPOT00000019445	ENSDORT00000014584	ENSMMUT00000020686	ENSCJAT00000033846	ENSMUST00000024763	ENSMICT00000005061	ENSPPYT00000020135	ENSOPRT00000007681	ENSOCUT00000005544	ENSRNOT00000026499	ENSSTOT00000004486	ENSTBET00000004104	ENSTSYT00000001388
ENST00000233084	ENSOGAT00000008737	ENSPTRT00000021747	ENSBTAT00000013075	ENSNLET00000018501	ENSGGOT00000012548	ENSCPOT00000009905	ENSDORT00000012847	ENSMMUT00000032974	ENSCJAT00000014125	ENSMUST00000071103	ENSMICT00000001029	ENSPPYT00000014681	ENSOPRT00000004324	ENSOCUT00000007013	ENSRNOT00000009100	ENSSTOT00000003976	ENSTBET00000008479	ENSTSYT00000002483
ENST00000534982	ENSOGAT00000003704	ENSPTRT00000027245	ENSBTAT00000055237	ENSNLET00000004295	ENSGGOT00000015999	ENSCPOT00000014999	ENSDORT00000006695	ENSMMUT00000006685	ENSCJAT00000030226	ENSMUST00000032191	ENSMICT00000016628	ENSPPYT00000015929	ENSOPRT00000005848	ENSOCUT00000012348	ENSRNOT00000009008	ENSSTOT00000007536	ENSTBET00000005000	ENSTSYT00000009493
ENST00000396475	ENSOGAT00000003400	ENSPTRT00000035113	ENSBTAT00000020351	ENSNLET00000018937	ENSGGOT00000005185	ENSCPOT00000015717	ENSDORT00000004128	ENSMMUT00000023794	ENSCJAT00000015052	ENSMUST00000036236	ENSMICT00000004209	ENSPPYT00000020650	ENSOPRT00000004282	ENSOCUT00000014660	ENSRNOT00000029332	ENSSTOT00000001908	ENSTBET00000003838	ENSTSYT00000005349
ENST00000458078	ENSOGAT00000024988	ENSPTRT00000036543	ENSBTAT00000065165	ENSNLET00000016828	ENSGGOT00000029022	ENSCPOT00000015699	ENSDORT00000006935	ENSMMUT00000009152	ENSCJAT00000024051	ENSMUST00000115017	ENSMICT00000014565	ENSPPYT00000021014	ENSOPRT00000012779	ENSOCUT00000014755	ENSRNOT00000032077	ENSSTOT00000003857	ENSTBET00000005925	ENSTSYT00000008172
ENST00000237283	ENSOGAT00000014715	ENSPTRT00000034469	ENSBTAT00000037717	ENSNLET00000019232	ENSGGOT00000016232	ENSCPOT00000005426	ENSDORT00000016095	ENSMMUT00000007547	ENSCJAT00000037691	ENSMUST00000019944	ENSMICT00000009793	ENSPPYT00000019855	ENSOPRT00000000145	ENSOCUT00000008925	ENSRNOT00000014239	ENSSTOT00000012822	ENSTBET00000008209	ENSTSYT00000000276
ENST00000310301	ENSOGAT00000000685	ENSPTRT00000035298	ENSBTAT00000009615	ENSNLET00000003850	ENSGGOT00000006709	ENSCPOT00000006206	ENSDORT00000015881	ENSMMUT00000014021	ENSCJAT00000024618	ENSMUST00000072961	ENSMICT00000005041	ENSPPYT00000020528	ENSOPRT00000015554	ENSOCUT00000000677	ENSRNOT00000017640	ENSSTOT00000007372	ENSTBET00000006191	ENSTSYT00000001918
ENST00000369076	ENSOGAT00000004789	ENSPTRT00000034111	ENSBTAT00000007100	ENSNLET00000016945	ENSGGOT00000016687	ENSCPOT00000014207	ENSDORT00000012975	ENSMMUT00000027645	ENSCJAT00000012286	ENSMUST00000039286	ENSMICT00000010329	ENSPPYT00000019645	ENSOPRT00000006070	ENSOCUT00000013357	ENSRNOT00000000354	ENSSTOT00000016509	ENSTBET00000000164	ENSTSYT00000007152
ENST00000264867	ENSOGAT00000014566	ENSPTRT00000029771	ENSBTAT00000022636	ENSNLET00000021359	ENSGGOT00000009852	ENSCPOT00000001538	ENSDORT00000016276	ENSMMUT00000002598	ENSCJAT00000053193	ENSMUST00000132734	ENSMICT00000015400	ENSPPYT00000017022	ENSOPRT00000012902	ENSOCUT00000014670	ENSRNOT00000006071	ENSSTOT00000012525	ENSTBET00000005156	ENSTSYT00000010427
ENST00000258886	ENSOGAT00000010629	ENSPTRT00000013538	ENSBTAT00000003252	ENSNLET00000011048	ENSGGOT00000015714	ENSCPOT00000014046	ENSDORT00000014342	ENSMMUT00000033025	ENSCJAT00000020151	ENSMUST00000034843	ENSMICT00000005764	ENSPPYT00000007909	ENSOPRT00000000344	ENSOCUT00000009910	ENSRNOT00000017900	ENSSTOT00000009287	ENSTBET00000010944	ENSTSYT00000011731
ENST00000545134	ENSOGAT00000001222	ENSPTRT00000009530	ENSBTAT00000063430	ENSNLET00000004151	ENSGGOT00000016298	ENSCPOT00000013818	ENSDORT00000013368	ENSMMUT00000023369	ENSCJAT00000028231	ENSMUST00000020449	ENSMICT00000017066	ENSPPYT00000005611	ENSOPRT00000010254	ENSOCUT00000005911	ENSRNOT00000038147	ENSSTOT00000007079	ENSTBET00000008695	ENSTSYT00000005175
ENST00000304658	ENSOGAT00000012851	ENSPTRT00000014604	ENSBTAT00000013621	ENSNLET00000014795	ENSGGOT00000005642	ENSCPOT00000012949	ENSDORT00000000921	ENSMMUT00000026184	ENSCJAT00000011816	ENSMUST00000168189	ENSMICT00000001929	ENSPPYT00000008498	ENSOPRT00000009292	ENSOCUT00000002734	ENSRNOT00000022502	ENSSTOT00000012172	ENSTBET00000009977	ENSTSYT00000012259
ENST00000265881	ENSOGAT00000016865	ENSPTRT00000008012	ENSBTAT00000007680	ENSNLET00000009356	ENSGGOT00000023899	ENSCPOT00000010643	ENSDORT00000011255	ENSMMUT00000017187	ENSCJAT00000024143	ENSMUST00000034524	ENSMICT00000010091	ENSPPYT00000004638	ENSOPRT00000011067	ENSOCUT00000004272	ENSRNOT00000047633	ENSSTOT00000012466	ENSTBET00000017038	ENSTSYT00000002212
ENST00000381226	ENSOGAT00000002706	ENSPTRT00000031330	ENSBTAT00000018934	ENSNLET00000003001	ENSGGOT00000015295	ENSCPOT00000015174	ENSDORT00000009063	ENSMMUT00000017373	ENSCJAT00000004145	ENSMUST00000109272	ENSMICT00000000431	ENSPPYT00000018001	ENSOPRT00000006011	ENSOCUT00000003135	ENSRNOT00000017571	ENSSTOT00000007234	ENSTBET00000013215	ENSTSYT00000010068
ENST00000446394	ENSOGAT00000006034	ENSPTRT00000005190	ENSBTAT00000015476	ENSNLET00000015866	ENSGGOT00000010323	ENSCPOT00000009003	ENSDORT00000002137	ENSMMUT00000000351	ENSCJAT00000021440	ENSMUST00000047247	ENSMICT00000012056	ENSPPYT00000002972	ENSOPRT00000014611	ENSOCUT00000015038	ENSRNOT00000024963	ENSSTOT00000009029	ENSTBET00000008462	ENSTSYT00000009145
ENST00000264741	ENSOGAT00000004441	ENSPTRT00000027582	ENSBTAT00000022042	ENSNLET00000006521	ENSGGOT00000013104	ENSCPOT00000014795	ENSDORT00000013180	ENSMMUT00000030850	ENSCJAT00000024599	ENSMUST00000044165	ENSMICT00000012259	ENSPPYT00000016300	ENSOPRT00000009119	ENSOCUT00000013705	ENSRNOT00000015113	ENSSTOT00000009420	ENSTBET00000013771	ENSTSYT00000000612
ENST00000333640	ENSOGAT00000007977	ENSPTRT00000009143	ENSBTAT00000021764	ENSNLET00000022680	ENSGGOT00000000229	ENSCPOT00000025319	ENSDORT00000014216	ENSMMUT00000001608	ENSCJAT00000034380	ENSMUST00000073837	ENSMICT00000009560	ENSPPYT00000005363	ENSOPRT00000015978	ENSOCUT00000013205	ENSRNOT00000049998	ENSSTOT00000009150	ENSTBET00000015840	ENSTSYT00000013273
ENST00000271324	ENSOGAT00000016717	ENSPTRT00000002043	ENSBTAT00000008479	ENSNLET00000004661	ENSGGOT00000005495	ENSCPOT00000010374	ENSDORT00000016196	ENSMMUT00000001175	ENSCJAT00000055217	ENSMUST00000038845	ENSMICT00000011299	ENSPPYT00000001252	ENSOPRT00000002994	ENSOCUT00000014419	ENSRNOT00000024344	ENSSTOT00000006857	ENSTBET00000001203	ENSTSYT00000008627
ENST00000263985	ENSOGAT00000014182	ENSPTRT00000030700	ENSBTAT00000053094	ENSNLET00000007437	ENSGGOT00000011141	ENSCPOT00000005077	ENSDORT00000010241	ENSMMUT00000015443	ENSCJAT00000004090	ENSMUST00000127348	ENSMICT00000004313	ENSPPYT00000017580	ENSOPRT00000011498	ENSOCUT00000016874	ENSRNOT00000057128	ENSSTOT00000005701	ENSTBET00000009810	ENSTSYT00000009976
ENST00000298048	ENSOGAT00000008986	ENSPTRT00000038734	ENSBTAT00000028900	ENSNLET00000004820	ENSGGOT00000014805	ENSCPOT00000014736	ENSDORT00000015637	ENSMMUT00000005354	ENSCJAT00000055908	ENSMUST00000045607	ENSMICT00000017678	ENSPPYT00000022227	ENSOPRT00000000722	ENSOCUT00000001041	ENSRNOT00000018650	ENSSTOT00000005124	ENSTBET00000003391	ENSTSYT00000003211
ENST00000299432	ENSOGAT00000002175	ENSPTRT00000004946	ENSBTAT00000026928	ENSNLET00000021258	ENSGGOT00000031704	ENSCPOT00000019614	ENSDORT00000003240	ENSMMUT00000022051	ENSCJAT00000055650	ENSMUST00000022353	ENSMICT00000013227	ENSPPYT00000002805	ENSOPRT00000001159	ENSOCUT00000005856	ENSRNOT00000010178	ENSSTOT00000007019	ENSTBET00000003478	ENSTSYT00000010790
ENST00000272444	ENSOGAT00000006001	ENSPTRT00000022447	ENSBTAT00000047256	ENSNLET00000000390	ENSGGOT00000010164	ENSCPOT00000025999	ENSDORT00000012506	ENSMMUT00000030309	ENSCJAT00000064977	ENSMUST00000032071	ENSMICT00000006110	ENSPPYT00000014264	ENSOPRT00000014603	ENSOCUT00000003648	ENSRNOT00000036247	ENSSTOT00000010722	ENSTBET00000014589	ENSTSYT00000001787
ENST00000253794	ENSOGAT00000009914	ENSPTRT00000016956	ENSBTAT00000026518	ENSNLET00000015068	ENSGGOT00000016095	ENSCPOT00000012658	ENSDORT00000000950	ENSMMUT00000003035	ENSCJAT00000025806	ENSMUST00000042477	ENSMICT00000010350	ENSPPYT00000009797	ENSOPRT00000012568	ENSOCUT00000015916	ENSRNOT00000043754	ENSSTOT00000003796	ENSTBET00000017166	ENSTSYT00000009993
ENST00000374343	ENSOGAT00000028904	ENSPTRT00000057136	ENSBTAT00000003890	ENSNLET00000011605	ENSGGOT00000007884	ENSCPOT00000012739	ENSDORT00000000638	ENSMMUT00000025304	ENSCJAT00000036578	ENSMUST00000030628	ENSMICT00000016258	ENSPPYT00000002035	ENSOPRT00000001212	ENSOCUT00000001826	ENSRNOT00000032623	ENSSTOT00000013649	ENSTBET00000008865	ENSTSYT00000006951
ENST00000534025	ENSOGAT00000014016	ENSPTRT00000006330	ENSBTAT00000039850	ENSNLET00000022378	ENSGGOT00000007841	ENSCPOT00000011216	ENSDORT00000013307	ENSMMUT00000002828	ENSCJAT00000022689	ENSMUST00000033333	ENSMICT00000005296	ENSPPYT00000004172	ENSOPRT00000004803	ENSOCUT00000003148	ENSRNOT00000018295	ENSSTOT00000002497	ENSTBET00000004931	ENSTSYT00000014085
ENST00000374399	ENSOGAT00000011038	ENSPTRT00000000685	ENSBTAT00000017344	ENSNLET00000011442	ENSGGOT00000014912	ENSCPOT00000008945	ENSDORT00000015285	ENSMMUT00000006326	ENSCJAT00000040979	ENSMUST00000102549	ENSMICT00000000547	ENSPPYT00000002049	ENSOPRT00000003981	ENSOCUT00000000381	ENSRNOT00000068004	ENSSTOT00000002874	ENSTBET00000015180	ENSTSYT00000012506
ENST00000393274	ENSOGAT00000007550	ENSPTRT00000065874	ENSBTAT00000033639	ENSNLET00000006097	ENSGGOT00000008545	ENSCPOT00000013069	ENSDORT00000014225	ENSMMUT00000046309	ENSCJAT00000024181	ENSMUST00000007523	ENSMICT00000013768	ENSPPYT00000001206	ENSOPRT00000004949	ENSOCUT00000016792	ENSRNOT00000025365	ENSSTOT00000014767	ENSTBET00000002359	ENSTSYT00000011729
ENST00000474844	ENSOGAT00000014078	ENSPTRT00000001323	ENSBTAT00000021120	ENSNLET00000021040	ENSGGOT00000032710	ENSCPOT00000006850	ENSDORT00000006843	ENSMMUT00000003867	ENSCJAT00000057874	ENSMUST00000030475	ENSMICT00000004910	ENSPPYT00000001673	ENSOPRT00000010533	ENSOCUT00000007597	ENSRNOT00000016714	ENSSTOT00000009791	ENSTBET00000012230	ENSTSYT00000000087
ENST00000270066	ENSOGAT00000012695	ENSPTRT00000020623	ENSBTAT00000015684	ENSNLET00000002213	ENSGGOT00000000430	ENSCPOT00000000925	ENSDORT00000005080	ENSMMUT00000020400	ENSCJAT00000026140	ENSMUST00000002280	ENSMICT00000007803	ENSPPYT00000011711	ENSOPRT00000004293	ENSOCUT00000002786	ENSRNOT00000026669	ENSSTOT00000009981	ENSTBET00000017363	ENSTSYT00000000377
ENST00000304698	ENSOGAT00000002187	ENSPTRT00000023580	ENSBTAT00000002209	ENSNLET00000008271	ENSGGOT00000024130	ENSCPOT00000020006	ENSDORT00000002530	ENSMMUT00000014565	ENSCJAT00000011405	ENSMUST00000051454	ENSMICT00000005190	ENSPPYT00000015121	ENSOPRT00000011352	ENSOCUT00000001825	ENSRNOT00000006504	ENSSTOT00000004757	ENSTBET00000015947	ENSTSYT00000002279
ENST00000314471	ENSOGAT00000033665	ENSPTRT00000020312	ENSBTAT00000006301	ENSNLET00000018458	ENSGGOT00000022187	ENSCPOT00000005995	ENSDORT00000013482	ENSMMUT00000033494	ENSCJAT00000027394	ENSMUST00000040531	ENSMICT00000006079	ENSPPYT00000011588	ENSOPRT00000004799	ENSOCUT00000000343	ENSRNOT00000026839	ENSSTOT00000003392	ENSTBET00000003887	ENSTSYT00000003913
ENST00000394585	ENSOGAT00000026684	ENSPTRT00000011823	ENSBTAT00000026406	ENSNLET00000018452	ENSGGOT00000008945	ENSCPOT00000011676	ENSDORT00000011326	ENSMMUT00000039800	ENSCJAT00000007323	ENSMUST00000062804	ENSMICT00000014108	ENSPPYT00000006985	ENSOPRT00000009373	ENSOCUT00000004666	ENSRNOT00000011220	ENSSTOT00000016506	ENSTBET00000015829	ENSTSYT00000000574
ENST00000306065	ENSOGAT00000015120	ENSPTRT00000019978	ENSBTAT00000055233	ENSNLET00000009723	ENSGGOT00000008640	ENSCPOT00000000161	ENSDORT00000001833	ENSMMUT00000011250	ENSCJAT00000036098	ENSMUST00000040844	ENSMICT00000000423	ENSPPYT00000011423	ENSOPRT00000016662	ENSOCUT00000013234	ENSRNOT00000017669	ENSSTOT00000013624	ENSTBET00000010111	ENSTSYT00000009855
ENST00000276654	ENSOGAT00000034261	ENSPTRT00000037927	ENSBTAT00000015304	ENSNLET00000004992	ENSGGOT00000011968	ENSCPOT00000025359	ENSDORT00000013058	ENSMMUT00000032069	ENSCJAT00000013049	ENSMUST00000022916	ENSMICT00000013467	ENSPPYT00000021949	ENSOPRT00000006365	ENSOCUT00000002247	ENSRNOT00000005668	ENSSTOT00000013072	ENSTBET00000006731	ENSTSYT00000000528
ENST00000389617	ENSOGAT00000006648	ENSPTRT00000028267	ENSBTAT00000063772	ENSNLET00000003549	ENSGGOT00000005081	ENSCPOT00000000446	ENSDORT00000010919	ENSMMUT00000031656	ENSCJAT00000029502	ENSMUST00000032157	ENSMICT00000006326	ENSPPYT00000015950	ENSOPRT00000013976	ENSOCUT00000016701	ENSRNOT00000007419	ENSSTOT00000010062	ENSTBET00000017444	ENSTSYT00000004235
ENST00000400017	ENSOGAT00000012898	ENSPTRT00000065707	ENSBTAT00000054087	ENSNLET00000018359	ENSGGOT00000010868	ENSCPOT00000002560	ENSDORT00000016508	ENSMMUT00000030329	ENSCJAT00000018100	ENSMUST00000111603	ENSMICT00000007700	ENSPPYT00000006617	ENSOPRT00000016083	ENSOCUT00000010796	ENSRNOT00000060495	ENSSTOT00000001822	ENSTBET00000011676	ENSTSYT00000011892
ENST00000303296	ENSOGAT00000008861	ENSPTRT00000006565	ENSBTAT00000039686	ENSNLET00000022853	ENSGGOT00000021992	ENSCPOT00000002105	ENSDORT00000008652	ENSMMUT00000012515	ENSCJAT00000058819	ENSMUST00000111125	ENSMICT00000011404	ENSPPYT00000004023	ENSOPRT00000014648	ENSOCUT00000001214	ENSRNOT00000067279	ENSSTOT00000009723	ENSTBET00000000765	ENSTSYT00000009421
ENST00000278520	ENSOGAT00000007292	ENSPTRT00000007831	ENSBTAT00000008639	ENSNLET00000009115	ENSGGOT00000016805	ENSCPOT00000026921	ENSDORT00000016570	ENSMMUT00000011187	ENSCJAT00000008363	ENSMUST00000110583	ENSMICT00000009409	ENSPPYT00000004515	ENSOPRT00000010078	ENSOCUT00000007312	ENSRNOT00000037457	ENSSTOT00000002307	ENSTBET00000011804	ENSTSYT00000000216
ENST00000324607	ENSOGAT00000010768	ENSPTRT00000044506	ENSBTAT00000021975	ENSNLET00000002190	ENSGGOT00000014436	ENSCPOT00000008034	ENSDORT00000004720	ENSMMUT00000024505	ENSCJAT00000038311	ENSMUST00000047311	ENSMICT00000007348	ENSPPYT00000018917	ENSOPRT00000008543	ENSOCUT00000010358	ENSRNOT00000059774	ENSSTOT00000008429	ENSTBET00000010414	ENSTSYT00000004011
ENST00000374692	ENSOGAT00000028418	ENSPTRT00000039245	ENSBTAT00000005404	ENSNLET00000022072	ENSGGOT00000007241	ENSCPOT00000007938	ENSDORT00000011776	ENSMMUT00000003805	ENSCJAT00000032814	ENSMUST00000030127	ENSMICT00000001722	ENSPPYT00000022710	ENSOPRT00000016070	ENSOCUT00000005810	ENSRNOT00000034570	ENSSTOT00000003015	ENSTBET00000017657	ENSTSYT00000007549
ENST00000267103	ENSOGAT00000009475	ENSPTRT00000009228	ENSBTAT00000026875	ENSNLET00000022577	ENSGGOT00000015847	ENSCPOT00000025682	ENSDORT00000015335	ENSMMUT00000031004	ENSCJAT00000036240	ENSMUST00000113682	ENSMICT00000014272	ENSPPYT00000005423	ENSOPRT00000015660	ENSOCUT00000011282	ENSRNOT00000017942	ENSSTOT00000004326	ENSTBET00000016466	ENSTSYT00000000731
ENST00000380003	ENSOGAT00000002211	ENSPTRT00000038557	ENSBTAT00000013268	ENSNLET00000006027	ENSGGOT00000028038	ENSCPOT00000009559	ENSDORT00000006734	ENSMMUT00000023188	ENSCJAT00000058239	ENSMUST00000108127	ENSMICT00000004861	ENSPPYT00000022329	ENSOPRT00000006850	ENSOCUT00000006511	ENSRNOT00000013181	ENSSTOT00000010691	ENSTBET00000013504	ENSTSYT00000000452
ENST00000371626	ENSOGAT00000003800	ENSPTRT00000001390	ENSBTAT00000014645	ENSNLET00000020453	ENSGGOT00000010244	ENSCPOT00000004044	ENSDORT00000005108	ENSMMUT00000004647	ENSCJAT00000042430	ENSMUST00000030296	ENSMICT00000002787	ENSPPYT00000001623	ENSOPRT00000013314	ENSOCUT00000001970	ENSRNOT00000010700	ENSSTOT00000006088	ENSTBET00000013189	ENSTSYT00000013628
ENST00000487273	ENSOGAT00000015353	ENSPTRT00000037386	ENSBTAT00000023197	ENSNLET00000015819	ENSGGOT00000006089	ENSCPOT00000007829	ENSDORT00000001530	ENSMMUT00000008433	ENSCJAT00000004398	ENSMUST00000084032	ENSMICT00000003597	ENSPPYT00000021611	ENSOPRT00000004406	ENSOCUT00000013388	ENSRNOT00000041330	ENSSTOT00000015050	ENSTBET00000017471	ENSTSYT00000013714
ENST00000374264	ENSOGAT00000003410	ENSPTRT00000046426	ENSBTAT00000043830	ENSNLET00000020966	ENSGGOT00000012676	ENSCPOT00000006097	ENSDORT00000011941	ENSMMUT00000029670	ENSCJAT00000034442	ENSMUST00000040166	ENSMICT00000009779	ENSPPYT00000022747	ENSOPRT00000003471	ENSOCUT00000010438	ENSRNOT00000059493	ENSSTOT00000005582	ENSTBET00000003050	ENSTSYT00000014378
ENST00000284770	ENSOGAT00000010949	ENSPTRT00000030918	ENSBTAT00000022835	ENSNLET00000014395	ENSGGOT00000001954	ENSCPOT00000004519	ENSDORT00000008196	ENSMMUT00000021606	ENSCJAT00000011657	ENSMUST00000034053	ENSMICT00000010914	ENSPPYT00000017730	ENSOPRT00000008964	ENSOCUT00000005808	ENSRNOT00000017054	ENSSTOT00000014195	ENSTBET00000000533	ENSTSYT00000004661
ENST00000336216	ENSOGAT00000006632	ENSPTRT00000013511	ENSBTAT00000027243	ENSNLET00000010703	ENSGGOT00000008478	ENSCPOT00000011817	ENSDORT00000000623	ENSMMUT00000010387	ENSCJAT00000021722	ENSMUST00000034879	ENSMICT00000011023	ENSPPYT00000007891	ENSOPRT00000010994	ENSOCUT00000005649	ENSRNOT00000022976	ENSSTOT00000013819	ENSTBET00000015280	ENSTSYT00000003481
ENST00000452770	ENSOGAT00000008804	ENSPTRT00000027791	ENSBTAT00000026436	ENSNLET00000008026	ENSGGOT00000003702	ENSCPOT00000002193	ENSDORT00000005287	ENSMMUT00000004723	ENSCJAT00000004539	ENSMUST00000084952	ENSMICT00000017656	ENSPPYT00000016193	ENSOPRT00000000824	ENSOCUT00000003126	ENSRNOT00000028364	ENSSTOT00000014859	ENSTBET00000016728	ENSTSYT00000010865
ENST00000519846	ENSOGAT00000007031	ENSPTRT00000037574	ENSBTAT00000061465	ENSNLET00000002781	ENSGGOT00000007658	ENSCPOT00000007414	ENSDORT00000016281	ENSMMUT00000012258	ENSCJAT00000017658	ENSMUST00000038841	ENSMICT00000006966	ENSPPYT00000021715	ENSOPRT00000002629	ENSOCUT00000005336	ENSRNOT00000009744	ENSSTOT00000012317	ENSTBET00000012107	ENSTSYT00000003862
ENST00000311027	ENSOGAT00000012424	ENSPTRT00000045254	ENSBTAT00000028927	ENSNLET00000018540	ENSGGOT00000008632	ENSCPOT00000013495	ENSDORT00000007749	ENSMMUT00000021884	ENSCJAT00000058029	ENSMUST00000099723	ENSMICT00000007082	ENSPPYT00000003942	ENSOPRT00000009547	ENSOCUT00000002105	ENSRNOT00000041505	ENSSTOT00000007670	ENSTBET00000000585	ENSTSYT00000001556
ENST00000393658	ENSOGAT00000001625	ENSPTRT00000030857	ENSBTAT00000005543	ENSNLET00000021369	ENSGGOT00000002644	ENSCPOT00000002331	ENSDORT00000011732	ENSMMUT00000041486	ENSCJAT00000010239	ENSMUST00000033915	ENSMICT00000003034	ENSPPYT00000017685	ENSOPRT00000009396	ENSOCUT00000007631	ENSRNOT00000014312	ENSSTOT00000002117	ENSTBET00000013952	ENSTSYT00000002875
ENST00000264198	ENSOGAT00000013122	ENSPTRT00000000569	ENSBTAT00000027782	ENSNLET00000010114	ENSGGOT00000006717	ENSCPOT00000014309	ENSDORT00000014991	ENSMMUT00000009650	ENSCJAT00000012711	ENSMUST00000044058	ENSMICT00000010566	ENSPPYT00000002118	ENSOPRT00000007354	ENSOCUT00000000626	ENSRNOT00000021833	ENSSTOT00000015670	ENSTBET00000013480	ENSTSYT00000010779
ENST00000370801	ENSOGAT00000014215	ENSPTRT00000001667	ENSBTAT00000043918	ENSNLET00000012369	ENSGGOT00000009285	ENSCPOT00000003178	ENSDORT00000010114	ENSMMUT00000028446	ENSCJAT00000013519	ENSMUST00000106100	ENSMICT00000004494	ENSPPYT00000001466	ENSOPRT00000013038	ENSOCUT00000002167	ENSRNOT00000068660	ENSSTOT00000016460	ENSTBET00000012842	ENSTSYT00000001813
ENST00000268483	ENSOGAT00000008659	ENSPTRT00000015376	ENSBTAT00000008329	ENSNLET00000007231	ENSGGOT00000013092	ENSCPOT00000014103	ENSDORT00000005221	ENSMMUT00000000971	ENSCJAT00000029399	ENSMUST00000034159	ENSMICT00000011391	ENSPPYT00000008834	ENSOPRT00000001860	ENSOCUT00000009060	ENSRNOT00000034676	ENSSTOT00000000528	ENSTBET00000001048	ENSTSYT00000013924
ENST00000314011	ENSOGAT00000009184	ENSPTRT00000044808	ENSBTAT00000003359	ENSNLET00000010824	ENSGGOT00000011315	ENSCPOT00000011502	ENSDORT00000007436	ENSMMUT00000015352	ENSCJAT00000010621	ENSMUST00000069195	ENSMICT00000005869	ENSPPYT00000002080	ENSOPRT00000012401	ENSOCUT00000010676	ENSRNOT00000037552	ENSSTOT00000001211	ENSTBET00000002808	ENSTSYT00000003140
ENST00000404459	ENSOGAT00000006429	ENSPTRT00000057644	ENSBTAT00000034704	ENSNLET00000015564	ENSGGOT00000003291	ENSCPOT00000007424	ENSDORT00000015420	ENSMMUT00000000692	ENSCJAT00000010501	ENSMUST00000025682	ENSMICT00000006496	ENSPPYT00000002933	ENSOPRT00000014957	ENSOCUT00000012494	ENSRNOT00000026242	ENSSTOT00000007835	ENSTBET00000007915	ENSTSYT00000003327
ENST00000306858	ENSOGAT00000013017	ENSPTRT00000033819	ENSBTAT00000021370	ENSNLET00000004224	ENSGGOT00000002228	ENSCPOT00000010384	ENSDORT00000015256	ENSMMUT00000040460	ENSCJAT00000021195	ENSMUST00000098546	ENSMICT00000007357	ENSPPYT00000019456	ENSOPRT00000002921	ENSOCUT00000003822	ENSRNOT00000015947	ENSSTOT00000004733	ENSTBET00000011440	ENSTSYT00000013500
ENST00000299563	ENSOGAT00000015617	ENSPTRT00000007583	ENSBTAT00000000969	ENSNLET00000021652	ENSGGOT00000014546	ENSCPOT00000022116	ENSDORT00000009673	ENSMMUT00000029583	ENSCJAT00000030379	ENSMUST00000080817	ENSMICT00000011144	ENSPPYT00000004376	ENSOPRT00000013895	ENSOCUT00000001788	ENSRNOT00000029629	ENSSTOT00000000462	ENSTBET00000003669	ENSTSYT00000002489
ENST00000264711	ENSOGAT00000015173	ENSPTRT00000021824	ENSBTAT00000024278	ENSNLET00000001384	ENSGGOT00000004102	ENSCPOT00000006912	ENSDORT00000015134	ENSMMUT00000043712	ENSCJAT00000015789	ENSMUST00000020986	ENSMICT00000001598	ENSPPYT00000014632	ENSOPRT00000008648	ENSOCUT00000011256	ENSRNOT00000005317	ENSSTOT00000006077	ENSTBET00000005453	ENSTSYT00000014097
ENST00000295756	ENSOGAT00000002591	ENSPTRT00000028415	ENSBTAT00000034536	ENSNLET00000005019	ENSGGOT00000005796	ENSCPOT00000004733	ENSDORT00000004116	ENSMMUT00000003902	ENSCJAT00000039564	ENSMUST00000170861	ENSMICT00000001125	ENSPPYT00000015777	ENSOPRT00000009420	ENSOCUT00000002916	ENSRNOT00000059924	ENSSTOT00000008271	ENSTBET00000007901	ENSTSYT00000001605
ENST00000551662	ENSOGAT00000004863	ENSPTRT00000009890	ENSBTAT00000043505	ENSNLET00000008471	ENSGGOT00000013203	ENSCPOT00000010305	ENSDORT00000004757	ENSMMUT00000013294	ENSCJAT00000029092	ENSMUST00000020500	ENSMICT00000014460	ENSPPYT00000005807	ENSOPRT00000009186	ENSOCUT00000005044	ENSRNOT00000010986	ENSSTOT00000014336	ENSTBET00000016848	ENSTSYT00000003430
ENST00000328046	ENSOGAT00000033278	ENSPTRT00000051043	ENSBTAT00000027396	ENSNLET00000012829	ENSGGOT00000034466	ENSCPOT00000000664	ENSDORT00000015614	ENSMMUT00000019437	ENSCJAT00000009457	ENSMUST00000170594	ENSMICT00000008752	ENSPPYT00000023553	ENSOPRT00000009476	ENSOCUT00000006666	ENSRNOT00000008558	ENSSTOT00000002203	ENSTBET00000016188	ENSTSYT00000012676
ENST00000506684	ENSOGAT00000026084	ENSPTRT00000031975	ENSBTAT00000035270	ENSNLET00000011083	ENSGGOT00000004309	ENSCPOT00000000476	ENSDORT00000007282	ENSMMUT00000032284	ENSCJAT00000012126	ENSMUST00000012426	ENSMICT00000001657	ENSPPYT00000018399	ENSOPRT00000002745	ENSOCUT00000005193	ENSRNOT00000064574	ENSSTOT00000007254	ENSTBET00000017414	ENSTSYT00000002321
ENST00000458013	ENSOGAT00000005187	ENSPTRT00000002400	ENSBTAT00000033845	ENSNLET00000013104	ENSGGOT00000041166	ENSCPOT00000007944	ENSDORT00000014173	ENSMMUT00000023771	ENSCJAT00000015317	ENSMUST00000107283	ENSMICT00000010629	ENSPPYT00000001038	ENSOPRT00000011109	ENSOCUT00000007427	ENSRNOT00000028389	ENSSTOT00000005293	ENSTBET00000009999	ENSTSYT00000008060
ENST00000306049	ENSOGAT00000001191	ENSPTRT00000016532	ENSBTAT00000024467	ENSNLET00000007464	ENSGGOT00000030878	ENSCPOT00000012024	ENSDORT00000000351	ENSMMUT00000012695	ENSCJAT00000027610	ENSMUST00000050207	ENSMICT00000009945	ENSPPYT00000009524	ENSOPRT00000017275	ENSOCUT00000015254	ENSRNOT00000005325	ENSSTOT00000012363	ENSTBET00000000830	ENSTSYT00000009882
ENST00000528494	ENSOGAT00000004554	ENSPTRT00000006680	ENSBTAT00000023036	ENSNLET00000017977	ENSGGOT00000005717	ENSCPOT00000013168	ENSDORT00000000382	ENSMMUT00000027634	ENSCJAT00000038945	ENSMUST00000076803	ENSMICT00000006906	ENSPPYT00000003959	ENSOPRT00000009176	ENSOCUT00000016151	ENSRNOT00000022841	ENSSTOT00000005089	ENSTBET00000002574	ENSTSYT00000007482
ENST00000367833	ENSOGAT00000004458	ENSPTRT00000058550	ENSBTAT00000007093	ENSNLET00000019036	ENSGGOT00000013041	ENSCPOT00000010035	ENSDORT00000012123	ENSMMUT00000029776	ENSCJAT00000032811	ENSMUST00000043235	ENSMICT00000007807	ENSPPYT00000000658	ENSOPRT00000011302	ENSOCUT00000014551	ENSRNOT00000004083	ENSSTOT00000014922	ENSTBET00000017072	ENSTSYT00000002102
ENST00000539406	ENSOGAT00000005421	ENSPTRT00000043671	ENSBTAT00000027880	ENSNLET00000019248	ENSGGOT00000012762	ENSCPOT00000013668	ENSDORT00000003980	ENSMMUT00000011650	ENSCJAT00000016439	ENSMUST00000046856	ENSMICT00000008146	ENSPPYT00000020606	ENSOPRT00000015284	ENSOCUT00000009400	ENSRNOT00000064524	ENSSTOT00000002344	ENSTBET00000017338	ENSTSYT00000002138
ENST00000392329	ENSOGAT00000016799	ENSPTRT00000023618	ENSBTAT00000009977	ENSNLET00000008487	ENSGGOT00000015660	ENSCPOT00000002129	ENSDORT00000008929	ENSMMUT00000010181	ENSCJAT00000062763	ENSMUST00000027271	ENSMICT00000010492	ENSPPYT00000015138	ENSOPRT00000004476	ENSOCUT00000000007	ENSRNOT00000016842	ENSSTOT00000001358	ENSTBET00000004313	ENSTSYT00000011213
ENST00000270642	ENSOGAT00000026411	ENSPTRT00000021169	ENSBTAT00000025938	ENSNLET00000007453	ENSGGOT00000011287	ENSCPOT00000025352	ENSDORT00000004286	ENSMMUT00000033099	ENSCJAT00000036422	ENSMUST00000107974	ENSMICT00000014804	ENSPPYT00000011994	ENSOPRT00000001193	ENSOCUT00000002206	ENSRNOT00000024150	ENSSTOT00000008052	ENSTBET00000003119	ENSTSYT00000000244
ENST00000246070	ENSOGAT00000007278	ENSPTRT00000024621	ENSBTAT00000010487	ENSNLET00000018332	ENSGGOT00000013433	ENSCPOT00000007454	ENSDORT00000015283	ENSMMUT00000004633	ENSCJAT00000041967	ENSMUST00000057503	ENSMICT00000006604	ENSPPYT00000012435	ENSOPRT00000008287	ENSOCUT00000010763	ENSRNOT00000007274	ENSSTOT00000000437	ENSTBET00000002419	ENSTSYT00000003567
ENST00000414202	ENSOGAT00000012019	ENSPTRT00000038413	ENSBTAT00000001692	ENSNLET00000011602	ENSGGOT00000004577	ENSCPOT00000013557	ENSDORT00000008572	ENSMMUT00000012605	ENSCJAT00000022393	ENSMUST00000043610	ENSMICT00000016313	ENSPPYT00000022421	ENSOPRT00000012690	ENSOCUT00000010109	ENSRNOT00000021783	ENSSTOT00000000321	ENSTBET00000002383	ENSTSYT00000012114
ENST00000429377	ENSOGAT00000033405	ENSPTRT00000006361	ENSBTAT00000055320	ENSNLET00000022497	ENSGGOT00000013029	ENSCPOT00000009119	ENSDORT00000014967	ENSMMUT00000016010	ENSCJAT00000021020	ENSMUST00000162415	ENSMICT00000017460	ENSPPYT00000004155	ENSOPRT00000006310	ENSOCUT00000013332	ENSRNOT00000064535	ENSSTOT00000013609	ENSTBET00000000555	ENSTSYT00000000600
ENST00000275764	ENSOGAT00000000999	ENSPTRT00000036551	ENSBTAT00000001772	ENSNLET00000016982	ENSGGOT00000013002	ENSCPOT00000013989	ENSDORT00000000574	ENSMMUT00000003282	ENSCJAT00000024258	ENSMUST00000114999	ENSMICT00000001660	ENSPPYT00000021020	ENSOPRT00000002092	ENSOCUT00000001063	ENSRNOT00000066626	ENSSTOT00000004800	ENSTBET00000007495	ENSTSYT00000009662
ENST00000276590	ENSOGAT00000013512	ENSPTRT00000037657	ENSBTAT00000002364	ENSNLET00000014547	ENSGGOT00000000404	ENSCPOT00000007677	ENSDORT00000003242	ENSMMUT00000017546	ENSCJAT00000015397	ENSMUST00000027071	ENSMICT00000012360	ENSPPYT00000021772	ENSOPRT00000011187	ENSOCUT00000008223	ENSRNOT00000010369	ENSSTOT00000005563	ENSTBET00000006881	ENSTSYT00000005876
ENST00000352210	ENSOGAT00000010057	ENSPTRT00000002874	ENSBTAT00000061124	ENSNLET00000017961	ENSGGOT00000030478	ENSCPOT00000015472	ENSDORT00000001694	ENSMMUT00000031845	ENSCJAT00000010136	ENSMUST00000073120	ENSMICT00000007256	ENSPPYT00000000737	ENSOPRT00000003280	ENSOCUT00000002401	ENSRNOT00000004896	ENSSTOT00000006439	ENSTBET00000016680	ENSTSYT00000000535
ENST00000523189	ENSOGAT00000013614	ENSPTRT00000055368	ENSBTAT00000036842	ENSNLET00000000380	ENSGGOT00000010430	ENSCPOT00000005689	ENSDORT00000001420	ENSMMUT00000040694	ENSCJAT00000038060	ENSMUST00000169831	ENSMICT00000008831	ENSPPYT00000018666	ENSOPRT00000015339	ENSOCUT00000011110	ENSRNOT00000059895	ENSSTOT00000014714	ENSTBET00000007941	ENSTSYT00000009715
ENST00000396652	ENSOGAT00000007593	ENSPTRT00000066427	ENSBTAT00000007076	ENSNLET00000020874	ENSGGOT00000009854	ENSCPOT00000014986	ENSDORT00000008225	ENSMMUT00000023630	ENSCJAT00000001713	ENSMUST00000041407	ENSMICT00000015707	ENSPPYT00000020701	ENSOPRT00000010585	ENSOCUT00000012536	ENSRNOT00000008106	ENSSTOT00000003490	ENSTBET00000016405	ENSTSYT00000011207
ENST00000227756	ENSOGAT00000013806	ENSPTRT00000006367	ENSBTAT00000003784	ENSNLET00000022508	ENSGGOT00000005645	ENSCPOT00000006928	ENSDORT00000005507	ENSMMUT00000006746	ENSCJAT00000020668	ENSMUST00000106663	ENSMICT00000017614	ENSPPYT00000004151	ENSOPRT00000009722	ENSOCUT00000015218	ENSRNOT00000022984	ENSSTOT00000016193	ENSTBET00000003149	ENSTSYT00000010003
ENST00000551671	ENSOGAT00000015148	ENSPTRT00000009837	ENSBTAT00000015767	ENSNLET00000007929	ENSGGOT00000010827	ENSCPOT00000025643	ENSDORT00000014770	ENSMMUT00000045757	ENSCJAT00000053692	ENSMUST00000116234	ENSMICT00000015798	ENSPPYT00000005774	ENSOPRT00000001810	ENSOCUT00000002216	ENSRNOT00000007623	ENSSTOT00000016023	ENSTBET00000015543	ENSTSYT00000013256
ENST00000255224	ENSOGAT00000003598	ENSPTRT00000018329	ENSBTAT00000002357	ENSNLET00000013713	ENSGGOT00000006122	ENSCPOT00000010031	ENSDORT00000012270	ENSMMUT00000023921	ENSCJAT00000007735	ENSMUST00000165336	ENSMICT00000015490	ENSPPYT00000010654	ENSOPRT00000003592	ENSOCUT00000009666	ENSRNOT00000023594	ENSSTOT00000009202	ENSTBET00000012492	ENSTSYT00000004445
ENST00000316355	ENSOGAT00000005219	ENSPTRT00000030143	ENSBTAT00000029017	ENSNLET00000010453	ENSGGOT00000033159	ENSCPOT00000013657	ENSDORT00000007154	ENSMMUT00000001308	ENSCJAT00000028814	ENSMUST00000121127	ENSMICT00000011771	ENSPPYT00000017276	ENSOPRT00000008517	ENSOCUT00000008386	ENSRNOT00000002876	ENSSTOT00000007719	ENSTBET00000007657	ENSTSYT00000004194
ENST00000389100	ENSOGAT00000011665	ENSPTRT00000031364	ENSBTAT00000061221	ENSNLET00000004107	ENSGGOT00000006489	ENSCPOT00000009707	ENSDORT00000004059	ENSMMUT00000029721	ENSCJAT00000030443	ENSMUST00000062286	ENSMICT00000013870	ENSPPYT00000018024	ENSOPRT00000004176	ENSOCUT00000010673	ENSRNOT00000066069	ENSSTOT00000012283	ENSTBET00000017449	ENSTSYT00000002782
ENST00000289081	ENSOGAT00000003299	ENSPTRT00000039099	ENSBTAT00000022853	ENSNLET00000021343	ENSGGOT00000006012	ENSCPOT00000014626	ENSDORT00000011345	ENSMMUT00000011608	ENSCJAT00000041938	ENSMUST00000073029	ENSMICT00000001157	ENSPPYT00000022638	ENSOPRT00000015933	ENSOCUT00000010479	ENSRNOT00000022884	ENSSTOT00000006339	ENSTBET00000008538	ENSTSYT00000013701
ENST00000552218	ENSOGAT00000013470	ENSPTRT00000009272	ENSBTAT00000007088	ENSNLET00000022513	ENSGGOT00000008347	ENSCPOT00000007814	ENSDORT00000010630	ENSMMUT00000007296	ENSCJAT00000037038	ENSMUST00000100162	ENSMICT00000008402	ENSPPYT00000005453	ENSOPRT00000015669	ENSOCUT00000013361	ENSRNOT00000065270	ENSSTOT00000014676	ENSTBET00000008720	ENSTSYT00000014348
ENST00000215885	ENSOGAT00000014561	ENSPTRT00000026649	ENSBTAT00000015876	ENSNLET00000005429	ENSGGOT00000027655	ENSCPOT00000012501	ENSDORT00000012304	ENSMMUT00000044082	ENSCJAT00000021168	ENSMUST00000064236	ENSMICT00000008568	ENSPPYT00000013616	ENSOPRT00000002189	ENSOCUT00000004122	ENSRNOT00000029848	ENSSTOT00000002248	ENSTBET00000001878	ENSTSYT00000001371
ENST00000451320	ENSOGAT00000005280	ENSPTRT00000030708	ENSBTAT00000029827	ENSNLET00000007513	ENSGGOT00000008191	ENSCPOT00000010582	ENSDORT00000008171	ENSMMUT00000026003	ENSCJAT00000003948	ENSMUST00000143514	ENSMICT00000009042	ENSPPYT00000017586	ENSOPRT00000009821	ENSOCUT00000014134	ENSRNOT00000014236	ENSSTOT00000006829	ENSTBET00000009347	ENSTSYT00000006281
ENST00000284061	ENSOGAT00000003869	ENSPTRT00000017302	ENSBTAT00000005830	ENSNLET00000010245	ENSGGOT00000011827	ENSCPOT00000011565	ENSDORT00000004137	ENSMMUT00000008122	ENSCJAT00000027065	ENSMUST00000107894	ENSMICT00000000654	ENSPPYT00000009643	ENSOPRT00000006084	ENSOCUT00000012802	ENSRNOT00000003179	ENSSTOT00000014230	ENSTBET00000003111	ENSTSYT00000007564
ENST00000409280	ENSOGAT00000015808	ENSPTRT00000035123	ENSBTAT00000025898	ENSNLET00000019034	ENSGGOT00000016533	ENSCPOT00000013919	ENSDORT00000004102	ENSMMUT00000008004	ENSCJAT00000033588	ENSMUST00000031852	ENSMICT00000000757	ENSPPYT00000020646	ENSOPRT00000004373	ENSOCUT00000009524	ENSRNOT00000014331	ENSSTOT00000008856	ENSTBET00000003982	ENSTSYT00000003779
ENST00000414154	ENSOGAT00000007925	ENSPTRT00000037447	ENSBTAT00000001296	ENSNLET00000016482	ENSGGOT00000008702	ENSCPOT00000002699	ENSDORT00000001075	ENSMMUT00000028402	ENSCJAT00000055689	ENSMUST00000163774	ENSMICT00000015418	ENSPPYT00000021643	ENSOPRT00000010432	ENSOCUT00000003674	ENSRNOT00000036506	ENSSTOT00000002082	ENSTBET00000008600	ENSTSYT00000001342
ENST00000305242	ENSOGAT00000012749	ENSPTRT00000004380	ENSBTAT00000000861	ENSNLET00000021684	ENSGGOT00000014607	ENSCPOT00000009690	ENSDORT00000013131	ENSMMUT00000029062	ENSCJAT00000025384	ENSMUST00000081275	ENSMICT00000002971	ENSPPYT00000002606	ENSOPRT00000012541	ENSOCUT00000007352	ENSRNOT00000025585	ENSSTOT00000006041	ENSTBET00000007848	ENSTSYT00000002153
ENST00000535318	ENSOGAT00000005945	ENSPTRT00000015025	ENSBTAT00000040344	ENSNLET00000003437	ENSGGOT00000003956	ENSCPOT00000021337	ENSDORT00000008976	ENSMMUT00000028620	ENSCJAT00000023763	ENSMUST00000048653	ENSMICT00000012431	ENSPPYT00000008678	ENSOPRT00000006869	ENSOCUT00000000564	ENSRNOT00000063791	ENSSTOT00000010221	ENSTBET00000004877	ENSTSYT00000013291
ENST00000447280	ENSOGAT00000015234	ENSPTRT00000024053	ENSBTAT00000011485	ENSNLET00000012431	ENSGGOT00000000982	ENSCPOT00000002043	ENSDORT00000003160	ENSMMUT00000008621	ENSCJAT00000024097	ENSMUST00000027467	ENSMICT00000014424	ENSPPYT00000015381	ENSOPRT00000016553	ENSOCUT00000004360	ENSRNOT00000020919	ENSSTOT00000009653	ENSTBET00000003707	ENSTSYT00000005448
ENST00000371710	ENSOGAT00000003792	ENSPTRT00000046220	ENSBTAT00000028214	ENSNLET00000020613	ENSGGOT00000004812	ENSCPOT00000010760	ENSDORT00000003127	ENSMMUT00000006692	ENSCJAT00000042423	ENSMUST00000030288	ENSMICT00000002739	ENSPPYT00000001629	ENSOPRT00000013219	ENSOCUT00000004310	ENSRNOT00000068067	ENSSTOT00000000695	ENSTBET00000011543	ENSTSYT00000003994
ENST00000418458	ENSOGAT00000004105	ENSPTRT00000028105	ENSBTAT00000019339	ENSNLET00000009173	ENSGGOT00000011489	ENSCPOT00000000285	ENSDORT00000000673	ENSMMUT00000017554	ENSCJAT00000013417	ENSMUST00000037739	ENSMICT00000016374	ENSPPYT00000016056	ENSOPRT00000009351	ENSOCUT00000009842	ENSRNOT00000033677	ENSSTOT00000000357	ENSTBET00000017524	ENSTSYT00000006850
ENST00000250498	ENSOGAT00000006219	ENSPTRT00000011252	ENSBTAT00000025397	ENSNLET00000018735	ENSGGOT00000023220	ENSCPOT00000000855	ENSDORT00000008172	ENSMMUT00000001196	ENSCJAT00000016915	ENSMUST00000022781	ENSMICT00000012680	ENSPPYT00000006663	ENSOPRT00000000941	ENSOCUT00000014265	ENSRNOT00000012233	ENSSTOT00000007906	ENSTBET00000009670	ENSTSYT00000013303
ENST00000291539	ENSOGAT00000015750	ENSPTRT00000026016	ENSBTAT00000029195	ENSNLET00000018691	ENSGGOT00000014325	ENSCPOT00000014339	ENSDORT00000002149	ENSMMUT00000031101	ENSCJAT00000030668	ENSMUST00000127929	ENSMICT00000016255	ENSPPYT00000013304	ENSOPRT00000002705	ENSOCUT00000004384	ENSRNOT00000001559	ENSSTOT00000001453	ENSTBET00000010376	ENSTSYT00000011569
ENST00000319359	ENSOGAT00000028545	ENSPTRT00000012925	ENSBTAT00000022860	ENSNLET00000006926	ENSGGOT00000013274	ENSCPOT00000008269	ENSDORT00000014427	ENSMMUT00000002385	ENSCJAT00000004952	ENSMUST00000028679	ENSMICT00000000120	ENSPPYT00000007579	ENSOPRT00000000950	ENSOCUT00000001159	ENSRNOT00000036823	ENSSTOT00000012175	ENSTBET00000010008	ENSTSYT00000014524
ENST00000369405	ENSOGAT00000028992	ENSPTRT00000005617	ENSBTAT00000008828	ENSNLET00000020183	ENSGGOT00000025413	ENSCPOT00000006225	ENSDORT00000009662	ENSMMUT00000001635	ENSCJAT00000037216	ENSMUST00000076891	ENSMICT00000007336	ENSPPYT00000003200	ENSOPRT00000004742	ENSOCUT00000003600	ENSRNOT00000054681	ENSSTOT00000013840	ENSTBET00000013684	ENSTSYT00000009672
ENST00000455972	ENSOGAT00000006811	ENSPTRT00000038843	ENSBTAT00000014839	ENSNLET00000000839	ENSGGOT00000011274	ENSCPOT00000003279	ENSDORT00000001566	ENSMMUT00000007600	ENSCJAT00000003190	ENSMUST00000096164	ENSMICT00000002917	ENSPPYT00000022473	ENSOPRT00000000934	ENSOCUT00000013500	ENSRNOT00000029109	ENSSTOT00000012672	ENSTBET00000017419	ENSTSYT00000000978
ENST00000360947	ENSOGAT00000026705	ENSPTRT00000011214	ENSBTAT00000027974	ENSNLET00000018293	ENSGGOT00000028724	ENSCPOT00000019724	ENSDORT00000009814	ENSMMUT00000009061	ENSCJAT00000054979	ENSMUST00000166169	ENSMICT00000016895	ENSPPYT00000006613	ENSOPRT00000004906	ENSOCUT00000009922	ENSRNOT00000015418	ENSSTOT00000014955	ENSTBET00000003392	ENSTSYT00000010320
ENST00000265371	ENSOGAT00000010294	ENSPTRT00000004457	ENSBTAT00000026471	ENSNLET00000023151	ENSGGOT00000001506	ENSCPOT00000009104	ENSDORT00000002432	ENSMMUT00000043022	ENSCJAT00000026837	ENSMUST00000026917	ENSMICT00000008501	ENSPPYT00000002639	ENSOPRT00000012822	ENSOCUT00000004580	ENSRNOT00000014492	ENSSTOT00000013485	ENSTBET00000013685	ENSTSYT00000001736
ENST00000335183	ENSOGAT00000009668	ENSPTRT00000011644	ENSBTAT00000043712	ENSNLET00000016191	ENSGGOT00000005751	ENSCPOT00000004248	ENSDORT00000001492	ENSMMUT00000017267	ENSCJAT00000014312	ENSMUST00000067426	ENSMICT00000016184	ENSPPYT00000006884	ENSOPRT00000005263	ENSOCUT00000003047	ENSRNOT00000012985	ENSSTOT00000011341	ENSTBET00000010206	ENSTSYT00000011244
ENST00000373970	ENSOGAT00000004252	ENSPTRT00000004697	ENSBTAT00000018901	ENSNLET00000015325	ENSGGOT00000002569	ENSCPOT00000025931	ENSDORT00000005722	ENSMMUT00000015172	ENSCJAT00000009668	ENSMUST00000025803	ENSMICT00000012912	ENSPPYT00000002928	ENSOPRT00000002739	ENSOCUT00000011482	ENSRNOT00000015771	ENSSTOT00000013544	ENSTBET00000015068	ENSTSYT00000001031
ENST00000284224	ENSOGAT00000003916	ENSPTRT00000018249	ENSBTAT00000031067	ENSNLET00000008333	ENSGGOT00000022906	ENSCPOT00000010533	ENSDORT00000007817	ENSMMUT00000016532	ENSCJAT00000040767	ENSMUST00000053017	ENSMICT00000002335	ENSPPYT00000010599	ENSOPRT00000009338	ENSOCUT00000004444	ENSRNOT00000021253	ENSSTOT00000013152	ENSTBET00000008224	ENSTSYT00000003131
ENST00000306052	ENSOGAT00000013339	ENSPTRT00000001424	ENSBTAT00000053259	ENSNLET00000022953	ENSGGOT00000004059	ENSCPOT00000011622	ENSDORT00000015656	ENSMMUT00000010655	ENSCJAT00000008963	ENSMUST00000030356	ENSMICT00000006417	ENSPPYT00000001600	ENSOPRT00000009041	ENSOCUT00000016653	ENSRNOT00000031250	ENSSTOT00000001257	ENSTBET00000011833	ENSTSYT00000014693
ENST00000064724	ENSOGAT00000027644	ENSPTRT00000029118	ENSBTAT00000025374	ENSNLET00000006699	ENSGGOT00000026419	ENSCPOT00000011723	ENSDORT00000015448	ENSMMUT00000012960	ENSCJAT00000004688	ENSMUST00000046174	ENSMICT00000017298	ENSPPYT00000016614	ENSOPRT00000015612	ENSOCUT00000005519	ENSRNOT00000014359	ENSSTOT00000008076	ENSTBET00000000259	ENSTSYT00000006758
ENST00000355962	ENSOGAT00000002847	ENSPTRT00000015355	ENSBTAT00000002866	ENSNLET00000007356	ENSGGOT00000004720	ENSCPOT00000008085	ENSDORT00000002832	ENSMMUT00000002859	ENSCJAT00000029184	ENSMUST00000001720	ENSMICT00000001889	ENSPPYT00000008848	ENSOPRT00000001231	ENSOCUT00000009958	ENSRNOT00000022721	ENSSTOT00000003062	ENSTBET00000007778	ENSTSYT00000006038
ENST00000425432	ENSOGAT00000007251	ENSPTRT00000044214	ENSBTAT00000032310	ENSNLET00000007309	ENSGGOT00000005892	ENSCPOT00000005705	ENSDORT00000002402	ENSMMUT00000023486	ENSCJAT00000029299	ENSMUST00000171701	ENSMICT00000011305	ENSPPYT00000008842	ENSOPRT00000001330	ENSOCUT00000009016	ENSRNOT00000000279	ENSSTOT00000015304	ENSTBET00000011532	ENSTSYT00000014686
ENST00000315796	ENSOGAT00000003052	ENSPTRT00000023417	ENSBTAT00000025394	ENSNLET00000007043	ENSGGOT00000003714	ENSCPOT00000004602	ENSDORT00000016597	ENSMMUT00000010220	ENSCJAT00000054183	ENSMUST00000037210	ENSMICT00000006970	ENSPPYT00000015023	ENSOPRT00000015552	ENSOCUT00000003701	ENSRNOT00000002081	ENSSTOT00000012958	ENSTBET00000009199	ENSTSYT00000003314
ENST00000435765	ENSOGAT00000005169	ENSPTRT00000036216	ENSBTAT00000065774	ENSNLET00000011891	ENSGGOT00000026023	ENSCPOT00000021295	ENSDORT00000000212	ENSMMUT00000004856	ENSCJAT00000057595	ENSMUST00000030769	ENSMICT00000008377	ENSPPYT00000020835	ENSOPRT00000005876	ENSOCUT00000010215	ENSRNOT00000016450	ENSSTOT00000005206	ENSTBET00000013910	ENSTSYT00000000666
ENST00000395035	ENSOGAT00000001015	ENSPTRT00000066332	ENSBTAT00000033135	ENSNLET00000020990	ENSGGOT00000009745	ENSCPOT00000007634	ENSDORT00000016682	ENSMMUT00000015387	ENSCJAT00000059244	ENSMUST00000086545	ENSMICT00000007985	ENSPPYT00000014497	ENSOPRT00000010345	ENSOCUT00000001117	ENSRNOT00000061975	ENSSTOT00000013506	ENSTBET00000009258	ENSTSYT00000008850
ENST00000261530	ENSOGAT00000009395	ENSPTRT00000012046	ENSBTAT00000004016	ENSNLET00000021143	ENSGGOT00000005629	ENSCPOT00000007431	ENSDORT00000000794	ENSMMUT00000012290	ENSCJAT00000040682	ENSMUST00000071106	ENSMICT00000006356	ENSPPYT00000007106	ENSOPRT00000002476	ENSOCUT00000004135	ENSRNOT00000013602	ENSSTOT00000015091	ENSTBET00000016319	ENSTSYT00000003397
ENST00000561180	ENSOGAT00000002806	ENSPTRT00000012734	ENSBTAT00000029159	ENSNLET00000014655	ENSGGOT00000002064	ENSCPOT00000002891	ENSDORT00000004495	ENSMMUT00000040839	ENSCJAT00000041222	ENSMUST00000102534	ENSMICT00000017348	ENSPPYT00000007476	ENSOPRT00000004759	ENSOCUT00000001787	ENSRNOT00000007687	ENSSTOT00000009457	ENSTBET00000014356	ENSTSYT00000001566
ENST00000304568	ENSOGAT00000001435	ENSPTRT00000024095	ENSBTAT00000028426	ENSNLET00000017103	ENSGGOT00000000304	ENSCPOT00000014382	ENSDORT00000013696	ENSMMUT00000018595	ENSCJAT00000025216	ENSMUST00000027331	ENSMICT00000013201	ENSPPYT00000015396	ENSOPRT00000012446	ENSOCUT00000000117	ENSRNOT00000020880	ENSSTOT00000008153	ENSTBET00000001871	ENSTSYT00000011679
ENST00000358901	ENSOGAT00000008611	ENSPTRT00000038667	ENSBTAT00000019970	ENSNLET00000006040	ENSGGOT00000003217	ENSCPOT00000012107	ENSDORT00000010299	ENSMMUT00000028405	ENSCJAT00000017525	ENSMUST00000030164	ENSMICT00000005484	ENSPPYT00000022274	ENSOPRT00000010735	ENSOCUT00000006438	ENSRNOT00000046102	ENSSTOT00000015185	ENSTBET00000004663	ENSTSYT00000009654
ENST00000359878	ENSOGAT00000012337	ENSPTRT00000023690	ENSBTAT00000002929	ENSNLET00000008816	ENSGGOT00000031517	ENSCPOT00000012193	ENSDORT00000007818	ENSMMUT00000011326	ENSCJAT00000058521	ENSMUST00000114410	ENSMICT00000003959	ENSPPYT00000015177	ENSOPRT00000001535	ENSOCUT00000011557	ENSRNOT00000029879	ENSSTOT00000007111	ENSTBET00000002969	ENSTSYT00000008434
ENST00000280295	ENSOGAT00000012385	ENSPTRT00000023556	ENSBTAT00000060951	ENSNLET00000008092	ENSGGOT00000028235	ENSCPOT00000001203	ENSDORT00000014390	ENSMMUT00000001655	ENSCJAT00000022253	ENSMUST00000090760	ENSMICT00000001905	ENSPPYT00000015102	ENSOPRT00000012345	ENSOCUT00000010141	ENSRNOT00000034769	ENSSTOT00000000569	ENSTBET00000005851	ENSTSYT00000008464
ENST00000324695	ENSOGAT00000013447	ENSPTRT00000024227	ENSBTAT00000019509	ENSNLET00000010900	ENSGGOT00000008424	ENSCPOT00000005569	ENSDORT00000001002	ENSMMUT00000029867	ENSCJAT00000055219	ENSMUST00000113114	ENSMICT00000014712	ENSPPYT00000015465	ENSOPRT00000001461	ENSOCUT00000002050	ENSRNOT00000025879	ENSSTOT00000014773	ENSTBET00000010108	ENSTSYT00000010727
ENST00000258412	ENSOGAT00000001794	ENSPTRT00000023944	ENSBTAT00000005137	ENSNLET00000010940	ENSGGOT00000006934	ENSCPOT00000001262	ENSDORT00000016111	ENSMMUT00000032112	ENSCJAT00000021184	ENSMUST00000113796	ENSMICT00000006847	ENSPPYT00000015311	ENSOPRT00000005857	ENSOCUT00000005524	ENSRNOT00000019848	ENSSTOT00000015201	ENSTBET00000003861	ENSTSYT00000001178
ENST00000463496	ENSOGAT00000012661	ENSPTRT00000035037	ENSBTAT00000010187	ENSNLET00000021080	ENSGGOT00000022061	ENSCPOT00000005540	ENSDORT00000007751	ENSMMUT00000017091	ENSCJAT00000001876	ENSMUST00000019400	ENSMICT00000011728	ENSPPYT00000020692	ENSOPRT00000010360	ENSOCUT00000003876	ENSRNOT00000006618	ENSSTOT00000003100	ENSTBET00000007209	ENSTSYT00000010838
ENST00000289407	ENSOGAT00000013699	ENSPTRT00000036456	ENSBTAT00000009542	ENSNLET00000016033	ENSGGOT00000025552	ENSCPOT00000024264	ENSDORT00000006517	ENSMMUT00000016954	ENSCJAT00000020037	ENSMUST00000046750	ENSMICT00000015333	ENSPPYT00000020978	ENSOPRT00000009000	ENSOCUT00000013166	ENSRNOT00000010989	ENSSTOT00000011424	ENSTBET00000013707	ENSTSYT00000004914
ENST00000517309	ENSOGAT00000004308	ENSPTRT00000037820	ENSBTAT00000018461	ENSNLET00000004631	ENSGGOT00000028871	ENSCPOT00000003535	ENSDORT00000010478	ENSMMUT00000012316	ENSCJAT00000040374	ENSMUST00000021990	ENSMICT00000009430	ENSPPYT00000021890	ENSOPRT00000008916	ENSOCUT00000004281	ENSRNOT00000006317	ENSSTOT00000008030	ENSTBET00000002775	ENSTSYT00000003021
ENST00000378116	ENSOGAT00000032078	ENSPTRT00000004256	ENSBTAT00000032523	ENSNLET00000015771	ENSGGOT00000005113	ENSCPOT00000009990	ENSDORT00000005909	ENSMMUT00000021435	ENSCJAT00000017746	ENSMUST00000115099	ENSMICT00000002827	ENSPPYT00000002527	ENSOPRT00000003752	ENSOCUT00000006923	ENSRNOT00000022136	ENSSTOT00000011319	ENSTBET00000005983	ENSTSYT00000006560
ENST00000372809	ENSOGAT00000005492	ENSPTRT00000048933	ENSBTAT00000017353	ENSNLET00000015393	ENSGGOT00000015332	ENSCPOT00000013108	ENSDORT00000012841	ENSMMUT00000015754	ENSCJAT00000005231	ENSMUST00000030408	ENSMICT00000016325	ENSPPYT00000001786	ENSOPRT00000012058	ENSOCUT00000001028	ENSRNOT00000019080	ENSSTOT00000005049	ENSTBET00000003241	ENSTSYT00000012058
ENST00000156471	ENSOGAT00000016017	ENSPTRT00000012712	ENSBTAT00000016895	ENSNLET00000014266	ENSGGOT00000014136	ENSCPOT00000000815	ENSDORT00000015155	ENSMMUT00000003312	ENSCJAT00000041357	ENSMUST00000043160	ENSMICT00000016988	ENSPPYT00000007457	ENSOPRT00000016530	ENSOCUT00000003493	ENSRNOT00000011952	ENSSTOT00000000399	ENSTBET00000013955	ENSTSYT00000004312
ENST00000371224	ENSOGAT00000003756	ENSPTRT00000005266	ENSBTAT00000015703	ENSNLET00000016764	ENSGGOT00000012543	ENSCPOT00000000180	ENSDORT00000014100	ENSMMUT00000004389	ENSCJAT00000031525	ENSMUST00000025979	ENSMICT00000015956	ENSPPYT00000003014	ENSOPRT00000003246	ENSOCUT00000005957	ENSRNOT00000020940	ENSSTOT00000007944	ENSTBET00000010175	ENSTSYT00000003677
ENST00000266980	ENSOGAT00000001528	ENSPTRT00000009356	ENSBTAT00000065123	ENSNLET00000022354	ENSGGOT00000009099	ENSCPOT00000025915	ENSDORT00000012942	ENSMMUT00000012126	ENSCJAT00000022259	ENSMUST00000167859	ENSMICT00000014490	ENSPPYT00000005507	ENSOPRT00000001355	ENSOCUT00000007202	ENSRNOT00000045547	ENSSTOT00000015943	ENSTBET00000002218	ENSTSYT00000005513
ENST00000404371	ENSOGAT00000009755	ENSPTRT00000021718	ENSBTAT00000002508	ENSNLET00000017882	ENSGGOT00000002290	ENSCPOT00000010902	ENSDORT00000003594	ENSMMUT00000018049	ENSCJAT00000013255	ENSMUST00000057288	ENSMICT00000017369	ENSPPYT00000014695	ENSOPRT00000001397	ENSOCUT00000012989	ENSRNOT00000060602	ENSSTOT00000005548	ENSTBET00000014670	ENSTSYT00000013891
ENST00000180173	ENSOGAT00000004229	ENSPTRT00000037089	ENSBTAT00000055365	ENSNLET00000003391	ENSGGOT00000010590	ENSCPOT00000011588	ENSDORT00000014881	ENSMMUT00000039130	ENSCJAT00000022834	ENSMUST00000048898	ENSMICT00000002876	ENSPPYT00000021428	ENSOPRT00000000588	ENSOCUT00000008370	ENSRNOT00000015625	ENSSTOT00000009216	ENSTBET00000007158	ENSTSYT00000007734
ENST00000281924	ENSOGAT00000016494	ENSPTRT00000047743	ENSBTAT00000024972	ENSNLET00000010725	ENSGGOT00000011082	ENSCPOT00000004997	ENSDORT00000015004	ENSMMUT00000014784	ENSCJAT00000022435	ENSMUST00000160616	ENSMICT00000000228	ENSPPYT00000014875	ENSOPRT00000009137	ENSOCUT00000011107	ENSRNOT00000005006	ENSSTOT00000004767	ENSTBET00000015000	ENSTSYT00000009656
ENST00000237281	ENSOGAT00000016816	ENSPTRT00000034492	ENSBTAT00000007075	ENSNLET00000019378	ENSGGOT00000005955	ENSCPOT00000013280	ENSDORT00000000627	ENSMMUT00000030864	ENSCJAT00000038021	ENSMUST00000070300	ENSMICT00000004872	ENSPPYT00000019872	ENSOPRT00000016012	ENSOCUT00000010587	ENSRNOT00000019918	ENSSTOT00000003720	ENSTBET00000013730	ENSTSYT00000006275
ENST00000393147	ENSOGAT00000009141	ENSPTRT00000023006	ENSBTAT00000006894	ENSNLET00000015001	ENSGGOT00000011997	ENSCPOT00000004945	ENSDORT00000014626	ENSMMUT00000030129	ENSCJAT00000060012	ENSMUST00000112606	ENSMICT00000008173	ENSPPYT00000014861	ENSOPRT00000012971	ENSOCUT00000008381	ENSRNOT00000003527	ENSSTOT00000014477	ENSTBET00000017407	ENSTSYT00000001842
ENST00000535342	ENSOGAT00000003288	ENSPTRT00000017523	ENSBTAT00000061482	ENSNLET00000018932	ENSGGOT00000015744	ENSCPOT00000003975	ENSDORT00000003440	ENSMMUT00000007927	ENSCJAT00000000582	ENSMUST00000061287	ENSMICT00000013234	ENSPPYT00000009993	ENSOPRT00000015658	ENSOCUT00000008459	ENSRNOT00000037067	ENSSTOT00000007255	ENSTBET00000002216	ENSTSYT00000012123
ENST00000272433	ENSOGAT00000009149	ENSPTRT00000022428	ENSBTAT00000047335	ENSNLET00000013266	ENSGGOT00000005284	ENSCPOT00000025095	ENSDORT00000006952	ENSMMUT00000003258	ENSCJAT00000055287	ENSMUST00000045846	ENSMICT00000011289	ENSPPYT00000014277	ENSOPRT00000015903	ENSOCUT00000000837	ENSRNOT00000057537	ENSSTOT00000002332	ENSTBET00000009372	ENSTSYT00000008072
ENST00000367244	ENSOGAT00000006515	ENSPTRT00000034559	ENSBTAT00000017827	ENSNLET00000019675	ENSGGOT00000027857	ENSCPOT00000003030	ENSDORT00000015940	ENSMMUT00000026801	ENSCJAT00000024860	ENSMUST00000019906	ENSMICT00000007708	ENSPPYT00000019920	ENSOPRT00000016284	ENSOCUT00000000512	ENSRNOT00000025477	ENSSTOT00000007650	ENSTBET00000000167	ENSTSYT00000003607
ENST00000491148	ENSOGAT00000008317	ENSPTRT00000028930	ENSBTAT00000000580	ENSNLET00000003173	ENSGGOT00000005342	ENSCPOT00000001494	ENSDORT00000001783	ENSMMUT00000007770	ENSCJAT00000002851	ENSMUST00000011607	ENSMICT00000005403	ENSPPYT00000016500	ENSOPRT00000004704	ENSOCUT00000003087	ENSRNOT00000068488	ENSSTOT00000008344	ENSTBET00000009780	ENSTSYT00000008699
ENST00000392837	ENSOGAT00000001210	ENSPTRT00000055599	ENSBTAT00000029270	ENSNLET00000008564	ENSGGOT00000015008	ENSCPOT00000005390	ENSDORT00000003964	ENSMMUT00000012910	ENSCJAT00000029379	ENSMUST00000038523	ENSMICT00000008964	ENSPPYT00000005814	ENSOPRT00000008646	ENSOCUT00000006126	ENSRNOT00000036806	ENSSTOT00000010169	ENSTBET00000014907	ENSTSYT00000008568
ENST00000393256	ENSOGAT00000034699	ENSPTRT00000047870	ENSBTAT00000049077	ENSNLET00000002958	ENSGGOT00000012213	ENSCPOT00000002881	ENSDORT00000016518	ENSMMUT00000025898	ENSCJAT00000008553	ENSMUST00000110341	ENSMICT00000008345	ENSPPYT00000014106	ENSOPRT00000003116	ENSOCUT00000007382	ENSRNOT00000051069	ENSSTOT00000001934	ENSTBET00000003463	ENSTSYT00000011548
ENST00000373969	ENSOGAT00000006802	ENSPTRT00000039398	ENSBTAT00000021192	ENSNLET00000021582	ENSGGOT00000005217	ENSCPOT00000006638	ENSDORT00000015055	ENSMMUT00000009236	ENSCJAT00000040016	ENSMUST00000030036	ENSMICT00000003252	ENSPPYT00000022809	ENSOPRT00000017016	ENSOCUT00000001372	ENSRNOT00000007578	ENSSTOT00000005389	ENSTBET00000002125	ENSTSYT00000011054
ENST00000409386	ENSOGAT00000030740	ENSPTRT00000039916	ENSBTAT00000007892	ENSNLET00000011343	ENSGGOT00000000389	ENSCPOT00000021051	ENSDORT00000016086	ENSMMUT00000030260	ENSCJAT00000020719	ENSMUST00000134054	ENSMICT00000006833	ENSPPYT00000023073	ENSOPRT00000005849	ENSOCUT00000002357	ENSRNOT00000024163	ENSSTOT00000015100	ENSTBET00000006635	ENSTSYT00000002276
ENST00000262461	ENSOGAT00000008604	ENSPTRT00000031837	ENSBTAT00000012701	ENSNLET00000015716	ENSGGOT00000016029	ENSCPOT00000014674	ENSDORT00000007301	ENSMMUT00000015688	ENSCJAT00000037713	ENSMUST00000115366	ENSMICT00000013259	ENSPPYT00000018310	ENSOPRT00000002163	ENSOCUT00000001812	ENSRNOT00000021921	ENSSTOT00000003250	ENSTBET00000007435	ENSTSYT00000013188
ENST00000393775	ENSOGAT00000015214	ENSPTRT00000065105	ENSBTAT00000066340	ENSNLET00000006123	ENSGGOT00000041114	ENSCPOT00000003252	ENSDORT00000003343	ENSMMUT00000011293	ENSCJAT00000017730	ENSMUST00000023478	ENSMICT00000000869	ENSPPYT00000015724	ENSOPRT00000007382	ENSOCUT00000017083	ENSRNOT00000066997	ENSSTOT00000003788	ENSTBET00000001997	ENSTSYT00000003230
ENST00000542664	ENSOGAT00000010053	ENSPTRT00000010769	ENSBTAT00000017943	ENSNLET00000003742	ENSGGOT00000007208	ENSCPOT00000003160	ENSDORT00000014000	ENSMMUT00000043322	ENSCJAT00000018146	ENSMUST00000036653	ENSMICT00000006407	ENSPPYT00000006335	ENSOPRT00000011475	ENSOCUT00000009972	ENSRNOT00000013408	ENSSTOT00000002858	ENSTBET00000004572	ENSTSYT00000013071
ENST00000406396	ENSOGAT00000002336	ENSPTRT00000018484	ENSBTAT00000010155	ENSNLET00000011143	ENSGGOT00000013365	ENSCPOT00000014562	ENSDORT00000002042	ENSMMUT00000009209	ENSCJAT00000013036	ENSMUST00000010049	ENSMICT00000001583	ENSPPYT00000010752	ENSOPRT00000013624	ENSOCUT00000014160	ENSRNOT00000003728	ENSSTOT00000007409	ENSTBET00000012330	ENSTSYT00000009477
ENST00000495893	ENSOGAT00000008960	ENSPTRT00000029115	ENSBTAT00000018519	ENSNLET00000006631	ENSGGOT00000002000	ENSCPOT00000005933	ENSDORT00000008567	ENSMMUT00000031219	ENSCJAT00000004908	ENSMUST00000129817	ENSMICT00000001089	ENSPPYT00000016609	ENSOPRT00000005174	ENSOCUT00000013124	ENSRNOT00000012759	ENSSTOT00000002798	ENSTBET00000008447	ENSTSYT00000014475
ENST00000297423	ENSOGAT00000025736	ENSPTRT00000037484	ENSBTAT00000061199	ENSNLET00000016861	ENSGGOT00000007637	ENSCPOT00000005422	ENSDORT00000004414	ENSMMUT00000018449	ENSCJAT00000026622	ENSMUST00000040248	ENSMICT00000015558	ENSPPYT00000021660	ENSOPRT00000010680	ENSOCUT00000001692	ENSRNOT00000057502	ENSSTOT00000010900	ENSTBET00000001057	ENSTSYT00000007291
ENST00000259939	ENSOGAT00000007758	ENSPTRT00000032827	ENSBTAT00000011931	ENSNLET00000002155	ENSGGOT00000008740	ENSCPOT00000014535	ENSDORT00000009555	ENSMMUT00000005522	ENSCJAT00000013591	ENSMUST00000068891	ENSMICT00000015478	ENSPPYT00000018915	ENSOPRT00000007403	ENSOCUT00000000543	ENSRNOT00000036876	ENSSTOT00000001505	ENSTBET00000009351	ENSTSYT00000004732
ENST00000348428	ENSOGAT00000015634	ENSPTRT00000018441	ENSBTAT00000046385	ENSNLET00000010642	ENSGGOT00000030550	ENSCPOT00000012612	ENSDORT00000016658	ENSMMUT00000019975	ENSCJAT00000006865	ENSMUST00000102868	ENSMICT00000007726	ENSPPYT00000010727	ENSOPRT00000010281	ENSOCUT00000007834	ENSRNOT00000068418	ENSSTOT00000009681	ENSTBET00000003549	ENSTSYT00000002018
ENST00000404826	ENSOGAT00000007116	ENSPTRT00000034863	ENSBTAT00000005275	ENSNLET00000019692	ENSGGOT00000012980	ENSCPOT00000015102	ENSDORT00000003868	ENSMMUT00000027717	ENSCJAT00000036718	ENSMUST00000085774	ENSMICT00000010319	ENSPPYT00000020180	ENSOPRT00000004779	ENSOCUT00000001242	ENSRNOT00000031149	ENSSTOT00000002029	ENSTBET00000005193	ENSTSYT00000010213
ENST00000409296	ENSOGAT00000000534	ENSPTRT00000055690	ENSBTAT00000024773	ENSNLET00000004094	ENSGGOT00000003268	ENSCPOT00000009497	ENSDORT00000002721	ENSMMUT00000020965	ENSCJAT00000060007	ENSMUST00000080856	ENSMICT00000016482	ENSPPYT00000018021	ENSOPRT00000012535	ENSOCUT00000004866	ENSRNOT00000060904	ENSSTOT00000006108	ENSTBET00000013358	ENSTSYT00000004964
ENST00000454516	ENSOGAT00000007300	ENSPTRT00000027534	ENSBTAT00000006348	ENSNLET00000005633	ENSGGOT00000024625	ENSCPOT00000006001	ENSDORT00000006397	ENSMMUT00000045291	ENSCJAT00000023096	ENSMUST00000070117	ENSMICT00000005730	ENSPPYT00000016327	ENSOPRT00000004892	ENSOCUT00000011434	ENSRNOT00000013815	ENSSTOT00000015177	ENSTBET00000002335	ENSTSYT00000002566
ENST00000380289	ENSOGAT00000012783	ENSPTRT00000040236	ENSBTAT00000009328	ENSNLET00000012136	ENSGGOT00000016934	ENSCPOT00000000480	ENSDORT00000007873	ENSMMUT00000033341	ENSCJAT00000008439	ENSMUST00000033730	ENSMICT00000011801	ENSPPYT00000023500	ENSOPRT00000000290	ENSOCUT00000012886	ENSRNOT00000005559	ENSSTOT00000000527	ENSTBET00000012002	ENSTSYT00000012434
ENST00000396777	ENSOGAT00000002788	ENSPTRT00000006252	ENSBTAT00000004638	ENSNLET00000022226	ENSGGOT00000005098	ENSCPOT00000006319	ENSDORT00000010669	ENSMMUT00000010822	ENSCJAT00000027353	ENSMUST00000131446	ENSMICT00000002101	ENSPPYT00000004220	ENSOPRT00000007955	ENSOCUT00000016945	ENSRNOT00000025159	ENSSTOT00000015033	ENSTBET00000013552	ENSTSYT00000012606
ENST00000259632	ENSOGAT00000008250	ENSPTRT00000038639	ENSBTAT00000025136	ENSNLET00000006213	ENSGGOT00000002932	ENSCPOT00000001560	ENSDORT00000001715	ENSMMUT00000029236	ENSCJAT00000016973	ENSMUST00000030158	ENSMICT00000002374	ENSPPYT00000023341	ENSOPRT00000009187	ENSOCUT00000000358	ENSRNOT00000019561	ENSSTOT00000002807	ENSTBET00000000616	ENSTSYT00000002019
ENST00000282223	ENSOGAT00000012830	ENSPTRT00000031962	ENSBTAT00000004556	ENSNLET00000010843	ENSGGOT00000000812	ENSCPOT00000000627	ENSDORT00000010027	ENSMMUT00000001902	ENSCJAT00000011771	ENSMUST00000172326	ENSMICT00000000619	ENSPPYT00000018391	ENSOPRT00000001128	ENSOCUT00000009840	ENSRNOT00000061187	ENSSTOT00000007310	ENSTBET00000012969	ENSTSYT00000004138
ENST00000407584	ENSOGAT00000004108	ENSPTRT00000028106	ENSBTAT00000019340	ENSNLET00000009176	ENSGGOT00000011494	ENSCPOT00000000286	ENSDORT00000000676	ENSMMUT00000046388	ENSCJAT00000013545	ENSMUST00000022476	ENSMICT00000016382	ENSPPYT00000016055	ENSOPRT00000009368	ENSOCUT00000009848	ENSRNOT00000013291	ENSSTOT00000000359	ENSTBET00000017620	ENSTSYT00000006852
ENST00000239926	ENSOGAT00000016525	ENSPTRT00000031968	ENSBTAT00000010231	ENSNLET00000010926	ENSGGOT00000014566	ENSCPOT00000001672	ENSDORT00000001567	ENSMMUT00000022966	ENSCJAT00000011939	ENSMUST00000025349	ENSMICT00000009961	ENSPPYT00000018396	ENSOPRT00000005412	ENSOCUT00000014413	ENSRNOT00000016511	ENSSTOT00000013748	ENSTBET00000016582	ENSTSYT00000002660
ENST00000327237	ENSOGAT00000002353	ENSPTRT00000014853	ENSBTAT00000065138	ENSNLET00000020925	ENSGGOT00000004489	ENSCPOT00000011649	ENSDORT00000011639	ENSMMUT00000001281	ENSCJAT00000001486	ENSMUST00000033044	ENSMICT00000006067	ENSPPYT00000009063	ENSOPRT00000001468	ENSOCUT00000009793	ENSRNOT00000027290	ENSSTOT00000003335	ENSTBET00000014599	ENSTSYT00000012485
ENST00000355190	ENSOGAT00000011280	ENSPTRT00000050375	ENSBTAT00000047590	ENSNLET00000013819	ENSGGOT00000013993	ENSCPOT00000011654	ENSDORT00000007835	ENSMMUT00000007051	ENSCJAT00000019378	ENSMUST00000064709	ENSMICT00000010564	ENSPPYT00000019255	ENSOPRT00000006060	ENSOCUT00000007047	ENSRNOT00000000631	ENSSTOT00000005040	ENSTBET00000008060	ENSTSYT00000013124
ENST00000367942	ENSOGAT00000001919	ENSPTRT00000002907	ENSBTAT00000061155	ENSNLET00000018561	ENSGGOT00000024471	ENSCPOT00000010419	ENSDORT00000006089	ENSMMUT00000005748	ENSCJAT00000008802	ENSMUST00000027974	ENSMICT00000000038	ENSPPYT00000000715	ENSOPRT00000001855	ENSOCUT00000007931	ENSRNOT00000029202	ENSSTOT00000005758	ENSTBET00000015864	ENSTSYT00000002036
ENST00000287097	ENSOGAT00000008682	ENSPTRT00000033934	ENSBTAT00000061271	ENSNLET00000006236	ENSGGOT00000004110	ENSCPOT00000007641	ENSDORT00000014660	ENSMMUT00000025025	ENSCJAT00000019767	ENSMUST00000093812	ENSMICT00000009634	ENSPPYT00000019522	ENSOPRT00000014562	ENSOCUT00000006452	ENSRNOT00000037665	ENSSTOT00000013305	ENSTBET00000004728	ENSTSYT00000006439
ENST00000343846	ENSOGAT00000016276	ENSPTRT00000058165	ENSBTAT00000027951	ENSNLET00000000591	ENSGGOT00000028625	ENSCPOT00000000328	ENSDORT00000012701	ENSMMUT00000045331	ENSCJAT00000023058	ENSMUST00000085724	ENSMICT00000004905	ENSPPYT00000000207	ENSOPRT00000013981	ENSOCUT00000006226	ENSRNOT00000052407	ENSSTOT00000010455	ENSTBET00000015765	ENSTSYT00000001538
ENST00000373829	ENSOGAT00000004838	ENSPTRT00000040965	ENSBTAT00000016136	ENSNLET00000001820	ENSGGOT00000004839	ENSCPOT00000010364	ENSDORT00000009295	ENSMMUT00000028823	ENSCJAT00000026088	ENSMUST00000033674	ENSMICT00000000235	ENSPPYT00000023861	ENSOPRT00000001162	ENSOCUT00000016642	ENSRNOT00000004888	ENSSTOT00000015874	ENSTBET00000003340	ENSTSYT00000007440
ENST00000254691	ENSOGAT00000032013	ENSPTRT00000031227	ENSBTAT00000019112	ENSNLET00000021031	ENSGGOT00000003267	ENSCPOT00000021707	ENSDORT00000008298	ENSMMUT00000033503	ENSCJAT00000014470	ENSMUST00000118365	ENSMICT00000012354	ENSPPYT00000017930	ENSOPRT00000003655	ENSOCUT00000004767	ENSRNOT00000039057	ENSSTOT00000008158	ENSTBET00000004553	ENSTSYT00000001243
ENST00000357137	ENSOGAT00000026101	ENSPTRT00000026939	ENSBTAT00000008149	ENSNLET00000019662	ENSGGOT00000003954	ENSCPOT00000002323	ENSDORT00000012777	ENSMMUT00000005774	ENSCJAT00000009141	ENSMUST00000163754	ENSMICT00000008888	ENSPPYT00000013771	ENSOPRT00000006714	ENSOCUT00000009644	ENSRNOT00000068666	ENSSTOT00000003202	ENSTBET00000008583	ENSTSYT00000012826
ENST00000262455	ENSOGAT00000013402	ENSPTRT00000039182	ENSBTAT00000012930	ENSNLET00000021777	ENSGGOT00000007738	ENSCPOT00000008432	ENSDORT00000016149	ENSMMUT00000015983	ENSCJAT00000022155	ENSMUST00000030028	ENSMICT00000003151	ENSPPYT00000022678	ENSOPRT00000000004	ENSOCUT00000004225	ENSRNOT00000007711	ENSSTOT00000010130	ENSTBET00000007266	ENSTSYT00000013491
ENST00000311160	ENSOGAT00000013777	ENSPTRT00000048607	ENSBTAT00000012722	ENSNLET00000005669	ENSGGOT00000003474	ENSCPOT00000005196	ENSDORT00000003112	ENSMMUT00000027241	ENSCJAT00000028751	ENSMUST00000020695	ENSMICT00000009491	ENSPPYT00000020472	ENSOPRT00000017131	ENSOCUT00000001215	ENSRNOT00000012661	ENSSTOT00000015798	ENSTBET00000003020	ENSTSYT00000012100
ENST00000435907	ENSOGAT00000004498	ENSPTRT00000044068	ENSBTAT00000004287	ENSNLET00000000600	ENSGGOT00000009772	ENSCPOT00000011196	ENSDORT00000008864	ENSMMUT00000014124	ENSCJAT00000020013	ENSMUST00000077472	ENSMICT00000008851	ENSPPYT00000003304	ENSOPRT00000007696	ENSOCUT00000007602	ENSRNOT00000022157	ENSSTOT00000006838	ENSTBET00000011397	ENSTSYT00000000305
ENST00000299134	ENSOGAT00000033629	ENSPTRT00000009936	ENSBTAT00000063609	ENSNLET00000008689	ENSGGOT00000014968	ENSCPOT00000014117	ENSDORT00000009099	ENSMMUT00000021177	ENSCJAT00000030176	ENSMUST00000058472	ENSMICT00000017624	ENSPPYT00000005835	ENSOPRT00000016202	ENSOCUT00000016228	ENSRNOT00000067290	ENSSTOT00000011612	ENSTBET00000013139	ENSTSYT00000011316
ENST00000341170	ENSOGAT00000013887	ENSPTRT00000039142	ENSBTAT00000019587	ENSNLET00000021556	ENSGGOT00000001384	ENSCPOT00000010156	ENSDORT00000013649	ENSMMUT00000017200	ENSCJAT00000021100	ENSMUST00000107772	ENSMICT00000012667	ENSPPYT00000022658	ENSOPRT00000015569	ENSOCUT00000013842	ENSRNOT00000012915	ENSSTOT00000002675	ENSTBET00000003297	ENSTSYT00000011895
ENST00000332853	ENSOGAT00000006896	ENSPTRT00000004637	ENSBTAT00000008254	ENSNLET00000020102	ENSGGOT00000032936	ENSCPOT00000028353	ENSDORT00000004874	ENSMMUT00000012447	ENSCJAT00000026260	ENSMUST00000053175	ENSMICT00000006767	ENSPPYT00000003423	ENSOPRT00000005761	ENSOCUT00000007504	ENSRNOT00000027198	ENSSTOT00000010489	ENSTBET00000003181	ENSTSYT00000012429
ENST00000299608	ENSOGAT00000009496	ENSPTRT00000018526	ENSBTAT00000009957	ENSNLET00000011612	ENSGGOT00000005514	ENSCPOT00000010580	ENSDORT00000008922	ENSMMUT00000024918	ENSCJAT00000005794	ENSMUST00000025515	ENSMICT00000014035	ENSPPYT00000010768	ENSOPRT00000011587	ENSOCUT00000005001	ENSRNOT00000061420	ENSSTOT00000009141	ENSTBET00000014947	ENSTSYT00000008401
ENST00000265734	ENSOGAT00000027280	ENSPTRT00000035908	ENSBTAT00000061349	ENSNLET00000019544	ENSGGOT00000026266	ENSCPOT00000004865	ENSDORT00000006523	ENSMMUT00000012156	ENSCJAT00000027002	ENSMUST00000165117	ENSMICT00000004216	ENSPPYT00000020758	ENSOPRT00000010582	ENSOCUT00000011075	ENSRNOT00000012597	ENSSTOT00000014025	ENSTBET00000005614	ENSTSYT00000013120
ENST00000439741	ENSOGAT00000006270	ENSPTRT00000050013	ENSBTAT00000016579	ENSNLET00000012406	ENSGGOT00000004793	ENSCPOT00000008346	ENSDORT00000016368	ENSMMUT00000046136	ENSCJAT00000001473	ENSMUST00000054356	ENSMICT00000000298	ENSPPYT00000001099	ENSOPRT00000010827	ENSOCUT00000004618	ENSRNOT00000056262	ENSSTOT00000006606	ENSTBET00000012051	ENSTSYT00000009309
ENST00000396636	ENSOGAT00000014845	ENSPTRT00000027503	ENSBTAT00000000271	ENSNLET00000004971	ENSGGOT00000002839	ENSCPOT00000004489	ENSDORT00000007869	ENSMMUT00000027137	ENSCJAT00000055809	ENSMUST00000112631	ENSMICT00000016287	ENSPPYT00000016349	ENSOPRT00000014578	ENSOCUT00000007850	ENSRNOT00000030991	ENSSTOT00000012144	ENSTBET00000008887	ENSTSYT00000008506
ENST00000371833	ENSOGAT00000005780	ENSPTRT00000001367	ENSBTAT00000012132	ENSNLET00000020796	ENSGGOT00000003584	ENSCPOT00000014020	ENSDORT00000005814	ENSMMUT00000026887	ENSCJAT00000041856	ENSMUST00000030274	ENSMICT00000003279	ENSPPYT00000001645	ENSOPRT00000012389	ENSOCUT00000006475	ENSRNOT00000010628	ENSSTOT00000006987	ENSTBET00000000683	ENSTSYT00000007534
ENST00000544117	ENSOGAT00000005521	ENSPTRT00000009029	ENSBTAT00000021929	ENSNLET00000022820	ENSGGOT00000013693	ENSCPOT00000002805	ENSDORT00000011811	ENSMMUT00000020316	ENSCJAT00000040710	ENSMUST00000169721	ENSMICT00000007358	ENSPPYT00000005290	ENSOPRT00000014765	ENSOCUT00000005450	ENSRNOT00000045473	ENSSTOT00000001969	ENSTBET00000002363	ENSTSYT00000001374
ENST00000431473	ENSOGAT00000000459	ENSPTRT00000001158	ENSBTAT00000023262	ENSNLET00000015670	ENSGGOT00000030896	ENSCPOT00000005239	ENSDORT00000015839	ENSMMUT00000018413	ENSCJAT00000005951	ENSMUST00000049994	ENSMICT00000014885	ENSPPYT00000001759	ENSOPRT00000003188	ENSOCUT00000014726	ENSRNOT00000011586	ENSSTOT00000000109	ENSTBET00000014617	ENSTSYT00000008446
ENST00000324300	ENSOGAT00000015820	ENSPTRT00000030657	ENSBTAT00000018173	ENSNLET00000007177	ENSGGOT00000000624	ENSCPOT00000004608	ENSDORT00000014305	ENSMMUT00000041713	ENSCJAT00000004681	ENSMUST00000034029	ENSMICT00000000580	ENSPPYT00000017560	ENSOPRT00000005997	ENSOCUT00000001249	ENSRNOT00000058162	ENSSTOT00000003950	ENSTBET00000000599	ENSTSYT00000002625
ENST00000367669	ENSOGAT00000015469	ENSPTRT00000058486	ENSBTAT00000044452	ENSNLET00000019682	ENSGGOT00000013507	ENSCPOT00000010857	ENSDORT00000013376	ENSMMUT00000044569	ENSCJAT00000017096	ENSMUST00000076894	ENSMICT00000008566	ENSPPYT00000000575	ENSOPRT00000010519	ENSOCUT00000005468	ENSRNOT00000066388	ENSSTOT00000008459	ENSTBET00000012416	ENSTSYT00000003658
ENST00000261592	ENSOGAT00000016013	ENSPTRT00000018281	ENSBTAT00000064561	ENSNLET00000008068	ENSGGOT00000004113	ENSCPOT00000013155	ENSDORT00000016293	ENSMMUT00000048243	ENSCJAT00000041157	ENSMUST00000164186	ENSMICT00000010137	ENSPPYT00000010619	ENSOPRT00000015804	ENSOCUT00000007904	ENSRNOT00000067286	ENSSTOT00000002666	ENSTBET00000000841	ENSTSYT00000006995
ENST00000370205	ENSOGAT00000012506	ENSPTRT00000001845	ENSBTAT00000005796	ENSNLET00000014328	ENSGGOT00000004857	ENSCPOT00000022238	ENSDORT00000008564	ENSMMUT00000033259	ENSCJAT00000035450	ENSMUST00000039442	ENSMICT00000008949	ENSPPYT00000001360	ENSOPRT00000004850	ENSOCUT00000006321	ENSRNOT00000015354	ENSSTOT00000015913	ENSTBET00000007639	ENSTSYT00000005998
ENST00000509005	ENSOGAT00000000389	ENSPTRT00000031490	ENSBTAT00000036838	ENSNLET00000006055	ENSGGOT00000003320	ENSCPOT00000006737	ENSDORT00000004398	ENSMMUT00000033078	ENSCJAT00000032346	ENSMUST00000040972	ENSMICT00000011815	ENSPPYT00000018086	ENSOPRT00000012982	ENSOCUT00000000617	ENSRNOT00000022259	ENSSTOT00000008978	ENSTBET00000009166	ENSTSYT00000008280
ENST00000487418	ENSOGAT00000009830	ENSPTRT00000012757	ENSBTAT00000005782	ENSNLET00000003867	ENSGGOT00000000182	ENSCPOT00000026625	ENSDORT00000007602	ENSMMUT00000020288	ENSCJAT00000040976	ENSMUST00000028807	ENSMICT00000008783	ENSPPYT00000007490	ENSOPRT00000011778	ENSOCUT00000008973	ENSRNOT00000013829	ENSSTOT00000014521	ENSTBET00000005780	ENSTSYT00000011279
ENST00000228318	ENSOGAT00000012227	ENSPTRT00000009797	ENSBTAT00000017131	ENSNLET00000007631	ENSGGOT00000015939	ENSCPOT00000011718	ENSDORT00000011432	ENSMMUT00000032414	ENSCJAT00000004633	ENSMUST00000170810	ENSMICT00000003520	ENSPPYT00000005755	ENSOPRT00000006723	ENSOCUT00000011848	ENSRNOT00000048575	ENSSTOT00000009183	ENSTBET00000014533	ENSTSYT00000004081
ENST00000341529	ENSOGAT00000008660	ENSPTRT00000015101	ENSBTAT00000009760	ENSNLET00000004994	ENSGGOT00000029076	ENSCPOT00000013558	ENSDORT00000015014	ENSMMUT00000033090	ENSCJAT00000032888	ENSMUST00000034339	ENSMICT00000010351	ENSPPYT00000008722	ENSOPRT00000010014	ENSOCUT00000010194	ENSRNOT00000017983	ENSSTOT00000006264	ENSTBET00000001325	ENSTSYT00000009594
ENST00000304883	ENSOGAT00000001468	ENSPTRT00000030392	ENSBTAT00000021612	ENSNLET00000016953	ENSGGOT00000002599	ENSCPOT00000027665	ENSDORT00000010680	ENSMMUT00000028540	ENSCJAT00000030525	ENSMUST00000029822	ENSMICT00000004751	ENSPPYT00000017400	ENSOPRT00000002305	ENSOCUT00000009581	ENSRNOT00000012473	ENSSTOT00000008033	ENSTBET00000014279	ENSTSYT00000011348
ENST00000306107	ENSOGAT00000004359	ENSPTRT00000028388	ENSBTAT00000000097	ENSNLET00000004462	ENSGGOT00000008522	ENSCPOT00000013299	ENSDORT00000009835	ENSMMUT00000012743	ENSCJAT00000009226	ENSMUST00000023312	ENSMICT00000012509	ENSPPYT00000015789	ENSOPRT00000016895	ENSOCUT00000011294	ENSRNOT00000002738	ENSSTOT00000000863	ENSTBET00000014601	ENSTSYT00000004143
ENST00000302516	ENSOGAT00000016058	ENSPTRT00000015329	ENSBTAT00000014050	ENSNLET00000020558	ENSGGOT00000003271	ENSCPOT00000000591	ENSDORT00000014176	ENSMMUT00000025032	ENSCJAT00000028853	ENSMUST00000042012	ENSMICT00000001366	ENSPPYT00000008859	ENSOPRT00000009859	ENSOCUT00000004142	ENSRNOT00000023854	ENSSTOT00000007077	ENSTBET00000002974	ENSTSYT00000007940
ENST00000413919	ENSOGAT00000017158	ENSPTRT00000035099	ENSBTAT00000021317	ENSNLET00000018835	ENSGGOT00000028410	ENSCPOT00000015210	ENSDORT00000013949	ENSMMUT00000042153	ENSCJAT00000002770	ENSMUST00000049887	ENSMICT00000013651	ENSPPYT00000020661	ENSOPRT00000013968	ENSOCUT00000014806	ENSRNOT00000013865	ENSSTOT00000008482	ENSTBET00000001633	ENSTSYT00000003375
ENST00000372622	ENSOGAT00000004988	ENSPTRT00000025245	ENSBTAT00000022261	ENSNLET00000008633	ENSGGOT00000004662	ENSCPOT00000012926	ENSDORT00000011772	ENSMMUT00000044992	ENSCJAT00000033801	ENSMUST00000017443	ENSMICT00000011207	ENSPPYT00000012852	ENSOPRT00000014260	ENSOCUT00000012171	ENSRNOT00000020280	ENSSTOT00000006484	ENSTBET00000006080	ENSTSYT00000004226
ENST00000523282	ENSOGAT00000000648	ENSPTRT00000056086	ENSBTAT00000028671	ENSNLET00000012030	ENSGGOT00000003440	ENSCPOT00000002825	ENSDORT00000009598	ENSMMUT00000025193	ENSCJAT00000061059	ENSMUST00000066668	ENSMICT00000015760	ENSPPYT00000015354	ENSOPRT00000007572	ENSOCUT00000012479	ENSRNOT00000026799	ENSSTOT00000011540	ENSTBET00000016498	ENSTSYT00000012761
ENST00000217800	ENSOGAT00000008034	ENSPTRT00000018414	ENSBTAT00000012034	ENSNLET00000015207	ENSGGOT00000014369	ENSCPOT00000010296	ENSDORT00000015769	ENSMMUT00000015271	ENSCJAT00000003507	ENSMUST00000043286	ENSMICT00000008977	ENSPPYT00000010707	ENSOPRT00000007658	ENSOCUT00000003747	ENSRNOT00000016189	ENSSTOT00000004390	ENSTBET00000010416	ENSTSYT00000010701
ENST00000409489	ENSOGAT00000001912	ENSPTRT00000035215	ENSBTAT00000063163	ENSNLET00000019406	ENSGGOT00000015921	ENSCPOT00000006071	ENSDORT00000006440	ENSMMUT00000007453	ENSCJAT00000017375	ENSMUST00000167234	ENSMICT00000012962	ENSPPYT00000020584	ENSOPRT00000006554	ENSOCUT00000000122	ENSRNOT00000016175	ENSSTOT00000013271	ENSTBET00000017139	ENSTSYT00000011010
ENST00000005178	ENSOGAT00000008475	ENSPTRT00000035942	ENSBTAT00000038879	ENSNLET00000020229	ENSGGOT00000002480	ENSCPOT00000014388	ENSDORT00000014682	ENSMMUT00000030108	ENSCJAT00000025775	ENSMUST00000019721	ENSMICT00000005749	ENSPPYT00000020738	ENSOPRT00000004120	ENSOCUT00000017119	ENSRNOT00000012760	ENSSTOT00000006061	ENSTBET00000011100	ENSTSYT00000011414
ENST00000371666	ENSOGAT00000001115	ENSPTRT00000041402	ENSBTAT00000025754	ENSNLET00000018917	ENSGGOT00000014699	ENSCPOT00000011152	ENSDORT00000012153	ENSMMUT00000016116	ENSCJAT00000010155	ENSMUST00000033418	ENSMICT00000011750	ENSPPYT00000024099	ENSOPRT00000007017	ENSOCUT00000013690	ENSRNOT00000017746	ENSSTOT00000015581	ENSTBET00000012648	ENSTSYT00000000370
ENST00000355100	ENSOGAT00000008138	ENSPTRT00000013695	ENSBTAT00000026579	ENSNLET00000014683	ENSGGOT00000011727	ENSCPOT00000009720	ENSDORT00000001937	ENSMMUT00000020230	ENSCJAT00000025046	ENSMUST00000037315	ENSMICT00000001925	ENSPPYT00000007982	ENSOPRT00000002748	ENSOCUT00000013527	ENSRNOT00000023506	ENSSTOT00000005847	ENSTBET00000011716	ENSTSYT00000005160
ENST00000280154	ENSOGAT00000012586	ENSPTRT00000005607	ENSBTAT00000025886	ENSNLET00000019864	ENSGGOT00000033795	ENSCPOT00000008031	ENSDORT00000002615	ENSMMUT00000024185	ENSCJAT00000036943	ENSMUST00000165617	ENSMICT00000008754	ENSPPYT00000003190	ENSOPRT00000015434	ENSOCUT00000016823	ENSRNOT00000020155	ENSSTOT00000015625	ENSTBET00000000359	ENSTSYT00000014537
ENST00000264448	ENSOGAT00000005991	ENSPTRT00000022437	ENSBTAT00000047267	ENSNLET00000000374	ENSGGOT00000013240	ENSCPOT00000007121	ENSDORT00000014407	ENSMMUT00000003995	ENSCJAT00000007975	ENSMUST00000072018	ENSMICT00000015565	ENSPPYT00000014267	ENSOPRT00000001367	ENSOCUT00000006237	ENSRNOT00000033545	ENSSTOT00000005465	ENSTBET00000000662	ENSTSYT00000004950
ENST00000354725	ENSOGAT00000013359	ENSPTRT00000048407	ENSBTAT00000014154	ENSNLET00000015675	ENSGGOT00000009661	ENSCPOT00000006316	ENSDORT00000000336	ENSMMUT00000021638	ENSCJAT00000034502	ENSMUST00000001460	ENSMICT00000000099	ENSPPYT00000020953	ENSOPRT00000013423	ENSOCUT00000001622	ENSRNOT00000047698	ENSSTOT00000002155	ENSTBET00000004010	ENSTSYT00000004979
ENST00000474710	ENSOGAT00000033212	ENSPTRT00000050494	ENSBTAT00000015819	ENSNLET00000006091	ENSGGOT00000029454	ENSCPOT00000024560	ENSDORT00000008535	ENSMMUT00000046945	ENSCJAT00000017875	ENSMUST00000114695	ENSMICT00000008806	ENSPPYT00000015729	ENSOPRT00000006657	ENSOCUT00000004232	ENSRNOT00000002692	ENSSTOT00000001825	ENSTBET00000016623	ENSTSYT00000009685
ENST00000356232	ENSOGAT00000004866	ENSPTRT00000049800	ENSBTAT00000005894	ENSNLET00000004391	ENSGGOT00000006125	ENSCPOT00000010020	ENSDORT00000014304	ENSMMUT00000030632	ENSCJAT00000027241	ENSMUST00000035166	ENSMICT00000004448	ENSPPYT00000016407	ENSOPRT00000002350	ENSOCUT00000015754	ENSRNOT00000015240	ENSSTOT00000012165	ENSTBET00000016104	ENSTSYT00000003101
ENST00000284268	ENSOGAT00000006666	ENSPTRT00000031095	ENSBTAT00000017818	ENSNLET00000015126	ENSGGOT00000015382	ENSCPOT00000012456	ENSDORT00000001668	ENSMMUT00000030877	ENSCJAT00000021185	ENSMUST00000022875	ENSMICT00000017365	ENSPPYT00000017846	ENSOPRT00000008429	ENSOCUT00000012052	ENSRNOT00000068484	ENSSTOT00000014633	ENSTBET00000000803	ENSTSYT00000013340
ENST00000289382	ENSOGAT00000014510	ENSPTRT00000022816	ENSBTAT00000010879	ENSNLET00000018487	ENSGGOT00000005096	ENSCPOT00000012145	ENSDORT00000004481	ENSMMUT00000001617	ENSCJAT00000041763	ENSMUST00000003219	ENSMICT00000002967	ENSPPYT00000014043	ENSOPRT00000009913	ENSOCUT00000004451	ENSRNOT00000031352	ENSSTOT00000002672	ENSTBET00000016385	ENSTSYT00000006726
ENST00000264515	ENSOGAT00000007384	ENSPTRT00000065984	ENSBTAT00000021204	ENSNLET00000018424	ENSGGOT00000014855	ENSCPOT00000011878	ENSDORT00000014245	ENSMMUT00000026438	ENSCJAT00000033416	ENSMUST00000027700	ENSMICT00000013219	ENSPPYT00000000355	ENSOPRT00000010120	ENSOCUT00000000095	ENSRNOT00000036110	ENSSTOT00000002659	ENSTBET00000000932	ENSTSYT00000000255
ENST00000202017	ENSOGAT00000029157	ENSPTRT00000024831	ENSBTAT00000012845	ENSNLET00000012391	ENSGGOT00000028230	ENSCPOT00000005982	ENSDORT00000010400	ENSMMUT00000001389	ENSCJAT00000040810	ENSMUST00000028972	ENSMICT00000015882	ENSPPYT00000012652	ENSOPRT00000006398	ENSOCUT00000016705	ENSRNOT00000012141	ENSSTOT00000009350	ENSTBET00000014226	ENSTSYT00000014701
ENST00000418425	ENSOGAT00000007967	ENSPTRT00000009138	ENSBTAT00000038541	ENSNLET00000022685	ENSGGOT00000025047	ENSCPOT00000007988	ENSDORT00000010377	ENSMMUT00000017460	ENSCJAT00000034232	ENSMUST00000037001	ENSMICT00000009528	ENSPPYT00000005360	ENSOPRT00000005718	ENSOCUT00000009011	ENSRNOT00000068073	ENSSTOT00000009101	ENSTBET00000011883	ENSTSYT00000012397
ENST00000336648	ENSOGAT00000002196	ENSPTRT00000025914	ENSBTAT00000057284	ENSNLET00000005746	ENSGGOT00000011178	ENSCPOT00000008030	ENSDORT00000011549	ENSMMUT00000021835	ENSCJAT00000005168	ENSMUST00000099512	ENSMICT00000013188	ENSPPYT00000013238	ENSOPRT00000004219	ENSOCUT00000012913	ENSRNOT00000002291	ENSSTOT00000015476	ENSTBET00000011928	ENSTSYT00000005904
ENST00000393077	ENSOGAT00000006613	ENSPTRT00000065104	ENSBTAT00000008830	ENSNLET00000020194	ENSGGOT00000010606	ENSCPOT00000006228	ENSDORT00000009664	ENSMMUT00000001637	ENSCJAT00000037229	ENSMUST00000095950	ENSMICT00000007340	ENSPPYT00000003201	ENSOPRT00000004758	ENSOCUT00000003604	ENSRNOT00000067460	ENSSTOT00000013849	ENSTBET00000013813	ENSTSYT00000009677
ENST00000392096	ENSOGAT00000014091	ENSPTRT00000023967	ENSBTAT00000019735	ENSNLET00000011524	ENSGGOT00000010830	ENSCPOT00000009213	ENSDORT00000002773	ENSMMUT00000001123	ENSCJAT00000022205	ENSMUST00000006721	ENSMICT00000007035	ENSPPYT00000015336	ENSOPRT00000010954	ENSOCUT00000000206	ENSRNOT00000024283	ENSSTOT00000009320	ENSTBET00000010678	ENSTSYT00000013996
ENST00000409228	ENSOGAT00000003102	ENSPTRT00000023651	ENSBTAT00000017564	ENSNLET00000008688	ENSGGOT00000016887	ENSCPOT00000008983	ENSDORT00000014757	ENSMMUT00000007913	ENSCJAT00000006897	ENSMUST00000027263	ENSMICT00000013713	ENSPPYT00000015155	ENSOPRT00000001552	ENSOCUT00000011687	ENSRNOT00000016856	ENSSTOT00000009655	ENSTBET00000007753	ENSTSYT00000002631
ENST00000374747	ENSOGAT00000015184	ENSPTRT00000017940	ENSBTAT00000042613	ENSNLET00000017622	ENSGGOT00000023679	ENSCPOT00000007874	ENSDORT00000010288	ENSMMUT00000040886	ENSCJAT00000003005	ENSMUST00000044271	ENSMICT00000008598	ENSPPYT00000010198	ENSOPRT00000009405	ENSOCUT00000003886	ENSRNOT00000054973	ENSSTOT00000000080	ENSTBET00000003209	ENSTSYT00000011993
ENST00000359591	ENSOGAT00000005093	ENSPTRT00000048448	ENSBTAT00000021782	ENSNLET00000018870	ENSGGOT00000015752	ENSCPOT00000014640	ENSDORT00000004922	ENSMMUT00000022641	ENSCJAT00000016608	ENSMUST00000022782	ENSMICT00000006355	ENSPPYT00000006672	ENSOPRT00000001045	ENSOCUT00000016379	ENSRNOT00000015468	ENSSTOT00000010327	ENSTBET00000011268	ENSTSYT00000006117
ENST00000493344	ENSOGAT00000012351	ENSPTRT00000021908	ENSBTAT00000025991	ENSNLET00000004304	ENSGGOT00000011025	ENSCPOT00000007830	ENSDORT00000006778	ENSMMUT00000011587	ENSCJAT00000001929	ENSMUST00000077693	ENSMICT00000015856	ENSPPYT00000014584	ENSOPRT00000010794	ENSOCUT00000017077	ENSRNOT00000051696	ENSSTOT00000011370	ENSTBET00000012924	ENSTSYT00000001021
ENST00000269593	ENSOGAT00000001644	ENSPTRT00000016809	ENSBTAT00000011361	ENSNLET00000002443	ENSGGOT00000009923	ENSCPOT00000008130	ENSDORT00000016055	ENSMMUT00000013106	ENSCJAT00000026347	ENSMUST00000017637	ENSMICT00000009800	ENSPPYT00000009888	ENSOPRT00000000948	ENSOCUT00000002825	ENSRNOT00000014153	ENSSTOT00000004856	ENSTBET00000001935	ENSTSYT00000004030
ENST00000357613	ENSOGAT00000000791	ENSPTRT00000046685	ENSBTAT00000020505	ENSNLET00000016623	ENSGGOT00000016949	ENSCPOT00000021959	ENSDORT00000006330	ENSMMUT00000002204	ENSCJAT00000027737	ENSMUST00000034431	ENSMICT00000014052	ENSPPYT00000008882	ENSOPRT00000016716	ENSOCUT00000009279	ENSRNOT00000026256	ENSSTOT00000004134	ENSTBET00000015593	ENSTSYT00000007318
ENST00000381647	ENSOGAT00000002037	ENSPTRT00000031239	ENSBTAT00000006486	ENSNLET00000021219	ENSGGOT00000009196	ENSCPOT00000013096	ENSDORT00000016319	ENSMMUT00000005625	ENSCJAT00000002226	ENSMUST00000046633	ENSMICT00000011883	ENSPPYT00000017939	ENSOPRT00000004655	ENSOCUT00000009242	ENSRNOT00000020829	ENSSTOT00000008281	ENSTBET00000010209	ENSTSYT00000005400
ENST00000394631	ENSOGAT00000004695	ENSPTRT00000030443	ENSBTAT00000000992	ENSNLET00000021119	ENSGGOT00000008719	ENSCPOT00000004261	ENSDORT00000000482	ENSMMUT00000029864	ENSCJAT00000007764	ENSMUST00000106366	ENSMICT00000003323	ENSPPYT00000017429	ENSOPRT00000012082	ENSOCUT00000003899	ENSRNOT00000013149	ENSSTOT00000005571	ENSTBET00000003459	ENSTSYT00000013668
ENST00000256178	ENSOGAT00000001766	ENSPTRT00000006357	ENSBTAT00000001061	ENSNLET00000022446	ENSGGOT00000033604	ENSCPOT00000007886	ENSDORT00000009830	ENSMMUT00000022177	ENSCJAT00000021461	ENSMUST00000033050	ENSMICT00000008620	ENSPPYT00000004158	ENSOPRT00000008212	ENSOCUT00000009658	ENSRNOT00000030474	ENSSTOT00000013578	ENSTBET00000006590	ENSTSYT00000001649
ENST00000539916	ENSOGAT00000010464	ENSPTRT00000003462	ENSBTAT00000063964	ENSNLET00000018577	ENSGGOT00000029649	ENSCPOT00000011081	ENSDORT00000009273	ENSMMUT00000005840	ENSCJAT00000021318	ENSMUST00000086556	ENSMICT00000005137	ENSPPYT00000000339	ENSOPRT00000006340	ENSOCUT00000014474	ENSRNOT00000065094	ENSSTOT00000014795	ENSTBET00000015496	ENSTSYT00000012626
ENST00000265087	ENSOGAT00000007542	ENSPTRT00000032427	ENSBTAT00000002384	ENSNLET00000000593	ENSGGOT00000013780	ENSCPOT00000015520	ENSDORT00000004430	ENSMMUT00000020736	ENSCJAT00000037594	ENSMUST00000020546	ENSMICT00000008196	ENSPPYT00000018687	ENSOPRT00000014310	ENSOCUT00000007981	ENSRNOT00000028146	ENSSTOT00000004407	ENSTBET00000007449	ENSTSYT00000004414
ENST00000220751	ENSOGAT00000012629	ENSPTRT00000037765	ENSBTAT00000020312	ENSNLET00000002538	ENSGGOT00000010883	ENSCPOT00000006480	ENSDORT00000002598	ENSMMUT00000029152	ENSCJAT00000000402	ENSMUST00000037035	ENSMICT00000007407	ENSPPYT00000021849	ENSOPRT00000003644	ENSOCUT00000001542	ENSRNOT00000012963	ENSSTOT00000015169	ENSTBET00000002102	ENSTSYT00000006348
ENST00000286574	ENSOGAT00000008057	ENSPTRT00000009517	ENSBTAT00000019636	ENSNLET00000003959	ENSGGOT00000002600	ENSCPOT00000001802	ENSDORT00000006658	ENSMMUT00000023720	ENSCJAT00000028500	ENSMUST00000020439	ENSMICT00000012090	ENSPPYT00000005601	ENSOPRT00000002531	ENSOCUT00000014907	ENSRNOT00000006426	ENSSTOT00000013984	ENSTBET00000003053	ENSTSYT00000002119
ENST00000256658	ENSOGAT00000024268	ENSPTRT00000002111	ENSBTAT00000004711	ENSNLET00000005858	ENSGGOT00000011442	ENSCPOT00000001302	ENSDORT00000002983	ENSMMUT00000027431	ENSCJAT00000023668	ENSMUST00000076599	ENSMICT00000001392	ENSPPYT00000001213	ENSOPRT00000015174	ENSOCUT00000003778	ENSRNOT00000026382	ENSSTOT00000014697	ENSTBET00000014398	ENSTSYT00000006551
ENST00000355312	ENSOGAT00000012285	ENSPTRT00000044198	ENSBTAT00000008526	ENSNLET00000003169	ENSGGOT00000009517	ENSCPOT00000012988	ENSDORT00000008862	ENSMMUT00000045622	ENSCJAT00000018852	ENSMUST00000086564	ENSMICT00000017196	ENSPPYT00000005854	ENSOPRT00000002566	ENSOCUT00000001058	ENSRNOT00000045946	ENSSTOT00000004581	ENSTBET00000003536	ENSTSYT00000000485
ENST00000528080	ENSOGAT00000003415	ENSPTRT00000006337	ENSBTAT00000002111	ENSNLET00000022389	ENSGGOT00000003001	ENSCPOT00000013908	ENSDORT00000005422	ENSMMUT00000007926	ENSCJAT00000022266	ENSMUST00000094097	ENSMICT00000004434	ENSPPYT00000023298	ENSOPRT00000005287	ENSOCUT00000005754	ENSRNOT00000016224	ENSSTOT00000010381	ENSTBET00000007247	ENSTSYT00000005733
ENST00000458310	ENSOGAT00000031934	ENSPTRT00000055776	ENSBTAT00000050739	ENSNLET00000018037	ENSGGOT00000031686	ENSCPOT00000003658	ENSDORT00000003707	ENSMMUT00000001714	ENSCJAT00000057110	ENSMUST00000050997	ENSMICT00000006729	ENSPPYT00000018195	ENSOPRT00000005487	ENSOCUT00000010886	ENSRNOT00000017098	ENSSTOT00000016414	ENSTBET00000008063	ENSTSYT00000009900
ENST00000246077	ENSOGAT00000002709	ENSPTRT00000067650	ENSBTAT00000017725	ENSNLET00000012112	ENSGGOT00000009032	ENSCPOT00000005787	ENSDORT00000000723	ENSMMUT00000006318	ENSCJAT00000040972	ENSMUST00000028963	ENSMICT00000013497	ENSPPYT00000012615	ENSOPRT00000005112	ENSOCUT00000014500	ENSRNOT00000008713	ENSSTOT00000008631	ENSTBET00000013799	ENSTSYT00000006236
ENST00000505177	ENSOGAT00000006372	ENSPTRT00000029544	ENSBTAT00000016693	ENSNLET00000001329	ENSGGOT00000004891	ENSCPOT00000011173	ENSDORT00000007383	ENSMMUT00000011685	ENSCJAT00000002991	ENSMUST00000114449	ENSMICT00000012947	ENSPPYT00000016888	ENSOPRT00000001445	ENSOCUT00000006606	ENSRNOT00000007181	ENSSTOT00000005711	ENSTBET00000015064	ENSTSYT00000013002
ENST00000331299	ENSOGAT00000013136	ENSPTRT00000011515	ENSBTAT00000045803	ENSNLET00000004993	ENSGGOT00000001539	ENSCPOT00000011054	ENSDORT00000006519	ENSMMUT00000013471	ENSCJAT00000032000	ENSMUST00000044634	ENSMICT00000010810	ENSPPYT00000006798	ENSOPRT00000003292	ENSOCUT00000014331	ENSRNOT00000034342	ENSSTOT00000015730	ENSTBET00000002652	ENSTSYT00000003210
ENST00000378747	ENSOGAT00000000625	ENSPTRT00000046629	ENSBTAT00000021808	ENSNLET00000015658	ENSGGOT00000014774	ENSCPOT00000007633	ENSDORT00000005802	ENSMMUT00000007881	ENSCJAT00000018610	ENSMUST00000114996	ENSMICT00000009523	ENSPPYT00000002505	ENSOPRT00000006553	ENSOCUT00000006796	ENSRNOT00000024162	ENSSTOT00000011168	ENSTBET00000016194	ENSTSYT00000001757
ENST00000295888	ENSOGAT00000015413	ENSPTRT00000030226	ENSBTAT00000061443	ENSNLET00000011836	ENSGGOT00000006436	ENSCPOT00000006815	ENSDORT00000011117	ENSMMUT00000029780	ENSCJAT00000010789	ENSMUST00000174598	ENSMICT00000004983	ENSPPYT00000017319	ENSOPRT00000012367	ENSOCUT00000017177	ENSRNOT00000033194	ENSSTOT00000009799	ENSTBET00000016124	ENSTSYT00000008306
ENST00000275235	ENSOGAT00000017080	ENSPTRT00000046894	ENSBTAT00000004546	ENSNLET00000019230	ENSGGOT00000003379	ENSCPOT00000001492	ENSDORT00000001145	ENSMMUT00000009019	ENSCJAT00000037676	ENSMUST00000162610	ENSMICT00000013600	ENSPPYT00000019854	ENSOPRT00000014190	ENSOCUT00000003605	ENSRNOT00000046539	ENSSTOT00000012815	ENSTBET00000006856	ENSTSYT00000011489
ENST00000234831	ENSOGAT00000016571	ENSPTRT00000001447	ENSBTAT00000012534	ENSNLET00000004363	ENSGGOT00000033888	ENSCPOT00000004941	ENSDORT00000002280	ENSMMUT00000047102	ENSCJAT00000009594	ENSMUST00000030361	ENSMICT00000014315	ENSPPYT00000001589	ENSOPRT00000005596	ENSOCUT00000008131	ENSRNOT00000013224	ENSSTOT00000002676	ENSTBET00000013236	ENSTSYT00000009831
ENST00000504387	ENSOGAT00000009546	ENSPTRT00000064592	ENSBTAT00000063698	ENSNLET00000000872	ENSGGOT00000016335	ENSCPOT00000012524	ENSDORT00000007483	ENSMMUT00000022684	ENSCJAT00000009763	ENSMUST00000169127	ENSMICT00000017359	ENSPPYT00000018878	ENSOPRT00000010671	ENSOCUT00000015288	ENSRNOT00000067374	ENSSTOT00000014993	ENSTBET00000001246	ENSTSYT00000004434
ENST00000341071	ENSOGAT00000031493	ENSPTRT00000000946	ENSBTAT00000027526	ENSNLET00000003494	ENSGGOT00000013169	ENSCPOT00000007196	ENSDORT00000003082	ENSMMUT00000001789	ENSCJAT00000001801	ENSMUST00000048162	ENSMICT00000014391	ENSPPYT00000001894	ENSOPRT00000000869	ENSOCUT00000011969	ENSRNOT00000011054	ENSSTOT00000006686	ENSTBET00000017746	ENSTSYT00000007686
ENST00000427836	ENSOGAT00000003684	ENSPTRT00000056154	ENSBTAT00000010299	ENSNLET00000009106	ENSGGOT00000001006	ENSCPOT00000004164	ENSDORT00000009938	ENSMMUT00000011337	ENSCJAT00000005540	ENSMUST00000097713	ENSMICT00000009769	ENSPPYT00000015257	ENSOPRT00000015222	ENSOCUT00000015377	ENSRNOT00000032782	ENSSTOT00000016491	ENSTBET00000012791	ENSTSYT00000019455
ENST00000355377	ENSOGAT00000012021	ENSPTRT00000067308	ENSBTAT00000018914	ENSNLET00000006861	ENSGGOT00000013728	ENSCPOT00000014828	ENSDORT00000002130	ENSMMUT00000020700	ENSCJAT00000019201	ENSMUST00000081574	ENSMICT00000013994	ENSPPYT00000008411	ENSOPRT00000001710	ENSOCUT00000004620	ENSRNOT00000023090	ENSSTOT00000003900	ENSTBET00000002092	ENSTSYT00000004956
ENST00000515030	ENSOGAT00000001690	ENSPTRT00000030902	ENSBTAT00000005684	ENSNLET00000014186	ENSGGOT00000010015	ENSCPOT00000002768	ENSDORT00000005463	ENSMMUT00000017054	ENSCJAT00000056100	ENSMUST00000110372	ENSMICT00000013095	ENSPPYT00000017715	ENSOPRT00000013399	ENSOCUT00000005617	ENSRNOT00000014235	ENSSTOT00000009032	ENSTBET00000007085	ENSTSYT00000004506
ENST00000308666	ENSOGAT00000031123	ENSPTRT00000008930	ENSBTAT00000056538	ENSNLET00000022921	ENSGGOT00000009508	ENSCPOT00000010298	ENSDORT00000003158	ENSMMUT00000019658	ENSCJAT00000035026	ENSMUST00000069511	ENSMICT00000001010	ENSPPYT00000005235	ENSOPRT00000014014	ENSOCUT00000016171	ENSRNOT00000021064	ENSSTOT00000012760	ENSTBET00000005571	ENSTSYT00000000694
ENST00000369805	ENSOGAT00000015817	ENSPTRT00000002002	ENSBTAT00000002409	ENSNLET00000004355	ENSGGOT00000004988	ENSCPOT00000013988	ENSDORT00000000764	ENSMMUT00000011234	ENSCJAT00000006142	ENSMUST00000037375	ENSMICT00000005197	ENSPPYT00000001272	ENSOPRT00000008671	ENSOCUT00000009702	ENSRNOT00000025274	ENSSTOT00000007281	ENSTBET00000006230	ENSTSYT00000004906
ENST00000354666	ENSOGAT00000012193	ENSPTRT00000050492	ENSBTAT00000003245	ENSNLET00000000853	ENSGGOT00000007483	ENSCPOT00000008215	ENSDORT00000013467	ENSMMUT00000019521	ENSCJAT00000009735	ENSMUST00000021793	ENSMICT00000004250	ENSPPYT00000018876	ENSOPRT00000012692	ENSOCUT00000013015	ENSRNOT00000019756	ENSSTOT00000009242	ENSTBET00000006528	ENSTSYT00000000234
ENST00000378313	ENSOGAT00000029233	ENSPTRT00000020425	ENSBTAT00000043989	ENSNLET00000017736	ENSGGOT00000028842	ENSCPOT00000009177	ENSDORT00000006709	ENSMMUT00000024888	ENSCJAT00000028924	ENSMUST00000108379	ENSMICT00000009751	ENSPPYT00000011633	ENSOPRT00000002339	ENSOCUT00000009819	ENSRNOT00000064573	ENSSTOT00000015056	ENSTBET00000012513	ENSTSYT00000007558
ENST00000369130	ENSOGAT00000006276	ENSPTRT00000002310	ENSBTAT00000027499	ENSNLET00000012476	ENSGGOT00000034476	ENSCPOT00000002570	ENSDORT00000005012	ENSMMUT00000019205	ENSCJAT00000001561	ENSMUST00000015891	ENSMICT00000015195	ENSPPYT00000001097	ENSOPRT00000010901	ENSOCUT00000014360	ENSRNOT00000028751	ENSSTOT00000014242	ENSTBET00000010759	ENSTSYT00000008737
ENST00000525566	ENSOGAT00000006288	ENSPTRT00000009880	ENSBTAT00000018473	ENSNLET00000008382	ENSGGOT00000012806	ENSCPOT00000012057	ENSDORT00000004937	ENSMMUT00000042892	ENSCJAT00000028575	ENSMUST00000020484	ENSMICT00000011682	ENSPPYT00000005798	ENSOPRT00000009023	ENSOCUT00000003961	ENSRNOT00000013613	ENSSTOT00000015689	ENSTBET00000005930	ENSTSYT00000011402
ENST00000409272	ENSOGAT00000008776	ENSPTRT00000022113	ENSBTAT00000023171	ENSNLET00000021494	ENSGGOT00000007272	ENSCPOT00000009596	ENSDORT00000003117	ENSMMUT00000043565	ENSCJAT00000001373	ENSMUST00000171795	ENSMICT00000004928	ENSPPYT00000014468	ENSOPRT00000002198	ENSOCUT00000005472	ENSRNOT00000009660	ENSSTOT00000004865	ENSTBET00000001568	ENSTSYT00000004680
ENST00000233099	ENSOGAT00000001386	ENSPTRT00000022020	ENSBTAT00000019031	ENSNLET00000020623	ENSGGOT00000005041	ENSCPOT00000012295	ENSDORT00000002240	ENSMMUT00000020954	ENSCJAT00000007241	ENSMUST00000097281	ENSMICT00000003307	ENSPPYT00000014521	ENSOPRT00000008137	ENSOCUT00000007211	ENSRNOT00000035905	ENSSTOT00000013874	ENSTBET00000012649	ENSTSYT00000004005
ENST00000338179	ENSOGAT00000013725	ENSPTRT00000003726	ENSBTAT00000011125	ENSNLET00000017433	ENSGGOT00000015305	ENSCPOT00000006961	ENSDORT00000016098	ENSMMUT00000004915	ENSCJAT00000055058	ENSMUST00000005003	ENSMICT00000010081	ENSPPYT00000000190	ENSOPRT00000008851	ENSOCUT00000000285	ENSRNOT00000029841	ENSSTOT00000005938	ENSTBET00000017371	ENSTSYT00000008782
ENST00000295488	ENSOGAT00000014192	ENSPTRT00000030212	ENSBTAT00000024158	ENSNLET00000011645	ENSGGOT00000008639	ENSCPOT00000009263	ENSDORT00000012747	ENSMMUT00000021521	ENSCJAT00000010207	ENSMUST00000044684	ENSMICT00000010927	ENSPPYT00000017312	ENSOPRT00000004023	ENSOCUT00000013304	ENSRNOT00000002974	ENSSTOT00000007972	ENSTBET00000012434	ENSTSYT00000012914
ENST00000370598	ENSOGAT00000002657	ENSPTRT00000033875	ENSBTAT00000063222	ENSNLET00000000643	ENSGGOT00000015643	ENSCPOT00000000142	ENSDORT00000014588	ENSMMUT00000006891	ENSCJAT00000007984	ENSMUST00000135518	ENSMICT00000009824	ENSPPYT00000019488	ENSOPRT00000014137	ENSOCUT00000009475	ENSRNOT00000016194	ENSSTOT00000005485	ENSTBET00000017302	ENSTSYT00000013662
ENST00000319669	ENSOGAT00000017104	ENSPTRT00000012730	ENSBTAT00000038937	ENSNLET00000014501	ENSGGOT00000023237	ENSCPOT00000008890	ENSDORT00000003830	ENSMMUT00000013501	ENSCJAT00000041252	ENSMUST00000028834	ENSMICT00000010322	ENSPPYT00000007472	ENSOPRT00000003052	ENSOCUT00000006821	ENSRNOT00000006809	ENSSTOT00000016167	ENSTBET00000006027	ENSTSYT00000007861
ENST00000216951	ENSOGAT00000001807	ENSPTRT00000024950	ENSBTAT00000004559	ENSNLET00000012976	ENSGGOT00000006984	ENSCPOT00000004246	ENSDORT00000008574	ENSMMUT00000025208	ENSCJAT00000035161	ENSMUST00000079691	ENSMICT00000004948	ENSPPYT00000012700	ENSOPRT00000010985	ENSOCUT00000002267	ENSRNOT00000025657	ENSSTOT00000011637	ENSTBET00000010712	ENSTSYT00000000906
ENST00000284984	ENSOGAT00000024957	ENSPTRT00000025784	ENSBTAT00000000944	ENSNLET00000003573	ENSGGOT00000007389	ENSCPOT00000006512	ENSDORT00000015007	ENSMMUT00000018804	ENSCJAT00000027185	ENSMUST00000023610	ENSMICT00000012524	ENSPPYT00000013133	ENSOPRT00000006454	ENSOCUT00000016362	ENSRNOT00000002187	ENSSTOT00000008857	ENSTBET00000010048	ENSTSYT00000001283
ENST00000380962	ENSOGAT00000005096	ENSPTRT00000064603	ENSBTAT00000037425	ENSNLET00000015025	ENSGGOT00000023539	ENSCPOT00000012686	ENSDORT00000002358	ENSMMUT00000039436	ENSCJAT00000040742	ENSMUST00000033025	ENSMICT00000001574	ENSPPYT00000008482	ENSOPRT00000016985	ENSOCUT00000012389	ENSRNOT00000019640	ENSSTOT00000000415	ENSTBET00000015383	ENSTSYT00000001692
ENST00000380985	ENSOGAT00000013439	ENSPTRT00000031390	ENSBTAT00000022482	ENSNLET00000004630	ENSGGOT00000016583	ENSCPOT00000007994	ENSDORT00000008485	ENSMMUT00000023208	ENSCJAT00000030911	ENSMUST00000109315	ENSMICT00000015556	ENSPPYT00000018038	ENSOPRT00000007765	ENSOCUT00000009418	ENSRNOT00000015853	ENSSTOT00000003481	ENSTBET00000007788	ENSTSYT00000004546
ENST00000259455	ENSOGAT00000006117	ENSPTRT00000042100	ENSBTAT00000018355	ENSNLET00000021699	ENSGGOT00000000378	ENSCPOT00000006203	ENSDORT00000001687	ENSMMUT00000013735	ENSCJAT00000021686	ENSMUST00000107749	ENSMICT00000012871	ENSPPYT00000022669	ENSOPRT00000005602	ENSOCUT00000016530	ENSRNOT00000011573	ENSSTOT00000004140	ENSTBET00000001116	ENSTSYT00000005475
ENST00000273130	ENSOGAT00000016170	ENSPTRT00000027533	ENSBTAT00000019566	ENSNLET00000005529	ENSGGOT00000016047	ENSCPOT00000011725	ENSDORT00000009390	ENSMMUT00000007694	ENSCJAT00000022960	ENSMUST00000047404	ENSMICT00000007997	ENSPPYT00000016328	ENSOPRT00000004855	ENSOCUT00000009533	ENSRNOT00000014532	ENSSTOT00000012614	ENSTBET00000009328	ENSTSYT00000013089
ENST00000260118	ENSOGAT00000015826	ENSPTRT00000037585	ENSBTAT00000009917	ENSNLET00000004891	ENSGGOT00000007775	ENSCPOT00000007753	ENSDORT00000015118	ENSMMUT00000012791	ENSCJAT00000017806	ENSMUST00000029904	ENSMICT00000000304	ENSPPYT00000021722	ENSOPRT00000011803	ENSOCUT00000003032	ENSRNOT00000009758	ENSSTOT00000013288	ENSTBET00000005186	ENSTSYT00000005499
ENST00000445915	ENSOGAT00000013979	ENSPTRT00000065370	ENSBTAT00000044762	ENSNLET00000012587	ENSGGOT00000006314	ENSCPOT00000008536	ENSDORT00000013164	ENSMMUT00000012251	ENSCJAT00000013690	ENSMUST00000102875	ENSMICT00000006510	ENSPPYT00000014355	ENSOPRT00000016185	ENSOCUT00000002137	ENSRNOT00000067668	ENSSTOT00000000788	ENSTBET00000012089	ENSTSYT00000014243
ENST00000494664	ENSOGAT00000032201	ENSPTRT00000028499	ENSBTAT00000016702	ENSNLET00000006304	ENSGGOT00000023230	ENSCPOT00000008874	ENSDORT00000002926	ENSMMUT00000016830	ENSCJAT00000017408	ENSMUST00000002925	ENSMICT00000007984	ENSPPYT00000015718	ENSOPRT00000007613	ENSOCUT00000009501	ENSRNOT00000004023	ENSSTOT00000003909	ENSTBET00000013908	ENSTSYT00000006176
ENST00000511217	ENSOGAT00000010032	ENSPTRT00000046209	ENSBTAT00000008898	ENSNLET00000012532	ENSGGOT00000008288	ENSCPOT00000012287	ENSDORT00000013988	ENSMMUT00000025839	ENSCJAT00000041968	ENSMUST00000072008	ENSMICT00000003255	ENSPPYT00000018508	ENSOPRT00000003046	ENSOCUT00000015664	ENSRNOT00000025408	ENSSTOT00000002436	ENSTBET00000002826	ENSTSYT00000008664
ENST00000204726	ENSOGAT00000006668	ENSPTRT00000010424	ENSBTAT00000012863	ENSNLET00000015847	ENSGGOT00000041037	ENSCPOT00000001516	ENSDORT00000013208	ENSMMUT00000045319	ENSCJAT00000025293	ENSMUST00000031477	ENSMICT00000004004	ENSPPYT00000006094	ENSOPRT00000006371	ENSOCUT00000008427	ENSRNOT00000056656	ENSSTOT00000006720	ENSTBET00000011000	ENSTSYT00000007192
ENST00000278826	ENSOGAT00000016134	ENSPTRT00000006975	ENSBTAT00000028789	ENSNLET00000003157	ENSGGOT00000004098	ENSCPOT00000025513	ENSDORT00000000138	ENSMMUT00000031469	ENSCJAT00000037833	ENSMUST00000025568	ENSMICT00000013769	ENSPPYT00000003812	ENSOPRT00000008333	ENSOCUT00000001677	ENSRNOT00000028092	ENSSTOT00000007674	ENSTBET00000010449	ENSTSYT00000011211
ENST00000397292	ENSOGAT00000006322	ENSPTRT00000038541	ENSBTAT00000017298	ENSNLET00000020842	ENSGGOT00000016695	ENSCPOT00000013094	ENSDORT00000002789	ENSMMUT00000008493	ENSCJAT00000015564	ENSMUST00000107107	ENSMICT00000007606	ENSPPYT00000022340	ENSOPRT00000009704	ENSOCUT00000015473	ENSRNOT00000010621	ENSSTOT00000013248	ENSTBET00000001495	ENSTSYT00000005934
ENST00000339180	ENSOGAT00000008720	ENSPTRT00000011317	ENSBTAT00000012794	ENSNLET00000019189	ENSGGOT00000017183	ENSCPOT00000007096	ENSDORT00000013238	ENSMMUT00000020693	ENSCJAT00000015350	ENSMUST00000037814	ENSMICT00000010034	ENSPPYT00000006700	ENSOPRT00000016510	ENSOCUT00000011028	ENSRNOT00000022664	ENSSTOT00000002571	ENSTBET00000001006	ENSTSYT00000006244
ENST00000505397	ENSOGAT00000002515	ENSPTRT00000030198	ENSBTAT00000030399	ENSNLET00000011440	ENSGGOT00000004186	ENSCPOT00000001151	ENSDORT00000002968	ENSMMUT00000011900	ENSCJAT00000009915	ENSMUST00000046154	ENSMICT00000008558	ENSPPYT00000017306	ENSOPRT00000012596	ENSOCUT00000012515	ENSRNOT00000003006	ENSSTOT00000012485	ENSTBET00000017492	ENSTSYT00000005314
ENST00000260645	ENSOGAT00000012796	ENSPTRT00000022102	ENSBTAT00000021768	ENSNLET00000021370	ENSGGOT00000027297	ENSCPOT00000021114	ENSDORT00000012230	ENSMMUT00000011298	ENSCJAT00000001550	ENSMUST00000066175	ENSMICT00000010466	ENSPPYT00000014474	ENSOPRT00000016753	ENSOCUT00000005301	ENSRNOT00000007174	ENSSTOT00000011170	ENSTBET00000013853	ENSTSYT00000013195
ENST00000235090	ENSOGAT00000028890	ENSPTRT00000002065	ENSBTAT00000018753	ENSNLET00000005037	ENSGGOT00000005238	ENSCPOT00000025424	ENSDORT00000013349	ENSMMUT00000004682	ENSCJAT00000025929	ENSMUST00000010278	ENSMICT00000011980	ENSPPYT00000001242	ENSOPRT00000003848	ENSOCUT00000007800	ENSRNOT00000022571	ENSSTOT00000000477	ENSTBET00000010324	ENSTSYT00000013918
ENST00000368786	ENSOGAT00000010901	ENSPTRT00000005791	ENSBTAT00000007615	ENSNLET00000000894	ENSGGOT00000011460	ENSCPOT00000004885	ENSDORT00000012150	ENSMMUT00000011938	ENSCJAT00000024147	ENSMUST00000106145	ENSMICT00000006185	ENSPPYT00000003322	ENSOPRT00000007324	ENSOCUT00000004570	ENSRNOT00000024279	ENSSTOT00000011818	ENSTBET00000002002	ENSTSYT00000004150
ENST00000360943	ENSOGAT00000001267	ENSPTRT00000042637	ENSBTAT00000043423	ENSNLET00000003470	ENSGGOT00000034731	ENSCPOT00000023010	ENSDORT00000009258	ENSMMUT00000029657	ENSCJAT00000014407	ENSMUST00000100528	ENSMICT00000013287	ENSPPYT00000010399	ENSOPRT00000010176	ENSOCUT00000009157	ENSRNOT00000055500	ENSSTOT00000000795	ENSTBET00000003818	ENSTSYT00000004567
ENST00000332517	ENSOGAT00000013174	ENSPTRT00000030953	ENSBTAT00000011393	ENSNLET00000014782	ENSGGOT00000002257	ENSCPOT00000010841	ENSDORT00000014160	ENSMMUT00000023415	ENSCJAT00000021065	ENSMUST00000059692	ENSMICT00000005708	ENSPPYT00000017747	ENSOPRT00000013463	ENSOCUT00000014333	ENSRNOT00000019474	ENSSTOT00000016318	ENSTBET00000004862	ENSTSYT00000014319
ENST00000334571	ENSOGAT00000006410	ENSPTRT00000011967	ENSBTAT00000027093	ENSNLET00000020573	ENSGGOT00000014406	ENSCPOT00000009697	ENSDORT00000009297	ENSMMUT00000015579	ENSCJAT00000040031	ENSMUST00000021661	ENSMICT00000003008	ENSPPYT00000007064	ENSOPRT00000002507	ENSOCUT00000006797	ENSRNOT00000014913	ENSSTOT00000016442	ENSTBET00000008327	ENSTSYT00000005635
ENST00000006658	ENSOGAT00000009491	ENSPTRT00000017248	ENSBTAT00000005441	ENSNLET00000009869	ENSGGOT00000003400	ENSCPOT00000025575	ENSDORT00000009128	ENSMMUT00000024250	ENSCJAT00000036785	ENSMUST00000041705	ENSMICT00000009770	ENSPPYT00000009677	ENSOPRT00000013397	ENSOCUT00000002727	ENSRNOT00000004377	ENSSTOT00000004110	ENSTBET00000001886	ENSTSYT00000012859
ENST00000509534	ENSOGAT00000011871	ENSPTRT00000032000	ENSBTAT00000047926	ENSNLET00000011615	ENSGGOT00000012869	ENSCPOT00000014420	ENSDORT00000006159	ENSMMUT00000041035	ENSCJAT00000013397	ENSMUST00000025215	ENSMICT00000013040	ENSPPYT00000018415	ENSOPRT00000016493	ENSOCUT00000007755	ENSRNOT00000026895	ENSSTOT00000009062	ENSTBET00000016495	ENSTSYT00000008628
ENST00000266679	ENSOGAT00000031886	ENSPTRT00000009565	ENSBTAT00000050636	ENSNLET00000004852	ENSGGOT00000029079	ENSCPOT00000015348	ENSDORT00000002950	ENSMMUT00000004145	ENSCJAT00000003449	ENSMUST00000069168	ENSMICT00000007384	ENSPPYT00000005629	ENSOPRT00000005196	ENSOCUT00000013501	ENSRNOT00000008193	ENSSTOT00000014133	ENSTBET00000015364	ENSTSYT00000008593
ENST00000258198	ENSOGAT00000026240	ENSPTRT00000015122	ENSBTAT00000000581	ENSNLET00000005097	ENSGGOT00000005245	ENSCPOT00000015559	ENSDORT00000010219	ENSMMUT00000015761	ENSCJAT00000032678	ENSMUST00000041769	ENSMICT00000005777	ENSPPYT00000008732	ENSOPRT00000004111	ENSOCUT00000006143	ENSRNOT00000034453	ENSSTOT00000007744	ENSTBET00000015228	ENSTSYT00000012519
ENST00000355423	ENSOGAT00000001462	ENSPTRT00000049320	ENSBTAT00000015560	ENSNLET00000001587	ENSGGOT00000009542	ENSCPOT00000012774	ENSDORT00000004092	ENSMMUT00000030451	ENSCJAT00000027551	ENSMUST00000140991	ENSMICT00000007549	ENSPPYT00000010080	ENSOPRT00000012703	ENSOCUT00000016220	ENSRNOT00000007362	ENSSTOT00000012995	ENSTBET00000015618	ENSTSYT00000009666
ENST00000242067	ENSOGAT00000005788	ENSPTRT00000035246	ENSBTAT00000008587	ENSNLET00000019688	ENSGGOT00000008156	ENSCPOT00000000941	ENSDORT00000004826	ENSMMUT00000004096	ENSCJAT00000017469	ENSMUST00000147405	ENSMICT00000017005	ENSPPYT00000020564	ENSOPRT00000002974	ENSOCUT00000000102	ENSRNOT00000060758	ENSSTOT00000009870	ENSTBET00000000191	ENSTSYT00000004978
ENST00000546305	ENSOGAT00000013873	ENSPTRT00000066948	ENSBTAT00000034795	ENSNLET00000008365	ENSGGOT00000022351	ENSCPOT00000014853	ENSDORT00000011322	ENSMMUT00000002022	ENSCJAT00000001591	ENSMUST00000081746	ENSMICT00000011871	ENSPPYT00000007658	ENSOPRT00000010324	ENSOCUT00000007681	ENSRNOT00000065520	ENSSTOT00000015591	ENSTBET00000016248	ENSTSYT00000002646
ENST00000399298	ENSOGAT00000007351	ENSPTRT00000004728	ENSBTAT00000019258	ENSNLET00000015455	ENSGGOT00000003613	ENSCPOT00000001078	ENSDORT00000011158	ENSMMUT00000020382	ENSCJAT00000013815	ENSMUST00000080995	ENSMICT00000010848	ENSPPYT00000002904	ENSOPRT00000009741	ENSOCUT00000010984	ENSRNOT00000038095	ENSSTOT00000002157	ENSTBET00000003246	ENSTSYT00000004039
ENST00000470972	ENSOGAT00000005535	ENSPTRT00000056387	ENSBTAT00000009435	ENSNLET00000022031	ENSGGOT00000030769	ENSCPOT00000015438	ENSDORT00000009385	ENSMMUT00000021065	ENSCJAT00000057228	ENSMUST00000107651	ENSMICT00000012374	ENSPPYT00000022706	ENSOPRT00000010427	ENSOCUT00000005948	ENSRNOT00000024354	ENSSTOT00000012385	ENSTBET00000016682	ENSTSYT00000000727
ENST00000325192	ENSOGAT00000006922	ENSPTRT00000011547	ENSBTAT00000050440	ENSNLET00000005468	ENSGGOT00000022321	ENSCPOT00000024190	ENSDORT00000010215	ENSMMUT00000024851	ENSCJAT00000031273	ENSMUST00000058135	ENSMICT00000000629	ENSPPYT00000006821	ENSOPRT00000016703	ENSOCUT00000008731	ENSRNOT00000034756	ENSSTOT00000009364	ENSTBET00000000240	ENSTSYT00000012457
ENST00000193391	ENSOGAT00000029130	ENSPTRT00000028362	ENSBTAT00000020801	ENSNLET00000003940	ENSGGOT00000026793	ENSCPOT00000008417	ENSDORT00000014599	ENSMMUT00000047078	ENSCJAT00000000958	ENSMUST00000160116	ENSMICT00000016557	ENSPPYT00000015805	ENSOPRT00000004021	ENSOCUT00000011113	ENSRNOT00000044900	ENSSTOT00000001290	ENSTBET00000012749	ENSTSYT00000013475
ENST00000289575	ENSOGAT00000011549	ENSPTRT00000007595	ENSBTAT00000020716	ENSNLET00000021597	ENSGGOT00000007366	ENSCPOT00000015199	ENSDORT00000006522	ENSMMUT00000012766	ENSCJAT00000059373	ENSMUST00000032985	ENSMICT00000011167	ENSPPYT00000004383	ENSOPRT00000007458	ENSOCUT00000014225	ENSRNOT00000024281	ENSSTOT00000012656	ENSTBET00000005355	ENSTSYT00000014300
ENST00000367569	ENSOGAT00000012155	ENSPTRT00000034482	ENSBTAT00000012920	ENSNLET00000024332	ENSGGOT00000001546	ENSCPOT00000012554	ENSDORT00000005837	ENSMMUT00000013168	ENSCJAT00000043249	ENSMUST00000105139	ENSMICT00000014912	ENSPPYT00000019866	ENSOPRT00000015096	ENSOCUT00000008747	ENSRNOT00000019994	ENSSTOT00000003339	ENSTBET00000003048	ENSTSYT00000012219
ENST00000405785	ENSOGAT00000008222	ENSPTRT00000034992	ENSBTAT00000018305	ENSNLET00000020564	ENSGGOT00000000657	ENSCPOT00000011857	ENSDORT00000008158	ENSMMUT00000001222	ENSCJAT00000001273	ENSMUST00000040017	ENSMICT00000017738	ENSPPYT00000020726	ENSOPRT00000015172	ENSOCUT00000000466	ENSRNOT00000010952	ENSSTOT00000014628	ENSTBET00000010633	ENSTSYT00000010697
ENST00000369151	ENSOGAT00000006774	ENSPTRT00000005688	ENSBTAT00000030962	ENSNLET00000021199	ENSGGOT00000001459	ENSCPOT00000011003	ENSDORT00000014803	ENSMMUT00000022980	ENSCJAT00000038408	ENSMUST00000081790	ENSMICT00000007498	ENSPPYT00000003253	ENSOPRT00000013838	ENSOCUT00000015517	ENSRNOT00000013289	ENSSTOT00000007075	ENSTBET00000014783	ENSTSYT00000008475
ENST00000340139	ENSOGAT00000005503	ENSPTRT00000030408	ENSBTAT00000047799	ENSNLET00000016762	ENSGGOT00000013546	ENSCPOT00000009212	ENSDORT00000009963	ENSMMUT00000016419	ENSCJAT00000030656	ENSMUST00000029650	ENSMICT00000010756	ENSPPYT00000017406	ENSOPRT00000001124	ENSOCUT00000011321	ENSRNOT00000016066	ENSSTOT00000003121	ENSTBET00000009560	ENSTSYT00000007455
ENST00000396087	ENSOGAT00000014592	ENSPTRT00000049537	ENSBTAT00000024536	ENSNLET00000022724	ENSGGOT00000007001	ENSCPOT00000019869	ENSDORT00000004183	ENSMMUT00000023807	ENSCJAT00000013279	ENSMUST00000064395	ENSMICT00000017104	ENSPPYT00000004078	ENSOPRT00000012926	ENSOCUT00000007605	ENSRNOT00000046529	ENSSTOT00000015551	ENSTBET00000004251	ENSTSYT00000002850
ENST00000377197	ENSOGAT00000009767	ENSPTRT00000056533	ENSBTAT00000047514	ENSNLET00000000896	ENSGGOT00000005863	ENSCPOT00000006705	ENSDORT00000000882	ENSMMUT00000022265	ENSCJAT00000003121	ENSMUST00000035849	ENSMICT00000001040	ENSPPYT00000022476	ENSOPRT00000000966	ENSOCUT00000008782	ENSRNOT00000054776	ENSSTOT00000012360	ENSTBET00000000933	ENSTSYT00000011111
ENST00000308497	ENSOGAT00000004803	ENSPTRT00000030889	ENSBTAT00000011697	ENSNLET00000013774	ENSGGOT00000013962	ENSCPOT00000010572	ENSDORT00000014878	ENSMMUT00000006918	ENSCJAT00000011061	ENSMUST00000079195	ENSMICT00000015475	ENSPPYT00000017705	ENSOPRT00000005352	ENSOCUT00000009716	ENSRNOT00000068562	ENSSTOT00000016500	ENSTBET00000012264	ENSTSYT00000008548
ENST00000259365	ENSOGAT00000009562	ENSPTRT00000043078	ENSBTAT00000019585	ENSNLET00000021547	ENSGGOT00000001379	ENSCPOT00000000494	ENSDORT00000013646	ENSMMUT00000017196	ENSCJAT00000021049	ENSMUST00000107773	ENSMICT00000012658	ENSPPYT00000022656	ENSOPRT00000010773	ENSOCUT00000013831	ENSRNOT00000013228	ENSSTOT00000002658	ENSTBET00000002961	ENSTSYT00000011884
ENST00000379115	ENSOGAT00000007861	ENSPTRT00000049726	ENSBTAT00000005994	ENSNLET00000022827	ENSGGOT00000024041	ENSCPOT00000007317	ENSDORT00000000254	ENSMMUT00000023620	ENSCJAT00000011623	ENSMUST00000111086	ENSMICT00000004089	ENSPPYT00000004036	ENSOPRT00000001674	ENSOCUT00000011073	ENSRNOT00000006302	ENSSTOT00000003207	ENSTBET00000004747	ENSTSYT00000011965
ENST00000271331	ENSOGAT00000009196	ENSPTRT00000002036	ENSBTAT00000013959	ENSNLET00000004620	ENSGGOT00000008453	ENSCPOT00000007607	ENSDORT00000002157	ENSMMUT00000026994	ENSCJAT00000006597	ENSMUST00000049852	ENSMICT00000005266	ENSPPYT00000001257	ENSOPRT00000004289	ENSOCUT00000012594	ENSRNOT00000024519	ENSSTOT00000005537	ENSTBET00000000964	ENSTSYT00000007764
ENST00000245157	ENSOGAT00000014905	ENSPTRT00000014991	ENSBTAT00000032389	ENSNLET00000002798	ENSGGOT00000016110	ENSCPOT00000003752	ENSDORT00000005133	ENSMMUT00000018631	ENSCJAT00000024648	ENSMUST00000034206	ENSMICT00000003879	ENSPPYT00000008664	ENSOPRT00000006712	ENSOCUT00000017261	ENSRNOT00000026076	ENSSTOT00000005893	ENSTBET00000008277	ENSTSYT00000014606
ENST00000369301	ENSOGAT00000008937	ENSPTRT00000005643	ENSBTAT00000004806	ENSNLET00000020622	ENSGGOT00000015245	ENSCPOT00000014358	ENSDORT00000009795	ENSMMUT00000023394	ENSCJAT00000037703	ENSMUST00000071423	ENSMICT00000016135	ENSPPYT00000003216	ENSOPRT00000005310	ENSOCUT00000004925	ENSRNOT00000022788	ENSSTOT00000001254	ENSTBET00000014004	ENSTSYT00000000333
ENST00000507469	ENSOGAT00000003113	ENSPTRT00000003202	ENSBTAT00000033964	ENSNLET00000022999	ENSGGOT00000002361	ENSCPOT00000008986	ENSDORT00000007332	ENSMMUT00000044871	ENSCJAT00000010098	ENSMUST00000111836	ENSMICT00000007153	ENSPPYT00000000505	ENSOPRT00000013978	ENSOCUT00000005891	ENSRNOT00000044235	ENSSTOT00000000403	ENSTBET00000003249	ENSTSYT00000002684
ENST00000231009	ENSOGAT00000007119	ENSPTRT00000031289	ENSBTAT00000006807	ENSNLET00000001449	ENSGGOT00000029293	ENSCPOT00000008144	ENSDORT00000012851	ENSMMUT00000028382	ENSCJAT00000003095	ENSMUST00000038212	ENSMICT00000014756	ENSPPYT00000017983	ENSOPRT00000010805	ENSOCUT00000008870	ENSRNOT00000014319	ENSSTOT00000015207	ENSTBET00000001599	ENSTSYT00000003825
ENST00000231420	ENSOGAT00000001472	ENSPTRT00000031163	ENSBTAT00000010113	ENSNLET00000018480	ENSGGOT00000006609	ENSCPOT00000008843	ENSDORT00000009372	ENSMMUT00000008823	ENSCJAT00000058240	ENSMUST00000110542	ENSMICT00000005688	ENSPPYT00000017891	ENSOPRT00000012908	ENSOCUT00000005265	ENSRNOT00000024035	ENSSTOT00000008426	ENSTBET00000012125	ENSTSYT00000009321
ENST00000262207	ENSOGAT00000005764	ENSPTRT00000037690	ENSBTAT00000005784	ENSNLET00000002409	ENSGGOT00000024644	ENSCPOT00000002903	ENSDORT00000007131	ENSMMUT00000015433	ENSCJAT00000001015	ENSMUST00000159958	ENSMICT00000003385	ENSPPYT00000021799	ENSOPRT00000010729	ENSOCUT00000009620	ENSRNOT00000024199	ENSSTOT00000008682	ENSTBET00000004523	ENSTSYT00000014198
ENST00000344921	ENSOGAT00000009934	ENSPTRT00000049584	ENSBTAT00000013170	ENSNLET00000000682	ENSGGOT00000041123	ENSCPOT00000014760	ENSDORT00000002679	ENSMMUT00000012729	ENSCJAT00000021013	ENSMUST00000121304	ENSMICT00000006781	ENSPPYT00000013504	ENSOPRT00000012142	ENSOCUT00000012671	ENSRNOT00000029458	ENSSTOT00000012413	ENSTBET00000017240	ENSTSYT00000008252
ENST00000039989	ENSOGAT00000013646	ENSPTRT00000006622	ENSBTAT00000061051	ENSNLET00000022915	ENSGGOT00000029270	ENSCPOT00000013658	ENSDORT00000002398	ENSMMUT00000001214	ENSCJAT00000022012	ENSMUST00000111237	ENSMICT00000014616	ENSPPYT00000003986	ENSOPRT00000009492	ENSOCUT00000014682	ENSRNOT00000056957	ENSSTOT00000009336	ENSTBET00000013800	ENSTSYT00000011009
ENST00000262507	ENSOGAT00000016868	ENSPTRT00000015036	ENSBTAT00000002419	ENSNLET00000003849	ENSGGOT00000012453	ENSCPOT00000004598	ENSDORT00000000080	ENSMMUT00000028817	ENSCJAT00000023393	ENSMUST00000034234	ENSMICT00000004667	ENSPPYT00000008689	ENSOPRT00000006963	ENSOCUT00000014972	ENSRNOT00000021716	ENSSTOT00000015149	ENSTBET00000009269	ENSTSYT00000003645
ENST00000506030	ENSOGAT00000002135	ENSPTRT00000031396	ENSBTAT00000000533	ENSNLET00000004833	ENSGGOT00000026565	ENSCPOT00000007459	ENSDORT00000005291	ENSMMUT00000010767	ENSCJAT00000031013	ENSMUST00000169083	ENSMICT00000007596	ENSPPYT00000018039	ENSOPRT00000012095	ENSOCUT00000003801	ENSRNOT00000061017	ENSSTOT00000003457	ENSTBET00000008478	ENSTSYT00000010928
ENST00000300574	ENSOGAT00000006158	ENSPTRT00000015716	ENSBTAT00000007443	ENSNLET00000000907	ENSGGOT00000006781	ENSCPOT00000006946	ENSDORT00000004561	ENSMMUT00000042451	ENSCJAT00000003993	ENSMUST00000017920	ENSMICT00000003830	ENSPPYT00000009112	ENSOPRT00000010115	ENSOCUT00000002846	ENSRNOT00000006407	ENSSTOT00000005095	ENSTBET00000002035	ENSTSYT00000009498
ENST00000372479	ENSOGAT00000009285	ENSPTRT00000041259	ENSBTAT00000001264	ENSNLET00000017676	ENSGGOT00000006616	ENSCPOT00000022863	ENSDORT00000008077	ENSMMUT00000032592	ENSCJAT00000015557	ENSMUST00000033810	ENSMICT00000009745	ENSPPYT00000024038	ENSOPRT00000000221	ENSOCUT00000015927	ENSRNOT00000068341	ENSSTOT00000001601	ENSTBET00000003311	ENSTSYT00000006947
ENST00000274031	ENSOGAT00000012219	ENSPTRT00000074719	ENSBTAT00000004658	ENSNLET00000006447	ENSGGOT00000011817	ENSCPOT00000024892	ENSDORT00000004857	ENSMMUT00000002595	ENSCJAT00000005908	ENSMUST00000037141	ENSMICT00000007052	ENSPPYT00000017520	ENSOPRT00000009372	ENSOCUT00000009365	ENSRNOT00000017736	ENSSTOT00000004415	ENSTBET00000010468	ENSTSYT00000005206
ENST00000350777	ENSOGAT00000007309	ENSPTRT00000008114	ENSBTAT00000019990	ENSNLET00000009435	ENSGGOT00000008121	ENSCPOT00000006553	ENSDORT00000001981	ENSMMUT00000020873	ENSCJAT00000027120	ENSMUST00000034629	ENSMICT00000002729	ENSPPYT00000004713	ENSOPRT00000016331	ENSOCUT00000010809	ENSRNOT00000012868	ENSSTOT00000014460	ENSTBET00000001410	ENSTSYT00000012227
ENST00000419934	ENSOGAT00000015068	ENSPTRT00000005277	ENSBTAT00000004744	ENSNLET00000016949	ENSGGOT00000006061	ENSCPOT00000001495	ENSDORT00000008264	ENSMMUT00000023448	ENSCJAT00000031632	ENSMUST00000119316	ENSMICT00000010859	ENSPPYT00000003021	ENSOPRT00000009971	ENSOCUT00000011114	ENSRNOT00000019521	ENSSTOT00000005629	ENSTBET00000002811	ENSTSYT00000009456
ENST00000379682	ENSOGAT00000008175	ENSPTRT00000040310	ENSBTAT00000063419	ENSNLET00000012618	ENSGGOT00000009492	ENSCPOT00000008773	ENSDORT00000007989	ENSMMUT00000006706	ENSCJAT00000010490	ENSMUST00000112464	ENSMICT00000004103	ENSPPYT00000023530	ENSOPRT00000000094	ENSOCUT00000015296	ENSRNOT00000006867	ENSSTOT00000013884	ENSTBET00000016898	ENSTSYT00000004802
ENST00000295550	ENSOGAT00000016059	ENSPTRT00000024248	ENSBTAT00000018479	ENSNLET00000014011	ENSGGOT00000002484	ENSCPOT00000011437	ENSDORT00000016705	ENSMMUT00000019737	ENSCJAT00000004273	ENSMUST00000056925	ENSMICT00000003444	ENSPPYT00000015479	ENSOPRT00000015166	ENSOCUT00000007478	ENSRNOT00000026707	ENSSTOT00000002714	ENSTBET00000016510	ENSTSYT00000002011
ENST00000333479	ENSOGAT00000001272	ENSPTRT00000010251	ENSBTAT00000025676	ENSNLET00000002753	ENSGGOT00000017142	ENSCPOT00000001445	ENSDORT00000016094	ENSMMUT00000010566	ENSCJAT00000040203	ENSMUST00000031366	ENSMICT00000000333	ENSPPYT00000005997	ENSOPRT00000013885	ENSOCUT00000013829	ENSRNOT00000047692	ENSSTOT00000001138	ENSTBET00000007893	ENSTSYT00000013363
ENST00000356107	ENSOGAT00000005274	ENSPTRT00000012641	ENSBTAT00000038824	ENSNLET00000015875	ENSGGOT00000028548	ENSCPOT00000011029	ENSDORT00000013418	ENSMMUT00000040935	ENSCJAT00000033983	ENSMUST00000102592	ENSMICT00000006885	ENSPPYT00000007418	ENSOPRT00000000142	ENSOCUT00000004340	ENSRNOT00000014988	ENSSTOT00000011149	ENSTBET00000015157	ENSTSYT00000005878
ENST00000485050	ENSOGAT00000015074	ENSPTRT00000058186	ENSBTAT00000054516	ENSNLET00000006033	ENSGGOT00000011877	ENSCPOT00000014130	ENSDORT00000004310	ENSMMUT00000002764	ENSCJAT00000018026	ENSMUST00000171199	ENSMICT00000001627	ENSPPYT00000015734	ENSOPRT00000014423	ENSOCUT00000002603	ENSRNOT00000000317	ENSSTOT00000000303	ENSTBET00000003355	ENSTSYT00000001899
ENST00000458580	ENSOGAT00000013099	ENSPTRT00000012794	ENSBTAT00000011011	ENSNLET00000004403	ENSGGOT00000004619	ENSCPOT00000010668	ENSDORT00000012078	ENSMMUT00000019638	ENSCJAT00000040811	ENSMUST00000171024	ENSMICT00000014343	ENSPPYT00000007511	ENSOPRT00000016435	ENSOCUT00000013815	ENSRNOT00000038365	ENSSTOT00000014980	ENSTBET00000003078	ENSTSYT00000004086
ENST00000348367	ENSOGAT00000008028	ENSPTRT00000005611	ENSBTAT00000015811	ENSNLET00000019994	ENSGGOT00000017029	ENSCPOT00000015771	ENSDORT00000016523	ENSMMUT00000010528	ENSCJAT00000037132	ENSMUST00000086868	ENSMICT00000007311	ENSPPYT00000003197	ENSOPRT00000004638	ENSOCUT00000007894	ENSRNOT00000047903	ENSSTOT00000009554	ENSTBET00000002830	ENSTSYT00000009805
ENST00000281623	ENSOGAT00000002040	ENSPTRT00000031241	ENSBTAT00000022583	ENSNLET00000021238	ENSGGOT00000005532	ENSCPOT00000013103	ENSDORT00000016320	ENSMMUT00000005623	ENSCJAT00000002268	ENSMUST00000022791	ENSMICT00000006130	ENSPPYT00000017940	ENSOPRT00000004678	ENSOCUT00000009247	ENSRNOT00000020925	ENSSTOT00000008298	ENSTBET00000010557	ENSTSYT00000005418
ENST00000305949	ENSOGAT00000014291	ENSPTRT00000038008	ENSBTAT00000008906	ENSNLET00000006572	ENSGGOT00000009916	ENSCPOT00000005859	ENSDORT00000013352	ENSMMUT00000003708	ENSCJAT00000010338	ENSMUST00000022998	ENSMICT00000014658	ENSPPYT00000021990	ENSOPRT00000015750	ENSOCUT00000016200	ENSRNOT00000068012	ENSSTOT00000008758	ENSTBET00000005101	ENSTSYT00000006037
ENST00000553891	ENSOGAT00000012393	ENSPTRT00000011927	ENSBTAT00000014664	ENSNLET00000003417	ENSGGOT00000005195	ENSCPOT00000014021	ENSDORT00000015228	ENSMMUT00000007241	ENSCJAT00000028208	ENSMUST00000048319	ENSMICT00000015513	ENSPPYT00000007048	ENSOPRT00000016734	ENSOCUT00000008398	ENSRNOT00000011662	ENSSTOT00000011119	ENSTBET00000001677	ENSTSYT00000014297
ENST00000524871	ENSOGAT00000004711	ENSPTRT00000007835	ENSBTAT00000027270	ENSNLET00000009124	ENSGGOT00000016861	ENSCPOT00000011428	ENSDORT00000008459	ENSMMUT00000023514	ENSCJAT00000001034	ENSMUST00000160216	ENSMICT00000015973	ENSPPYT00000004517	ENSOPRT00000004925	ENSOCUT00000015049	ENSRNOT00000009418	ENSSTOT00000003758	ENSTBET00000001616	ENSTSYT00000001636
ENST00000288532	ENSOGAT00000008521	ENSPTRT00000010161	ENSBTAT00000020929	ENSNLET00000020028	ENSGGOT00000008834	ENSCPOT00000006890	ENSDORT00000006156	ENSMMUT00000028984	ENSCJAT00000002215	ENSMUST00000040421	ENSMICT00000006370	ENSPPYT00000005956	ENSOPRT00000004259	ENSOCUT00000014867	ENSRNOT00000001546	ENSSTOT00000012560	ENSTBET00000009253	ENSTSYT00000007065
ENST00000378912	ENSOGAT00000016050	ENSPTRT00000015316	ENSBTAT00000017266	ENSNLET00000020520	ENSGGOT00000005980	ENSCPOT00000015182	ENSDORT00000014058	ENSMMUT00000004495	ENSCJAT00000028586	ENSMUST00000041382	ENSMICT00000001345	ENSPPYT00000008861	ENSOPRT00000009774	ENSOCUT00000004124	ENSRNOT00000048012	ENSSTOT00000006999	ENSTBET00000001920	ENSTSYT00000012119
ENST00000378623	ENSOGAT00000011987	ENSPTRT00000006688	ENSBTAT00000043997	ENSNLET00000018208	ENSGGOT00000001034	ENSCPOT00000007142	ENSDORT00000001794	ENSMMUT00000044124	ENSCJAT00000039203	ENSMUST00000028689	ENSMICT00000007000	ENSPPYT00000003951	ENSOPRT00000009390	ENSOCUT00000002059	ENSRNOT00000021353	ENSSTOT00000005283	ENSTBET00000013425	ENSTSYT00000010802
ENST00000382873	ENSOGAT00000001513	ENSPTRT00000062330	ENSBTAT00000008384	ENSNLET00000010333	ENSGGOT00000005583	ENSCPOT00000013351	ENSDORT00000002793	ENSMMUT00000031752	ENSCJAT00000007497	ENSMUST00000025484	ENSMICT00000003069	ENSPPYT00000010720	ENSOPRT00000007408	ENSOCUT00000003019	ENSRNOT00000024328	ENSSTOT00000002144	ENSTBET00000015039	ENSTSYT00000005445
ENST00000558653	ENSOGAT00000013295	ENSPTRT00000013025	ENSBTAT00000008144	ENSNLET00000007852	ENSGGOT00000024460	ENSCPOT00000003505	ENSDORT00000011656	ENSMMUT00000040599	ENSCJAT00000002936	ENSMUST00000170908	ENSMICT00000005001	ENSPPYT00000007628	ENSOPRT00000002938	ENSOCUT00000016782	ENSRNOT00000012966	ENSSTOT00000005410	ENSTBET00000008024	ENSTSYT00000008179
ENST00000326427	ENSOGAT00000004748	ENSPTRT00000024137	ENSBTAT00000023734	ENSNLET00000017705	ENSGGOT00000002246	ENSCPOT00000012835	ENSDORT00000013471	ENSMMUT00000008702	ENSCJAT00000059871	ENSMUST00000027425	ENSMICT00000004120	ENSPPYT00000015418	ENSOPRT00000015870	ENSOCUT00000011359	ENSRNOT00000023535	ENSSTOT00000009339	ENSTBET00000011931	ENSTSYT00000010900
ENST00000560031	ENSOGAT00000013289	ENSPTRT00000067544	ENSBTAT00000005236	ENSNLET00000007832	ENSGGOT00000002856	ENSCPOT00000003497	ENSDORT00000011650	ENSMMUT00000040603	ENSCJAT00000002980	ENSMUST00000094604	ENSMICT00000013027	ENSPPYT00000007624	ENSOPRT00000016598	ENSOCUT00000006459	ENSRNOT00000012447	ENSSTOT00000005369	ENSTBET00000006358	ENSTSYT00000008158
ENST00000442287	ENSOGAT00000008153	ENSPTRT00000013707	ENSBTAT00000011993	ENSNLET00000014904	ENSGGOT00000011779	ENSCPOT00000007338	ENSDORT00000014142	ENSMMUT00000015604	ENSCJAT00000024820	ENSMUST00000073889	ENSMICT00000001955	ENSPPYT00000007985	ENSOPRT00000013023	ENSOCUT00000011507	ENSRNOT00000044567	ENSSTOT00000006879	ENSTBET00000013190	ENSTSYT00000012930
ENST00000296292	ENSOGAT00000003298	ENSPTRT00000028128	ENSBTAT00000061626	ENSNLET00000009209	ENSGGOT00000003220	ENSCPOT00000010891	ENSDORT00000008360	ENSMMUT00000022755	ENSCJAT00000014084	ENSMUST00000064230	ENSMICT00000010891	ENSPPYT00000016043	ENSOPRT00000011018	ENSOCUT00000004546	ENSRNOT00000035542	ENSSTOT00000004564	ENSTBET00000011394	ENSTSYT00000010702
ENST00000239891	ENSOGAT00000002744	ENSPTRT00000010650	ENSBTAT00000015727	ENSNLET00000002189	ENSGGOT00000003112	ENSCPOT00000004496	ENSDORT00000009002	ENSMMUT00000005075	ENSCJAT00000035397	ENSMUST00000044567	ENSMICT00000003347	ENSPPYT00000006259	ENSOPRT00000004941	ENSOCUT00000007457	ENSRNOT00000058001	ENSSTOT00000002023	ENSTBET00000011707	ENSTSYT00000013225
ENST00000264257	ENSOGAT00000008054	ENSPTRT00000022831	ENSBTAT00000007489	ENSNLET00000018693	ENSGGOT00000003368	ENSCPOT00000003247	ENSDORT00000008125	ENSMMUT00000023222	ENSCJAT00000016228	ENSMUST00000095020	ENSMICT00000015302	ENSPPYT00000014055	ENSOPRT00000005964	ENSOCUT00000009728	ENSRNOT00000019880	ENSSTOT00000004142	ENSTBET00000011807	ENSTSYT00000005446
ENST00000486442	ENSOGAT00000004576	ENSPTRT00000021792	ENSBTAT00000043605	ENSNLET00000000870	ENSGGOT00000013936	ENSCPOT00000014316	ENSDORT00000012917	ENSMMUT00000022538	ENSCJAT00000014879	ENSMUST00000020958	ENSMICT00000003483	ENSPPYT00000014648	ENSOPRT00000016834	ENSOCUT00000017373	ENSRNOT00000007160	ENSSTOT00000008416	ENSTBET00000006199	ENSTSYT00000008635
ENST00000282041	ENSOGAT00000030062	ENSPTRT00000046427	ENSBTAT00000016012	ENSNLET00000013952	ENSGGOT00000007878	ENSCPOT00000012230	ENSDORT00000013624	ENSMMUT00000005037	ENSCJAT00000016726	ENSMUST00000044622	ENSMICT00000017498	ENSPPYT00000010660	ENSOPRT00000004791	ENSOCUT00000003952	ENSRNOT00000022767	ENSSTOT00000003637	ENSTBET00000002566	ENSTSYT00000001562
ENST00000418476	ENSOGAT00000005738	ENSPTRT00000013766	ENSBTAT00000024799	ENSNLET00000015618	ENSGGOT00000008245	ENSCPOT00000001789	ENSDORT00000014066	ENSMMUT00000007201	ENSCJAT00000018732	ENSMUST00000107368	ENSMICT00000008331	ENSPPYT00000008016	ENSOPRT00000011841	ENSOCUT00000013784	ENSRNOT00000017407	ENSSTOT00000014857	ENSTBET00000000338	ENSTSYT00000005831
ENST00000227638	ENSOGAT00000015296	ENSPTRT00000007801	ENSBTAT00000015932	ENSNLET00000009069	ENSGGOT00000005623	ENSCPOT00000002861	ENSDORT00000004772	ENSMMUT00000006383	ENSCJAT00000007582	ENSMUST00000164273	ENSMICT00000006408	ENSPPYT00000004497	ENSOPRT00000014175	ENSOCUT00000009469	ENSRNOT00000013577	ENSSTOT00000006840	ENSTBET00000001711	ENSTSYT00000013664
ENST00000552432	ENSOGAT00000008399	ENSPTRT00000009488	ENSBTAT00000028361	ENSNLET00000021849	ENSGGOT00000032915	ENSCPOT00000010313	ENSDORT00000012107	ENSMMUT00000046246	ENSCJAT00000058339	ENSMUST00000063318	ENSMICT00000014663	ENSPPYT00000005581	ENSOPRT00000008328	ENSOCUT00000003051	ENSRNOT00000010379	ENSSTOT00000008040	ENSTBET00000014444	ENSTSYT00000005961
ENST00000339950	ENSOGAT00000009361	ENSPTRT00000001537	ENSBTAT00000027254	ENSNLET00000019055	ENSGGOT00000025009	ENSCPOT00000010510	ENSDORT00000008268	ENSMMUT00000003313	ENSCJAT00000061509	ENSMUST00000091358	ENSMICT00000001501	ENSPPYT00000001545	ENSOPRT00000015697	ENSOCUT00000012941	ENSRNOT00000010933	ENSSTOT00000004962	ENSTBET00000009952	ENSTSYT00000009692
ENST00000263635	ENSOGAT00000005662	ENSPTRT00000023282	ENSBTAT00000005473	ENSNLET00000001271	ENSGGOT00000009514	ENSCPOT00000008757	ENSDORT00000010963	ENSMMUT00000008647	ENSCJAT00000029602	ENSMUST00000139863	ENSMICT00000010486	ENSPPYT00000014947	ENSOPRT00000002523	ENSOCUT00000015117	ENSRNOT00000038983	ENSSTOT00000009750	ENSTBET00000001294	ENSTSYT00000001727
ENST00000328273	ENSOGAT00000014387	ENSPTRT00000014784	ENSBTAT00000004433	ENSNLET00000020399	ENSGGOT00000027783	ENSCPOT00000022675	ENSDORT00000009885	ENSMMUT00000029361	ENSCJAT00000005466	ENSMUST00000033086	ENSMICT00000001545	ENSPPYT00000008570	ENSOPRT00000012679	ENSOCUT00000013944	ENSRNOT00000025419	ENSSTOT00000004807	ENSTBET00000013173	ENSTSYT00000007450
ENST00000290363	ENSOGAT00000004849	ENSPTRT00000002319	ENSBTAT00000028639	ENSNLET00000012560	ENSGGOT00000017021	ENSCPOT00000009395	ENSDORT00000002896	ENSMMUT00000030109	ENSCJAT00000019214	ENSMUST00000159739	ENSMICT00000015357	ENSPPYT00000001091	ENSOPRT00000010118	ENSOCUT00000009044	ENSRNOT00000056322	ENSSTOT00000001879	ENSTBET00000000874	ENSTSYT00000006878
ENST00000537664	ENSOGAT00000003359	ENSPTRT00000034774	ENSBTAT00000005065	ENSNLET00000000353	ENSGGOT00000005237	ENSCPOT00000015666	ENSDORT00000007630	ENSMMUT00000029746	ENSCJAT00000040567	ENSMUST00000055352	ENSMICT00000017133	ENSPPYT00000020013	ENSOPRT00000010721	ENSOCUT00000012579	ENSRNOT00000002036	ENSSTOT00000013528	ENSTBET00000014383	ENSTSYT00000013275
ENST00000370113	ENSOGAT00000014971	ENSPTRT00000001893	ENSBTAT00000025321	ENSNLET00000015198	ENSGGOT00000004438	ENSCPOT00000026456	ENSDORT00000013732	ENSMMUT00000005274	ENSCJAT00000006717	ENSMUST00000106502	ENSMICT00000005230	ENSPPYT00000001333	ENSOPRT00000004309	ENSOCUT00000002007	ENSRNOT00000019190	ENSSTOT00000005347	ENSTBET00000013894	ENSTSYT00000004348
ENST00000342560	ENSOGAT00000007716	ENSPTRT00000059046	ENSBTAT00000003358	ENSNLET00000008394	ENSGGOT00000003348	ENSCPOT00000021607	ENSDORT00000008322	ENSMMUT00000004020	ENSCJAT00000034670	ENSMUST00000109420	ENSMICT00000008226	ENSPPYT00000012797	ENSOPRT00000014238	ENSOCUT00000004100	ENSRNOT00000011748	ENSSTOT00000015652	ENSTBET00000004888	ENSTSYT00000005952
ENST00000358691	ENSOGAT00000002487	ENSPTRT00000017533	ENSBTAT00000006233	ENSNLET00000000309	ENSGGOT00000012376	ENSCPOT00000015057	ENSDORT00000014257	ENSMMUT00000041135	ENSCJAT00000057035	ENSMUST00000106746	ENSMICT00000013354	ENSPPYT00000010002	ENSOPRT00000015771	ENSOCUT00000008866	ENSRNOT00000030300	ENSSTOT00000007856	ENSTBET00000003170	ENSTSYT00000009452
ENST00000294119	ENSOGAT00000011415	ENSPTRT00000007043	ENSBTAT00000013845	ENSNLET00000005148	ENSGGOT00000005073	ENSCPOT00000004801	ENSDORT00000009406	ENSMMUT00000008090	ENSCJAT00000036841	ENSMUST00000096255	ENSMICT00000009922	ENSPPYT00000003772	ENSOPRT00000014691	ENSOCUT00000017279	ENSRNOT00000026651	ENSSTOT00000004309	ENSTBET00000007974	ENSTSYT00000004439
ENST00000287936	ENSOGAT00000003781	ENSPTRT00000031507	ENSBTAT00000010315	ENSNLET00000006528	ENSGGOT00000010967	ENSCPOT00000004321	ENSDORT00000005455	ENSMMUT00000008582	ENSCJAT00000033064	ENSMUST00000022176	ENSMICT00000014541	ENSPPYT00000018099	ENSOPRT00000003628	ENSOCUT00000017428	ENSRNOT00000022055	ENSSTOT00000009621	ENSTBET00000004431	ENSTSYT00000010407
ENST00000290130	ENSOGAT00000017142	ENSPTRT00000025830	ENSBTAT00000022106	ENSNLET00000004645	ENSGGOT00000004853	ENSCPOT00000021869	ENSDORT00000006732	ENSMMUT00000031719	ENSCJAT00000017631	ENSMUST00000099554	ENSMICT00000016395	ENSPPYT00000013181	ENSOPRT00000013427	ENSOCUT00000009375	ENSRNOT00000037444	ENSSTOT00000004501	ENSTBET00000009739	ENSTSYT00000001351
ENST00000329956	ENSOGAT00000000018	ENSPTRT00000015170	ENSBTAT00000002402	ENSNLET00000005907	ENSGGOT00000008510	ENSCPOT00000010978	ENSDORT00000005868	ENSMMUT00000013722	ENSCJAT00000031539	ENSMUST00000109355	ENSMICT00000012054	ENSPPYT00000008764	ENSOPRT00000013332	ENSOCUT00000010081	ENSRNOT00000022622	ENSSTOT00000016446	ENSTBET00000002829	ENSTSYT00000005473
ENST00000227322	ENSOGAT00000009982	ENSPTRT00000008019	ENSBTAT00000003433	ENSNLET00000009363	ENSGGOT00000023658	ENSCPOT00000014587	ENSDORT00000012404	ENSMMUT00000028537	ENSCJAT00000024412	ENSMUST00000156440	ENSMICT00000002323	ENSPPYT00000029393	ENSOPRT00000016250	ENSOCUT00000004619	ENSRNOT00000025149	ENSSTOT00000002310	ENSTBET00000004789	ENSTSYT00000007314
ENST00000454009	ENSOGAT00000004917	ENSPTRT00000045905	ENSBTAT00000021039	ENSNLET00000019808	ENSGGOT00000008828	ENSCPOT00000001119	ENSDORT00000011180	ENSMMUT00000020262	ENSCJAT00000039795	ENSMUST00000048129	ENSMICT00000001070	ENSPPYT00000021470	ENSOPRT00000013181	ENSOCUT00000006269	ENSRNOT00000013284	ENSSTOT00000016523	ENSTBET00000012084	ENSTSYT00000009638
ENST00000467467	ENSOGAT00000034137	ENSPTRT00000047540	ENSBTAT00000010277	ENSNLET00000003931	ENSGGOT00000000215	ENSCPOT00000009941	ENSDORT00000009862	ENSMMUT00000026682	ENSCJAT00000002481	ENSMUST00000029380	ENSMICT00000004453	ENSPPYT00000016508	ENSOPRT00000013834	ENSOCUT00000013749	ENSRNOT00000022507	ENSSTOT00000010882	ENSTBET00000003369	ENSTSYT00000007168
ENST00000342081	ENSOGAT00000010690	ENSPTRT00000030084	ENSBTAT00000015836	ENSNLET00000010112	ENSGGOT00000003176	ENSCPOT00000008913	ENSDORT00000014674	ENSMMUT00000005430	ENSCJAT00000027105	ENSMUST00000031317	ENSMICT00000005087	ENSPPYT00000017230	ENSOPRT00000002223	ENSOCUT00000011825	ENSRNOT00000003836	ENSSTOT00000000133	ENSTBET00000015355	ENSTSYT00000010681
ENST00000367749	ENSOGAT00000016487	ENSPTRT00000003034	ENSBTAT00000022185	ENSNLET00000019343	ENSGGOT00000012625	ENSCPOT00000014140	ENSDORT00000009308	ENSMMUT00000019969	ENSCJAT00000018970	ENSMUST00000111525	ENSMICT00000017123	ENSPPYT00000000620	ENSOPRT00000011069	ENSOCUT00000002796	ENSRNOT00000004599	ENSSTOT00000003097	ENSTBET00000002791	ENSTSYT00000010437
ENST00000378789	ENSOGAT00000009152	ENSPTRT00000024607	ENSBTAT00000061291	ENSNLET00000017998	ENSGGOT00000004385	ENSCPOT00000000448	ENSDORT00000011791	ENSMMUT00000012855	ENSCJAT00000041917	ENSMUST00000028704	ENSMICT00000003030	ENSPPYT00000012431	ENSOPRT00000003329	ENSOCUT00000011536	ENSRNOT00000006330	ENSSTOT00000003917	ENSTBET00000009357	ENSTSYT00000011218
ENST00000222462	ENSOGAT00000004219	ENSPTRT00000036352	ENSBTAT00000003825	ENSNLET00000015183	ENSGGOT00000016561	ENSCPOT00000005696	ENSDORT00000014500	ENSMMUT00000003477	ENSCJAT00000061637	ENSMUST00000031681	ENSMICT00000013954	ENSPPYT00000020920	ENSOPRT00000016941	ENSOCUT00000014737	ENSRNOT00000007822	ENSSTOT00000006576	ENSTBET00000006818	ENSTSYT00000010205
ENST00000367654	ENSOGAT00000010667	ENSPTRT00000043399	ENSBTAT00000061165	ENSNLET00000019738	ENSGGOT00000013437	ENSCPOT00000012034	ENSDORT00000015501	ENSMMUT00000032661	ENSCJAT00000016748	ENSMUST00000046110	ENSMICT00000005992	ENSPPYT00000000573	ENSOPRT00000005416	ENSOCUT00000015553	ENSRNOT00000007660	ENSSTOT00000011888	ENSTBET00000010389	ENSTSYT00000008527
ENST00000361927	ENSOGAT00000003128	ENSPTRT00000044909	ENSBTAT00000050322	ENSNLET00000023004	ENSGGOT00000013403	ENSCPOT00000003574	ENSDORT00000007340	ENSMMUT00000023035	ENSCJAT00000010324	ENSMUST00000044311	ENSMICT00000007205	ENSPPYT00000000498	ENSOPRT00000015468	ENSOCUT00000016332	ENSRNOT00000030109	ENSSTOT00000013220	ENSTBET00000008207	ENSTSYT00000008986
ENST00000339618	ENSOGAT00000017084	ENSPTRT00000016358	ENSBTAT00000056266	ENSNLET00000013698	ENSGGOT00000007699	ENSCPOT00000013925	ENSDORT00000001470	ENSMMUT00000019833	ENSCJAT00000022798	ENSMUST00000074127	ENSMICT00000001236	ENSPPYT00000010315	ENSOPRT00000017158	ENSOCUT00000011904	ENSRNOT00000040910	ENSSTOT00000007914	ENSTBET00000015632	ENSTSYT00000002301
ENST00000321037	ENSOGAT00000014381	ENSPTRT00000033713	ENSBTAT00000004547	ENSNLET00000004557	ENSGGOT00000007498	ENSCPOT00000015311	ENSDORT00000015218	ENSMMUT00000040499	ENSCJAT00000023508	ENSMUST00000143137	ENSMICT00000017750	ENSPPYT00000019383	ENSOPRT00000006731	ENSOCUT00000012926	ENSRNOT00000045937	ENSSTOT00000011889	ENSTBET00000007600	ENSTSYT00000010276
ENST00000380130	ENSOGAT00000015759	ENSPTRT00000010611	ENSBTAT00000001315	ENSNLET00000001358	ENSGGOT00000015904	ENSCPOT00000003409	ENSDORT00000007051	ENSMMUT00000010050	ENSCJAT00000036137	ENSMUST00000016279	ENSMICT00000012013	ENSPPYT00000006233	ENSOPRT00000015425	ENSOCUT00000016895	ENSRNOT00000061446	ENSSTOT00000005587	ENSTBET00000016030	ENSTSYT00000000480
ENST00000545128	ENSOGAT00000013118	ENSPTRT00000038898	ENSBTAT00000036658	ENSNLET00000002556	ENSGGOT00000025459	ENSCPOT00000002714	ENSDORT00000004033	ENSMMUT00000030986	ENSCJAT00000024978	ENSMUST00000025618	ENSMICT00000014068	ENSPPYT00000022506	ENSOPRT00000015838	ENSOCUT00000001620	ENSRNOT00000054793	ENSSTOT00000012058	ENSTBET00000000188	ENSTSYT00000013296
ENST00000539410	ENSOGAT00000003683	ENSPTRT00000009830	ENSBTAT00000018363	ENSNLET00000007795	ENSGGOT00000026190	ENSCPOT00000004168	ENSDORT00000001224	ENSMMUT00000028631	ENSCJAT00000003944	ENSMUST00000099375	ENSMICT00000013534	ENSPPYT00000005770	ENSOPRT00000011014	ENSOCUT00000007183	ENSRNOT00000033123	ENSSTOT00000011785	ENSTBET00000016725	ENSTSYT00000011057
ENST00000517399	ENSOGAT00000000275	ENSPTRT00000003596	ENSBTAT00000061152	ENSNLET00000018556	ENSGGOT00000027246	ENSCPOT00000003915	ENSDORT00000000751	ENSMMUT00000016431	ENSCJAT00000030496	ENSMUST00000047409	ENSMICT00000014709	ENSPPYT00000000252	ENSOPRT00000016848	ENSOCUT00000008575	ENSRNOT00000005095	ENSSTOT00000001090	ENSTBET00000010102	ENSTSYT00000014365
ENST00000286827	ENSOGAT00000004010	ENSPTRT00000025824	ENSBTAT00000023716	ENSNLET00000004500	ENSGGOT00000014809	ENSCPOT00000013742	ENSDORT00000003677	ENSMMUT00000027491	ENSCJAT00000017365	ENSMUST00000163370	ENSMICT00000016841	ENSPPYT00000013175	ENSOPRT00000016671	ENSOCUT00000016533	ENSRNOT00000046486	ENSSTOT00000005891	ENSTBET00000014799	ENSTSYT00000002685
ENST00000298746	ENSOGAT00000015901	ENSPTRT00000005657	ENSBTAT00000017209	ENSNLET00000020829	ENSGGOT00000004611	ENSCPOT00000005678	ENSDORT00000010431	ENSMMUT00000002689	ENSCJAT00000037970	ENSMUST00000026073	ENSMICT00000016318	ENSPPYT00000003232	ENSOPRT00000009366	ENSOCUT00000009456	ENSRNOT00000023309	ENSSTOT00000003669	ENSTBET00000002877	ENSTSYT00000000446
ENST00000375533	ENSOGAT00000002862	ENSPTRT00000004388	ENSBTAT00000003960	ENSNLET00000021722	ENSGGOT00000004622	ENSCPOT00000005034	ENSDORT00000011372	ENSMMUT00000000823	ENSCJAT00000025601	ENSMUST00000025075	ENSMICT00000003981	ENSPPYT00000002612	ENSOPRT00000001679	ENSOCUT00000007402	ENSRNOT00000021801	ENSSTOT00000014963	ENSTBET00000017287	ENSTSYT00000009563
ENST00000334379	ENSOGAT00000008773	ENSPTRT00000042447	ENSBTAT00000002544	ENSNLET00000017997	ENSGGOT00000017075	ENSCPOT00000009207	ENSDORT00000011192	ENSMMUT00000006105	ENSCJAT00000053990	ENSMUST00000019927	ENSMICT00000014102	ENSPPYT00000019775	ENSOPRT00000001774	ENSOCUT00000011783	ENSRNOT00000019436	ENSSTOT00000006690	ENSTBET00000001868	ENSTSYT00000004447
ENST00000285393	ENSOGAT00000009234	ENSPTRT00000037740	ENSBTAT00000028091	ENSNLET00000001858	ENSGGOT00000013802	ENSCPOT00000009727	ENSDORT00000016038	ENSMMUT00000002291	ENSCJAT00000000620	ENSMUST00000029900	ENSMICT00000006222	ENSPPYT00000021839	ENSOPRT00000004565	ENSOCUT00000010246	ENSRNOT00000009334	ENSSTOT00000014844	ENSTBET00000001660	ENSTSYT00000014040
ENST00000372172	ENSOGAT00000009675	ENSPTRT00000001273	ENSBTAT00000025307	ENSNLET00000018190	ENSGGOT00000001538	ENSCPOT00000013114	ENSDORT00000010561	ENSMMUT00000018229	ENSCJAT00000008594	ENSMUST00000050067	ENSMICT00000010333	ENSPPYT00000001701	ENSOPRT00000004131	ENSOCUT00000003446	ENSRNOT00000024737	ENSSTOT00000006487	ENSTBET00000015601	ENSTSYT00000008250
ENST00000290953	ENSOGAT00000027765	ENSPTRT00000015179	ENSBTAT00000019347	ENSNLET00000006022	ENSGGOT00000013027	ENSCPOT00000011010	ENSDORT00000013973	ENSMMUT00000011243	ENSCJAT00000031484	ENSMUST00000005849	ENSMICT00000012091	ENSPPYT00000008770	ENSOPRT00000013409	ENSOCUT00000011854	ENSRNOT00000059628	ENSSTOT00000016451	ENSTBET00000007171	ENSTSYT00000002534
ENST00000262866	ENSOGAT00000000893	ENSPTRT00000025033	ENSBTAT00000026137	ENSNLET00000007800	ENSGGOT00000012932	ENSCPOT00000011543	ENSDORT00000008455	ENSMMUT00000011461	ENSCJAT00000002486	ENSMUST00000029164	ENSMICT00000000369	ENSPPYT00000012736	ENSOPRT00000012257	ENSOCUT00000004574	ENSRNOT00000027581	ENSSTOT00000013925	ENSTBET00000006907	ENSTSYT00000000385
ENST00000440460	ENSOGAT00000008558	ENSPTRT00000034508	ENSBTAT00000040095	ENSNLET00000009306	ENSGGOT00000002528	ENSCPOT00000008645	ENSDORT00000001925	ENSMMUT00000024711	ENSCJAT00000021233	ENSMUST00000117646	ENSMICT00000015847	ENSPPYT00000004609	ENSOPRT00000012623	ENSOCUT00000012921	ENSRNOT00000068654	ENSSTOT00000011118	ENSTBET00000010955	ENSTSYT00000013179
ENST00000338104	ENSOGAT00000013290	ENSPTRT00000012147	ENSBTAT00000042964	ENSNLET00000021663	ENSGGOT00000041059	ENSCPOT00000000702	ENSDORT00000001384	ENSMMUT00000006530	ENSCJAT00000034277	ENSMUST00000079146	ENSMICT00000016703	ENSPPYT00000007156	ENSOPRT00000001252	ENSOCUT00000015556	ENSRNOT00000006199	ENSSTOT00000013257	ENSTBET00000011603	ENSTSYT00000010788
ENST00000371790	ENSOGAT00000009665	ENSPTRT00000005173	ENSBTAT00000006118	ENSNLET00000015691	ENSGGOT00000004008	ENSCPOT00000009423	ENSDORT00000003059	ENSMMUT00000024658	ENSCJAT00000021826	ENSMUST00000009522	ENSMICT00000016822	ENSPPYT00000002960	ENSOPRT00000015795	ENSOCUT00000014498	ENSRNOT00000030024	ENSSTOT00000009605	ENSTBET00000004478	ENSTSYT00000000854
ENST00000412891	ENSOGAT00000014632	ENSPTRT00000010726	ENSBTAT00000016503	ENSNLET00000002419	ENSGGOT00000006223	ENSCPOT00000024592	ENSDORT00000001329	ENSMMUT00000003304	ENSCJAT00000034239	ENSMUST00000022589	ENSMICT00000007707	ENSPPYT00000006302	ENSOPRT00000016210	ENSOCUT00000006640	ENSRNOT00000011986	ENSSTOT00000015957	ENSTBET00000003552	ENSTSYT00000009821
ENST00000559471	ENSOGAT00000013285	ENSPTRT00000058496	ENSBTAT00000065830	ENSNLET00000007790	ENSGGOT00000007331	ENSCPOT00000003486	ENSDORT00000008662	ENSMMUT00000013742	ENSCJAT00000003143	ENSMUST00000053699	ENSMICT00000013008	ENSPPYT00000007621	ENSOPRT00000010906	ENSOCUT00000006446	ENSRNOT00000011551	ENSSTOT00000005326	ENSTBET00000013940	ENSTSYT00000004352
ENST00000252011	ENSOGAT00000000955	ENSPTRT00000059069	ENSBTAT00000005281	ENSNLET00000008232	ENSGGOT00000014023	ENSCPOT00000012529	ENSDORT00000005573	ENSMMUT00000004782	ENSCJAT00000022450	ENSMUST00000109478	ENSMICT00000013298	ENSPPYT00000012773	ENSOPRT00000013615	ENSOCUT00000009421	ENSRNOT00000068625	ENSSTOT00000002011	ENSTBET00000001850	ENSTSYT00000007052
ENST00000503945	ENSOGAT00000033433	ENSPTRT00000029749	ENSBTAT00000025895	ENSNLET00000021171	ENSGGOT00000001121	ENSCPOT00000012737	ENSDORT00000008041	ENSMMUT00000008052	ENSCJAT00000024643	ENSMUST00000156481	ENSMICT00000006790	ENSPPYT00000017005	ENSOPRT00000003918	ENSOCUT00000001574	ENSRNOT00000004912	ENSSTOT00000000926	ENSTBET00000012288	ENSTSYT00000006837
ENST00000312465	ENSOGAT00000011246	ENSPTRT00000030168	ENSBTAT00000023064	ENSNLET00000010866	ENSGGOT00000002957	ENSCPOT00000001412	ENSDORT00000013481	ENSMMUT00000012686	ENSCJAT00000029351	ENSMUST00000031280	ENSMICT00000002164	ENSPPYT00000017291	ENSOPRT00000016396	ENSOCUT00000012415	ENSRNOT00000035446	ENSSTOT00000007666	ENSTBET00000007198	ENSTSYT00000014496
ENST00000299505	ENSOGAT00000008835	ENSPTRT00000020010	ENSBTAT00000017535	ENSNLET00000014996	ENSGGOT00000010374	ENSCPOT00000028356	ENSDORT00000012861	ENSMMUT00000008228	ENSCJAT00000020374	ENSMUST00000085592	ENSMICT00000011608	ENSPPYT00000011441	ENSOPRT00000003699	ENSOCUT00000002238	ENSRNOT00000028688	ENSSTOT00000002568	ENSTBET00000003921	ENSTSYT00000011891
ENST00000375490	ENSOGAT00000016862	ENSPTRT00000008011	ENSBTAT00000010804	ENSNLET00000009355	ENSGGOT00000009976	ENSCPOT00000005377	ENSDORT00000011258	ENSMMUT00000017188	ENSCJAT00000023199	ENSMUST00000048956	ENSMICT00000010083	ENSPPYT00000004637	ENSOPRT00000011052	ENSOCUT00000003341	ENSRNOT00000007736	ENSSTOT00000012457	ENSTBET00000008636	ENSTSYT00000013730
ENST00000392687	ENSOGAT00000007897	ENSPTRT00000065769	ENSBTAT00000061640	ENSNLET00000005848	ENSGGOT00000016858	ENSCPOT00000021482	ENSDORT00000004631	ENSMMUT00000020123	ENSCJAT00000012003	ENSMUST00000028426	ENSMICT00000017318	ENSPPYT00000014989	ENSOPRT00000013218	ENSOCUT00000005720	ENSRNOT00000058763	ENSSTOT00000007390	ENSTBET00000010135	ENSTSYT00000003208
ENST00000392238	ENSOGAT00000001497	ENSPTRT00000023775	ENSBTAT00000024963	ENSNLET00000008953	ENSGGOT00000014864	ENSCPOT00000007137	ENSDORT00000015289	ENSMMUT00000029754	ENSCJAT00000009911	ENSMUST00000036540	ENSMICT00000002696	ENSPPYT00000015216	ENSOPRT00000008343	ENSOCUT00000006349	ENSRNOT00000031261	ENSSTOT00000013454	ENSTBET00000002518	ENSTSYT00000013148
ENST00000381870	ENSOGAT00000004454	ENSPTRT00000015814	ENSBTAT00000056260	ENSNLET00000022004	ENSGGOT00000009933	ENSCPOT00000020055	ENSDORT00000013800	ENSMMUT00000042413	ENSCJAT00000013957	ENSMUST00000108476	ENSMICT00000015735	ENSPPYT00000009164	ENSOPRT00000016882	ENSOCUT00000008001	ENSRNOT00000030343	ENSSTOT00000002056	ENSTBET00000005669	ENSTSYT00000001096
ENST00000371088	ENSOGAT00000005257	ENSPTRT00000059135	ENSBTAT00000003365	ENSNLET00000019238	ENSGGOT00000012520	ENSCPOT00000021370	ENSDORT00000013731	ENSMMUT00000028185	ENSCJAT00000032246	ENSMUST00000170632	ENSMICT00000013621	ENSPPYT00000001534	ENSOPRT00000013098	ENSOCUT00000000286	ENSRNOT00000013089	ENSSTOT00000013536	ENSTBET00000000201	ENSTSYT00000012074
ENST00000371120	ENSOGAT00000004022	ENSPTRT00000001543	ENSBTAT00000013356	ENSNLET00000019210	ENSGGOT00000007287	ENSCPOT00000001628	ENSDORT00000015253	ENSMMUT00000000560	ENSCJAT00000032095	ENSMUST00000030279	ENSMICT00000001539	ENSPPYT00000001539	ENSOPRT00000010914	ENSOCUT00000007697	ENSRNOT00000011631	ENSSTOT00000012443	ENSTBET00000010881	ENSTSYT00000009579
ENST00000345317	ENSOGAT00000012370	ENSPTRT00000035142	ENSBTAT00000046037	ENSNLET00000019110	ENSGGOT00000002160	ENSCPOT00000000261	ENSDORT00000009682	ENSMMUT00000026074	ENSCJAT00000029470	ENSMUST00000078214	ENSMICT00000000466	ENSPPYT00000020637	ENSOPRT00000011763	ENSOCUT00000011330	ENSRNOT00000016608	ENSSTOT00000015528	ENSTBET00000014197	ENSTSYT00000002307
ENST00000333677	ENSOGAT00000003257	ENSPTRT00000032190	ENSBTAT00000020493	ENSNLET00000013026	ENSGGOT00000001883	ENSCPOT00000002943	ENSDORT00000016036	ENSMMUT00000024897	ENSCJAT00000041514	ENSMUST00000171629	ENSMICT00000002695	ENSPPYT00000018547	ENSOPRT00000016891	ENSOCUT00000006829	ENSRNOT00000032189	ENSSTOT00000008680	ENSTBET00000001422	ENSTSYT00000014229
ENST00000262173	ENSOGAT00000001327	ENSPTRT00000056739	ENSBTAT00000012047	ENSNLET00000011828	ENSGGOT00000005550	ENSCPOT00000011494	ENSDORT00000016722	ENSMMUT00000004935	ENSCJAT00000063489	ENSMUST00000009679	ENSMICT00000001419	ENSPPYT00000010475	ENSOPRT00000015457	ENSOCUT00000005612	ENSRNOT00000022410	ENSSTOT00000005346	ENSTBET00000005606	ENSTSYT00000008424
ENST00000392552	ENSOGAT00000016578	ENSPTRT00000023448	ENSBTAT00000028831	ENSNLET00000007497	ENSGGOT00000029275	ENSCPOT00000010380	ENSDORT00000013684	ENSMMUT00000029545	ENSCJAT00000015573	ENSMUST00000076463	ENSMICT00000000371	ENSPPYT00000015044	ENSOPRT00000010530	ENSOCUT00000003686	ENSRNOT00000025158	ENSSTOT00000001240	ENSTBET00000007953	ENSTSYT00000012367
ENST00000506176	ENSOGAT00000006391	ENSPTRT00000031845	ENSBTAT00000044112	ENSNLET00000015805	ENSGGOT00000027869	ENSCPOT00000000265	ENSDORT00000010692	ENSMMUT00000023941	ENSCJAT00000037846	ENSMUST00000025500	ENSMICT00000004555	ENSPPYT00000018312	ENSOPRT00000010948	ENSOCUT00000017140	ENSRNOT00000045244	ENSSTOT00000007922	ENSTBET00000009769	ENSTSYT00000007370
ENST00000504514	ENSOGAT00000007015	ENSPTRT00000031516	ENSBTAT00000061288	ENSNLET00000006695	ENSGGOT00000015154	ENSCPOT00000005488	ENSDORT00000007845	ENSMMUT00000041433	ENSCJAT00000054418	ENSMUST00000055607	ENSMICT00000006484	ENSPPYT00000018104	ENSOPRT00000003059	ENSOCUT00000002936	ENSRNOT00000034041	ENSSTOT00000009827	ENSTBET00000012307	ENSTSYT00000000321
ENST00000296280	ENSOGAT00000016045	ENSPTRT00000029310	ENSBTAT00000016545	ENSNLET00000008537	ENSGGOT00000001227	ENSCPOT00000015878	ENSDORT00000015182	ENSMMUT00000018240	ENSCJAT00000022473	ENSMUST00000089883	ENSMICT00000007416	ENSPPYT00000016741	ENSOPRT00000009034	ENSOCUT00000000050	ENSRNOT00000002512	ENSSTOT00000010267	ENSTBET00000004060	ENSTSYT00000012383
ENST00000367313	ENSOGAT00000027665	ENSPTRT00000003331	ENSBTAT00000034341	ENSNLET00000023077	ENSGGOT00000012736	ENSCPOT00000005356	ENSDORT00000006349	ENSMMUT00000018967	ENSCJAT00000010598	ENSMUST00000038760	ENSMICT00000003006	ENSPPYT00000000427	ENSOPRT00000011836	ENSOCUT00000007731	ENSRNOT00000012801	ENSSTOT00000012842	ENSTBET00000009346	ENSTSYT00000009032
ENST00000550804	ENSOGAT00000003615	ENSPTRT00000044463	ENSBTAT00000019992	ENSNLET00000022549	ENSGGOT00000011586	ENSCPOT00000023458	ENSDORT00000002958	ENSMMUT00000013195	ENSCJAT00000036874	ENSMUST00000023818	ENSMICT00000011695	ENSPPYT00000005438	ENSOPRT00000002993	ENSOCUT00000008945	ENSRNOT00000021523	ENSSTOT00000007149	ENSTBET00000005782	ENSTSYT00000001185
ENST00000266719	ENSOGAT00000008615	ENSPTRT00000009721	ENSBTAT00000019066	ENSNLET00000007101	ENSGGOT00000009691	ENSCPOT00000000680	ENSDORT00000005983	ENSMMUT00000017542	ENSCJAT00000038358	ENSMUST00000170547	ENSMICT00000000375	ENSPPYT00000005714	ENSOPRT00000011581	ENSOCUT00000013390	ENSRNOT00000006234	ENSSTOT00000011956	ENSTBET00000002281	ENSTSYT00000010175
ENST00000453321	ENSOGAT00000002984	ENSPTRT00000037787	ENSBTAT00000061587	ENSNLET00000003764	ENSGGOT00000030088	ENSCPOT00000007891	ENSDORT00000004151	ENSMMUT00000038961	ENSCJAT00000055151	ENSMUST00000108293	ENSMICT00000015182	ENSPPYT00000021867	ENSOPRT00000000304	ENSOCUT00000010397	ENSRNOT00000021839	ENSSTOT00000010306	ENSTBET00000003540	ENSTSYT00000008969
ENST00000280701	ENSOGAT00000002398	ENSPTRT00000027499	ENSBTAT00000009250	ENSNLET00000004890	ENSGGOT00000033039	ENSCPOT00000026012	ENSDORT00000010197	ENSMMUT00000020756	ENSCJAT00000007858	ENSMUST00000112624	ENSMICT00000013516	ENSPPYT00000016351	ENSOPRT00000013042	ENSOCUT00000015376	ENSRNOT00000008035	ENSSTOT00000004957	ENSTBET00000012059	ENSTSYT00000014133
ENST00000536493	ENSOGAT00000000484	ENSPTRT00000013133	ENSBTAT00000017894	ENSNLET00000014443	ENSGGOT00000012194	ENSCPOT00000013362	ENSDORT00000007592	ENSMMUT00000015705	ENSCJAT00000015470	ENSMUST00000113570	ENSMICT00000006129	ENSPPYT00000007687	ENSOPRT00000015623	ENSOCUT00000009796	ENSRNOT00000021442	ENSSTOT00000014132	ENSTBET00000002544	ENSTSYT00000001457
ENST00000491677	ENSOGAT00000008351	ENSPTRT00000047891	ENSBTAT00000003801	ENSNLET00000021486	ENSGGOT00000011062	ENSCPOT00000006626	ENSDORT00000012352	ENSMMUT00000012977	ENSCJAT00000002229	ENSMUST00000119866	ENSMICT00000017813	ENSPPYT00000002781	ENSOPRT00000002368	ENSOCUT00000009069	ENSRNOT00000017663	ENSSTOT00000005502	ENSTBET00000008767	ENSTSYT00000012936
ENST00000217455	ENSOGAT00000004995	ENSPTRT00000025258	ENSBTAT00000027152	ENSNLET00000008652	ENSGGOT00000003075	ENSCPOT00000012932	ENSDORT00000011784	ENSMMUT00000045001	ENSCJAT00000033729	ENSMUST00000103094	ENSMICT00000011231	ENSPPYT00000012857	ENSOPRT00000014313	ENSOCUT00000012183	ENSRNOT00000020740	ENSSTOT00000006529	ENSTBET00000003304	ENSTSYT00000012051
ENST00000263035	ENSOGAT00000005806	ENSPTRT00000004199	ENSBTAT00000000364	ENSNLET00000015730	ENSGGOT00000010897	ENSCPOT00000008323	ENSDORT00000002480	ENSMMUT00000024090	ENSCJAT00000036360	ENSMUST00000026924	ENSMICT00000001732	ENSPPYT00000003411	ENSOPRT00000007229	ENSOCUT00000009298	ENSRNOT00000065978	ENSSTOT00000016476	ENSTBET00000013127	ENSTSYT00000000058
ENST00000222598	ENSOGAT00000028988	ENSPTRT00000035948	ENSBTAT00000024811	ENSNLET00000020395	ENSGGOT00000004420	ENSCPOT00000001817	ENSDORT00000009582	ENSMMUT00000020926	ENSCJAT00000001087	ENSMUST00000052609	ENSMICT00000014446	ENSPPYT00000020731	ENSOPRT00000001879	ENSOCUT00000008598	ENSRNOT00000014946	ENSSTOT00000005148	ENSTBET00000006079	ENSTSYT00000010485
ENST00000454200	ENSOGAT00000004875	ENSPTRT00000041137	ENSBTAT00000007524	ENSNLET00000009573	ENSGGOT00000022709	ENSCPOT00000003700	ENSDORT00000005787	ENSMMUT00000047899	ENSCJAT00000053878	ENSMUST00000033608	ENSMICT00000017916	ENSPPYT00000023962	ENSOPRT00000012770	ENSOCUT00000011199	ENSRNOT00000004909	ENSSTOT00000005450	ENSTBET00000017366	ENSTSYT00000012655
ENST00000553490	ENSOGAT00000002699	ENSPTRT00000011983	ENSBTAT00000029271	ENSNLET00000020716	ENSGGOT00000027320	ENSCPOT00000001153	ENSDORT00000014855	ENSMMUT00000009192	ENSCJAT00000040193	ENSMUST00000021668	ENSMICT00000006008	ENSPPYT00000007075	ENSOPRT00000011665	ENSOCUT00000014065	ENSRNOT00000016076	ENSSTOT00000007980	ENSTBET00000011365	ENSTSYT00000000378
ENST00000324439	ENSOGAT00000033052	ENSPTRT00000030102	ENSBTAT00000009461	ENSNLET00000010183	ENSGGOT00000027085	ENSCPOT00000027794	ENSDORT00000003938	ENSMMUT00000012834	ENSCJAT00000039224	ENSMUST00000031345	ENSMICT00000009476	ENSPPYT00000017246	ENSOPRT00000012373	ENSOCUT00000000363	ENSRNOT00000003470	ENSSTOT00000012945	ENSTBET00000011395	ENSTSYT00000013137
ENST00000039007	ENSOGAT00000008769	ENSPTRT00000040474	ENSBTAT00000001947	ENSNLET00000001516	ENSGGOT00000011749	ENSCPOT00000013020	ENSDORT00000014051	ENSMMUT00000018941	ENSCJAT00000040856	ENSMUST00000049910	ENSMICT00000011722	ENSPPYT00000023616	ENSOPRT00000011367	ENSOCUT00000010529	ENSRNOT00000004686	ENSSTOT00000004054	ENSTBET00000009277	ENSTSYT00000000488
ENST00000368603	ENSOGAT00000016964	ENSPTRT00000002504	ENSBTAT00000019554	ENSNLET00000013760	ENSGGOT00000005630	ENSCPOT00000013021	ENSDORT00000011769	ENSMMUT00000045911	ENSCJAT00000053858	ENSMUST00000107369	ENSMICT00000010313	ENSPPYT00000000959	ENSOPRT00000003978	ENSOCUT00000011782	ENSRNOT00000032062	ENSSTOT00000014523	ENSTBET00000012486	ENSTSYT00000004182
ENST00000242719	ENSOGAT00000011685	ENSPTRT00000073171	ENSBTAT00000017277	ENSNLET00000020703	ENSGGOT00000004941	ENSCPOT00000024997	ENSDORT00000000218	ENSMMUT00000016957	ENSCJAT00000042405	ENSMUST00000030284	ENSMICT00000008366	ENSPPYT00000001635	ENSOPRT00000007010	ENSOCUT00000007579	ENSRNOT00000063848	ENSSTOT00000008997	ENSTBET00000008137	ENSTSYT00000000115
ENST00000255006	ENSOGAT00000011542	ENSPTRT00000024712	ENSBTAT00000056271	ENSNLET00000012582	ENSGGOT00000030265	ENSCPOT00000013306	ENSDORT00000000391	ENSMMUT00000044407	ENSCJAT00000010362	ENSMUST00000110005	ENSMICT00000006040	ENSPPYT00000012483	ENSOPRT00000009311	ENSOCUT00000015451	ENSRNOT00000064695	ENSSTOT00000014726	ENSTBET00000009581	ENSTSYT00000012190
ENST00000452346	ENSOGAT00000000110	ENSPTRT00000028436	ENSBTAT00000020053	ENSNLET00000005345	ENSGGOT00000003588	ENSCPOT00000003479	ENSDORT00000011252	ENSMMUT00000022600	ENSCJAT00000039747	ENSMUST00000036617	ENSMICT00000009354	ENSPPYT00000015760	ENSOPRT00000009550	ENSOCUT00000009918	ENSRNOT00000045521	ENSSTOT00000001305	ENSTBET00000005991	ENSTSYT00000006215
ENST00000371980	ENSOGAT00000000911	ENSPTRT00000001314	ENSBTAT00000033319	ENSNLET00000021120	ENSGGOT00000032450	ENSCPOT00000021305	ENSDORT00000002625	ENSMMUT00000008182	ENSCJAT00000041441	ENSMUST00000030469	ENSMICT00000003261	ENSPPYT00000001678	ENSOPRT00000016002	ENSOCUT00000005222	ENSRNOT00000035348	ENSSTOT00000009267	ENSTBET00000009659	ENSTSYT00000012832
ENST00000425103	ENSOGAT00000007097	ENSPTRT00000029665	ENSBTAT00000025878	ENSNLET00000015060	ENSGGOT00000015151	ENSCPOT00000014655	ENSDORT00000001831	ENSMMUT00000023891	ENSCJAT00000059251	ENSMUST00000087545	ENSMICT00000007938	ENSPPYT00000016950	ENSOPRT00000001156	ENSOCUT00000017302	ENSRNOT00000029191	ENSSTOT00000015136	ENSTBET00000004124	ENSTSYT00000004582
ENST00000239849	ENSOGAT00000024177	ENSPTRT00000010716	ENSBTAT00000011745	ENSNLET00000002125	ENSGGOT00000011230	ENSCPOT00000004708	ENSDORT00000013741	ENSMMUT00000025120	ENSCJAT00000034332	ENSMUST00000110858	ENSMICT00000017493	ENSPPYT00000006297	ENSOPRT00000001739	ENSOCUT00000011049	ENSRNOT00000049056	ENSSTOT00000010099	ENSTBET00000011247	ENSTSYT00000011161
ENST00000312504	ENSOGAT00000004043	ENSPTRT00000023893	ENSBTAT00000024690	ENSNLET00000009204	ENSGGOT00000016161	ENSCPOT00000013649	ENSDORT00000010438	ENSMMUT00000000557	ENSCJAT00000022296	ENSMUST00000053922	ENSMICT00000013878	ENSPPYT00000015281	ENSOPRT00000015014	ENSOCUT00000014296	ENSRNOT00000033459	ENSSTOT00000011928	ENSTBET00000006593	ENSTSYT00000001465
ENST00000220822	ENSOGAT00000006881	ENSPTRT00000037688	ENSBTAT00000016739	ENSNLET00000002157	ENSGGOT00000002848	ENSCPOT00000002309	ENSDORT00000006224	ENSMMUT00000031412	ENSCJAT00000001038	ENSMUST00000026879	ENSMICT00000000673	ENSPPYT00000021796	ENSOPRT00000013731	ENSOCUT00000007699	ENSRNOT00000007994	ENSSTOT00000004091	ENSTBET00000002194	ENSTSYT00000010182
ENST00000223528	ENSOGAT00000028215	ENSPTRT00000039243	ENSBTAT00000013538	ENSNLET00000022067	ENSGGOT00000005650	ENSCPOT00000009637	ENSDORT00000012879	ENSMMUT00000003802	ENSCJAT00000032797	ENSMUST00000128667	ENSMICT00000001712	ENSPPYT00000022708	ENSOPRT00000013817	ENSOCUT00000011819	ENSRNOT00000036799	ENSSTOT00000010263	ENSTBET00000017556	ENSTSYT00000003308
ENST00000326291	ENSOGAT00000009872	ENSPTRT00000010883	ENSBTAT00000046767	ENSNLET00000020523	ENSGGOT00000011540	ENSCPOT00000007260	ENSDORT00000007297	ENSMMUT00000001155	ENSCJAT00000041572	ENSMUST00000022650	ENSMICT00000011935	ENSPPYT00000006411	ENSOPRT00000015912	ENSOCUT00000006671	ENSRNOT00000036350	ENSSTOT00000000313	ENSTBET00000008134	ENSTSYT00000005188
ENST00000374279	ENSOGAT00000031498	ENSPTRT00000039304	ENSBTAT00000043826	ENSNLET00000020952	ENSGGOT00000034010	ENSCPOT00000009264	ENSDORT00000006031	ENSMMUT00000030297	ENSCJAT00000034407	ENSMUST00000030074	ENSMICT00000009763	ENSPPYT00000022746	ENSOPRT00000003435	ENSOCUT00000014364	ENSRNOT00000021110	ENSSTOT00000002159	ENSTBET00000011694	ENSTSYT00000008450
... (truncated at 1000 rows)

## Sheet: Sheet2

## Sheet: Sheet3
